# In situ electron microscopy of LaNiO_3_ transformation during reduction and methane dry reforming

**DOI:** 10.1038/s41467-026-76419-1

**Published:** 2026-08-07

**Authors:** Pengfei Cao, Christoph Malleier, Dylan Jennings, Leander Haug, Maged F. Bekheet, Joachim Mayer, Rafal E. Dunin-Borkowski, Simon Penner, Marc Heggen

**Affiliations:** 1 Ernst Ruska-Centre for Microscopy and Spectroscopy with Electrons (ER-C), Forschungszentrum Jülich GmbH, Jülich, Germany; 2 Institute for a sustainable Hydrogen Economy (IHE-1), Forschungszentrum Jülich GmbH, Jülich, Germany; 3 Department of Physical Chemistry, University of Innsbruck, Innsbruck, Austria; 4 Institute of Energy Materials and Devices, Materials Synthesis and Processing (IMD-2), Forschungszentrum Jülich GmbH, Jülich, Germany; 5 Faculty III Process Sciences, Institute of Materials Science and Technology, Chair of Advanced Ceramic Materials, Technische Universität Berlin, Berlin, Germany; 6 Central Facility for Electron Microscopy (GFE), RWTH Aachen University, Aachen, Germany; 7 Present Address: Center for Interface Dominated High Performance Materials, Advanced Transmission Electron Microscopy (ATEM), Ruhr-University-Bochum, Bochum, Germany

**Keywords:** Nanoparticles, Materials for energy and catalysis

## Abstract

By in situ electron microscopy we show the intermediate phase formation during decomposition of LaNiO_3_, a model methane dry reforming (DRM) catalyst, under different gas atmospheres. By combining dark- and bright-field imaging with secondary-electron contrast and operando electronic structure characterization, we localize the coupled formation of Ni(O) particles and La_2_NiO_4_ as key intermediates under DRM operation preceding full decomposition into metallic Ni and La_2_O_3_. Reduced and bar-level reactant partial pressures enable detailed observation of early decomposition stages. Ni particles formed in vacuum or DRM mixtures undergo transient surface oxidation by lattice oxygen, while highly dynamic Ni particles result from hydrogen reduction. At high temperatures, concurrent Ni formation, LaNiO_3_-to-La_2_NiO_4_ transitions, Ostwald ripening, and particle fragmentation/agglomeration govern the dynamics. DRM exposure of reduction-formed Ni leads to rapid oxidation to sintering-resistant NiO with size-dependent dissolution/agglomeration characteristics. As CO_2_ cannot directly oxidize Ni here, oxygen migration from the perovskite bulk drives this transient, DRM-detrimental NiO formation.

## Abstract (web summary)

This study presents the decomposition of LaNiO3, a model methane dry reforming catalyst using in situ electron microscopy. Intermediate phase formation is monitored on the atomic scale under different gas atmospheres and oxygen partial pressure regimes.

## Introduction

Methane dry reforming (DRM) is a viable method of converting the two harmful anthropogenic greenhouse gases, carbon dioxide and methane, into a useful hydrogen–carbon monoxide mixture (syngas)^1^. The latter can be used to produce a variety of useful synthetic chemicals, including methanol, synthetic natural gas, liquefied petroleum gas, methane, Fischer-Tropsch products, and aldehyde oxo-synthesis products^2^. Catalyst development in DRM, which aims to understand and suppress coke formation from reactant activation, requires a thorough control and understanding of the catalyst structure and morphology at the atomic level under close-to-real operational conditions^3,4^.

Due to their thermal (in)stability and outstanding solid-state properties, perovskite materials have emerged as particularly promising candidates for overcoming the drawbacks of conventional supported Ni catalysts in DRM^4^. One such property is the use of perovskites as precursor materials for a controlled decomposition into a metal-oxide or metal-perovskite interface with improved catalytic and sintering properties via the formation or exsolution of metal particles from the perovskite lattice. In situ exsolution strategies have been widely studied for such perovskite-type complex oxides under reducing conditions, but less so directly in catalytic gas mixtures^5–12^. These approaches have enabled the use of many transition and/or earth alkaline metals at the respective A or B perovskite lattice sites. Successful preparation of active oxide- or perovskite-supported metal and/or alloy nanoparticles includes both noble and transition metals^5,9^.

In recent decades, in situ electron microscopy has provided essential insight into various aspects of heterogeneous catalysis and related exsolution phenomena. This includes in situ/operando observation of catalyst synthesis, particle size redistribution during catalytic reactions, surface reconstruction, elemental composition and phase evolution, and the evolution of the shape and morphology of catalyst particles. It also includes the adsorption of reactant gases, catalyst particle reconstruction in correlation with gas product analysis, and spatial and temporal monitoring of catalytic reactions^13–19^.

In situ transmission electron microscopy (TEM) provides insight into the structural behavior of perovskites under reducing atmospheres, allowing one to monitor and characterize the structural and morphological changes of particle formation at the atomic scale^9,11,12,20^. Examples include, e.g., monitoring the exsolution and growth of Co nanoparticles from Co-doped Pr_0.5_Ba_0.5_Mn_0.9_Co_0.1_O_3_ and SrTi_0.75_Co_0.25_O_3-δ_ perovskites^11,20^, following the spatial and temporal exsolution of individual nanoparticles from La_0.43_Ca_0.37_Ni_0.06_Ti_0.94_O_3_^12^, or studying the formation and exsolution of Ni particles from the archetypal LaNiO_3_ material. This reveals oxygen spillover from the perovskite to Ni particles, providing insight into the catalytic activity of Ni particles formed from decomposing LaNiO_3_ during DRM^21^.

In the LaNiO_3_/La_2_NiO_4_ system in particular, the formation and properties of Ni particles, the stability of the perovskite, and the pathway of perovskite decomposition depend strongly on the reduction potential of the reducing agent^22–25^. In situ electron microscopy vacuum treatments revealed that although Ni particles form locally, the remaining perovskite structure essentially remains intact^21^. In contrast, in situ X-ray diffraction during hydrogen reduction at elevated temperatures confirmed the total collapse of the perovskite structure, resulting in Ni^0^ nanoparticles forming in contact with La_2_O_3_^23^. Thus, this system can be switched from a metal-perovskite to a metal-oxide system simply by adjusting the reduction potential of the gas atmosphere. Important for DRM applications, direct treatment in CO_2_/CH_4_ mixtures steers the transformation of LaNiO_3_ over a sequence of intermediary phases, also towards a Ni-La_2_O_3_ interface. However, the extent and physicochemical properties of the interface differ due to the different sizes of the Ni particles^23^. Due to the relatively more oxidizing conditions of a DRM atmosphere compared to pure hydrogen, thermodynamics allows for the detection of transient phases, such as La_2_NiO_4_, en route to full perovskite decomposition^23,25^.

The formation of Ni particles from LaNiO_3_ perovskites is either a reduction-induced or a self-activated step, both initializing the catalytic DRM performance^22^. The missing link in fully understanding the decomposition process and the catalytic action of LaNiO_3_ is studying the early stages of its partial decomposition into LaNiO_3_, Ni particles, and La_2_NiO_4_. The simultaneous emergence of the latter two raises questions about beneficial Ni particle size and distribution, as well as the control of Ni particle dynamics and its effect on DRM activity. Additionally, vacuum heating and catalytic DRM experiments have indicated that the intermediate oxidation of emerging Ni particles by reactive lattice oxygen from LaNiO_3_ profoundly affects the catalytic action. To address these issues, it is imperative to separate thermal, purely reduction-induced, and DRM-triggered structural and morphological changes. The main goal, therefore, is to monitor and reveal the different pathways of Ni formation from the perovskite in relation to the reduction potential of the gas atmospheres using a variety of in situ and operando methods. This will be combined with dedicated DRM experiments to establish a relationship between structure, morphology, electronic structure, and catalytic properties. We will rely on the unique ability to combine bulk and surface characterization by in situ aberration-corrected environmental scanning transmission electron microscopy under DRM conditions, and in a hydrogen atmosphere. This will enable us to simultaneously capture atomically-resolved dark-field (DF), bright-field (BF), and high-resolution secondary electron (SE) images, together with energy-dispersive X-ray spectroscopy (EDX) measurements to monitor the bulk structural, compositional, and surface morphological changes of the samples. Complementary methods include operando X-ray photoelectron spectroscopy, operando X-ray diffraction, and dedicated DRM experiments. A crucial point for the latter is ensuring that the established structure-activity correlation can be transferred from the model low-Pa pressure conditions to bar-level DRM catalysis. In other words, the catalytic “pressure-gap” must be properly overcome.

## Results

LaNiO_3_ is one of the most promising model perovskites in DRM due to its excellent activity and coking resistance. This resistance is related to the exsolution/formation of Ni and the phase reconstruction of the parent perovskite host into La_2_NiO_4_. This process is followed by the subsequent anchoring of the Ni particles within the La_2_O_3_ matrix formed after full perovskite decomposition^26^. To fully structurally understand the early stages of this process and to highlight the eventual dynamics of the emerged Ni particles, as well as their potential impact on catalytic DRM operation, the formation of Ni from LaNiO_3_ was investigated in atmospheres with different reduction strengths, as well as in a CO_2_:CH_4_ mixture. This was done to distinguish between thermal, reduction-, and reaction-induced effects.

### Activation of LaNiO_3_ under hydrogen reduction conditions

#### Temperature-dependent formation of Ni particles

**Fig. 1:**
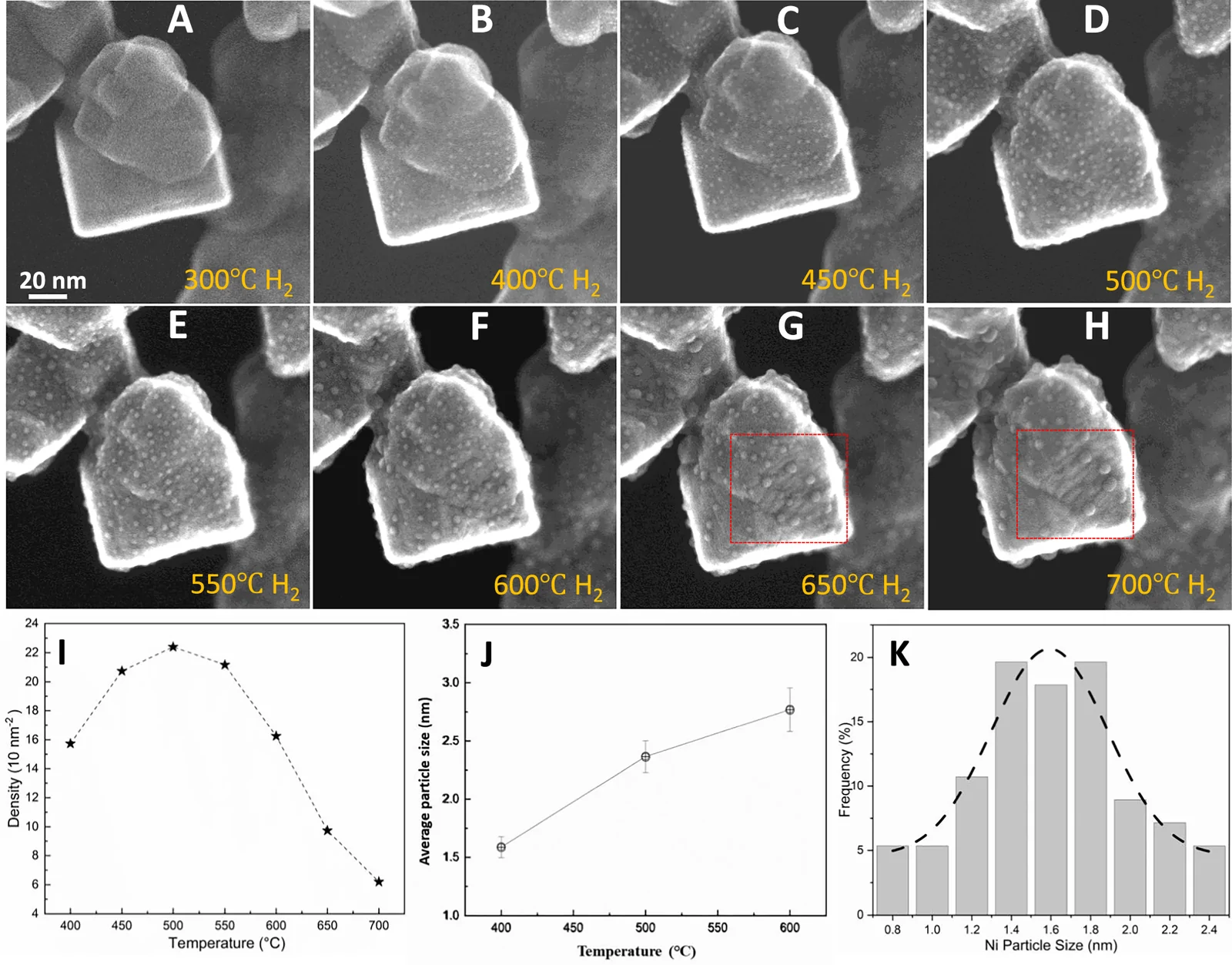
Temperature-dependent evolution of LaNiO_3_ and Ni particle formation during hydrogen reduction. STEM-SE imaging was performed at selected temperatures between 300 and 700 °C (**A**–**H**) as indicated, and these panels share a scale bar of 20 nm. The red dashed squares indicate the formation of a roughened stepped surface. **I** The Ni particle density as a function of temperature during H_2_ reduction. **J** The average particle size analysis of the Ni particles formed on the LaNiO_3_ surface as a function of temperature during the reduction (error bar represents the standard deviation of the measurement). **K** The Ni particle size distribution at 400 °C.

Since hydrogen reduction of Ni-based perovskites prior to the DRM reaction boosts their activity, we have at first focused on in situ monitoring of the Ni formation from LaNiO_3_ during hydrogen treatments. Figure 1 shows a series of secondary electron (SE) images that illustrate the temperature-dependent dynamics of Ni exsolution from 300 to 700 °C in 1.5 Pa H_2_. Ni particles emerge at 400 °C with a narrow, high-density particle size distribution with a maximum particle size of around 1.6 nm (Fig. 1I). The small size and high-density distribution of the Ni particles already indicate a potentially high catalytic performance during DRM^27^.

Up to a temperature of ~550 °C, the size and density of the Ni particles barely change (Fig. 1J). Above 550 °C, Ostwald ripening causes Ni particles to sinter, resulting in larger particle sizes and a decreased particle density (Fig. 1K). Additionally, the surface of selected areas roughens and features a pronounced step structure, which starts to appear at around 450 °C (Fig. S1) and becomes more pronounced at 550 °C. Around 650 °C, SE images also feature the stepped surface structure inside the red dashed square (Fig. 1G, H), implying a pronounced structural change of the host material accompanying Ni emergence.

**Fig. 2:**
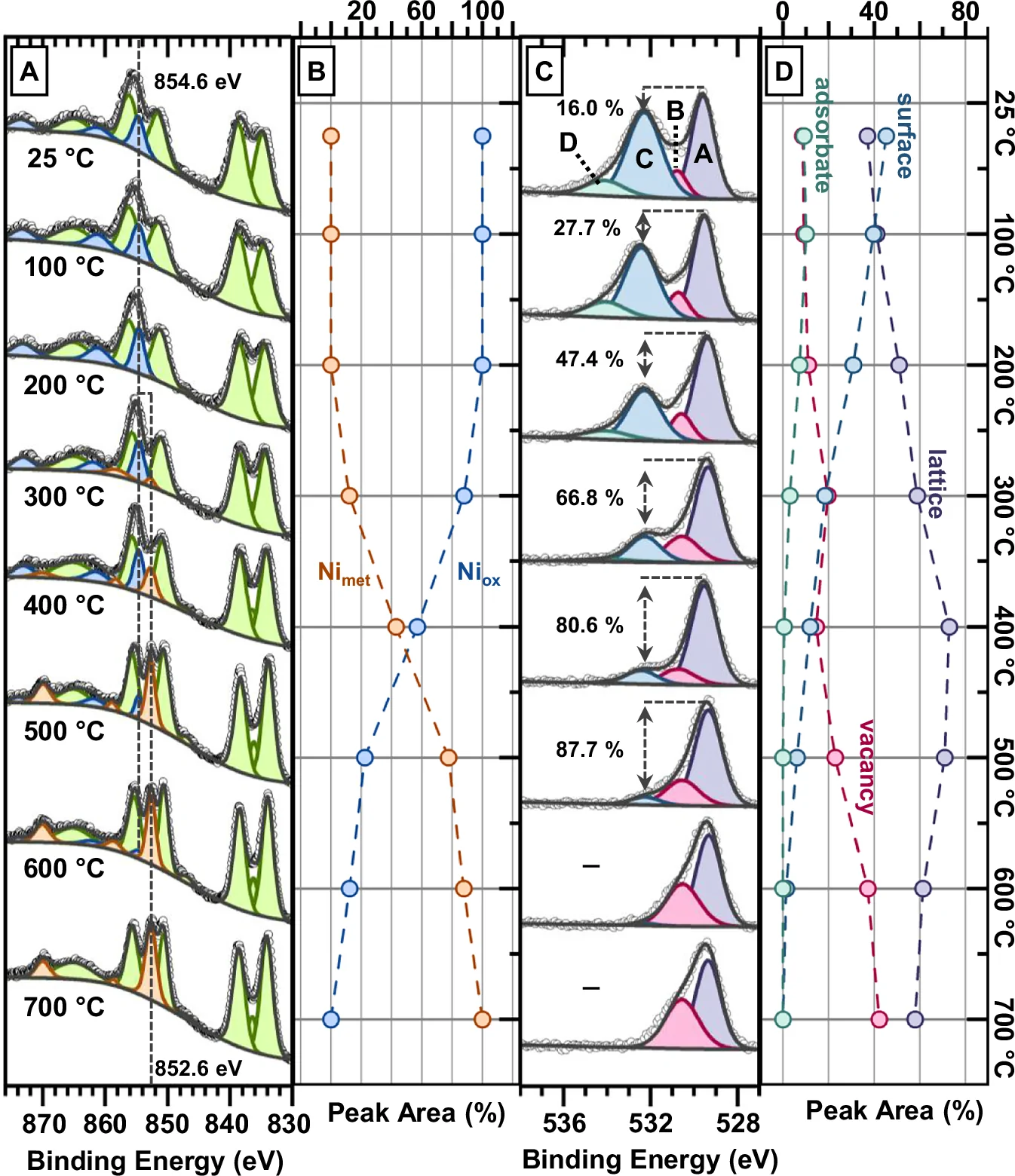
Temperature-resolved operando XPS investigation of LaNiO_3_ during hydrogen reduction (300–700 °C, 10 Pa). **A** High-resolution Ni 2p and La 3d regions. The La region is shown in green, oxidized Ni in blue, and metallic Ni in orange. **B** Metallic vs. oxidic Ni evolution. **C** O 1s components: A—lattice (purple); B—vacancy (pink); C—surface (blue); D—oxygenate species (green). The relative intensity differences between bulk and surface oxygen species are indicated. **D** Evolution of oxygen species. See the “Methods” section for details on the fitting procedure. The evolution of the mass spectra is shown in Fig. S2.

As heating under a hydrogen atmosphere using STEM only provides localized insight, we performed complementary operando XPS studies at 10 Pa H_2_ to correlate the onset of nickel formation with the appearance of Ni particles on the LaNiO_3_ surface in SE images (Fig. 2). With an information depth below 5 nm for La *3d*, Ni *2p*, and O *1s* photoelectrons, the spectra probe near-surface transformations. Up to 300 °C, the La *3d*_*5/2*_ and *3d*_*3/2*_ line shapes remain characteristic for LaNiO_3_, and Ni is present exclusively in its oxidized Ni^3+^ state (Ni *2p*_*3/2*_ at 854.6 eV)^28^. Metallic Ni appears first on the surface at 300 °C as seen in the Ni *2p*_*3/2*_ peak at 852.6 eV (Fig. 2A), in agreement with STEM-SE observations. Upon heating to 700 °C, the Ni^0^ *2p*_*3/2*_ contribution increases markedly, indicating progressive reduction and an increase in the amount of Ni particles. Within the XPS probe depth, Ni is fully reduced at 700 °C. Concomitantly, systematic changes in the La *3d* data between 300 and 700 °C reveal a structural transformation of the host lattice. The evolution of the La *3d*_*5/2*_ and La *3d*_*3/2*_ line shapes and intensity ratios—accounting for overlap with Ni *2p*—reveals a transition from LaNiO_3_ to La_2_NiO_4_^29^. The relative intensity ratio of the La *3d*_*3/2*_ peak at 851.3 eV and 856.1 eV switches at progressively higher temperatures beyond 550 °C, and resembles that of La_2_O_3_ at 700 °C^30^. As the La *3d*_*3/2*_ peak strongly overlaps with the *Ni 2p* peak, we based our interpretation also on the corresponding La *3d*_*5/2*_ area. The shape of the La *3d*_*5/2*_ peaks also shifts from a shape typically found in LaNiO_3_ at 300 °C^31^ to one similar to that of La_2_O_3_ at 700 °C^30^. Benchmark measurements (Fig. S2) confirm this assignment, with characteristic inversion of La *3d*_*5/2*_ and La *3d*_*3/2*_ peak intensities and the emergence of a broad plasmon feature at 848.5 eV. Analogous trends are observed in the O *1s* region. In due course, the formation of oxygen vacancies under a reducing atmosphere likely leads to charge compensation, inducing a Jahn-Teller lattice distortion^32^. The O *1s* spectra further evidence progressive oxygen depletion, characterized by the removal of surface-bound oxygen species (species “C”), and the concomitant formation of vacancy-associated oxygen (species “B”). Notably, the overall intensity of the O *1s* signal decreases substantially at higher hydrogen reduction temperatures, indicating that the surface-near layers of LaNiO_3_ become increasingly devoid of oxygen. This is corroborated by simultaneous mass spectrometry profiles, where both H_2_ consumption (*m*/*z* = 2) and H_2_O formation (*m*/*z* = 18) follow the reaction ${{{\rm{H}}}}_{2}+{{\rm{O}}}\to {{{\rm{H}}}}_{2}{{\rm{O}}}\,$ synchronously (Fig. S3).

We have previously demonstrated through in situ XRD under ambient hydrogen conditions that LaNiO_3_ rapidly transforms to metallic Ni particles and La_2_O_3_ via oxygen-deficient LaNiO_2.7_ and LaNiO_2.5_, without the intermediate presence of La_2_NiO_4_. La_2_NiO_4_ is only present in less reducing atmospheres (i.e., higher oxygen partial pressures)^33^, such as those under typical DRM conditions. In contrast, the in situ STEM-SE imaging, due to the lower hydrogen partial pressures, allows us to thermodynamically stabilize the La_2_NiO_4_ intermediate structure along the reduction pathway. This enables us to distinguish it structurally and morphologically from the LaNiO_3_ matrix and to monitor its role in the formation of the Ni particles. La_2_NiO_4_ is subsequently unstable under hydrogen reduction itself and eventually decomposes into metallic Ni and La_2_O_3_^33^.

In order to address the critical conceptual gap regarding Ni particle formation during (partial) LaNiO_3_ decomposition, we must distinguish between true exsolution and solid-state recrystallization effects. True exsolution often uses non-stoichiometric perovskite formulations to trigger Ni exsolution along established crystallographic alignment relationships while maintaining the original parent perovskite host lattice. Recrystallization, on the other hand, results in complete lattice restructuring, thereby creating new socket sites with altered anchoring and interfacial properties^34,35^. While the latter is certainly true at more advanced states of LaNiO_3_ decomposition, where La_2_NiO_4_ begins to form alongside simultaneous Ni particle formation and ultimately collapses to form La_2_O_3_ and Ni, we have recently shown^36^ that in the early stages of hydrogen reduction, where no La_2_NiO_4_ is formed and the redox chemistry is dominated by oxygen removal to form bulk oxygen-deficient LaNiO_2.7_ and LaNiO_2.5_, true exsolution of Ni occurs, leading to surface-bound Ni particles. That said, the type of Ni particle formation observed here is more likely associated with bulk recrystallization.

#### Dynamics of emerged Ni nanoparticles and structural evolution of perovskites in H_2_

**Fig. 3:**
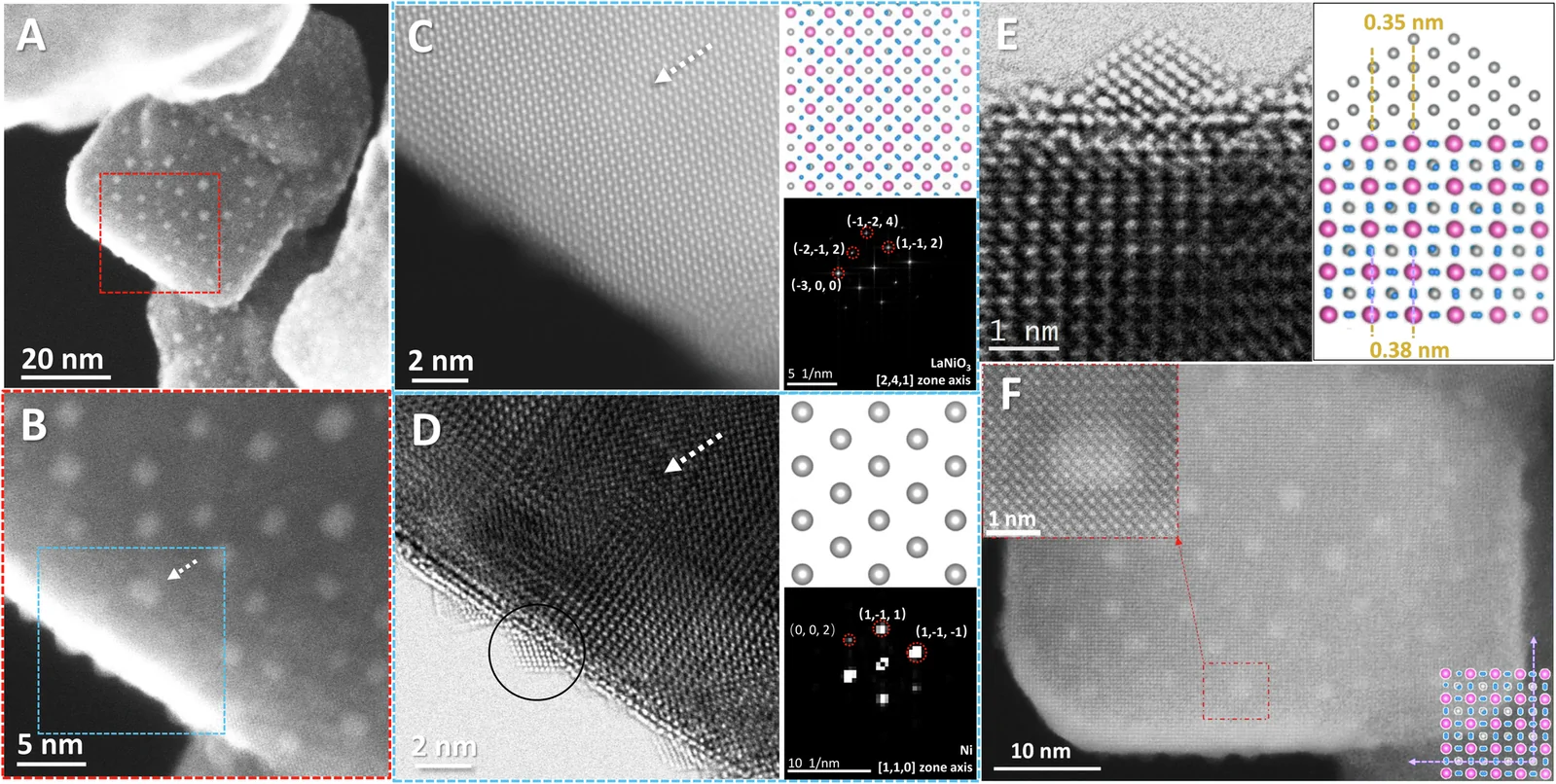
Structural analysis of the onset of surface-bound Ni particle formation on LaNiO_3_ after hydrogen reduction at 400 °C. **A** STEM-SE image of Ni particles and LaNiO_3_ host. **B** Enlarged SE image of the region in the red dashed box. **C** DF image of the region of the blue dashed box in (**B**) with crystal structure model and FFT patterns corresponding to LaNiO_3_. **D** BF image of region within the blue dashed box in (**B**) with crystal structure model and FFT patterns corresponding to the Ni particles at the surface. The white arrows indicate the same position of a representative Ni particle in SE, DF, and BF images, respectively. **E** Atomically-resolved BF image with a Ni particle emerged from the host material, and a scheme demonstrating the epitaxial growth relationship between the Ni particle and host material with a 8 % lattice mismatch. **F** Overview image and enlarged inset showing atomically-resolved STEM-SE images of Ni particles on the LaNiO_3_ host. For the crystal structure models, Ni, O, and La atoms are represented by atomic spheres in silver, blue, and pink, respectively.

Because the potential dynamics of the emerging Ni particles significantly impact their size and distribution and enable sintering control, we directly investigated these processes using in situ electron microscopy. Figures 3 and S4 show atomically resolved BF, DF, and SE images recorded simultaneously at 400 °C, when the Ni particles start to emerge. The corresponding fast-Fourier transform (FFT) patterns (Fig. 3C, D) confirm that the emerged metallic Ni particles are oriented along the $\left[110\right]$, and the LaNiO_3_ matrix oriented along the $\left[241\right]$ zone axis. Compared to pristine LaNiO_3_, LaNiO_3_ at 400 °C retains its perovskite structure, although the $(\bar{2}\bar{1}2)$ lattice plane distance is slightly compressed (Fig. S5). We relate this minor structural change to the partial reduction of Ni^3+^ in LaNiO_3_ (as evidenced by the XP spectra discussed in Fig. 2)^37^. As seen in Fig. 3B, a 2-nm Ni particle is visible in the SE image, but is not yet observed in the corresponding enlarged DF or BF images (see the arrow in Fig. 3C, D). This suggests that the Ni particles form in surface-near regions rather than deep within the LaNiO_3_ matrix. Atomic-resolution SE imaging of the those Ni particles at the surface reveals a crystallographic orientation of the Ni lattice along the {2 1 2} or {1 2 4} planes of LaNiO_3_ (Fig. 3F). Figure 3E demonstrates in the cross-section the coherent growth relationship of the Ni particles to the LaNiO_3_ matrix, showing the influence of lattice constraint from the host material on the growth of Ni particles. Accordingly, Ni exsolves from the LaNiO_3_ lattice structure through surface nucleation and epitaxial growth of Ni nanoparticles.

**Fig. 4:**
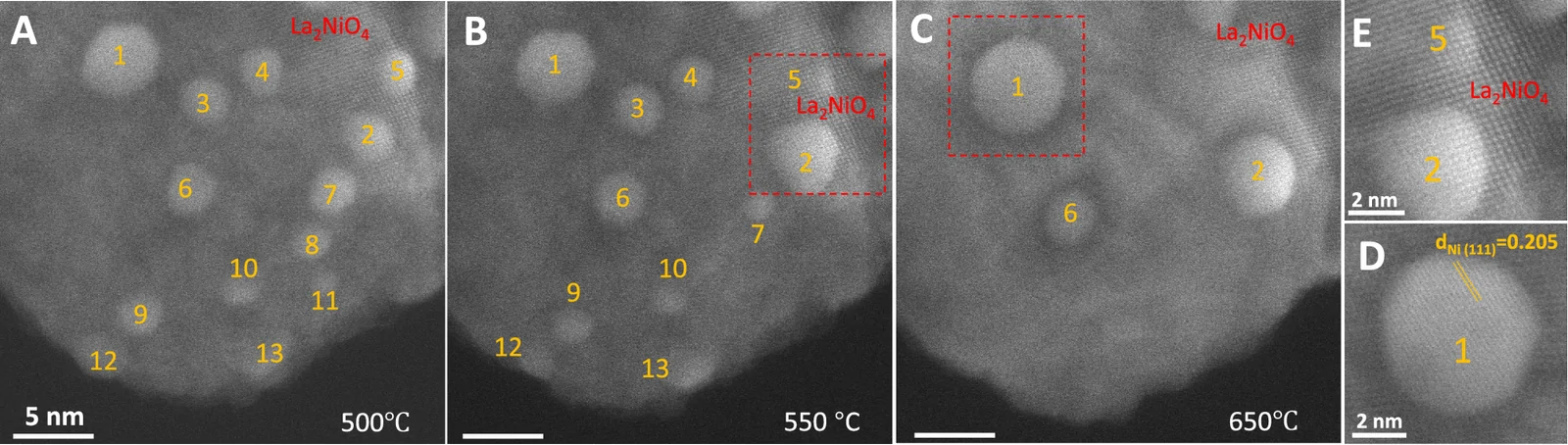
Localized dynamic coalescence behavior of Ni nanoparticles during in situ hydrogen reduction. STEM-SE imaging has been performed at selected temperatures at 500 °C (**A**), 550 °C (**B**), and 650 °C (**C**), and three panels share a scale bar of 5 nm. **D**, **E** Atomically-resolved STEM images of the isolated Ni particle “1” in the red frames of (**B**, **D**).

At temperatures above 550 °C, structural changes in relation to the host matrix and the morphology of the emerging Ni particles become evident. Smaller particles (labeled as 4–13 in Fig. 4) progressively shrink in size and eventually disappear, while the larger particles (labeled 1 and 2 in Fig. 4) continuously grow. We have performed a quantitative analysis of the particle size to estimate the volume of Ni particles at 500 and 650 °C (Table S1). The analysis shows that the combined volume of particles 1, 2, and 6 at 650 °C is 210.8 nm³, which is greater than their combined volume at 500 °C. This indicates fragmentation of particles 3, 4, 5, and 7–13, as well as mass transfer into particles 1 and 2 by annealing-driven Ostwald ripening (OR)^38^. The observed increase in volume is likely due to the precipitation or formation of additional nickel particles.

**Fig. 5:**
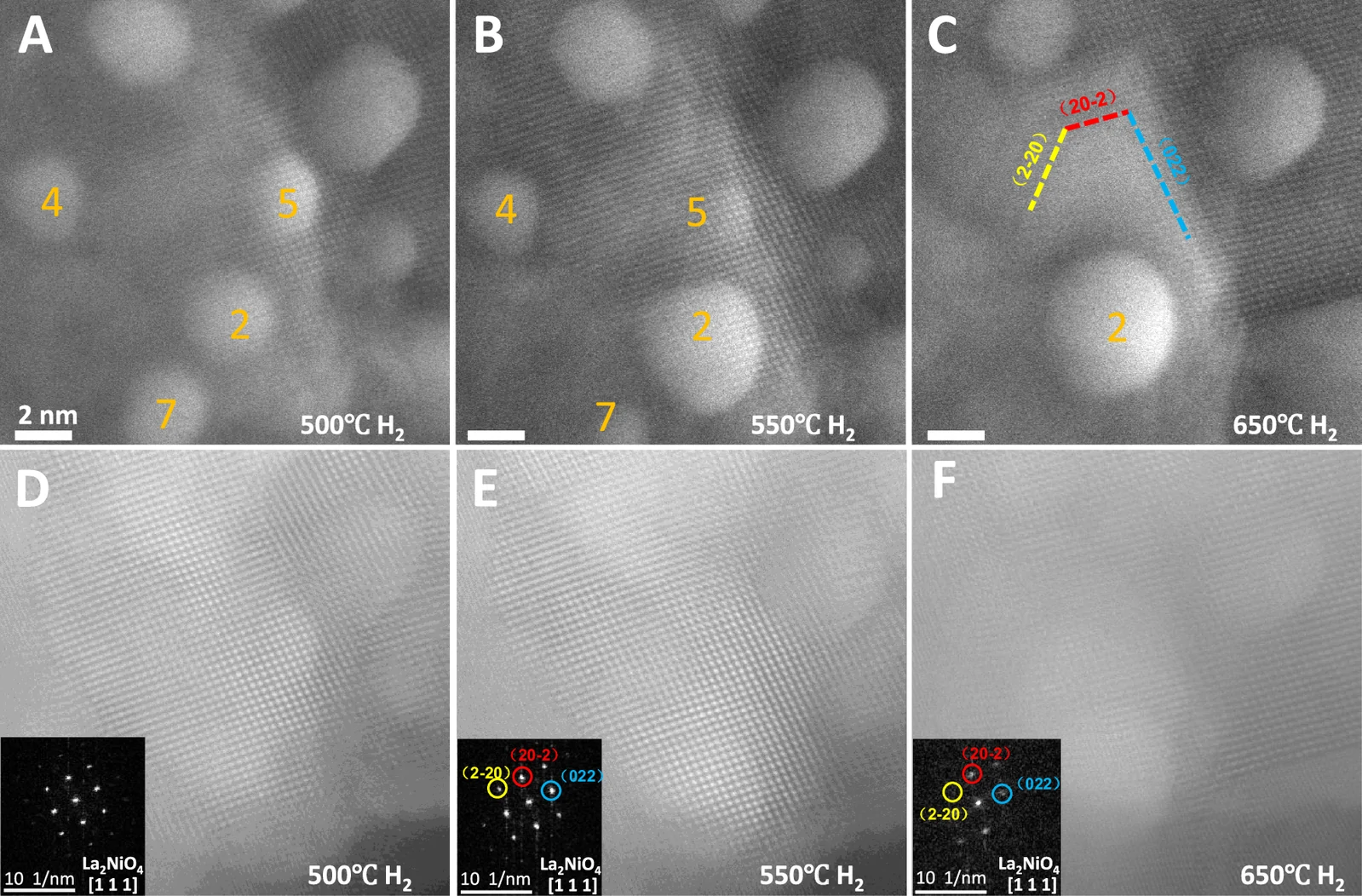
Local phase and structure analysis of LaNiO_3_ during in situ hydrogen reduction at selected temperatures to detect neighboring La_2_NiO_4_ and Ni particles. High-resolution SE imaging was performed at 500 °C (**A**), 550 °C (**B**), and 650 °C (**C**). Corresponding high-resolution DF images and the corresponding FFT patterns were recorded at 500 °C (**D**), 550 °C (**E**), and 650 °C (**F**). To verify the presence of La_2_NiO_4_ in the platelet-like region of (**C**), we have added colored lines as a guide to the eye.

The Ruddlesden–Popper (RP) phase La_2_NiO_4_ is important for the LaNiO_3_ activation step and is identified via the corresponding FFT patterns of the DF images acquired simultaneously (Fig. 5). Supplementary Movie 1 and the correlated snapshots in Fig. S6 directly display the dynamic OR process in real time. The coexistence of Ni and La_2_NiO_4_ (Fig. 4D, E) provides evidence of a phase transition from layered LaNiO_3_ to RP-La_2_NiO_4_ accompanied by additional Ni formation. Compared to in situ XRD, in which LaNiO_3_ began to transform into La_2_NiO_4_ at around 760 °C, these results suggest that the presence of H_2_ accelerates the decomposition of perovskites at lower temperatures. Interestingly, as the temperature increased from 500 °C, where La_2_NiO_4_ first appears, to 650 °C, SE images (Fig. 5) reveal that the atomic structure of La_2_NiO_4_ becomes more distinct, exhibiting a platelet-like morphology. Colored lines were added in Fig. 5C as a guide to the eye in the respective direct and reciprocal lattices to verify the presence of La_2_NiO_4_. This morphology is consistent with the similar surface features observed in Fig. 1. Since SE imaging is highly surface-specific to a few nanometers, this result indicates a more thorough bulk decomposition of LaNiO_3_ into metallic Ni particles and La_2_NiO_4_. Often Ni particles and La_2_NiO_4_ are found in close proximity (see Figs. 4 and 5), as the formation of La_2_NiO_4_ (via the reaction $2{{\rm{LaNi}}}{{{\rm{O}}}}_{3}+2{{{\rm{H}}}}_{2}\longrightarrow {{{\rm{La}}}}_{2}{{\rm{Ni}}}{{{\rm{O}}}}_{4}+2{{\rm{Ni}}}+2{{{\rm{H}}}}_{2}{{\rm{O}}}$) necessarily is entangled with Ni formation, generating more Ni particles. The mechanistic reason for the transformation of LaNiO_3_ into La_2_NiO_4_ under reductive or high-temperature conditions is the creation of oxygen vacancies that create a lattice distortion to energetically favor the layered arrangement over the 3D perovskite and accordingly force the reconstruction of the cation layers and the formation of RP-La_2_NiO_4_^39^. Previous HRTEM/EELS analysis of the LaNiO_3_ → La_2_NiO_4_ transformation revealed that the formation of Ni particles (which is accompanied by the creation of oxygen vacancies and finally, La_2_NiO_4_ formation) prefers to start near stacking faults and grain boundaries, i.e., as the surface roughens during annealing^21^. We therefore suggest that the roughened stepped structure in Fig. S1 acts as a starting point for exactly these transformations.

In addition to fragmentation and ripening, the migration of Ni particles is also observed in Fig. S7 and Supplementary Movie 2. There, the Ni particles undergo stretching and fragmentation into smaller entities that move and merge with other particles. These observations suggest that multiple concurrent mechanisms, including continuous Ni formation, LaNiO_3_ → La_2_NiO_4_ phase transitions, OR processes, and particle migration, all drive the dynamic changes of the surface-bound Ni particles at temperatures above 550 °C.

The morphology of the Ni particles becomes increasingly unstable, and the fragmentation and ripening are more pronounced at 700 °C (Fig. S8 and Supplementary Movie 2). After an isothermal period at 700 °C for 10 min, nearly all of the Ni particles disappear from a surface region approximately 50 nm by 70 nm (Fig. S9). Upon close examination of a representative Ni particle (marked by a white arrow in Fig. S8F), the particle’s complete disappearance leaves behind a region with dark SE contrast that was previously occupied by the Ni particle. Since SE imaging is highly sensitive to the electronic structures of the sample surface^40^, this darker contrast region likely corresponds to lower Ni content compared to the surrounding Ni-richer surface areas. This observation indicates that enhanced Ni fragmentation and movement at such high temperatures leads to Ni redistribution across a wider area.

### Activation of LaNiO_3_ under DRM conditions

To fully understand the activation of LaNiO_3_ under DRM conditions and its associated catalytic action, we will briefly discuss the thermal effects of vacuum annealing, as the structural and compositional consequences of this process are remarkably similar with respect to La_2_NiO_4_ formation and Ni oxidation.

#### Thermally-induced Ni particle formation and Ni oxidation behavior upon vacuum annealing

**Fig. 6:**
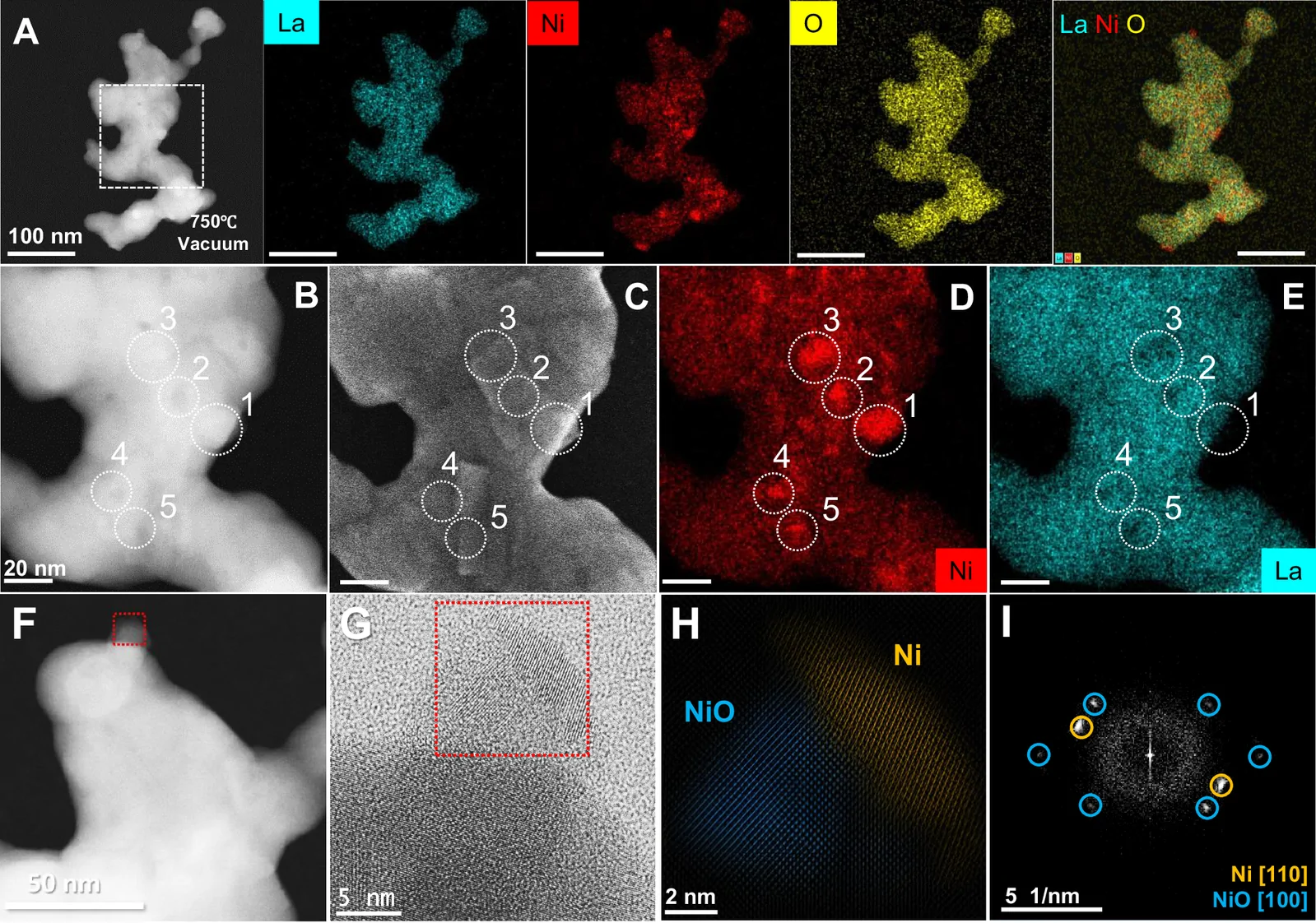
Localized in situ formation of La_2_NiO_4_ and NiO after vacuum heating of LaNiO_3_ at 750 °C. **A** DF STEM image and individual and mixed elemental distribution maps of La–L, Ni–K, and O–K; enlarged DF and SE STEM image (**B**, **C**) of the region in the white frame in (**A**), with local distribution of Ni and La (**D**, **E**). Ni-enriched regions are shown in the white circles 1–5. **F** STEM image of a localized NiO particle with a high-resolution image in (**G**). **H**, **I** FFT analysis of the region within the red frame to verify the simultaneous presence of Ni and NiO in [110] and [100] orientation. The reference state at 200 °C is shown in Fig. S15.

The initial structure of the sample at 200 °C (Fig. S10A) shows a uniform chemical distribution of lanthanum (La), nickel (Ni), and oxygen (O). The surface exhibits no additional small particles. Representative DF and SE image series (Fig. S11) taken at various temperatures revealed that Ni particles, marked by red circles, began to emerge at about 700 °C and grew larger at 750 °C. This finding is further confirmed by EDX results (Fig. 6A). EDX maps after heating to 750 °C show a decrease in La concentration and enrichment in Ni (five areas are marked by white dashed circles in Fig. 6B–E). In some locations, corresponding SE images (Fig. 6C) show an edge-like surface, but no distinct spherical surface features. We have further extracted representative Ni/La atomic ratios for the exsolved Ni-rich regions (Fig. S12). For the regions 01 to 10, the Ni/La atomic ratio is greater than 1, confirming that these regions are Ni-rich. While we cannot determine if the Ni-rich particles are located within the interior or on the reverse side of the substrate, this behavior contrasts with Ni particles formed in H₂, where the emerged Ni particles already predominantly form on the parent perovskite surface. Compared to Ni formation in an H2 atmosphere, the density of Ni particles is significantly lower, while the average particle size is larger.

La_2_NiO_4_ with pronounced facets is observed again in the SE images close to the Ni-enriched features in the white circles in Fig. 6C, D, as well as within the blue rectangles in Fig. S11B–H. EDX analysis (Fig. 6D, E) revealed that the vicinity of faceted La_2_NiO_4_ is Ni-rich (especially visible in area 1 and Fig. S13). Closer examination of area 1 shows that the surface-protruding part contains both Ni and O, and the relatively weak oxygen intensity implies the presence of both Ni and NiO. This is confirmed by the more detailed Ni particle imaged in Fig. 6F, G, where FFT analysis (Fig. 6H) of the boxed region in Fig. 6G corroborates both NiO and Ni. Throughout the heating process, no significant migration of these particles was observed, suggesting that the NiO formation enhances the interaction between the particles and the host material^4,5^.

The temperature at which faceted La_2_NiO_4_ appears (Fig. S10B) matches well with the results of in situ powder X-ray diffraction (PXRD) experiments in He (see discussion related to Fig. S14)^6^. In these experiments, metallic Ni is completely oxidized to NiO by lattice oxygen. These findings suggest that the lattice oxygen in the host matrix is highly mobile and correlated with the associated dynamic formation of oxygen vacancies^26^.

**Fig. 7:**
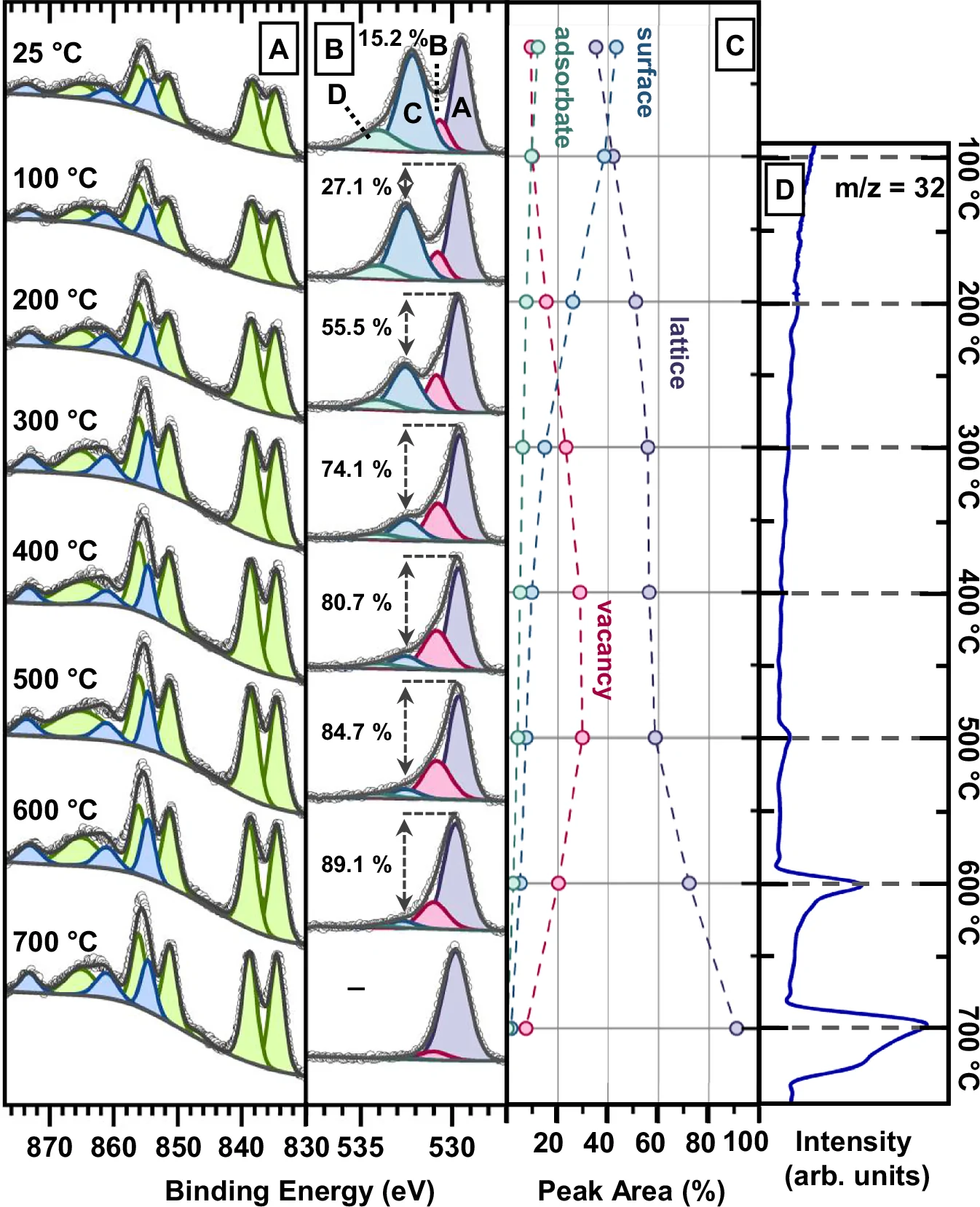
Surface-near oxygen chemistry monitored by operando X-ray photoelectron spectroscopy during vacuum annealing at a base pressure of 10^−6^ Pa. The La *3d*/ Ni *2p* and the O *1s* regions are shown in (**A**, **B**). The La region is shown in green, oxidized Ni in blue. O *1s* assignment: A—lattice (purple); B—vacancy (pink); C—surface (blue); D—oxygenate species (green). **C** Evolution of the individual oxygen components fitted to the O 1s data. The relative intensity difference between bulk and surface oxygen species is indicated. **D** Concomitantly collected mass signal of *m*/*z* = 32. Details of the fitting procedure are discussed in the “Methods” section.

Further evidence of the exclusive participation of reactive lattice oxygen is provided by operando XPS in vacuo annealing experiments (Fig. 7). Between 25 and 700 °C, the La *3d*/ Ni *2p* spectra (Panel A) show no signs of global emergence of surface-bound metallic Ni, closely matching the electron microscopy data in Fig. 6. Above 700 °C, local formation of surface-oxidized Ni particles is observed, but the vast majority remains still buried within the perovskite matrix. The O *1s* spectra (Panels B and C) indicate a sequential evolution of near-surface oxygen chemistry: initial removal of oxygenate species and adsorbed water at low temperatures is followed by depletion of surface-bound oxygen up to 400 °C, and subsequently of vacancy-associated oxygen between 500 and 700 °C (Panel C). Consistently, the *m*/*z* = 32 signal (Panel D) assigned to O_2_ desorption exhibits a pronounced increase at 500, 600, and 700 °C. This behavior is attributed to progressive access to lattice oxygen in deeper layers, which segregates to the surface, recombines, and desorbs as O_2_. Partial reversibility is evidenced by replenishment of oxygen vacancies upon exposure to 20 Pa of O_2_ at 25 °C for 10 min (Fig. S15).

#### Reaction-induced Ni particle formation and oxidation behavior by in situ heating in a 1:1 CO_2_:CH_4_ model DRM mixture

**Fig. 8:**
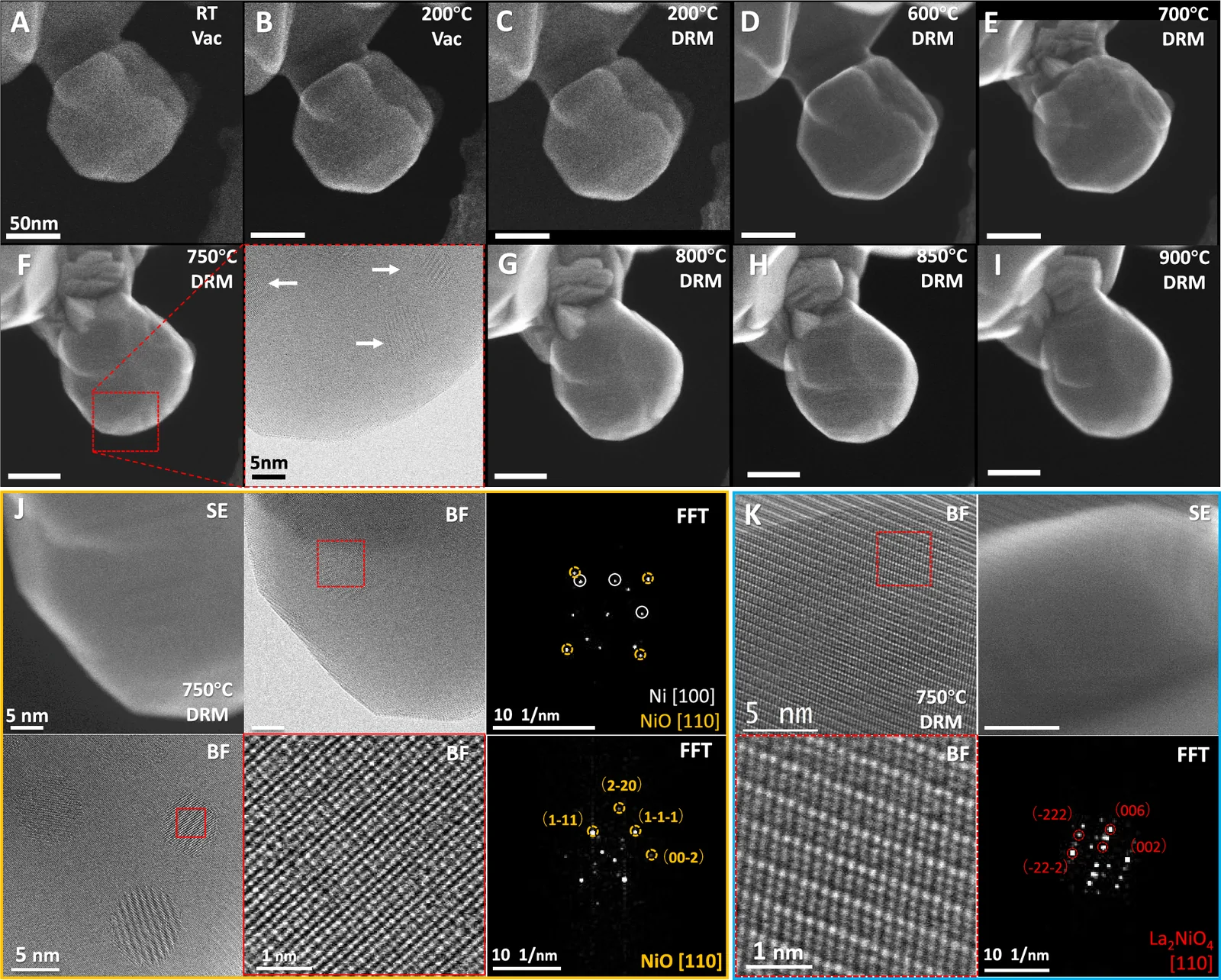
Localized in situ formation of La_2_NiO_4_ and NiO from LaNiO_3_ decomposition in a DRM reaction mixture. Reference SE images at 25 °C (**A**) and at 200 °C in vacuum (**B**). SE images after switching to a CO_2_:CH_4_ = 1:1 DRM mixture at 200 °C (**C**), and after subsequent heating to 600 °C (**D**), 700 °C (**E**), 750 °C (**F**)—an enlarged STEM-BF image of the region indicated by a red dashed lines shows the presence of Ni nanoparticles (white arrows); 800 °C (**G**), 850 °C (**H**), and 900 °C (**I**). **J** SE/BF imaging and the corresponding FFT analysis of sample structure at 750 °C (within the red‑squared regions) to verify the presence of Ni and NiO oriented along the [100] and [110] axes. The representative BF image shows the region with Ni exsolution, enlaged high resolution BF image of the selected area, and its corresponding FFT provides evidence of NiO particles. **K** SE/BF imaging the surface structure formed as a result of phase change at 750 °C, the enlarged BF image of slected region in the red frame, and its corresponding FFT analysis indicating La_2_NiO_4_ oriented along the [110] axis.

As the gas mixture (CO_2_:CH_4_ = 1:1) is introduced at 200 °C (Figs. 8 and S16), LaNiO_3_ maintains its morphology up to around 600 °C. However, a phase and morphological transformation toward faceted La_2_NiO_4_ and Ni particles occurs at 700 °C. The appearance of surface steps can be directly related to the transformation towards these phases (Fig. S16). This transformation closely resembles the reduction (see Fig. 5) and vacuum heating results at comparable temperatures (see Fig. 6). Above 800 °C, the morphological reconstruction continues; however, the number of facets decreases at high temperatures, indicating high mobility. Compared to the reduction in hydrogen, global formation of Ni particles is suppressed, but at 750 °C, localized Ni particle formation, as evidenced by the lattice fringes indicated by arrows, occurs (Panel F). Figure 8J–S, obtained through combined high-resolution imaging and FFT analysis, reveals the presence of both oriented metallic Ni and NiO.

In essence, the results show that self-activation by LaNiO_3_ is possible under model DRM conditions, from which two important conclusions can be inferred: Firstly, La_2_NiO_4_, as the important transient phase under bar-level DRM operation conditions^23^, can be thermodynamically stabilized in the model experiments under vacuum, hydrogen reduction, and DRM conditions^26^. Secondly, as the reduced CO_2_ and CH_4_ partial pressures lead to a more oxidizing reaction mixture with little hydrogen, Ni particle formation is suppressed. However, the oxidation of Ni to NiO by reactive lattice oxygen from the parent LaNiO_3_ structure can be verified in both vacuum and DRM experiments.

#### Ni nanoparticle dynamics and structural evolution of LaNiO_3_ by heating in a 1:1 CO_2_:CH_4_ model DRM mixture after hydrogen pre-reduction

**Fig. 9:**
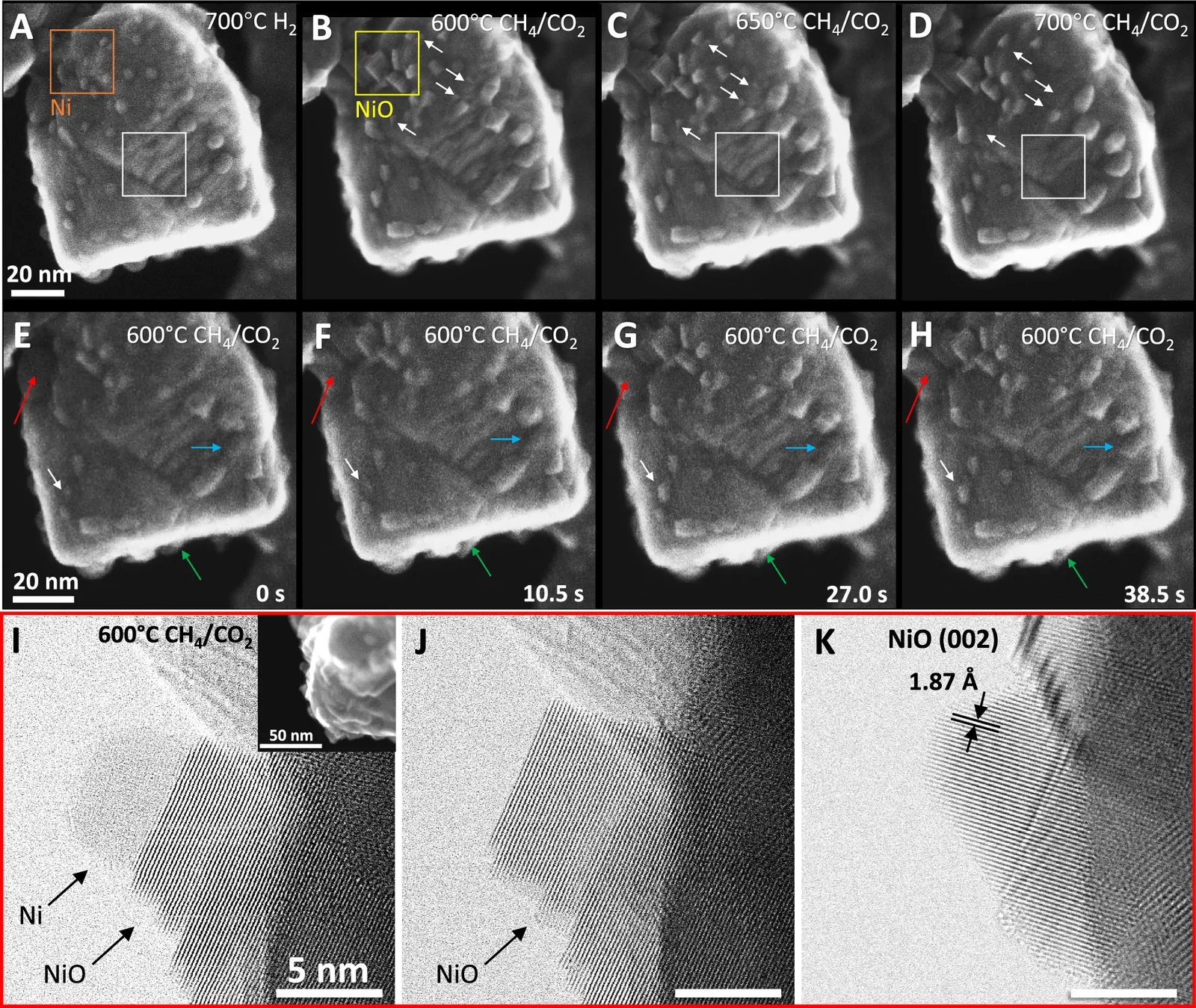
Structural evolution of LaNiO_3_ after hydrogen reduction and subsequent exposure to a CH_4_:CO_2_ = 1:1 model DRM mixture imaged by in situ STEM. **A** initial catalyst structure in H_2_ at 700 °C, the orange frame highlights the spherical Ni particles; catalyst structure in CH_4_ + CO_2_ at 600 °C (**B**, yellow frame highlights the surface-anchored NiO particles), 650 °C (**C**), and 700 °C (**D**). The region marked by the white frame indicates a further phase change of La_2_NiO_3_. **E**–**H** Snapshots of the dynamic oxidation process in CH_4_ + CO_2_ at 600 °C at 0 s, 10.5 s, 27 s, and 38.5 s. The fast oxidation process at three representative sites is marked by arrows in red, blue, and green, respectively. **I**–**K** Evolution of the oxidation of an isolated emerged Ni particle into NiO upon heating in CH_4_ + CO_2_ at 600 °C. Gas pressure: 10 Pa.

Since hydrogen pre-reduction is often the first step in activating DRM catalysts, we have further focused on the structural and morphological transformations of Ni particles on a partially decomposed LaNiO_3_ catalyst. We formed Ni particles by performing an initial reduction step in H_2_ at 700 °C. Then, we lowered the temperature to 600 °C and switched the reaction atmosphere to an oxidizing CH_4_:CO_2_ = 1:1 mixture. Figure 9A, B provides in situ insight into the structural changes before and after introducing the DRM gas mixture. The spherical Ni particles (orange frame in Panel A) rapidly oxidize into more faceted NiO particles (yellow frame in Panel B) when exposed to the gas mixture. The positions of the NiO particles correspond to those of the original Ni particles. Panels I–K highlight the evolution of the oxidation process of an isolated Ni particle and corroborate the in situ anisotropic growth of nickel oxide through layered metal oxidation. In this process, individual layers of NiO grow via disconnected migration along the metal-oxide interface^41^. Finally, a fully oxidized NiO particle with distinct NiO (002) lattice planes forms. Note that Fig. 9K shows the identical particle, but its shape and lattice orientation change. Supplementary Movie 3 records the dynamic oxidation process at 600 °C with representative snapshots over 38.5 s shown in Fig. 9E–H, exemplifying the dynamic sequential oxidation of Ni particles, indicated by red, green, white, and blue arrows. The oxidation process increases the particle size by incorporating oxygen atoms into the Ni lattice, leading to an expansion of the unit cell^42^.

As discussed above, the oxygen required for this oxidation stems from reactive surface-near lattice oxygen within the host matrix. The rapid oxidation of Ni particles demonstrates the efficient oxygen atom diffusion via oxygen vacancies to the Ni particles^43^. We tested the eventual oxidation of emerged Ni particles with directed CO_2_ treatments via in situ XPS. After hydrogen reduction at 700 °C in 20 Pa H_2_ (Fig. S17A—corresponding to the last La 3 d/ Ni 2p spectrum in Fig. 2) and CO_2_ adsorption between 25 and 700 °C, no oxidation of the metallic Ni particles was detected in pure CO_2_ (Fig. S17B). Hence, under the applied experimental conditions, Ni oxidation is primarily driven by reactive lattice oxygen. As previously discussed, oxygen vacancies in LaNiO_3_ are generated following Ni formation during the reduction process. These vacancies are considered active sites for CO_2_ decomposition and carbon removal^44^. The spectrum in Fig. S17B also directly reveals that CO_2_ is activated by partially refilling the oxygen vacancies induced by prior hydrogen reduction, thereby simultaneously releasing CO (Fig. S17C). This is a necessary prerequisite for the carbon removal chemistry. Therefore, the role of CO_2_ is twofold: (i) it is activated at oxygen vacancy sites, which refills oxygen and (ii) it helps to remove surface carbon deposition via the inverse Boudouard reaction $({{\rm{C}}}{{{\rm{O}}}}_{2}+{{\rm{C}}}\longleftrightarrow 2{{\rm{CO}}})$. Since carbon deposition is a major reason for catalyst deactivation in DRM and was directly observed on Ni surfaces using in situ TEM^45,46^, these findings demonstrate this catalyst’s excellent resistance to carbon deposition.

At 650 °C, the size, position, and morphology of the NiO particles remain unchanged (Fig. 9B, C). This stability indicates their greater sintering resistance than Ni nanoparticles at similar temperatures upon H_2_ exposure. This is due to the formation of surface-anchored NiO particles, which strengthen the interaction between metal and support and suppress their migration. Additionally, Supplementary Movie 3 confirms that no dynamic fragmentation-ripening or particle migration occurs at 650 °C. At 700 °C some small NiO particles, indicated by white arrows, experienced fragmentation compared to their state at 600 and 650 °C (Fig. 9B–D). Supplementary Movie 4 demonstrates that these small NiO particles exhibit a dynamic fragmentation behavior that is clearly distinct from metallic Ni particles present in H_2_ at similar temperatures. Furthermore, apart from the dissolution of small NiO particles, there is no evidence of particle growth elsewhere. Rather, larger NiO particles shrink in size. This suggests that only particle dissolution occurs without subsequent ripening. The morphology of stepped La_2_NiO_4_ (white frames in Panels A–C) remains essentially unchanged by the treatment. This mimics the vacuum-preheated sample, in which NiO@Ni particles form alongside La_2_NiO_4_. No substantial structural changes are observed at 700 °C when exposed to the gas mixture. The particles remain stable even at 850 °C (Fig. S18). This is expected because the structural and compositional consequences of both treatments are essentially similar, as discussed above.

### Correlation of structure and morphology evolution to DRM activity: transfer from model studies to technically relevant DRM operation conditions

**Fig. 10:**
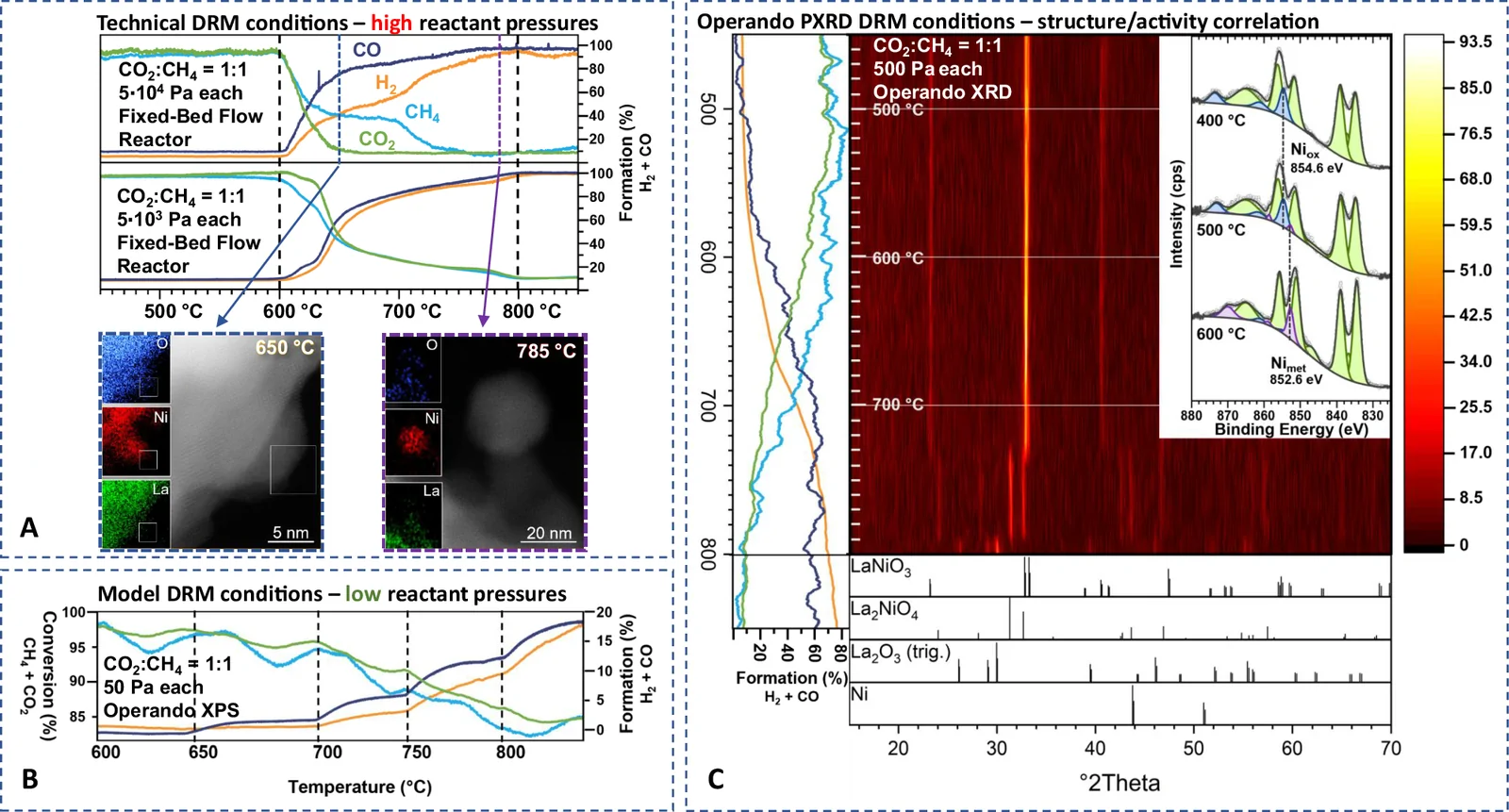
Validation of catalytic DRM profiles over LaNiO_3_ from model to technical operation conditions. **A** Comparison of DRM profiles measured in the fixed-bed flow reactor during LaNiO_3_ decomposition in a CO_2_:CH_4_ = 1:1 mixture (5 × 10^4 ^Pa and 5 × 10^3 ^Pa reactant pressure, respectively). Heating rate: 5 °C min^−1^, 200 mg catalyst. Ex situ STEM images/ EDX maps of an isolated oxidized and metallic Ni particle after heating to and quenching the DRM reaction at 650 °C (orange frame) and 785 °C (red frame), respectively. The white box in the STEM/EDX maps denotes the identical location in both images. **B** Catalytic DRM profile in a CO_2_:CH_4_ = 1:1 mixture (50 Pa reactant pressure each) measured during operando XPS. **C** Catalytic DRM profile in a CO_2_:CH_4_ = 1:1 mixture (1 × 10^3^ Pa reactant pressure each) measured during operando PXRD (left). Correlated evolution of the operando collected PXRD patterns between 450 °C and 800 °C (right) to verify the LaNiO_3_ decomposition into Ni and La_2_O_3_ via La_2_NiO_4_, corroborating ref. ^23^. The color scheme in the DRM profiles in (**A**) is the same for all DRM profiles in (**B**, **C**). The inset in (**C**) shows the operando-collected La *3d*/ Ni *2p* spectra in a CO_2_:CH_4_ = 1:1 DRM mixture (50 Pa reactant pressure each) at selected temperatures. The La region (La *3d*) is shown in green, oxidized Ni (Ni *2p*) in blue, and metallic Ni (Ni *2p*) in lilac.

To demonstrate the transferability of the catalytic properties of LaNiO_3_ from model studies to technical bar-level catalysis, and to validate the correlation of operando and in situ structural and spectroscopic information across several pressure regimes, we present catalytic DRM data obtained during LaNiO_3_ activation in our fixed-bed flow reactor (Fig. 10A; 5 × 10^4^ Pa and 5 × 10^3^ Pa CO_2_ and CH_4_ reactant pressures, respectively), during operando XPS (Fig. 10B; 50 Pa CO_2_ and CH_4_, respectively) and during operando PXRD experiments (Fig. 10C; 500 Pa CO_2_ and CH_4_, respectively, He added to 10^5^ Pa total pressure) as evidenced by CO_2_/CH_4_ conversion and H_2_/CO formation. While we cannot simulate the active DRM conditions in the (S)TEM experiments, the latter, in combination with operando PXRD and XPS, demonstrates that the initial stages of LaNiO_3_ activation—the formation of Ni particles, the concurrent appearance of La_2_NiO_4_, and the transient formation of oxidized Ni—are consistent across various DRM test conditions. Qualitatively, the DRM data are similar at all reactant partial pressures with respect to light-off temperature and H_2_/CO ratio (all of which feature a ratio of unity in the isothermal regions, indicating exclusive DRM activity). We cannot resolve the change in the catalytic activity associated with intermediate Ni oxidation during operando XPS due to the low reactant concentration and small amount of catalyst material. However, the change in activity is representative of the other experiments. Figure 10 and the STEM images of an isolated oxidized NiO and a metallic Ni particle after quenching the DRM reaction at 5 × 10^4^ Pa CO_2_/CH_4_ reactant pressure at 650 °C and 785 °C (orange and red frames in Fig. 10A, respectively; quantification details are provided in Figs. S19–S21) show that the transient formation of oxidized Ni by reactive oxygen occurs in the model in situ electron microscopy experiments and under bar-level DRM conditions likewise. Furthermore, complementary operando PXRD tests under comparable DRM conditions show that structurally, the LaNiO_3_ → La_2_NiO_4_ + Ni → La_2_O_3_ + Ni sequence is essentially independent of the reactant partial pressures and features similar formation of the final product structures (compare ref. ^23^ and Fig. 10/Fig. S22). Evidently, LaNiO_3_ can be directly activated under all presented catalytic DRM conditions. As shown in Fig. S23, once LaNiO_3_ is decomposed into Ni + La_2_O_3_, the catalytic light-off temperatures shift to lower temperatures, revealing the intrinsic DRM properties of the resulting Ni/La_2_O_3_ interface. This is because LaNiO_3_ is already activated before the second DRM cycle. A direct quantitative comparison is not straightforward because, for the DRM mixtures at 500 Pa and 50 Pa individual reactant pressure, the technical detection limit of the mass spectrometer is reached. This means that the given conversions represent the lower limit, and the true conversion is supposedly much higher.

One of the most important outcomes is that the formation of NiO at the initial stage of the DRM reaction partially hinders the overall catalytic activity compared to metallic Ni particles as active sites^47^. Oxidized Ni surfaces are poor methane activators, where the sticking probability of CH_4_ on these surfaces is typically at least one order of magnitude lower than on metallic Ni surfaces. We attribute this to the ongoing transfer of oxygen from the decomposing La–Ni–O phases (e.g., LaNiO_3_ or La_2_NiO_4_), which leads to the intermediate oxidation of the emerged metallic Ni particles. This phenomenon was previously observed in DRM profiles under ambient conditions as a characteristic deceleration in catalytic activity^23^. Under the conditions discussed in the framework of Fig. S23 (fixed-bed flow reactor at 500 Pa reactant pressure each), it manifests itself as the complete consumption of H_2_, arising from the reduced gas-phase reactant concentrations in the presence of an unchanged catalyst oxygen reservoir. A sharp acceleration of DRM activity then occurs at 650 °C, which is due to the reduction of NiO to metallic Ni. In both bar-level and 500 Pa experiments, this acceleration is caused by the cessation of oxygen transfer due to full decomposition of the La–Ni–O phases. This keeps the Ni reduced, resulting in a strong acceleration of DRM activity. Notably, the temperature at which NiO is reduced coincides precisely with the pronounced activity onset observed in bar-level experiments^23^. This allows us to draw two key conclusions: Firstly, DRM proceeds under identical thermodynamic control at both pressures, thereby resolving the pressure-gap discrepancy. Secondly, the Ni → NiO oxidation is governed by the catalyst’s internal oxygen reservoir, whose effective influence diminishes with increasing reactant pressure. The present study focuses on the regime of Ni oxidation: that is, we monitor the temperature region where the oxygen transfer is still occurring, and the remaining La–Ni–O phases are still present. We, therefore, prove that indeed the dynamic Ni oxidation is responsible for the observed peculiar DRM profiles.

We used LaNiO_3_ as the archetypal DRM material to follow its decomposition, the associated formation of intermediate phases, as well as Ni emergence from the parent perovskite lattice in different gas atmospheres, employing in situ electron microscopy. The combined use of simultaneously acquired dark-field, bright-field, and secondary electron imaging, together with operando probing of bulk and surface (electronic) structure, allowed us to pinpoint the locally entangled formation of Ni(O) particles and La_2_NiO_4_ as key intermediates en route to complete decomposition into metallic Ni particles and La_2_O_3_. The reduced reactant partial pressures, in comparison to real technological conditions, allowed us to more closely follow the initial stages of perovskite decomposition and activation by in situ electron microscopy imaging and electron spectroscopy. In contrast to vacuum annealing, where Ni particles remain embedded within the perovskite bulk, hydrogen reduction induces extensive Ni particle emergence at the perovskite surface. Particles formed in vacuo or in DRM atmospheres are prone to transient surface oxidation by lattice oxygen, whereas those formed under hydrogen reduction are highly dynamic and remain metallic. These temperature-dependent dynamical changes, especially at temperatures beyond 550 °C, are driven by multiple concurrent mechanisms, including ongoing Ni formation, phase transitions from LaNiO_3_ to La_2_NiO_4_, agglomeration processes, and particle migration. Under DRM conditions, the reaction environment becomes progressively more reducing due to H_2_ formation, promoting particle growth and decreasing particle density, ultimately being detrimental to catalytic performance. These high dynamics will eventually negatively impact the DRM performance. In the early stages of perovskite activation that were investigated, treatments in CO_2_:CH_4_ mixtures (with higher oxidation potential due to the presence of CO_2_) resemble those during vacuum annealing. Subsequent DRM treatments of Ni particles previously formed via hydrogen reduction revealed equally rapid oxidation to NiO. These NiO particles are more resistant to sintering, but also exhibit a size-dependent dissolution/agglomeration dynamics. As CO_2_ alone does not oxidize Ni under these conditions, this transient reaction process is entirely driven by oxygen migration from the perovskite bulk. CO_2_ activation is performed by partially replenishing the oxygen vacancies created during pre-reduction. However, as long as reactive oxygen is then resupplied from oxygen-containing perovskite phases, Ni oxidation will proceed. Only upon depletion of this oxygen reservoir is NiO reduced, coinciding with accelerated DRM activity. In situ electron microscopy, thus, provides structural evidence for this detrimental, ongoing oxidation process in the early stages of LaNiO_3_ decomposition. Moreover, we verified that not only intermediate Ni oxidation, but also the locally entangled appearance of faceted La_2_NiO_4_ and Ni particles as pivotal intermediate phases are salient features under both model and technical DRM operating conditions. This implies that, at progressed reaction levels, Ni particle formation is driven by recrystallization from LaNiO_3_ to La_2_NiO_4_, whose appearance and stability is strongly tied to the oxygen partial pressure. If the reduction strength is high enough, the formation of La_2_NiO_4_ is not observed. Summarizing, our studies allow us to introduce different oxygen partial pressure regimes, enabling us to access oxygen-partial-pressure-dependent phase stability regimes under more oxidizing conditions, including reduced hydrogen partial pressure, and monitor the formation and presence of the appearing phases.

## Methods

### Material synthesis

LaNiO_3_ was synthesized via a self-combustion method^48^. Stoichiometric amounts of La(NO_3_)_3_ and Ni(NO_3_)_2_ (both 99.99 % pure, Alfa Aesar) were dissolved in water and mixed in an aqueous solution. Glycine (NH_2_CH_2_CO_2_H) was added dropwise in an equimolar ratio ${{NO}}_{3}^{-}$ to ${{\mathrm{NO}}}_{3}^{-}$. The resulting green solution was subsequently heated for approximately 3 h at 95 °C until it turned into a viscous green gel. Further heating to 250 °C resulted in the formation of metal oxides and carbon residues under a controlled exothermal reaction. Calcination at 750 °C for 10 h in air produced the rhombohedral LaNiO_3_ perovskite structure (Fig. S13 and Table S2). The specific surface area, as determined by BET and measured by N_2_ adsorption at −196 °C using an Anton Paar Autosorb 6000 instrument, is 4.6 m^2^ g^−1^. Fig. S24 shows the respective pore size distribution alongside the cumulative pore volume, revealing a maximum half pore width of around 300 Å. The particle size of the as-prepared LaNiO_3_ is primarily distributed between 20 and 40 nm (Fig. S25). La_2_NiO_4_ was synthesized in the same way as LaNiO_3_ with adjusted stoichiometric La/Ni amounts and a final annealing step in air at 900 °C for 12 h.

### In situ scanning transmission electron microscopy (STEM)

In situ and ex situ STEM experiments were performed on a Hitachi High-Technologies HF5000 environmental S/TEM, equipped with a secondary electron detector. Pure hydrogen and a CO_2_:CH_4_ = 1:1 gas mixture were used for the reduction and DRM experiments, respectively. Gas pressures in the column were around 1.5 Pa. Vacuum heating experiments were performed at an instrument base pressure of 10^−6^ Pa, and all imaging was done at 200 kV. Beam currents during in situ measurements were around 100–200 pA, depending on the experiment. To prevent surface contamination during STEM investigations, LaNiO_3_ samples were preheated for 30 min at 200 °C before the experiments. Details of the in situ heating procedure and the number of repeated individual experiments are provided in Fig. S26 and Table S3. We used a temperature profile in which the temperature increased continuously from 200 to 800 °C at a heating rate of 10 °C min^−1^. After each 50 °C increment, the specimen was held for 10–30 min, and images were also collected at 50 °C step intervals. Comparison with pristine LaNiO_3_ at 25 °C (Figs. S27A and S28A–C) shows that the heat treatment at 200 °C under vacuum conditions and beam irradiation does not induce any obvious structural changes (Figs. S27B and S28D, E). To further assess the influence of electron beam irradiation, we exposed the sample to an electron beam at 300 and 400 °C for 10 min in vacuo. No morphology changes or damage to the sample due to the electron beam were detected (Fig. S27C, D). Pristine LaNiO_3_ samples were loaded onto a MEMS chip for the in situ experiments. The environmental scanning electron microscope operates under flowing gas conditions in the column. Hydrogen and CO_2_ + CH_4_ were used with a purity of 99.999% (O_2_ levels <1 ppm).

### Operando X-ray photoelectron spectroscopy (XPS)

The UHV system used for the near-ambient pressure X-ray photoelectron spectroscopy (NAP XPS) is a customized SPECS setup that maintains a base pressure below 10^−8^ Pa^49^. It comprises an analysis chamber, which can be backfilled with oxidative, reductive, and reactive gas atmospheres up to 3000 Pa, a PHIOBOS 150 NAP hemispherical energy analyzer with a 1D-DLD detector, a µFOCUS 600 NAP monochromatic small-spot X-ray source (Al Kα), and a flood gun (FG22/35). A 4-axis manipulator adapted for laser heating and gas-phase reaction was used for all experiments. High-resolution spectra of the Ni *2p*/La *3d*, Ni *3p*, and O *1s* regions were collected in 50 °C increments from 25 to 700 °C at a rate of 10 °C min^−1^. We relied on the fitted metallic and oxidic Ni *2p* peaks to perform quantitative analysis of the surface-bound Ni species. We fitted the spectra with a Shirley-type background and performed the quantitative analysis using the CasaXPS processing software based on the relative sensitivity factors. The analysis of the La *3d*/ Ni *2p* regions was accomplished by first evaluating all the La components with strict position, FWHM, and area constraints following the physically stipulated principles (area(La *3d*_*3/2*_) = area(La *3d*_*5/2*_) × 0.666667; FWHM(La *3d*_*3/2*_) = FWHM(La *3d*_*5/2*_))^50^. The remaining spectral data was fitted to minimize the RMS of the measured data to the simulated envelope by fitting oxidic Ni (symmetric peak shape GL(30)) and metallic Ni (asymmetric peak shape LA(1, 640)) with the corresponding Ni *2p*_*1/2*_ (0.5 times area of Ni *2p*_*3/2*_)^48^. The La spectral data was fitted by employing 7 peak features (2 La *3d*_*5/2*_, 2 La *3d*_*3/2*_, 1 plasmon feature to account for multiplet splitting in the 5/2 region, 1 plasmon loss feature at 848.5 eV and 1 Auger peak at 864.0 eV, following current literature)^51^. The O *1s* signals were fitted with respective components as reported for Ni–O systems^52^. Hydrogen with a purity of 99.999% (O_2_ levels <1 ppm) was used and controlled through MKS mass flow controllers. Operando measurements were performed using a PrismaPro mass spectrometer with a combined Pfeiffer HiCube pumping stage.

### In situ and operando powder X-ray diffraction (PXRD)

In situ high-temperature synchrotron PXRD experiments in pure helium were performed at the beamline 12.2.2 of the Advanced Light Source at the Lawrence Berkeley National Laboratory in California^53^. Diffraction patterns were collected in angle-dispersive transmission mode with a focused 25 keV monochromatic beam (*λ* = 0.4984 Å/15 μm spot size). The powder sample was heated in a 0.7-mm-inner-diameter quartz capillary under a continuous helium gas flow of 10 L min^−1^ injected through a 0.5-mm tungsten tube. The capillary was heated at a 10 °C min^−1^ to 800 °C in an infrared-heated silicon carbide (SiC) tube furnace^54^. Diffraction patterns were recorded by a Pilatus 3 S 1M detector (981 × 1043 pixels, pixel size 172 × 172 µm^2^, 30 ms readout time) every 60 s during the heating cycle. Rietveld refinement was performed using the FULLPROF program, and helium was used with a purity of 99.999%^55^.

Operando PXRD analysis of LaNiO_3_ was carried out on a Rigaku SmartLab diffractometer (theta/theta geometry, Cu-Kα_1,2_), using a HyPix-3000 detector in 1D scan mode with a Reactor X high-temperature attachment. The analysis was performed up to 800 °C in a flow of 500 Pa CO_2_ + 500 Pa CH_4_, with helium added to 10^5^ Pa and controlled through MKS mass flow controllers. The powder was placed in a quartz sample holder, and continuous patterns were recorded in parallel beam alignment within the 15° to 70° 2θ range with a step width of 0.01°. Operando measurements were performed using a PrismaPro mass spectrometer with a combined Pfeiffer HiCube pumping stage, which was connected to the Reactor-X chamber via a capillary.

### Catalytic dry reforming of methane (DRM)

For the catalytic experiments in the fixed-bed flow reactor, 200 mg of the sample powder was placed in a bed of quartz wool inside a quartz reactor tube with an inner diameter of 7 mm. H_2_ pre-treatments were conducted in a continuous flow of 20 ml min^−1^ pure H_2_ at 800 °C for 2 h. For the catalytic measurements, CH_4_ and CO_2_ were mixed at a 1:1 ratio at reactant pressures of 5 × 10^4^ Pa, 5 × 10^3^ Pa, or 500 Pa, with a flow rate of 20 ml min^−1^ each and a heating rate of 5 °C min^−1^ from 50 to 800 °C. This was followed by an isothermal period at 800 °C for 30 min. An external S-type thermocouple was placed in close proximity to the reactor tube to ensure accurate temperature control. The output gas was detected directly by an online quadrupole mass spectrometer (Balzers QME 125). CO_2_, CH_4_, CO, He, and H_2_ were measured at their respective *m*/*z* ratios of *m*/*z* 44, 16, 28, 4, and 2. Relevant fragmentation patterns were considered. To minimize condensation from potential H_2_O formation, silica gel was placed at the reactor output. To display the catalytic data, we show the conversion/formation of the relevant reactant signals as a qualitative measure of the catalytic activity. Due to the ongoing structural transformations during the DRM reaction, active-site-normalized reaction rates and, consequently, turnover frequency values cannot be reliably calculated.

## Supplementary information

Supplementary Information

Description of Additional Supplementary Files

Supplementary Movie 1

Supplementary Movie 2

Supplementary Movie 3

Supplementary Movie 4

Transparent Peer Review file

## Source data

Source Data 1

Source Data 2

## Data Availability

The STEM, XPS, and XRD data that support the findings of this study are available in the LFU repository with the identifier https://doi.org/10.48323/g915w-41a98. Source data are provided with this paper.
