# Supplementary material for: In situ electron microscopy of LaNiO3 transformation during reduction and methane dry reforming
Source: Nat Commun. 2026 Aug 7;17:9487. doi: 10.1038/s41467-026-76419-1 (PMC13542283; doi:10.1038/s41467-026-76419-1)
Supplement: Supplementary file 1 [file 41467_2026_76419_MOESM1_ESM.pdf]

Supporting Information

In situ Electron Microscopy of $LaNiO_3$ Transformation during Reduction and Methane Dry Reforming

Pengfei Cao[a,b,♯], Christoph Malleier,[c, ♯] Dylan Jennings[d], Leander Haug,[c] Maged F. Bekheet[e], Joachim Mayer[a,f], Rafal E. Dunin-Borkowski[a], Simon Penner[c*], Marc Heggen[a*]

[a] *Ernst Ruska-Centre for Microscopy and Spectroscopy with Electrons (ER-C), Forschungszentrum Jülich GmbH, 52425 Jülich, Germany*

[b] *Institute for a sustainable Hydrogen Economy (IHE-1), Forschungszentrum Jülich GmbH, Jülich 52428, Germany*

[c] *Department of Physical Chemistry, University of Innsbruck, Innrain 52c, A-6020 Innsbruck, Austria*

[d] *Institute of Energy Materials and Devices, Materials Synthesis and Processing (IMD-2), Forschungszentrum Jülich GmbH, 52425 Jülich, Germany, Present address: Center for Interface Dominated High Performance Materials, Advanced Transmission Electron Microscopy (ATEM), Ruhr-University-Bochum, 44801 Bochum, Germany*

[e] *Technische Universität Berlin, Faculty III Process Sciences, Institute of Materials Science and Technology, Chair of Advanced Ceramic Materials, Straße des 17. Juni 135, 10623 Berlin, Germany*

[f] *Central Facility for Electron Microscopy (GFE), RWTH Aachen University, 52064 Aachen, Germany*

Keywords: Perovskites, Ni particle formation, in situ TEM heating, in situ X-ray diffraction, in situ X-ray photoelectron spectroscopy

♯These two authors contributed equally

*Corresponding authors: simon.penner@uibk.ac.at; m.heggen@fz-juelich.de

Section A HR-TEM images of surface roughening of $LaNiO_3$ during reduction in H_2

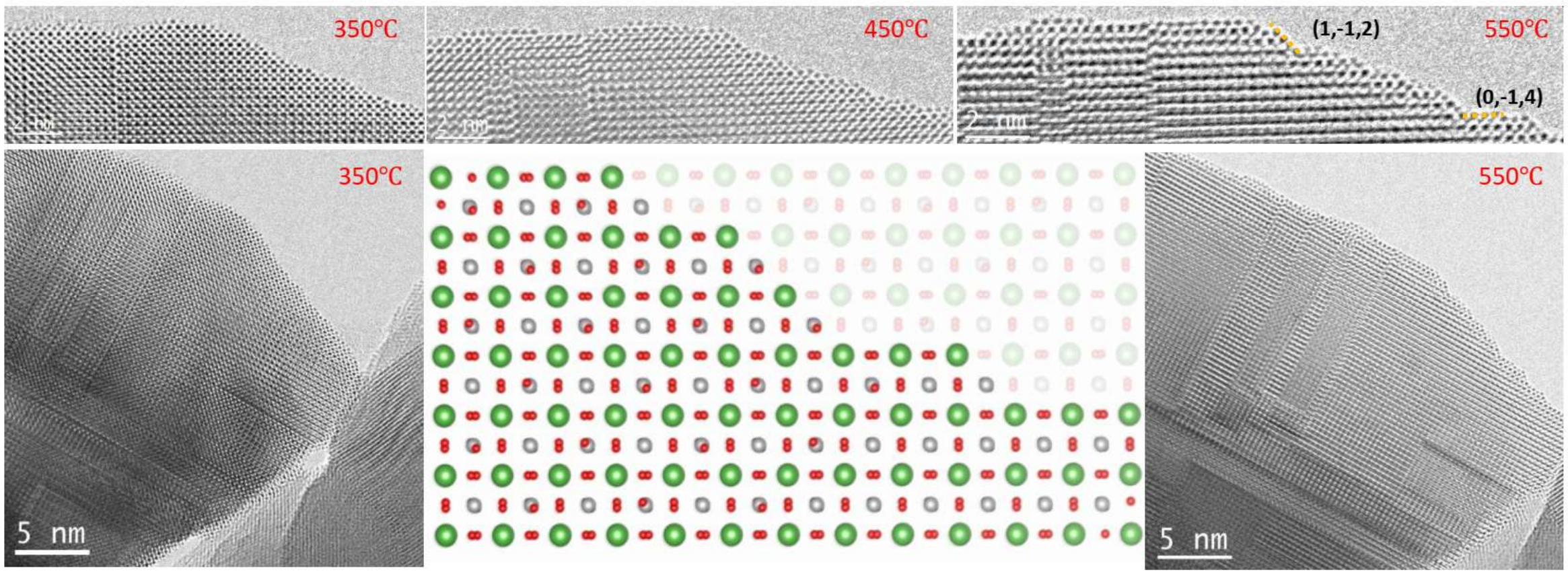

Fig. S1 | Sequence of HR-TEM images of the $LaNiO_3$ surface progressively roughening with increasing hydrogen reduction temperature between 350 °C and 550 °C. A model and lattice planes formed are also highlighted.

Section B Benchmark XP spectra of $LaNiO_3$, La_2NiO_4 and La_2O_3

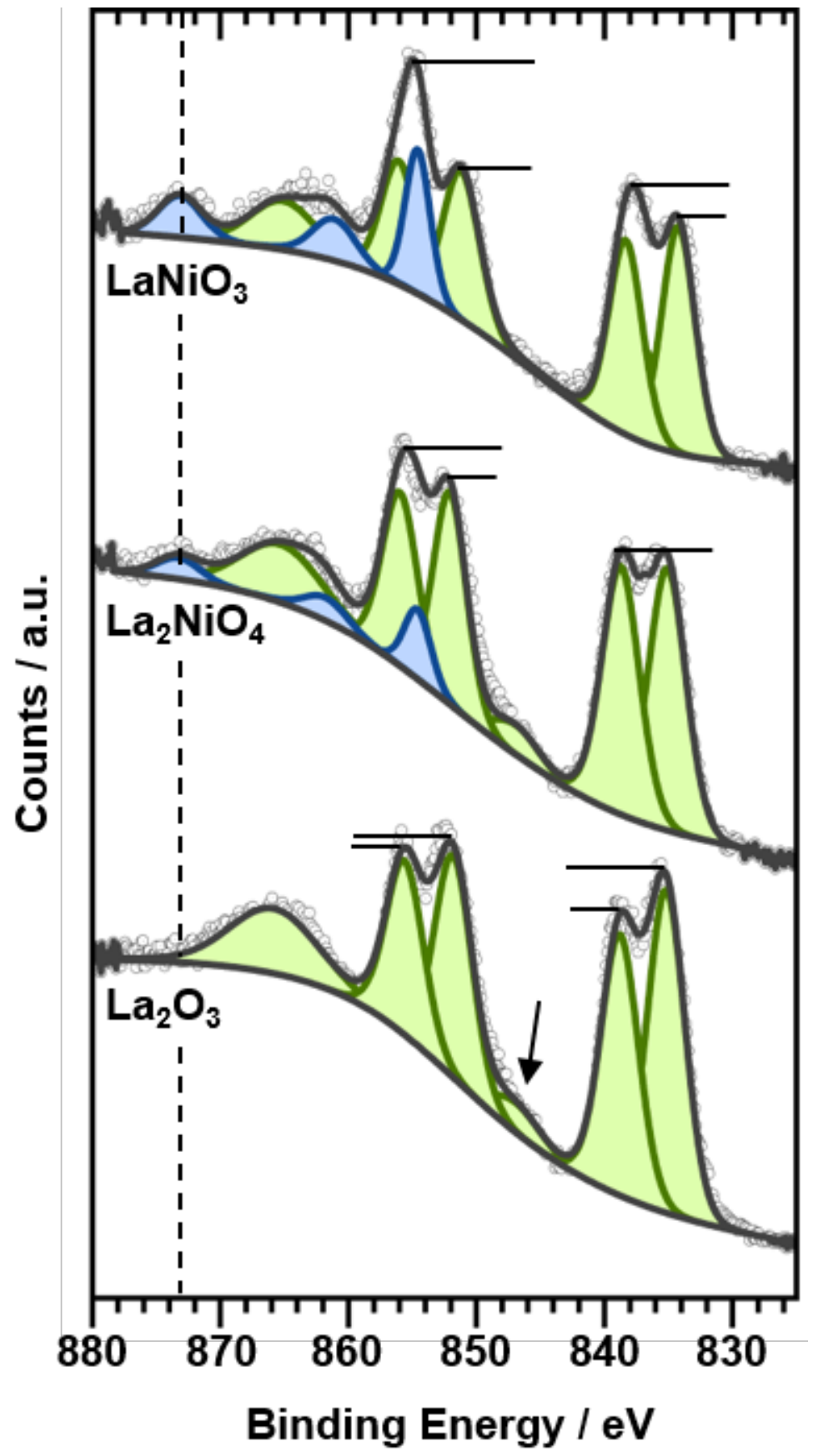

Fig. S2 | High-resolution benchmark La 3d/ Ni 2p and O 1s spectra of $LaNiO_3$, La_2NiO_4 and La_2O_3

Section C Operando in situ X-ray photoelectron spectroscopy of $LaNiO_3$ during hydrogen reduction

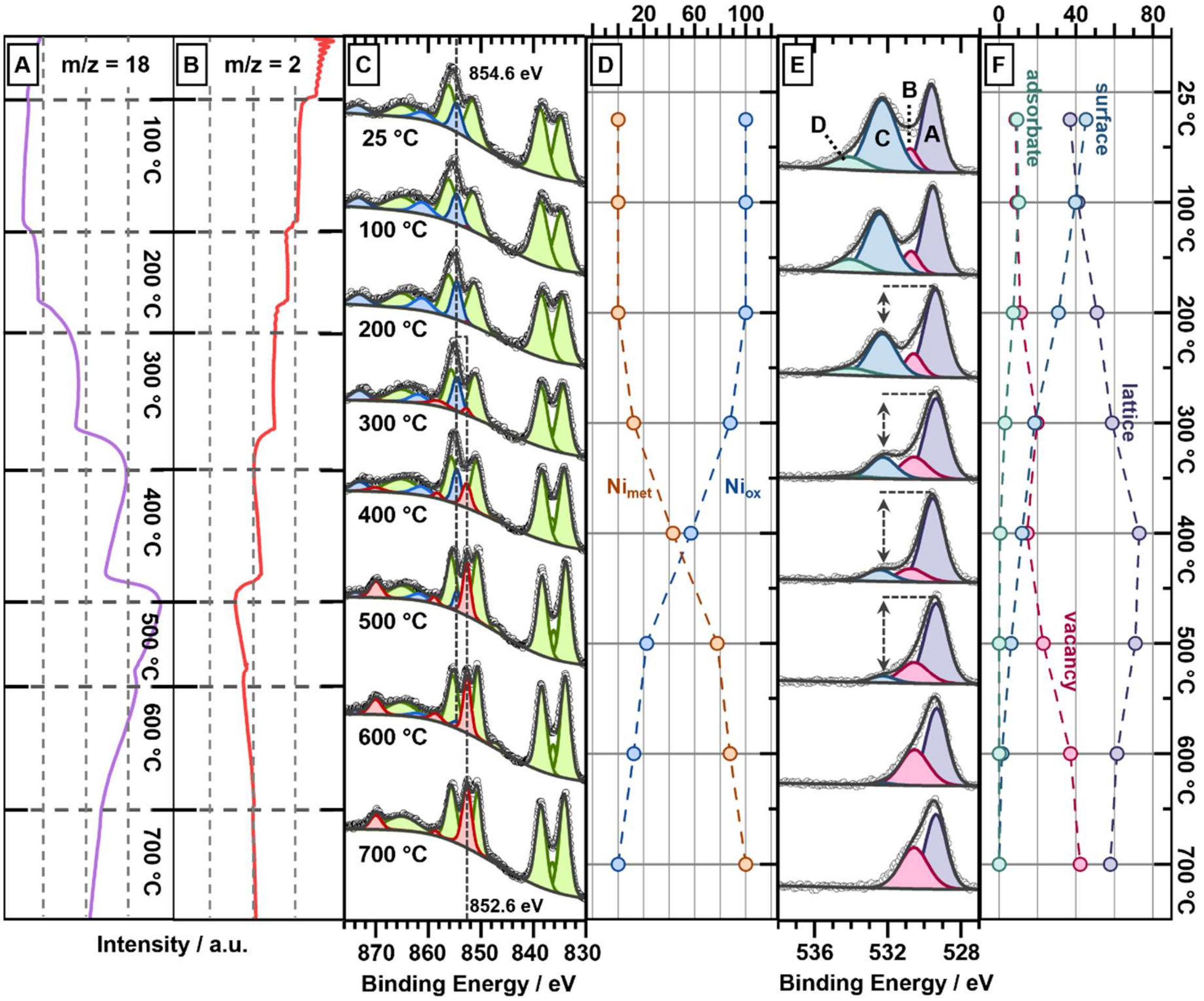

Fig. S3 | Temperature-dependent evolution of the surface electronic structure of $LaNiO_3$ during hydrogen reduction at selected temperatures between 300 °C and 700 °C monitored by in situ X-ray photoelectron spectroscopy. Panel A and B: Simultaneously collected mass spectra of m/z = 2 (hydrogen) and m/z = 18 (water). Panel C: High-resolution Ni *2p* and La *3d* regions. Panel D: Evolution of the metallic and oxide Ni components. Panel E: O *1s* region. A – lattice (purple); B – vacancy (lilac); C – surface (blue); D – oxygenate species (green). Panel F:

Evolution of the oxygen components. Hydrogen pressure: 20 Pa. Details of the fitting procedure discussed in Section 2.3.

Section D Additional characterization of Ni exsolution dynamics at 400 °C under hydrogen

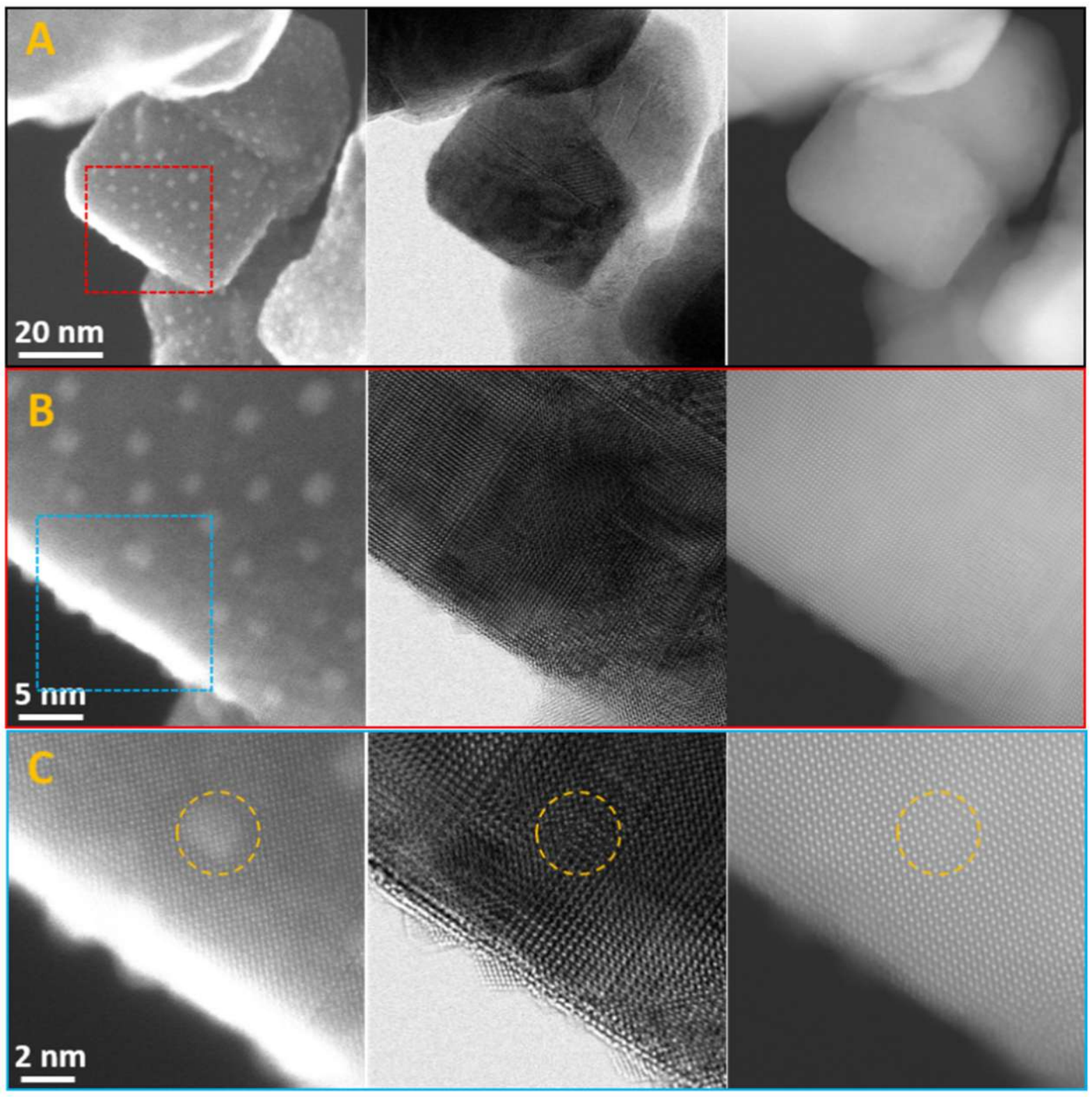

Fig. S4 | Simultaneously recorded SE, BF, and DF images at 400°C in H_2. Panel A: low magnification; Panel B: enlarged images of selected region indicated by the red box, Panel C: atomic-resolution image of the selected region indicated by the blue box.

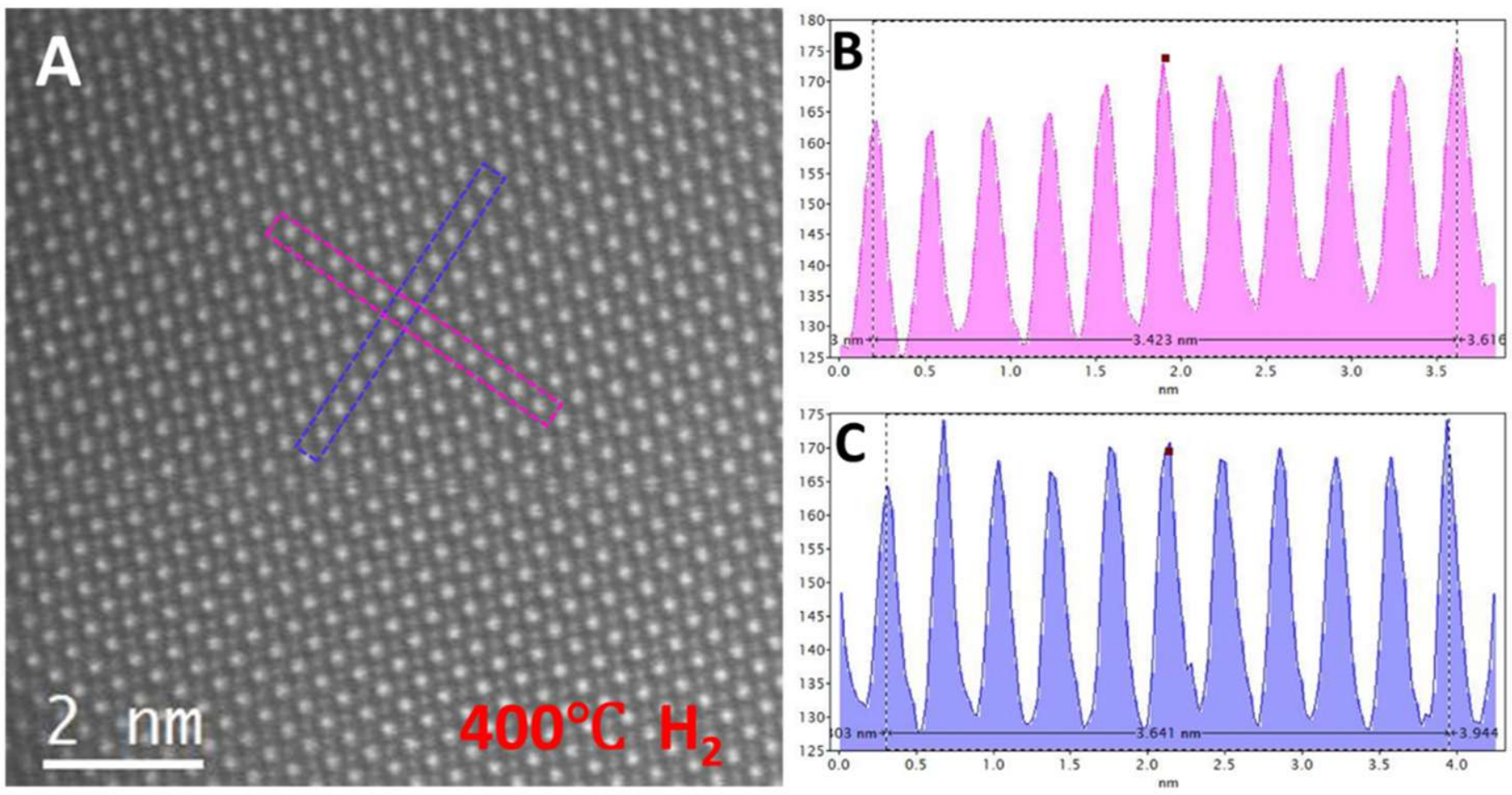

Fig. S5 | Distortion of the perovskite structure after hydrogen reduction at 400 °C. Panel A: STEM-HAADF image indicating that the host matrix retains its perovskite structure at 400 in H_2. However, the lattice spacing in the pink box appears compressed (B), compared to that in blue box (C), indicating a structure distortion. In perfect rhombohedral $LaNiO_3$, the lattice spacing along the perpendicular atom rows is symmetric.

Table S1 Quantitative analysis of Ni particles at 500 °C and 650 °C.

500 °C			650 °C		
No.	Diameter (nm)	Volume (nm^3)	No.	Diameter (nm)	Volume (nm^3)
1	4.8	56.8	1	6.3	132.8
2	2.9	12.9	2	5.0	65.8
3	2.8	11.4	3	0	0
4	2.5	8.5	4	0	0
5	2.7	10.1	5	0	0
6	2.9	12.8	6	2.9	12.2
7	2.5	8.1	7	0	0
8	2.0	4.0	8	0	0
9	2.7	10.2	9	0	0
10	2.2	5.2	10	0	0
11	1.0	0.6	11	0	0
12	2.4	6.9	12	0	0
13	2.1	4.6	13	0	0
Sum		152.1			210.8

Quantitative analysis of Ni particle of **Figure 4**: We have performed a quantitative analysis to compare the volume of Ni particles at 500 °C and 650 °C. The exsolved Ni volume was estimated by assuming spherical particle shapes. We measured the diameters of particles 1 - 13 and calculated their volumes using the equation for the sphere volume $V = \frac{4}{3}r^3\pi$. The results show that the combined volume of particles 1, 2, and 6 at 650°C is 210.8 nm^3, which is greater than their combined volume at 500°C (152.1 nm^3). This indicates the dissolution of particles 3, 4, 5, and 7-13 and a mass transfer into particles 1 and 2. The observed increase in volume is likely attributed to additional nickel particle precipitation/exsolution.

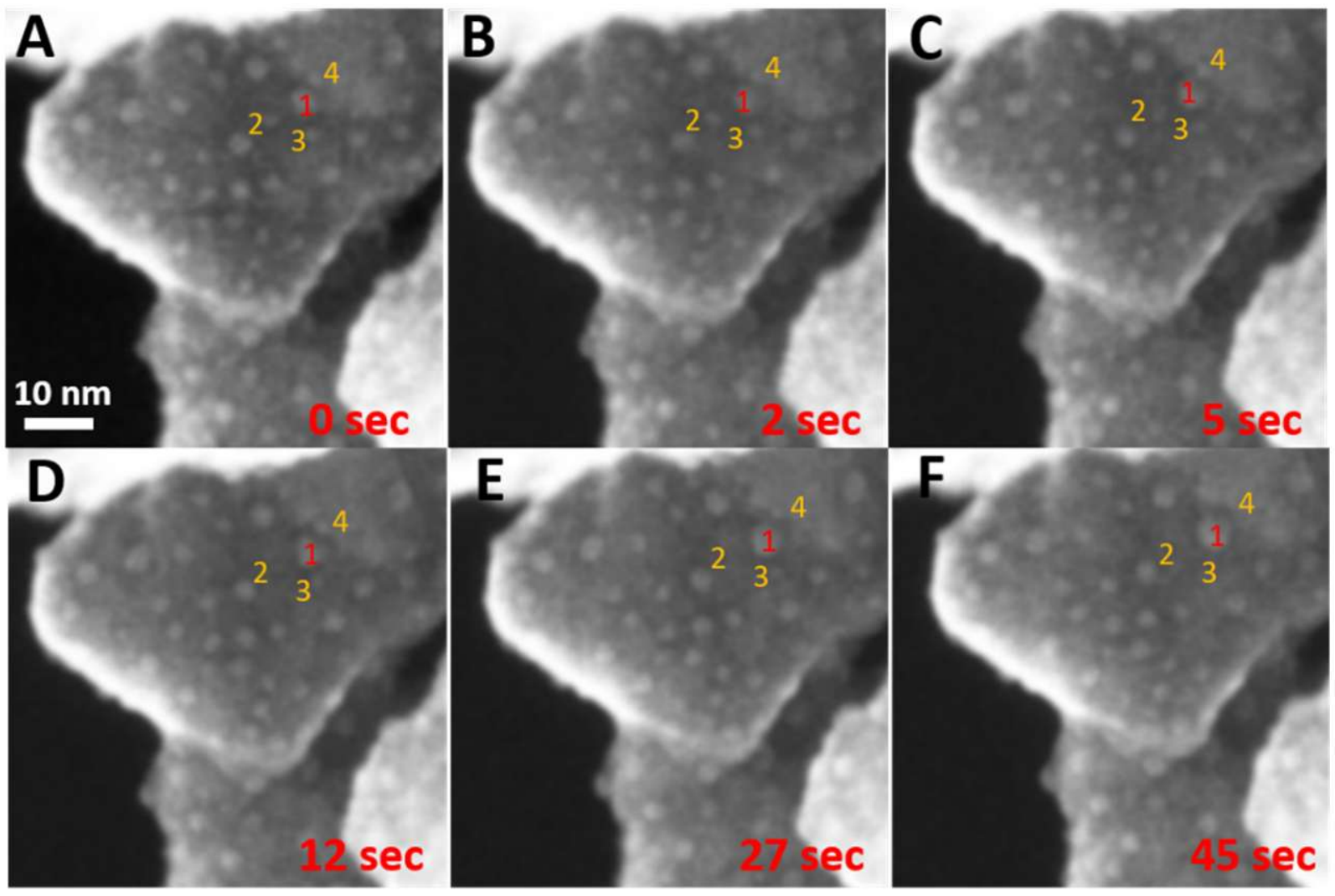

Fig. S6 | In situ STEM-SE imaging of Ni particle sintering at 600°C in H_2 over a time period of 45 seconds. Four representative particles are labelled, with particle 1 being the larger one in the center and particles 2, 3, and 4 being smaller and surrounding of particle 1.

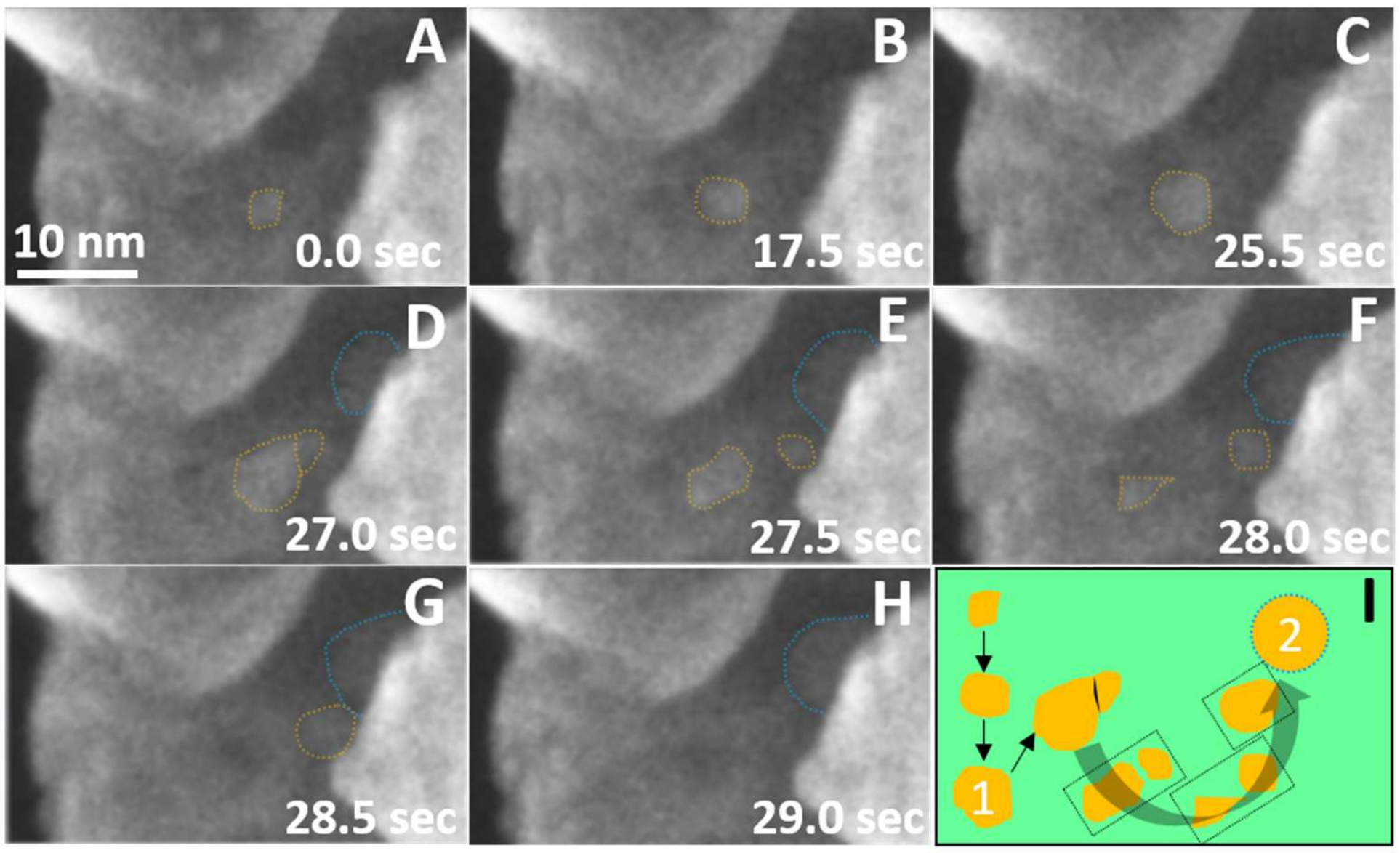

Fig. S7 | In situ STEM-SE imaging of particle migration over a period of 29.0 seconds at a temperature of 700 °C in H_2. Panel A: 0.0 sec, Panel B: 17.5 sec, Panel C: 25.5 sec, Panel D: 27.0 sec, Panel E: 27.5 sec, Panel F: 28.0 sec, Panel G: 28.5 sec and Panel H: 29.0 sec; Panel I: Schematic representation of the particle migration pathway for Ni coalescence.

In addition to fragmentation and diffusion, the mobility and splitting behavior of a Ni particle was tracked. This Ni particle (yellow dashed circle), was tracked in **Fig. S7** and **Movie S2**, which was initial 1.3 nm in size and grew up to 5 nm after 25.5 sec at 700°C (**Fig. S7A-C**). Later, this particle shows and elongated shape and gets split into two small segments. Later, the upper right small particle is probably absorbed by another big particle (blue dashed line). As depicted in **Fig. S7G**, a dark contrast feature is present at the region where the Ni particle is detached from the host material.

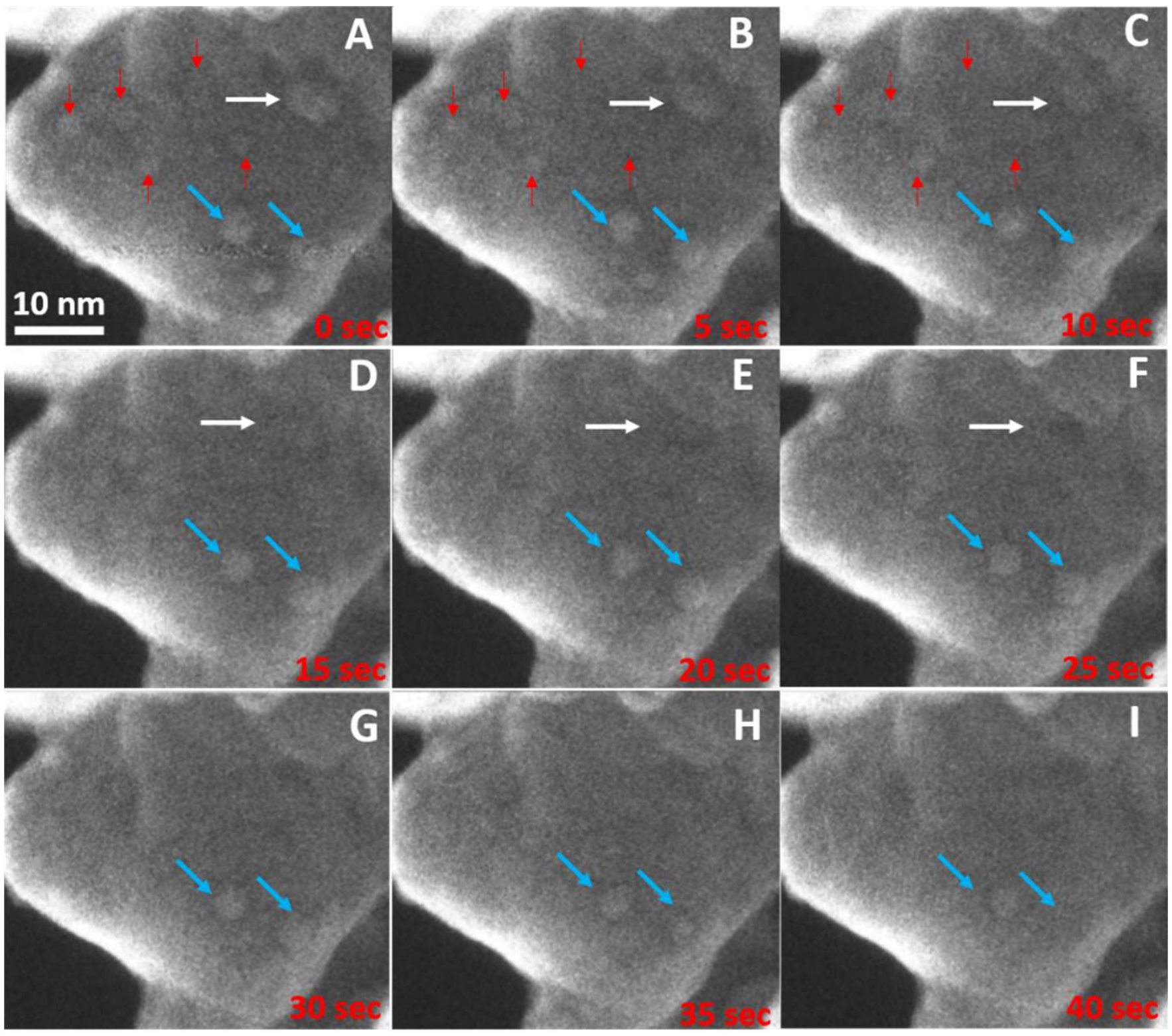

Fig. S8 | In situ STEM-SE imaging of Ni particle sintering at 700 °C in H_2 for 40 seconds. Representative particles are identified by arrows in different colors, indicating their fragmentation rates: red denotes the fastest, followed by white, and then blue.

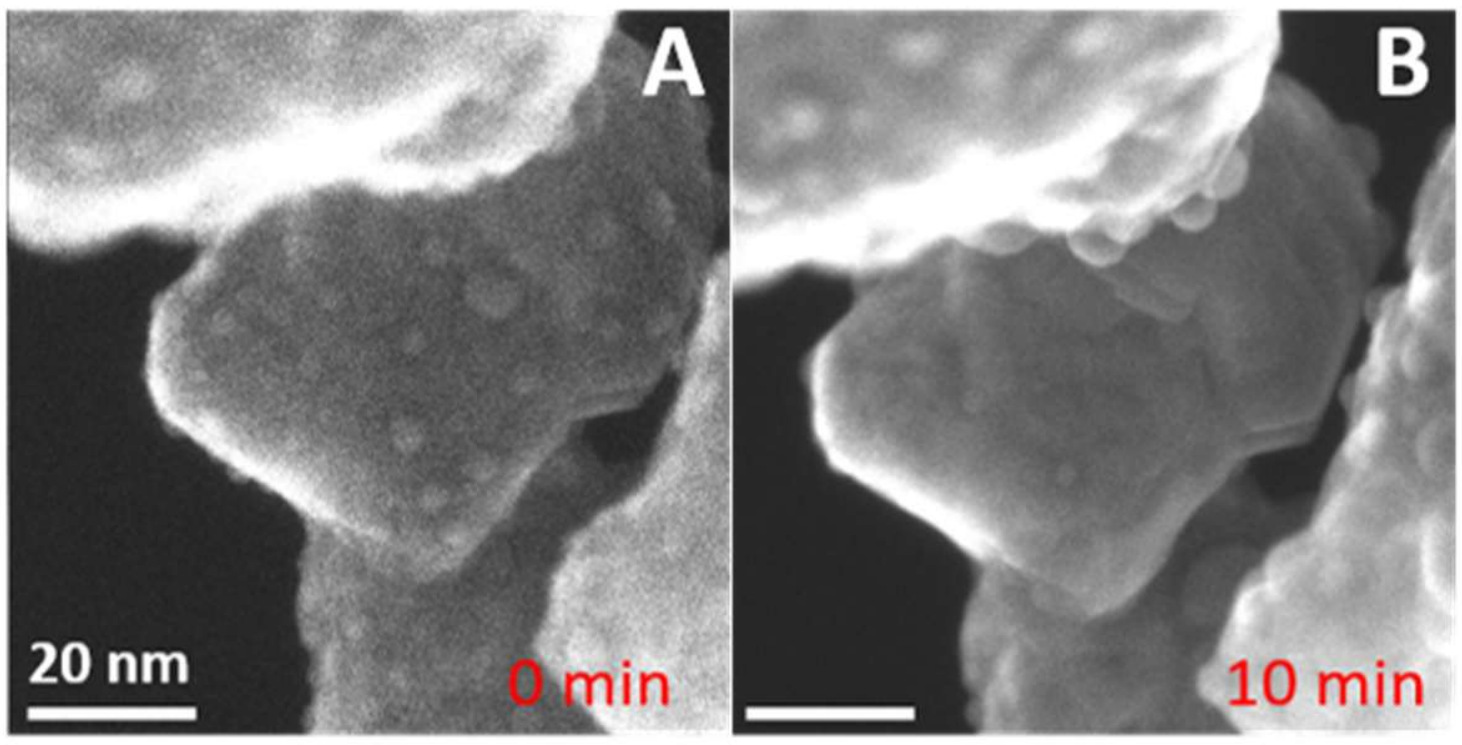

Fig. S9 | In situ SE imaging of the pronounced Ni fragmentation process before (Panel A) and after the isothermal step at 700 °C in H_2 for 10 min (Panel B).

Section E Additional characterization of Ni formation during vacuum heating

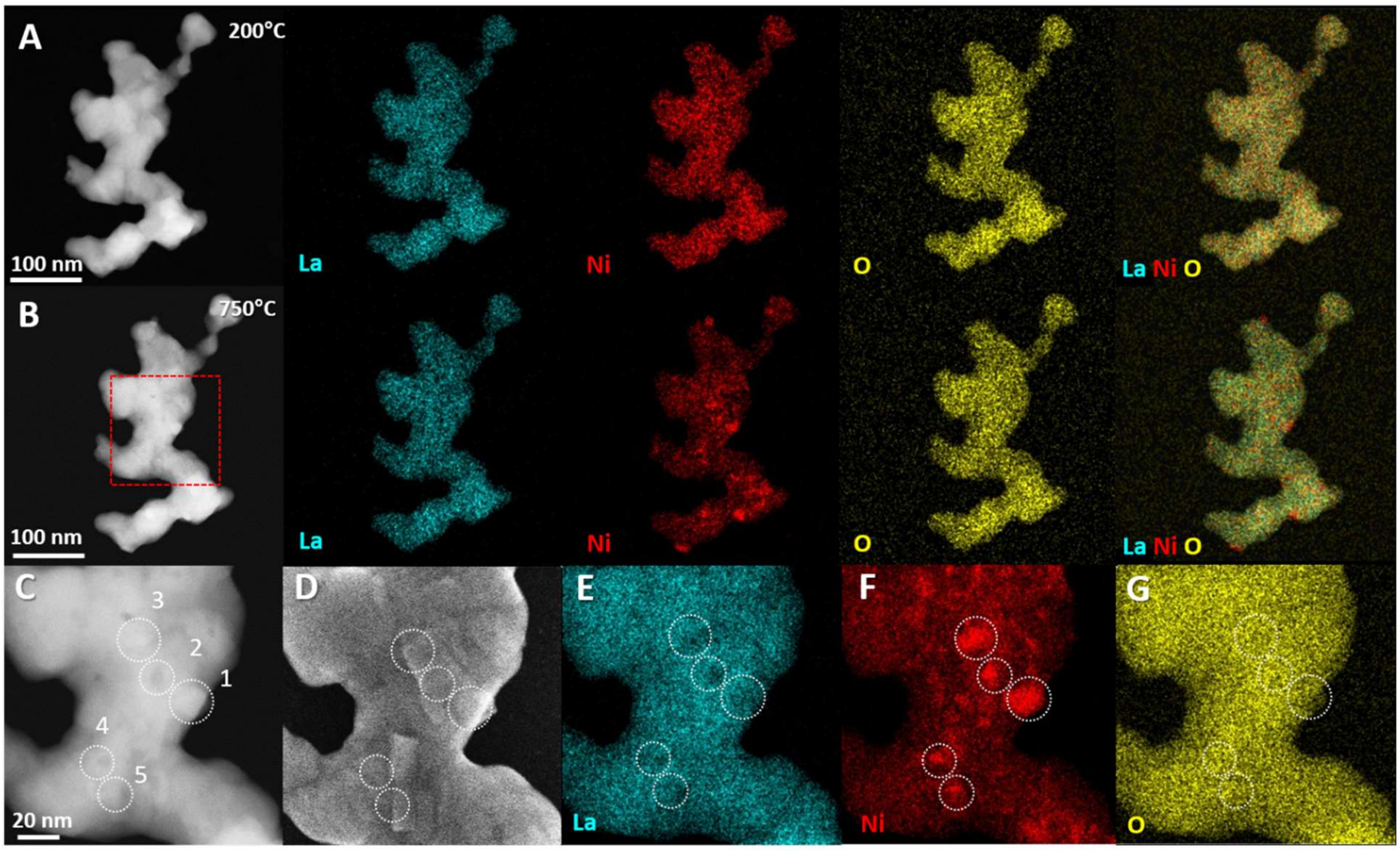

Fig. S10 | HAADF-STEM images and EDX maps of $LaNiO_3$ before and after vacuum heating. Panel A: Sample at 200 °C. Independent and mixed elemental distribution maps of La-L, Ni-K, and O-K; Panel B: Sample after a full heating-cooling cycle with a maximum temperature of 750 °C; Panel C: enlarged DF image of the region in the red dashed box after heating; Panel D: corresponding SE image; Panels E, F and G: elemental distribution of Ni, La and O, where Ni-rich intensity areas are marked by white dashed circles.

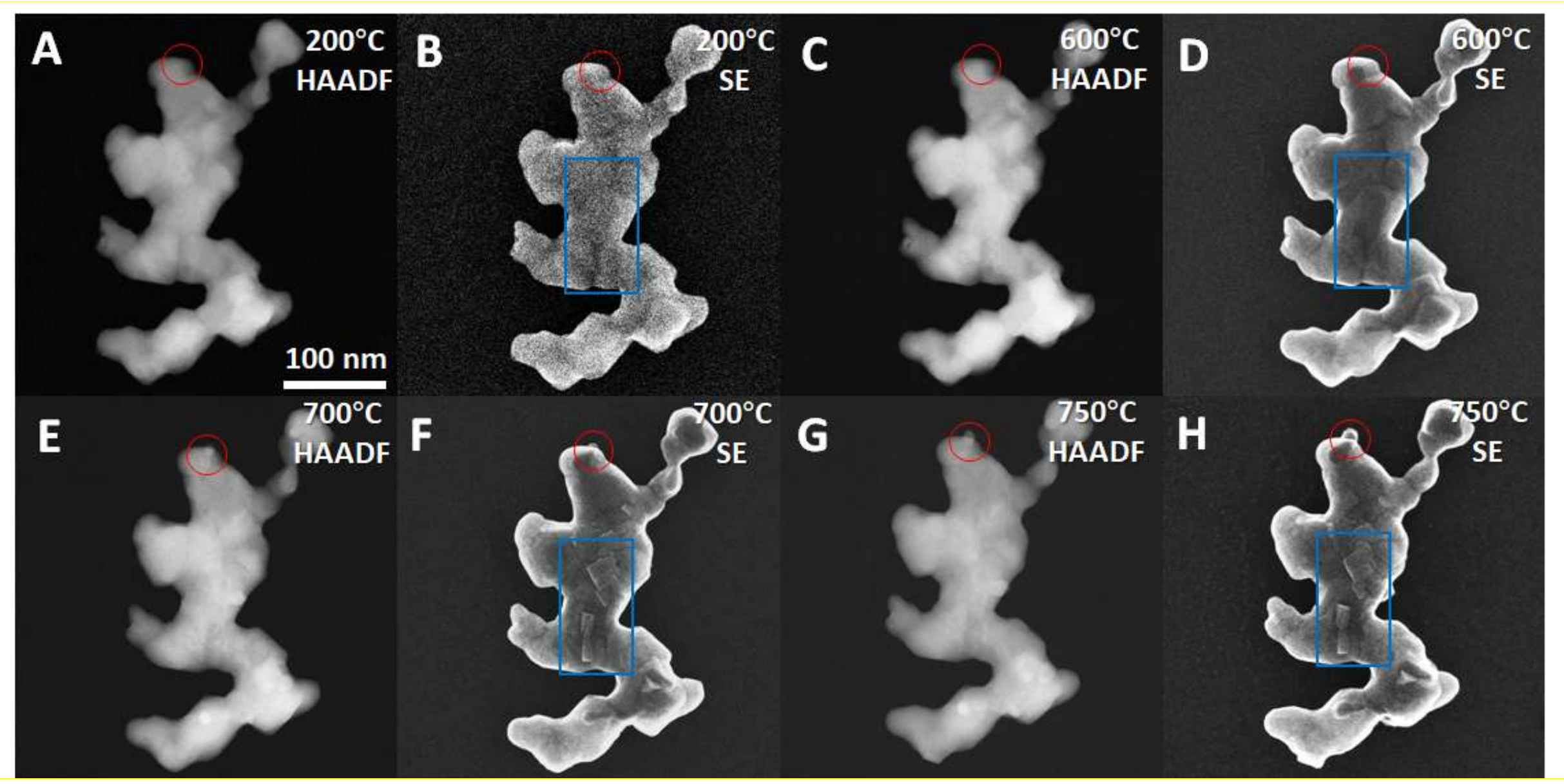

Fig. S11 | In situ STEM-HAADF and SE imaging series during vacuum heating of $LaNiO_3$ between 200 °C and 750 °C. Panels A and B: 200 °C, Panels C and D: 600 °C, Panels E and F: 700 °C, Panels G and H: 750 °C.

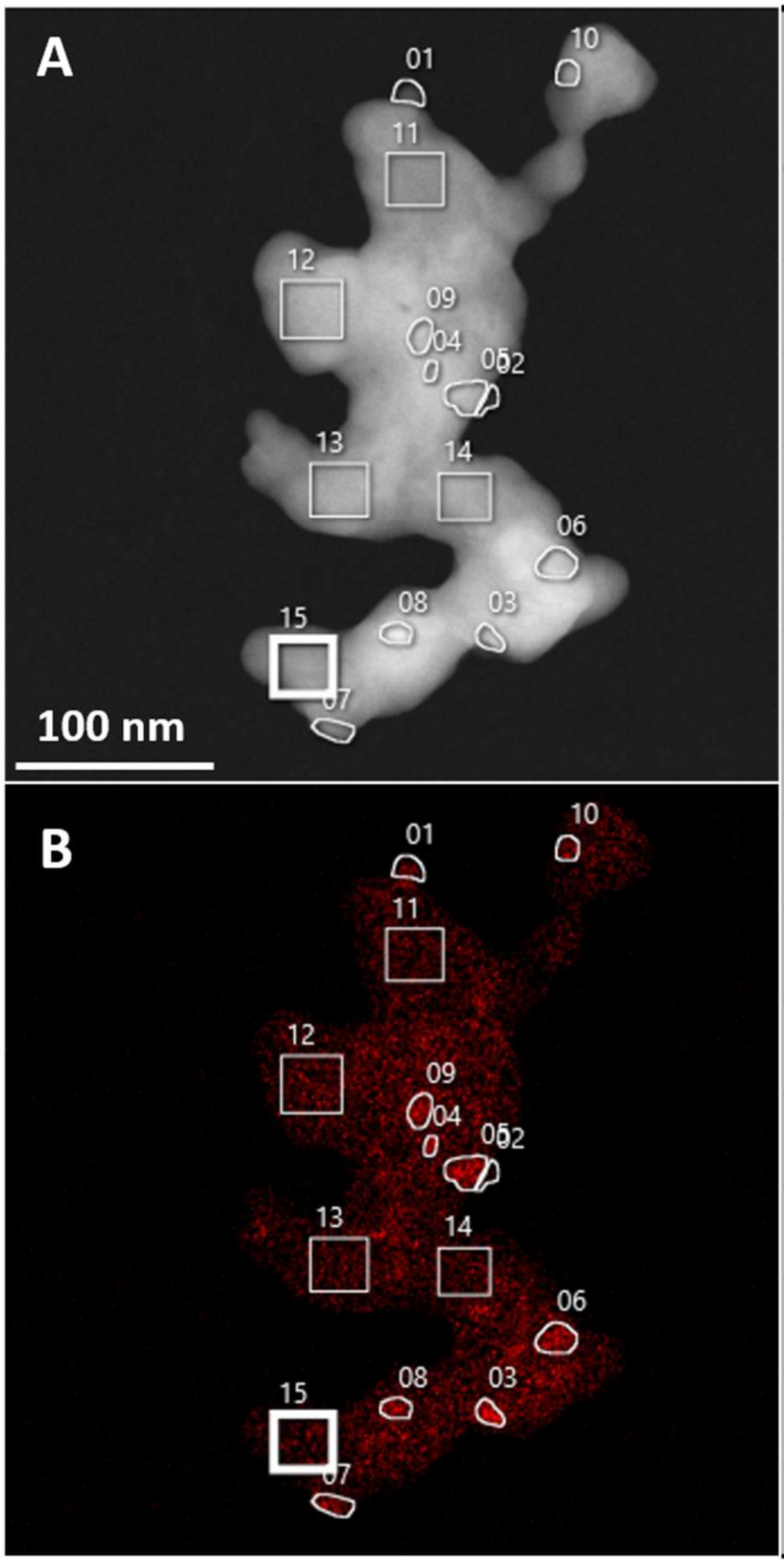

Sites	Ni atom%	La atom%	Ni/La
01	26.6	1.9	7.0
02	19.4	3.8	5.1
03	25.2	10.4	2.4
04	20.7	6.7	3.9
05	22.2	13.7	1.62
06	16.3	15.3	1.06
07	20.6	8.3	2.48
08	14.1	16.7	0.84
09	16.0	13.0	1.23
10	14.1	12.6	1.12
11	8.2	15.8	0.52
12	8.0	16.1	0.50
13	7.7	17.9	0.43
14	8.3	17.2	0.48
15	7.7	16.2	0.48

Fig. S12 | Quantitative EDX analysis of the $LaNiO_3$ grains shown in Figure 6 to confirm the formation of Ni particles on $LaNiO_3$ after vacuum heating to 800 °C. Panel A: HAADF-STEM image; Panel B: EDX elemental distribution map of Ni. 15 analyzed areas are indicated by white circles.

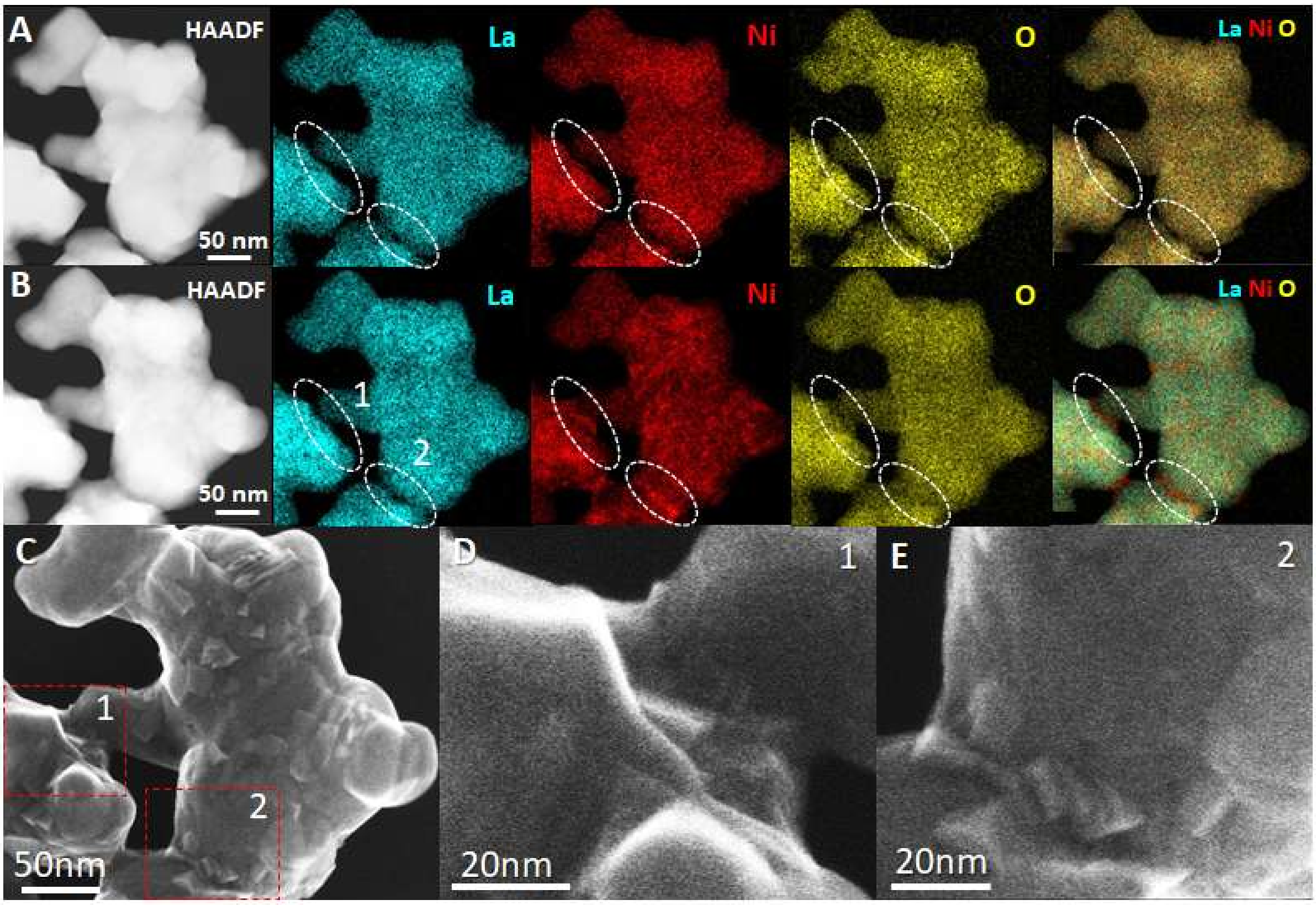

Fig. S13 | Corroboration of the simultaneous presence of La_2NiO_4 and NiO in close vicinity by combined in situ STEM/SE imaging and EDX elemental mapping of $LaNiO_3$ after a full vacuum heating-cooling cycle to a maximum temperature of 750 °C. Panel A and B: HAADF image, including individual EDX maps and superposition of La-L, Ni-K, and O-K intensity of $LaNiO_3$ at 200 °C (A) and after annealing to 750 °C (B). Intraparticle regions (boundary 1 and 2) are marked by white dash line ovals, respectively. Panel C: SE image taken at 750°C. Panel D and E: enlarged SE images of the boxed regions corresponding to boundary 1 and 2, respectively.

Further evidence of the locally entangled appearance of both phases is provided during heat-induced structural reconstruction. Gaps between adjacent host particles at 200 °C are filled by Ni and O at 750 °C (Fig. S13), where faceted La_2NiO_4 appeared, accompanying the emergence of Ni as demonstrated by SE images.

Section F In situ X-ray diffraction experiments under helium

Table S2 Summary of structural parameters for all mentioned phases in this work.

Chemical Formula	Space Group	Crystal System	a, b, c (Å)	α, β, γ (°)
$LaNiO_3$	*R 3 c*	rhombohedral	a=b= 5.4535(1) Å, c= 13.1010(3) Å	α=β, γ =120°
La_2NiO_4	*F m m m*	orthorhombic	a=b=5.4652(1) Å, c= 12.6780(2) Å	α=β= γ
Ni	*F m* 3 *m*	cubic	a=b=c=3.5779(0) Å	α=β=γ=90°
NiO	*F m* 3 *m*	cubic	a=b=c=4.1780(10) Å	α=β=γ=90°

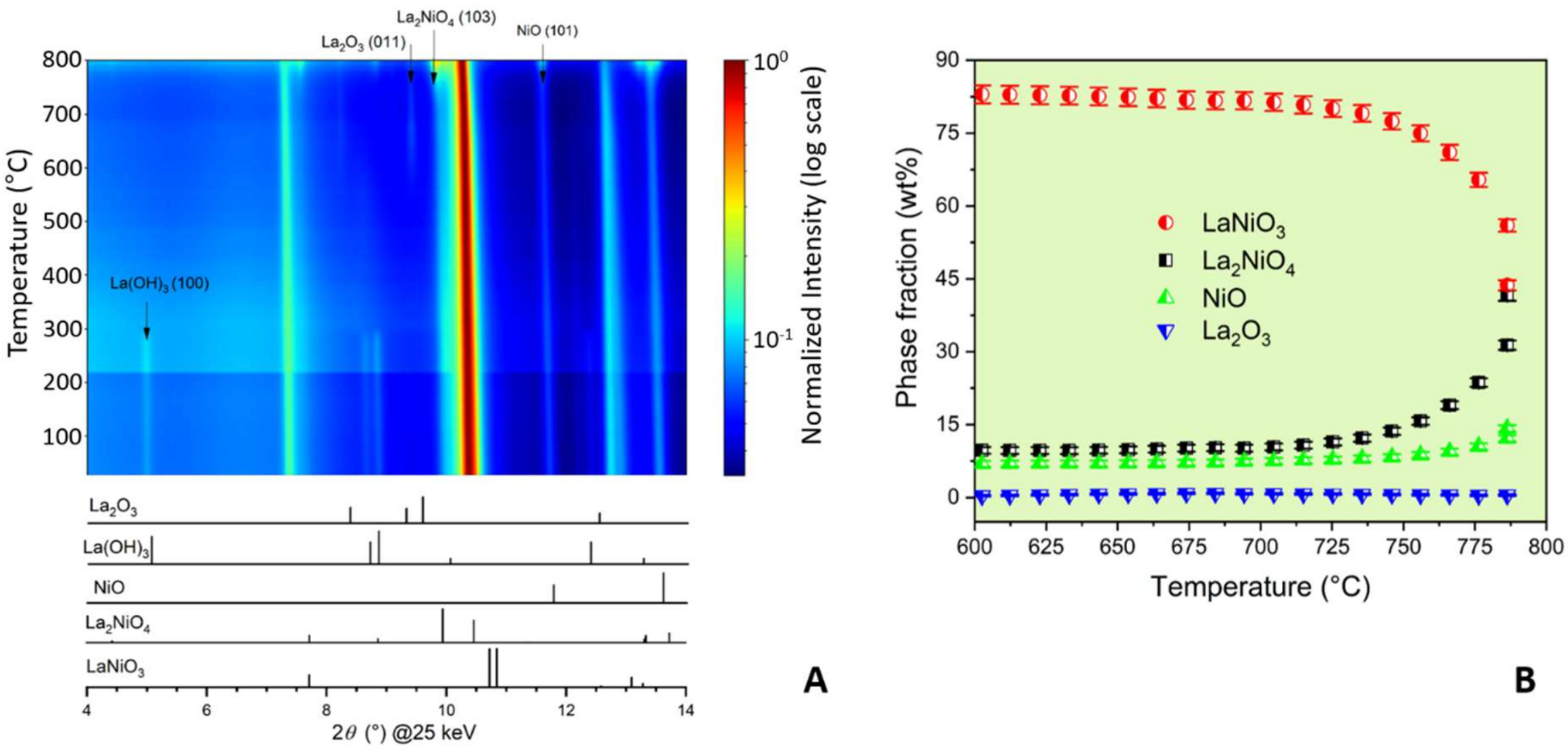

Fig. S14 | Two-dimensional contour plot of in situ collected XRD patterns during heating $LaNiO_3$ to 800 °C in He atmosphere. He was delivered as ultra-pure (99.999%). With the O_2 impurity levels, as specified by the supplier, as < 1 ppm, and the capillary operated near atmospheric pressure ($\approx 10^5$ Pa), the maximum oxygen partial pressure amounts to roughly 0.1 Pa). Panels A shows the patterns during the heating. The reference structures are shown as bars below the pattern. Panels B: weight fraction analysis obtained from Rietveld refinement of the pattern.[1,2]

Rietveld refinement of the PXRD patterns (Fig. S14) shows that $LaNiO_3$ starts to transform into La_2NiO_4 via $2LaNiO_3(s) \rightarrow La_2NiO_4(s) + NiO(s) + O(g)$ at ~760 °C, a transformation that is completed after 10 min during the isothermal step at 800 °C. The amount of NiO remains stable until ~725 °C, after which it increases due to the oxidation of metallic nickel by oxygen released during the transformation of $LaNiO_3$ into La_2NiO_4. The final phase composition of the sample after complete decomposition is 79.4 wt.-% La_2NiO_4, 20.1 wt.-% NiO and 0.5 wt.-% La_2O_3.

Section G Operando XPS measurements in O_2

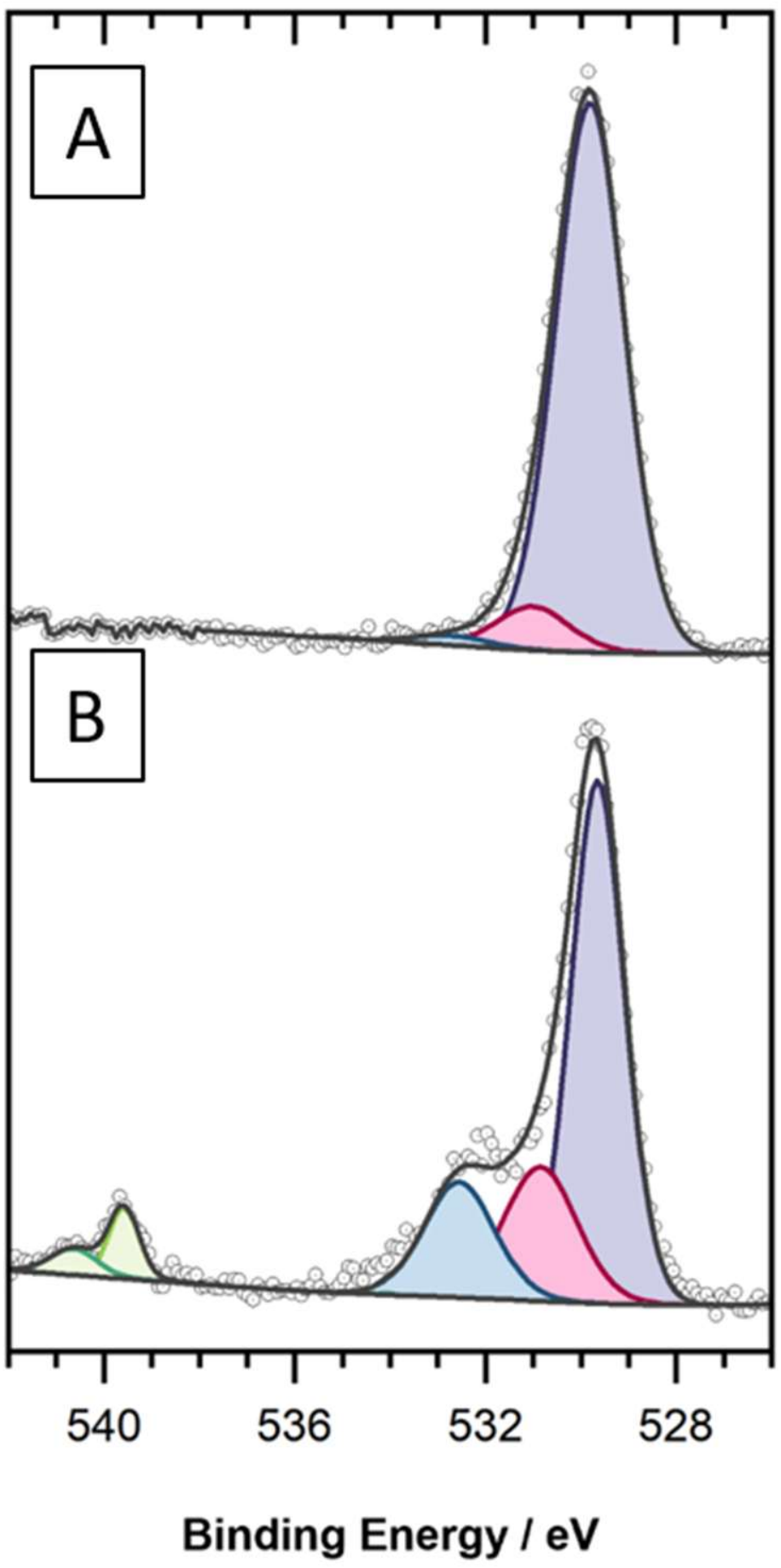

Fig. S15 | High-resolution O 1s XP spectra after 700 °C annealing *in vacuo* (Panel A, corresponding to the bottom-most spectrum in Figure 7) in comparison to after a treatment in 20 Pa O_2 for 10 min at 25 °C (Panel B). Assignment of the individual oxygen species as in Figure 7. The oxygen component at 540 eV refers to gas-phase oxygen species.

Section H Additional characterization of Ni particle formation during heating in a CO_2:CH_4 mixture without hydrogen pre-reduction

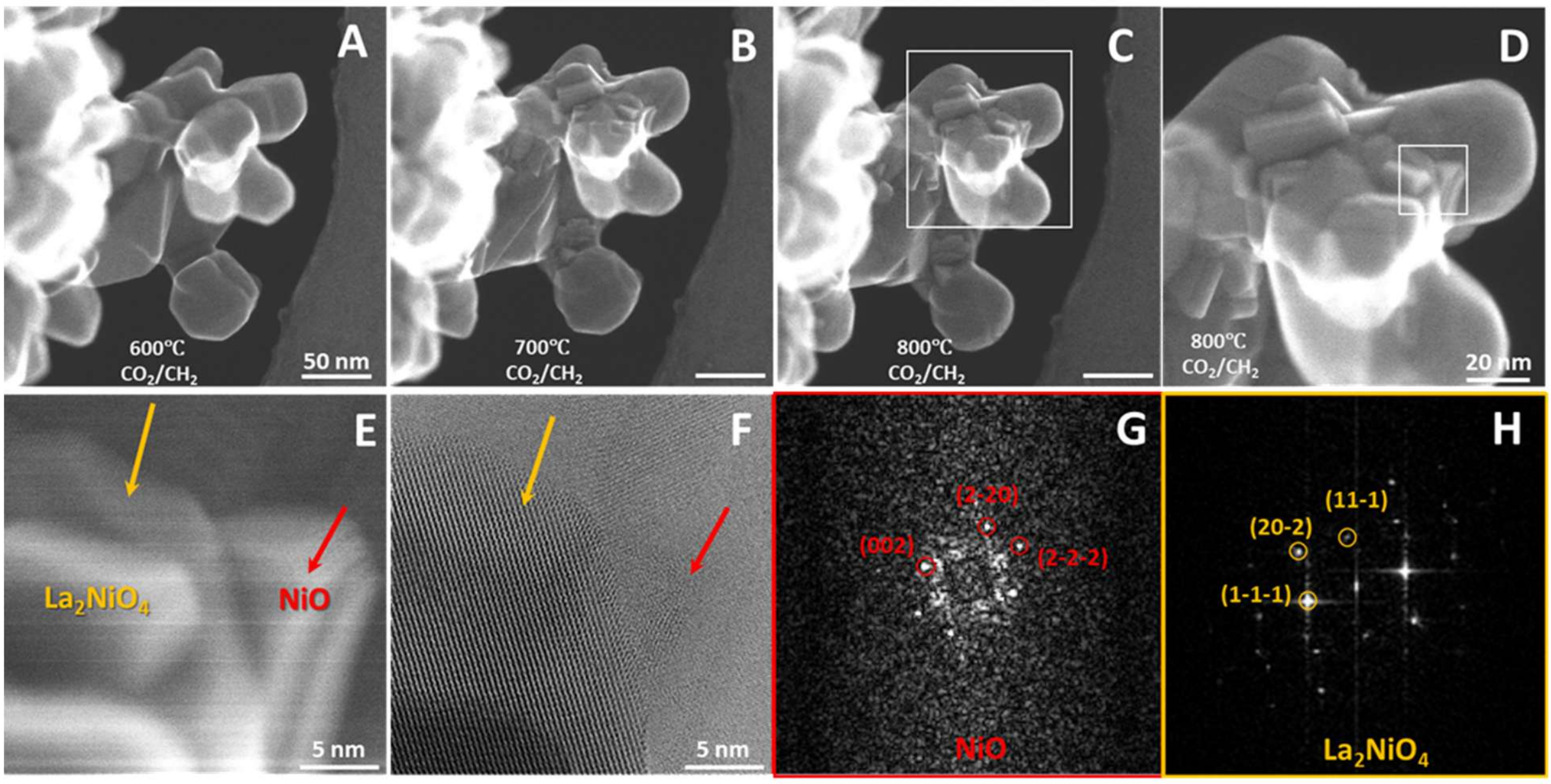

Fig. S16 | Formation of facetted NiO during heating in a DRM mixture without hydrogen pre-reduction. An In situ STEM-SE imaging series is shown during heating $LaNiO_3$ in a CO_2:CH_4 = 1:1 mixture at 600 °C (Panel A), 700 °C (Panel B), 800 °C (Panel C and D). Panel E: Enlarged SE image of the region in the white box in Panel D; Panel F: corresponding BF image of the region in white box in Panel D; Panel G and H: FFT analysis of the area indicated by a yellow and red arrow shoeing the presence of the La_2NiO_4 and NiO phase, respectively.

Section I Oxidation behavior of emerged Ni particles in pure CO_2 monitored by operando XPS

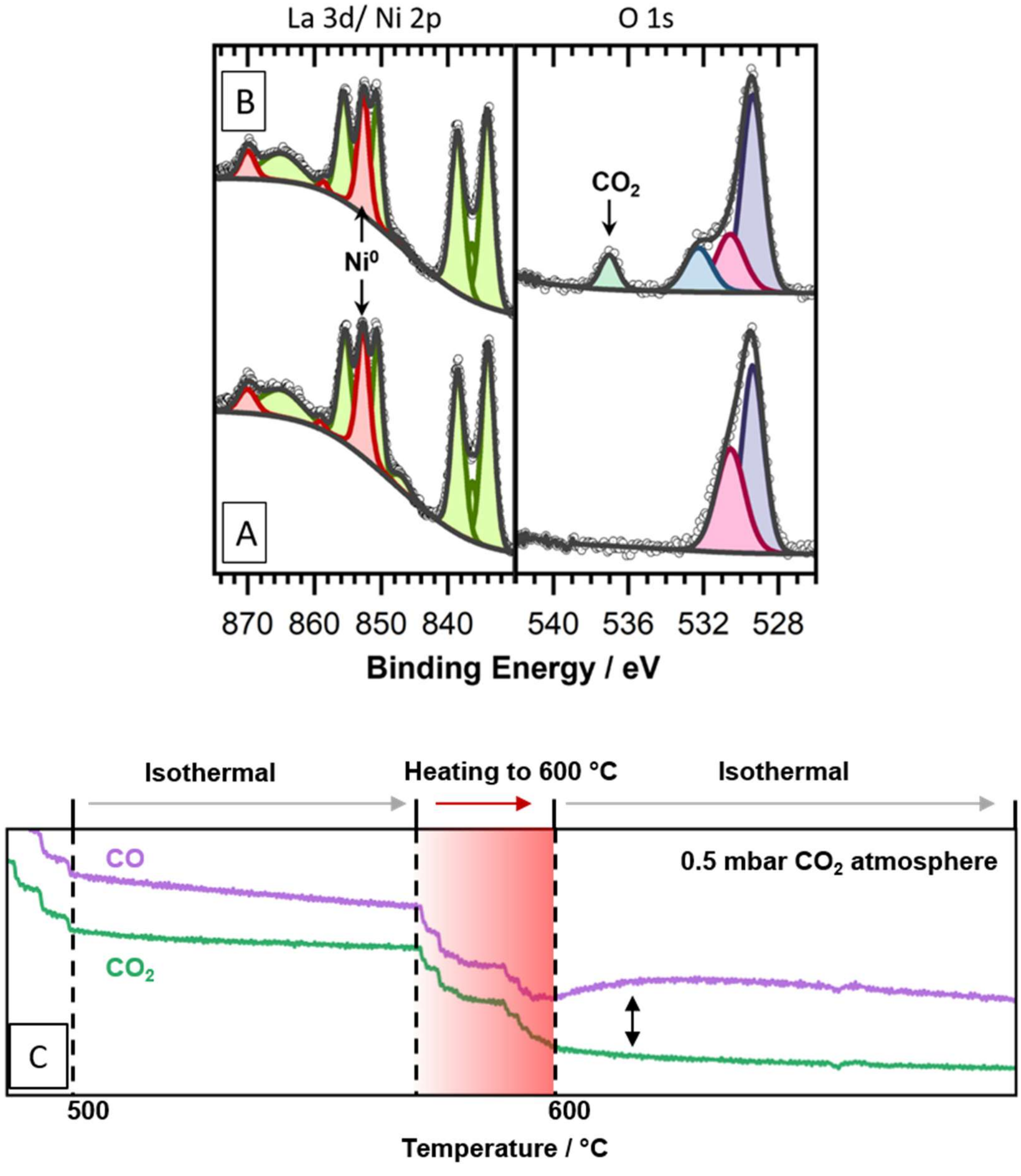

Fig. S17 | Oxygen vacancy quenching by CO_2 after H_2 reduction. High-resolution La 3d Ni 2p and O 1s XP spectra after reduction in 10 Pa H_2 (Panel A, cf. last spectrum in Figure 2, Panel A) and treatment in 50 Pa CO_2 at 600 °C (Panel B). CO_2 was admitted at 25 °C and spectra were taken in 100 °C steps up to 600 °C. The displayed spectrum is representative for all spectra. The feature

at 537 eV refers to gas-phase CO_2. Assignment of the oxygen components as in Figure 2). Panel C: Evolution of the masses m/z = 28 (CO) and m/z = 44 (CO_2) during heating between 500 °C and 600 °C and in the isothermal sections during collection of the XP spectra.

Section J Additional characterization of Ni exsolution dynamics at elevated temperatures in a CO_2:CH_4 mixture

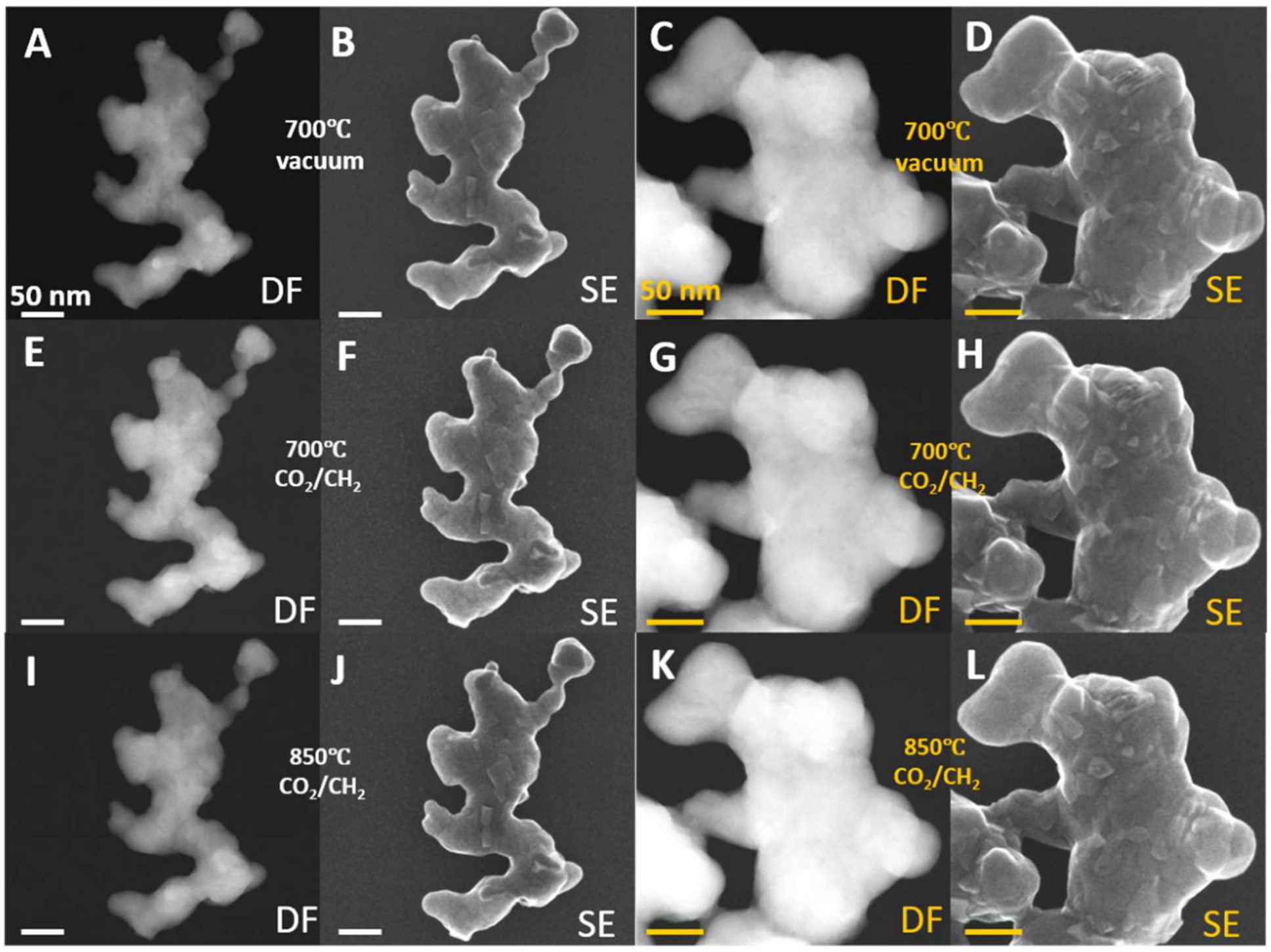

Fig. S18 | Stability of La_2NiO_4 after vacuum pre-heating and admission of a DRM mixture. DF and SE imaging of two $LaNiO_3$ grains analyzed before and after injection of a CO_2/CH_4 = 1:1 mixture. Panels A and B: sample 1 at 700 °C in vacuo; Panels C and D: sample 2 at 700 °C in vacuo; Panels E and F: sample 1 at 700 °C in CO_2:CH_4; Panels G and H: sample 2 at 700 °C in CO_2:CH_4; Panels I and J: sample 1 at 850 °C in CO_2:CH_4; Panels K and L: sample 2 at 850 °C in CO_2:CH_4.

Section K Quantification of the STEM/EDX data in Figure 10

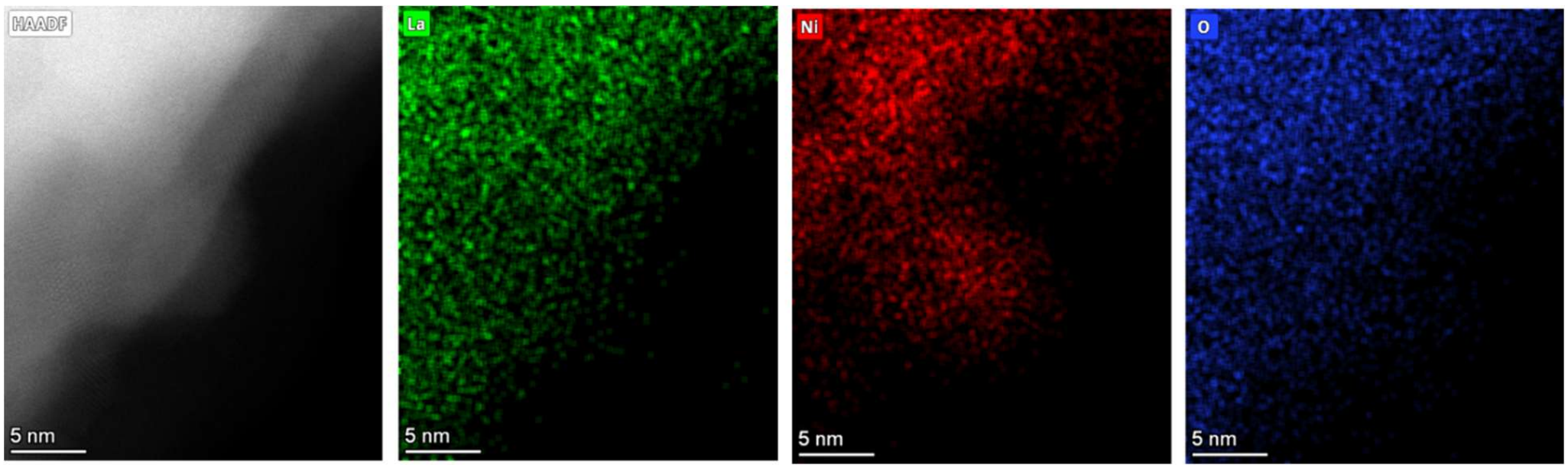

Fig. S19 | Transient formation of oxidized Ni in $LaNiO_3$ after quenching the DRM reaction under bar-level DRM conditions at 650 °C. STEM image and respective EDX maps of O, Ni and La are shown.

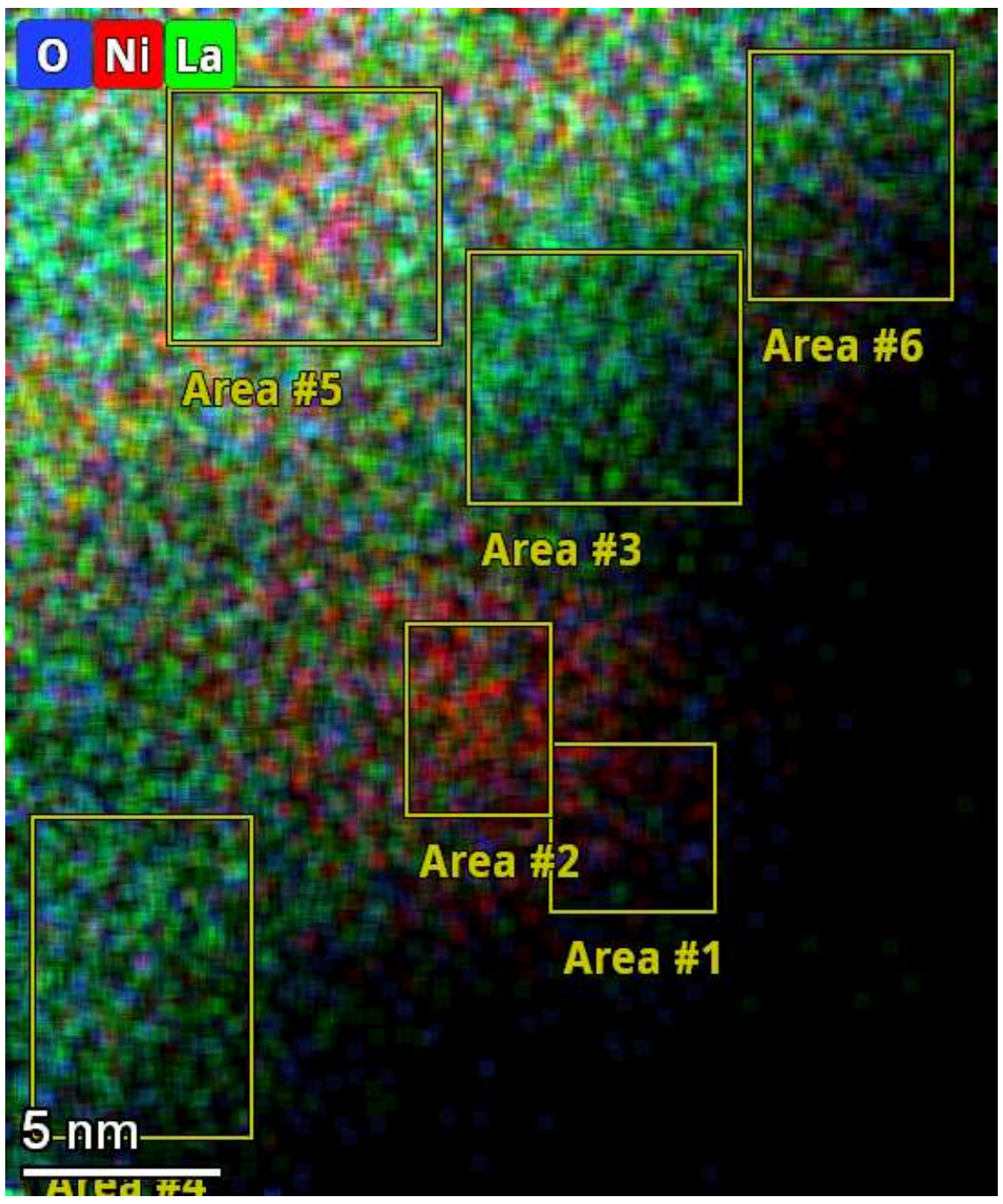

Fig. S20 | Full quantification (together with Table S3) **of different regions around the oxidized Ni particle shown in Figure 10 and Figure S18**.

Table S3: Atomic ratios of Ni, O and La derived by EDX mapping of different regions around the oxidized Ni particle shown in Figure 10 and Figure S23.

Atomic Fraction (%)/Element	Ni	O	La
Area 1	42.11±4.91	54.77±4.9	3.12±1.20
Area 2	50.98±4.00	39.20±3.42	9.82±1.31
Area 3	5.38±0.83	71.85±1.93	22.77±1.94
Area 4	5.68±0.88	71.00±1.95	23.32±1.97
Area 5	28.32±3.02	56.47±2.54	15.21±1.53
Area 6	12.41±1.69	69.94±1.96	17.65±1.64

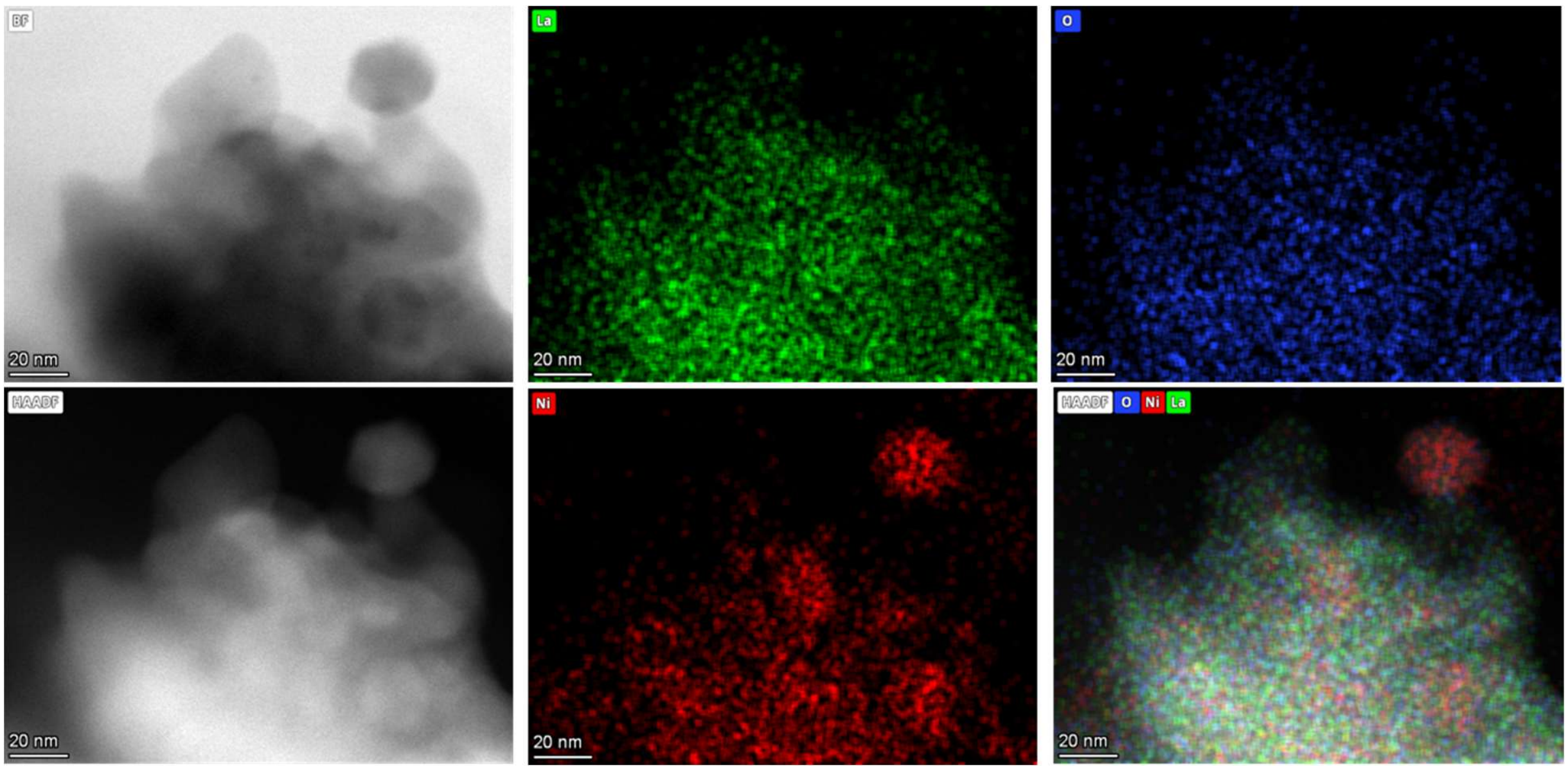

Fig. S21 | Formation of metallic Ni in $LaNiO_3$ after quenching the DRM reaction under bar-level DRM conditions at 785 °C. STEM/HAADF image and respective EDX maps of O, Ni and La are shown, alongside bright-field (BF) image.

Section L Additional structure and catalytic DRM data und detailed discussion of catalytic properties

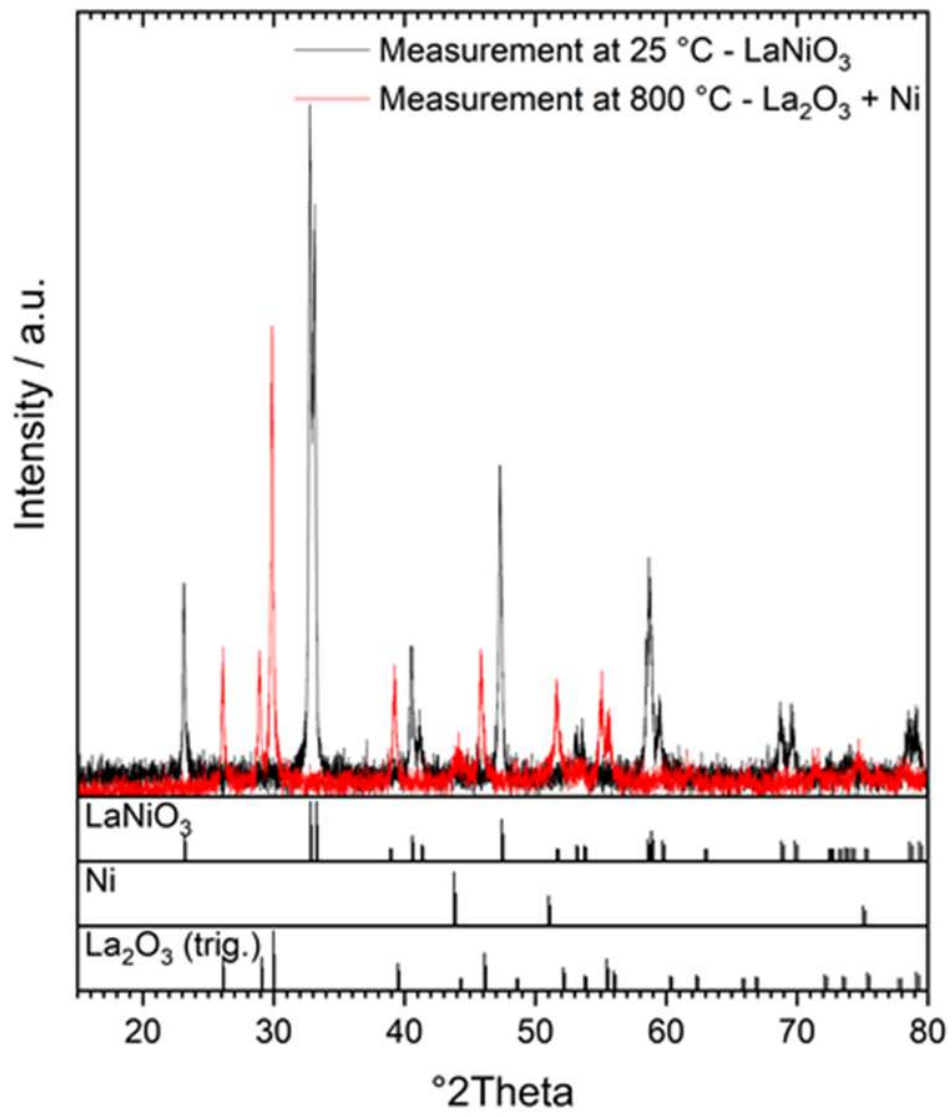

Fig. S22 | Final decomposition of $LaNiO_3$ into Ni and La_2O_3 after direct activation in the DRM mixture. Comparison of the PXRD patterns in situ collected at 25 °C and 800 °C (cf. Figure 10)

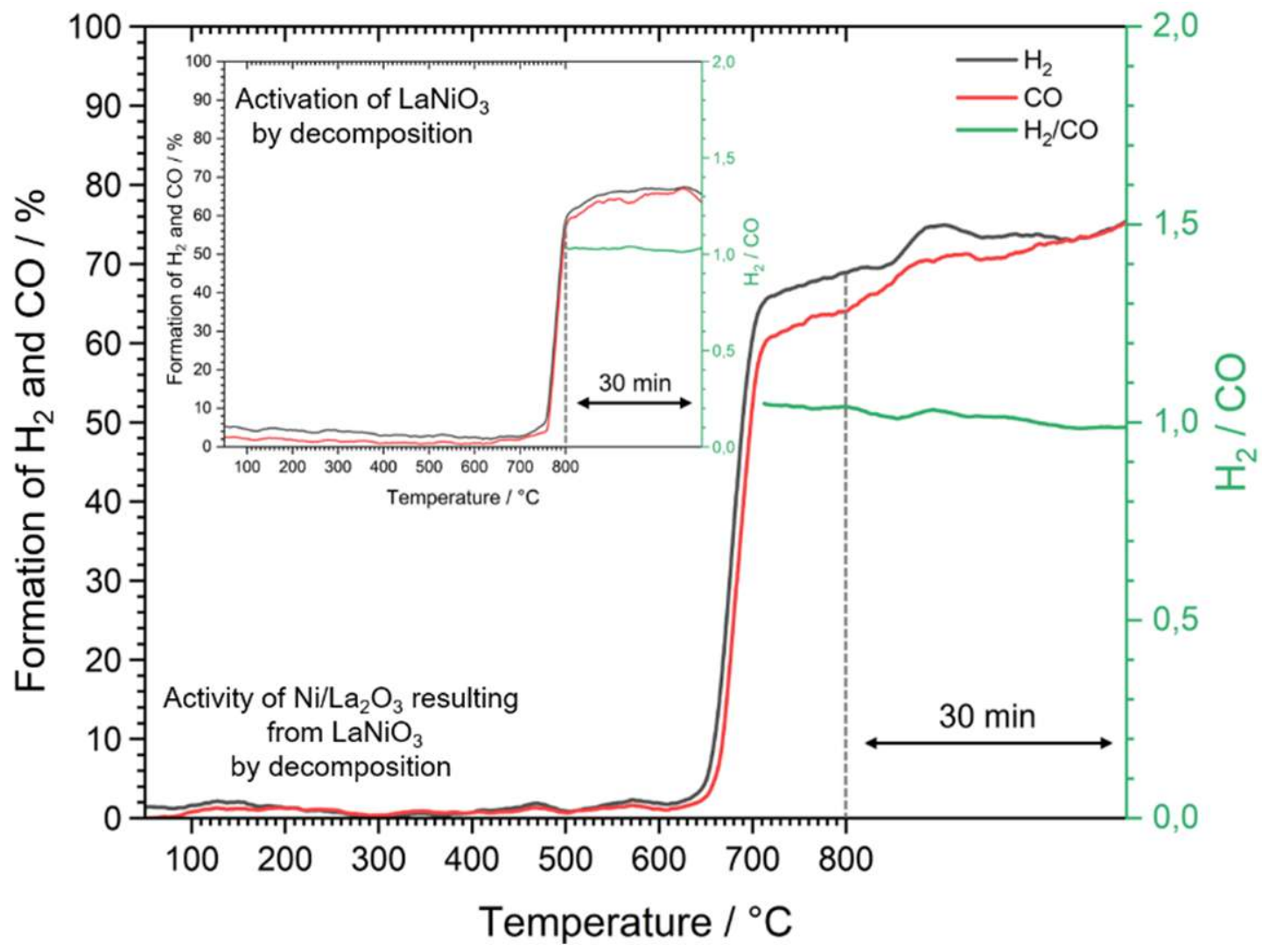

Fig. S23 | Activation of $LaNiO_3$ directly in the DRM mixture and intrinsic DRM activity of the evolved Ni-La_2O_3 interface. *Small Panel*: Catalytic DRM profiles during $LaNiO_3$ decomposition in a CO_2:CH_4 = 1:1 mixture (500 Pa reactant pressure each, helium added to 10^5 Pa pressure). *Large Panel*: Catalytic DRM profiles in a CO_2:CH_4 = 1:1 mixture (500 Pa each, helium added to 10^5 Pa pressure) on Ni-La_2O_3 resulting from $LaNiO_3$ decomposition. Heating rate: 5 °C min^{-1}, 200 mg catalyst

As for a detailed discussion of the catalytic properties outlined in Figure 10 (main paper), we note that the two DRM profiles shown in **Figure 10A** and **B** were conducted in the fixed-bed flow reactor. Without hydrogen pre-reduction, catalytic activity starts at approximately 600 °C in both experiments. CO and H_2 formation accelerates at roughly the same temperatures. Characteristic changes in the slopes of the catalytic profiles, which we associate with the intermediate formation of oxidized Ni, are present in both measurements. For the DRM experiment in $5 \cdot 10^5$ Pa CO_2/CH_4, the H_2 formation is lower compared to CO in the activation period between 600 and 800 °C, which matches the associated decrease in the CH_4 signal. We address this issue to increased interaction and consume of H_2 with/from the perovskite (e.g., further reduction or

dissolution). Once a reaction temperature of 800 °C is reached, stable DRM activity with an apparent H_2/CO ratio of unity (as expected for exclusive DRM activity) is observed of the course of the 30 min isothermal period. For the respective measurements at $5 \cdot 10^4$ Pa CO_2/CH_4, the features are less pronounced and the CO/H_2 profiles more or less follow the same trend, although also in this case, the H_2 formation is slightly lower than CO up to 800 °C, where again stable DRM activity is observed, as judged by the H_2/CO ratio of unity. The dotted orange and red lines in the catalytic profiles indicate the temperatures, where the reaction was quenched to verify the presence of oxidized nickel (650 °C) and 785 °C (metallic nickel). Quenching experiments have been performed for the sample treated in $5 \cdot 10^5$ Pa CO_2/CH_4 to maximize the expected oxidation behavior. Indeed, the ex situ STEM images, alongside the respective EDX maps, reveal that this is the case. To verify that the activity trends can be extrapolated to model DRM pressure regimes in the low-Pa regime, Fig. 10B shows operando-measured DRM profiles during the XPS analysis. For these experiments, the sample was exposed to the DRM mixture at 50 Pa CO_2/CH_4 reactant pressure and the masses at relevant m/z ratios monitored during measuring. Evidently, also under these conditions, DRM activity is observed and starts at approximately 650 °C, where it accelerates, as the reaction temperature is raised. The wobbly shape of the CO_2 and CH_4 signals is due to the pumping of the reactant gases. Whereas CO and H_2 are formed and only pumped away, CO_2 and CH_4 are simultaneously fed and pumped away.

Finally, Fig 10C shows that also under operando PXRD measurements DRM activity is observed at 500 Pa reactant pressure of CO_2/CH_4. DRM activity starts at around 580-600 °C at saturates at around 800 °C. Structurally, crystalline La_2NiO_4 and Ni as decomposition products of $LaNiO_3$ are detected at around 730 °C, before at ca. 780 °C, crystalline Ni and La_2O_3 start to emerge. At 800 °C, only metallic Ni and La_2O_3 are observed (Fig. 21). Note, that although no

changes in the PXRD patterns are observed between 600 and 730 °C, DRM activity arises. To resolve this apparent contradiction, we have performed operando XPS analysis in a 20 Pa CO_2/CH_4 DRM mixture (insets in Figure 10C) and revealed, that already at 400 °C, surface-bound Ni emerges, which accordingly drives the catalytic activity.

Section M Pore-Size distribution and cumulative pore volume

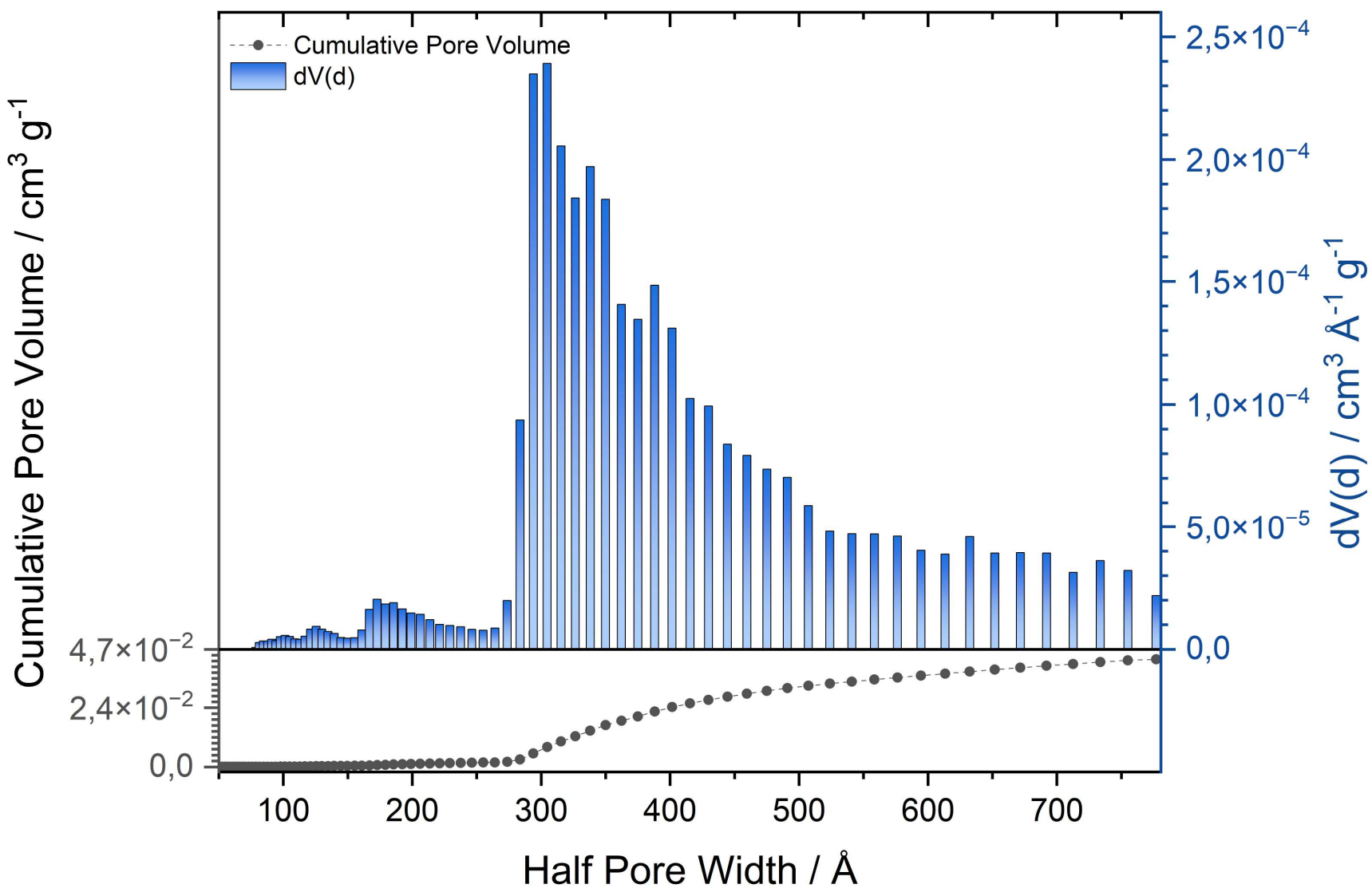

Fig. S24 | Cumulative pore volume, as well as pore size distribution, for the $LaNiO_3$ sample of this work.

Section N Characterization of the initial $LaNiO_3$ state

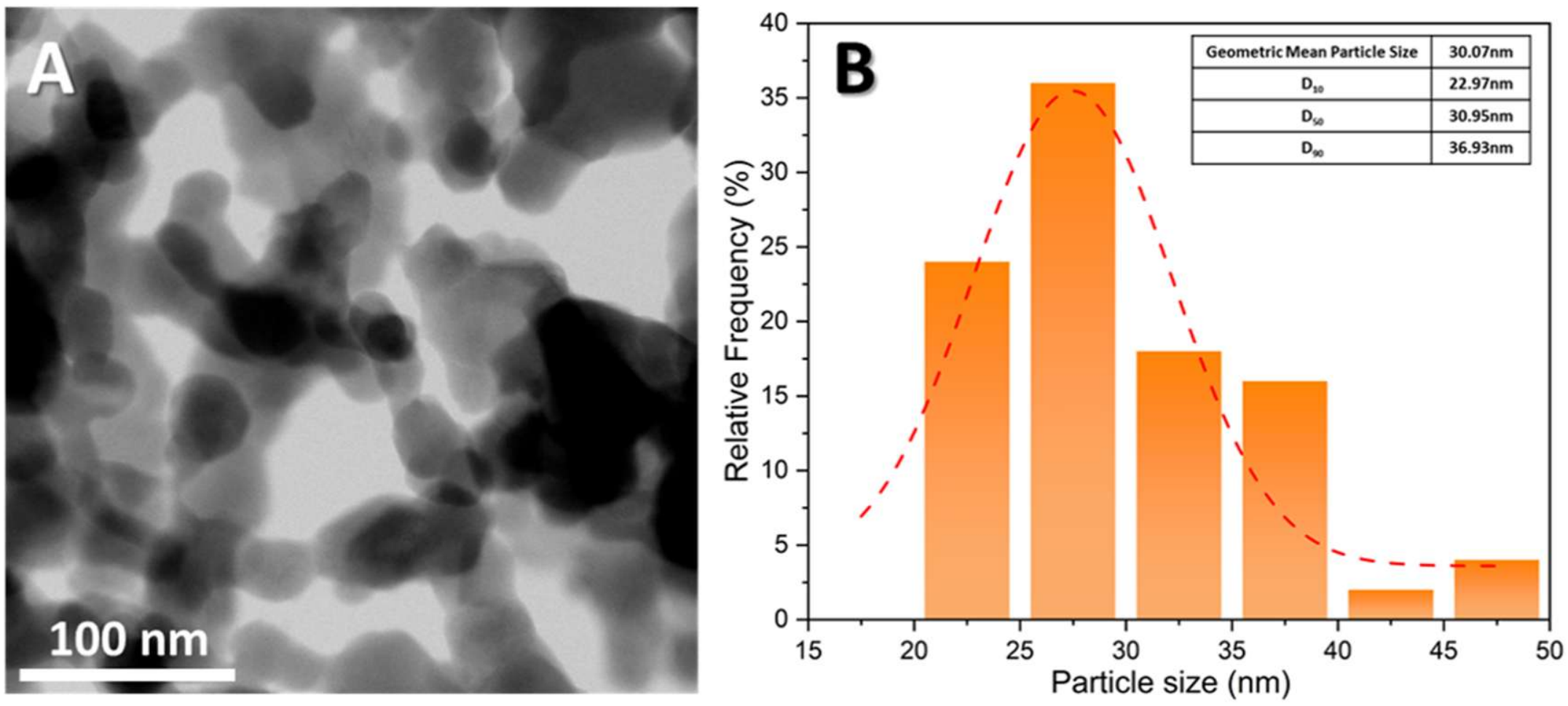

Fig. S25 | Representative low-magnification BF image of $LaNiO_3$ particles after synthesis (Panel A), **statistical analysis of particle size** (>200 particles measured, Panel B). A maximum of particle size is found at approximately 27 nm.

Section O Details of the in situ heating experiments

The tip of the MEMS-based heating holder and chip (Norcada Inc., Edmonton, Canada) are shown in **Figure S25**. The homogeneously heated region is approximately 50 μm. Temperature calibration is performed by the MEMS-chip manufacturer and a heater temperature accuracy of ±1 °C is specified. We note that the actual temperature can differ from the manufacturer's specification and strongly depends on mounting or thermal contact. To obtain reproducible results, we have repeated our in situ TEM experiments in various atmospheres. A detailed overview of the number of repeats is presented in **Table S4**. The temperature was usually continuously increased from 200 °C to 800 °C at a heating rate of 10 °C min^{-1}. After each 50 °C increment, the specimen was held isothermally for 10 - 30 min and images were also collected at 50 °C intervals.

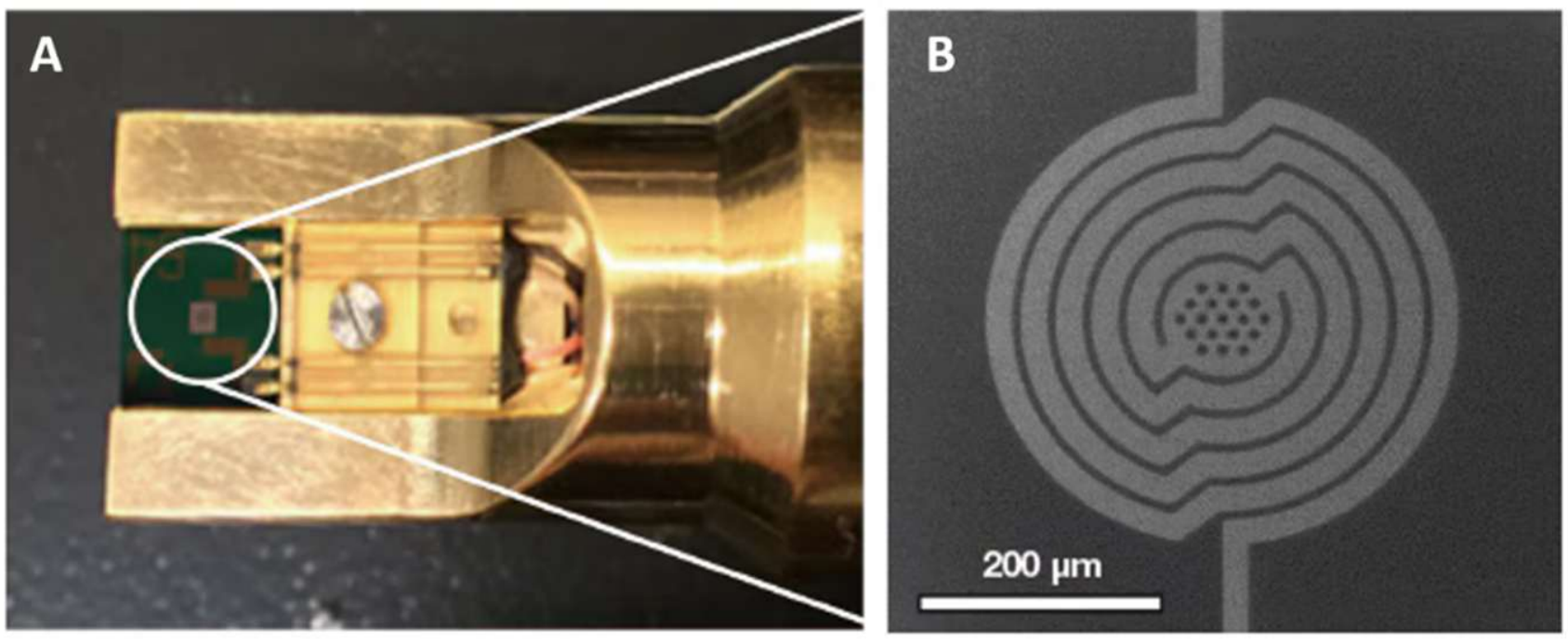

Fig. S26 | MEMS-based heated specimen holder for atomic-scale observations. Panel A: Overview of the specimen mount. Panel B: SEM image of the MEMS-based heating unit.[3]

Table S4: Number of independent repeats per atmosphere.

In situ TEM experiments		
Conditions	Number of independent repeats	Notes
Vacuum	5	RT – 800 °C: 4x<br>RT – 900 °C: 1x
H_2	3	RT – 800 °C: 3x
$CH_4 + CO_2$	3	RT – 800 °C: 2x<br>RT – 900 °C: 1x
Vacuum followed by $CH_4 + CO_2$	2	RT – 800 °C: Vacuum<br>800 °C - 600 °C: Vacuum<br>600 °C - 800 °C: $CH_4 + CO_2$
H_2 followed by $CH_4 + CO_2$	2	RT – 800 °C: H_2<br>800 ℃ - 600 °C:H_2<br>600 °C - 800 °C: $CH_4 + CO_2$

Section P Details of $LaNiO_3$ after heating to 400 °C *in vacuo* and effect of beam irradiation

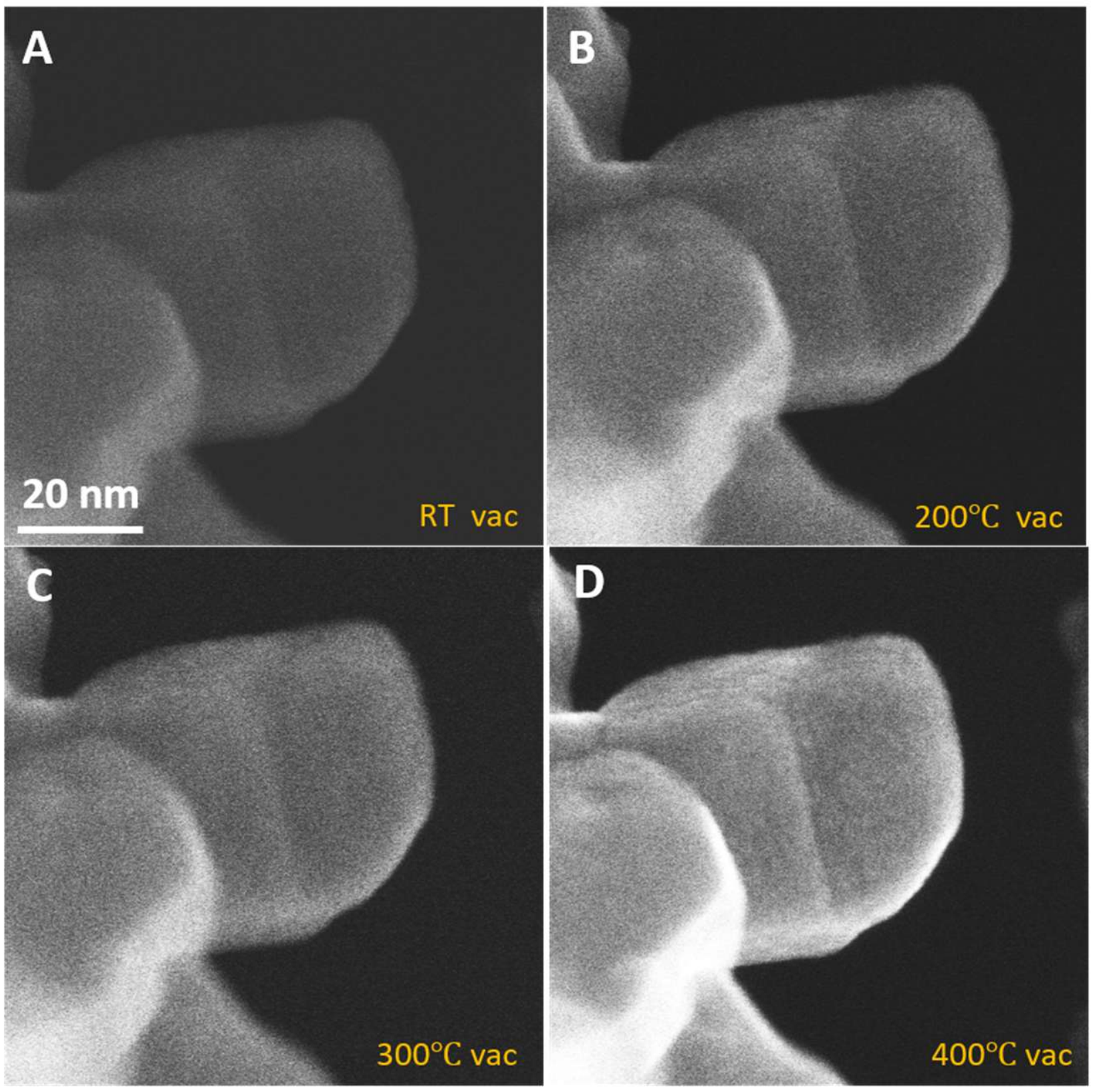

Fig. S27 | Electron-beam irradiation analysis of $LaNiO_3$ heated *in vacuo* from 25 °C to 400 °C.

Panel A: 25 °C; Panel B: 200 °C; Panel C: 300 °C; Panel D: 400 °C

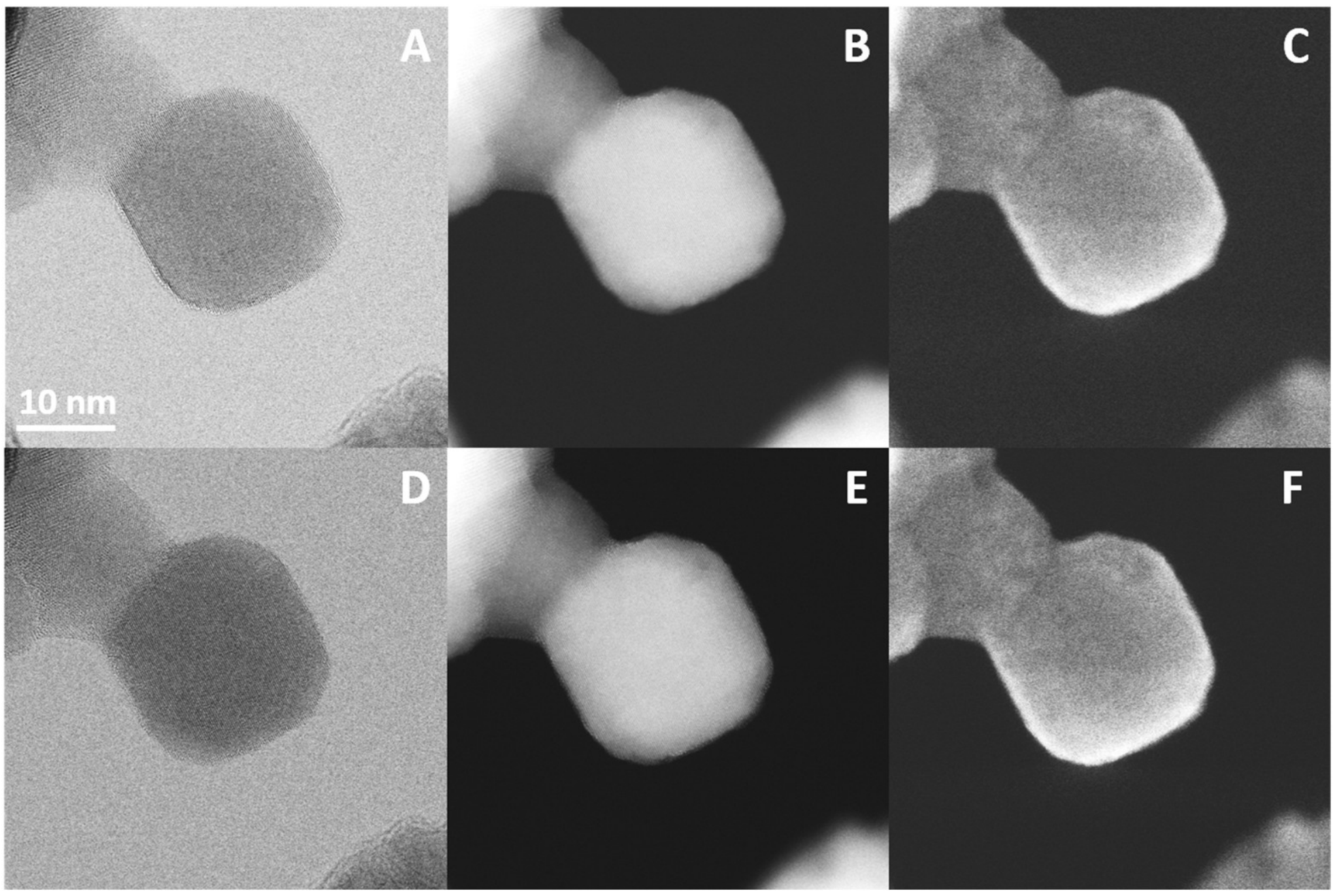

Fig. S28 | Simultaneously recorded BF, DF and SE images of $LaNiO_3$ particles during heating in vacuo. Top row, Panels A-C: BF image, DF image, and SE image at 25 °C. Bottom row, Panels D-F: BF image, DF image, and SE image at 200°C.

Section Q Thermodynamic calculations related to the oxygen partial pressure

Cantera, an open-source chemical kinetics software (https://github.com/Cantera/Cantera), was used to investigate the influence of partial oxygen pressure on Ni formation from $LaNiO_3$. The calculation is based on the law of mass action and the change in Gibbs free energy (ΔG°) for the decomposition:

$$2LaNiO_3(s) \rightarrow La_2NiO_4(s) + Ni^0(s) + 2O(g)$$

The standard formation enthalpies ΔG° and entropies ΔS^0 were adopted from thermodynamic databases. The critical oxygen partial pressure, P_{O2}, at specific temperature T is determined by

$$\Delta G(T, p_{O2}) = \Delta G°(T) + \frac{3}{2} RT \ln(\frac{p_{O2}}{p°})$$

Here, ΔG°(T) is the standard Gibbs free-energy change at T, $p°$ is the standard pressure, R is the gas constant. By applying van't Hoff equation:

$$\Delta G°(T) = \sum G_{products} - \sum G_{reactants}$$

to simulate the heating process, we initialized the thermodynamic state of all species at standard pressure ($p°$=1 atm) and then iteratively increasing the temperature from 100 °C to 800 °C.

Table S5: Critical oxygen partial pressure for Ni exsolution from $LaNiO_3$

Temperature (°C)	O_2 partial pressure in vacuum (Pa)	Critical O_2 partial pressure (Pa)
100	$1*10^{-7}$	$2.6*10^{-41}$
200	$1*10^{-7}$	$2.6*10^{-29}$
300	$1*10^{-7}$	$2.6*10^{-22}$
400	$1*10^{-7}$	$2.6*10^{-16}$
500	$1*10^{-7}$	$2.6*10^{-12}$
600	$1*10^{-7}$	$2.6*10^{-9}$
700	$1*10^{-7}$	$2.6*10^{-7}$
800	$1*10^{-7}$	$2.6*10^{-5}$

References:

[1] Doran, A., L. Schlicker, C.M. Beavers, S. Bhat, M.F. Bekheet, and A. Gurlo, *Review of Scientific Instruments* **2017**, *88*, 013903.
[2] Schlicker, L., A. Doran, P. Schneppmüller, A. Gili, M. Czasny, S. Penner, and A. Gurlo, *Review of Scientific Instruments* **2018**, *89*, 033904.
[3] High-Tech, H.; Available from: https://www.hitachihightech.com/global/en/sinews/technical_explanation/130317/
