# Supplementary material for: In situ electron microscopy of LaNiO3 transformation during reduction and methane dry reforming
Source: Nat Commun. 2026 Aug 7;17:9487. doi: 10.1038/s41467-026-76419-1 (PMC13542283; doi:10.1038/s41467-026-76419-1)
Supplement: Supplementary file 2 [file 41467_2026_76419_MOESM2_ESM.pdf]

Description of Additional Supplementary Files

File Name: Supplementary Movie 1

Description: In situ STEM-SE movie of Ni particle sintering at 600°C in H_2.

File Name: Supplementary Movie 2

Description: In situ STEM-SE movie of particle migration at 700°C in H_2.

File Name: Supplementary Movie 3

Description: In situ STEM-SE movie showing structural evolution of LaNiO3 during exposure to a CH4:CO2 = 1:1 at 600°C.

File Name: Supplementary Movie 4

Description: In situ STEM-SE movie showing dynamic fragmentation of NiO particles during exposure to a CH4:CO2 = 1:1 at 700°C.
