# Supplementary material for: In situ electron microscopy of LaNiO3 transformation during reduction and methane dry reforming
Source: Nat Commun. 2026 Aug 7;17:9487. doi: 10.1038/s41467-026-76419-1 (PMC13542283; doi:10.1038/s41467-026-76419-1)
Supplement: Supplementary file 3 [file 41467_2026_76419_MOESM7_ESM.pdf]

nature portfolio

Peer Review File

In situ Electron Microscopy of LaNiO3 Transformation during Reduction and Methane Dry Reforming

Corresponding Author: Dr Marc Heggen

Parts of this Peer Review File have been redacted as indicated to remove third-party material.

This file contains all reviewer reports in order by version, followed by all author rebuttals in order by version.

Version 0:

Reviewer comments:

Reviewer #1

(Remarks to the Author)
In this manuscript, Cao et al. focuses on the structural evolution behavior of LaNiO3 perovskite catalyst during DRM reaction. Through in-situ TEM characterization, the study elucidates the structural transformations of LaNiO3, nickel exsolution phenomena, and associated kinetic processes under various gaseous environments, providing crucial insights into the complex behavior of perovskite catalysts under realistic reaction conditions. However, the main points of the manuscript are excessively dispersed, and the scientific significance requires further refinement and consolidation. Some suggestions for improvement are listed below.

1, The manuscript presents an excess of experimental details, which, at times, prove to be a source of distraction and detraction from the clarity of the central scientific arguments. In order to facilitate the identification of the key findings and mechanistic insights, it is necessary to present the material in a more concise and focused manner. Moreover, the narrative would benefit from enhanced logical coherence between sections, as the current text structure tends to list results without sufficiently integrating them into a cohesive storyline.
2, The in-situ TEM experimental pressure significantly differs from the actual DRM operational pressure; It is essential to elucidate how the conclusions drawn from these in-situ experiments accurately reflect the phenomena occurring during the catalytic process.
3, The author puts forward the hypothesis that the particle coarsening observed in a hydrogen atmosphere above 550°C is attributable to Ostwald ripening. Nevertheless, the potential for additional nickel precipitation due to elevated temperatures has not been sufficiently excluded. It is therefore supplementary in-situ experiments with high spatial and temporal spatial resolution are needed.
4, On page 12, line 227, the author asserts that particles precipitate within the substrate; however, neither STEM SE images nor DF images can definitively determine whether particles are located on the surface, within the interior, or on the reverse side of the substrate. The description associated with Figure 8B exhibits the same issue.
5, The vacuum heating subsection is not directly related to the catalytic reaction and does not contribute to the main arguments of the manuscript; therefore, it is unnecessary to include this discussion in the main text.
6, The authors suggest that Figure 9 represents the OA process; however, particles 1, 2, and 6 do not exhibit significant size changes between 550 and 650°C. The determination of whether the disappearance of particles is not attributable to new structural evolution induced by temperature elevation remains unclear.

Reviewer #2

(Remarks to the Author)
Well-designed study with sophisticated in-situ techniques and clear research objectives. The work addresses an important gap in understanding perovskite catalyst behavior. The microscopy convincingly captures qualitative pathways (Ni exsolution, $LaNiO_3 \rightarrow La_2NiO_4$) but, in its current form, the study does not yet provide sufficient quantitative or catalytic evidence to support the broader claims about DRM activity and industrial relevance. Addressing the points above would greatly enhance the manuscript’s rigor and impact.
Introduction

A critical conceptual gap in this work is the inadequately distinguish between solid-state recrystallization and true exsolution, which are fundamentally different processes with distinct mechanistic and practical implications. What the authors describe as "exsolution" is actually solid-state recrystallization occurring in their stoichiometric $LaNiO_3$ starting material, which results in complete lattice restructuring to form the La_2NiO_4 Ruddlesden-Popper phase and creates new "socket" sites with different anchoring properties. This process allows for potential reversibility under certain conditions and represents a phase transformation rather than true exsolution. In contrast, genuine exsolution involves non-stoichiometric perovskites (A-site deficient) that form nanoparticles while maintaining the same residual host lattice structure, resulting in minimal structural reorganization, potentially greater long-term stability, but typically irreversible behavior due to the maintained lattice framework. This distinction is particularly important when evaluating the authors' claim on page 4 that "hydrogen treatments at elevated temperatures induced a total collapse of the perovskite structure, triggering the formation of Ni in contact with La_2O_3" - this statement is not generally applicable to all perovskite systems but is specifically characteristic of stoichiometric starting materials undergoing recrystallization. Since most recent exsolved catalysts often employ non-stoichiometric compositions designed for true exsolution, the observed recrystallization behavior may be less relevant than claimed, and the authors should qualify their conclusions about general "perovskite behavior" accordingly while acknowledging that their mechanistic insights apply specifically to stoichiometric recrystallization processes.
Good literature survey of exsolution studies, but the section repeatedly cites earlier work to justify catalytic relevance while the present study contributes no new catalytic testing (activity, selectivity, stability).
Methodology
LaNiO3 synthesis – potential reproducibility issues No particle-size or surface-area data for the as-prepared powder; these affect reduction kinetics.
TEM Only a single low-T control (200 °C) is presented to argue that electron-beam irradiation is harmless. No controls at ≥400 °C where reduction clearly accelerates.
Heating rates in the MEMS chip, actual temperature calibration, and the number of independent repeats per atmosphere are not stated.
Results
First Ni appears at 400 °C in SE images (Fig. 1) and even at 300 °C in XPS. The disparity between 1.5 Pa (TEM) and 20 Pa (XPS) H_2 runs is not discussed; pressure strongly affects oxygen removal.
Particle-size distribution is shown only in an inset (single field of view) with no statistical treatment or error bars.
On page 10, the authors attribute the formation of different decomposition products (La_2NiO_4 under DRM conditions versus metallic Ni + La_2O_3 under hydrogen) to kinetic effects, arguing that "reduction is slowed down" under DRM conditions. However, this interpretation misses the more fundamental thermodynamic explanation. The primary distinction is thermodynamic, not kinetic - the vastly different oxygen partial pressures (pO_2) in these environments dictate different equilibrium product distributions. Under hydrogen's highly reducing conditions (very low pO_2), metallic Ni + La_2O_3 are thermodynamically favored, while the moderately oxidizing DRM atmosphere (higher pO_2 due to CO_2) thermodynamically stabilizes the intermediate La_2NiO_4 phase. While reaction kinetics may differ between atmospheres, the fundamental reason for different final products is that each environment drives the system toward different thermodynamic endpoints determined by pO_2-dependent phase stability relationships.
Quantitative gap. Claims of "high-density" Ni are not backed up by areal number densities.
Observation of core–shell Ni@NiO relies on a single particle cross-section (Fig. 3 & Fig. S2). No statistics on how widespread this motif is.
La_2NiO_4 onset (~760 °C) is supported by ex-situ synchrotron XRD in He, not vacuum. Substituting He for high vacuum may shift the equilibrium oxygen chemical potential; the authors do not justify this proxy.
EDX semi-quantification: counts are shown qualitatively (colour maps) but not converted to Ni/La atomic ratios.
On page 21, the authors attribute the observed mobility of emergent nanoparticles to inherent Ni behavior, but this is more likely due to altered metal-support interactions resulting from the structural transformation. Since the host lattice recrystallizes from $LaNiO_3$ to the La_2NiO_4 Ruddlesden-Popper phase, this creates entirely new interfacial properties and bonding environments for the nanoparticles compared to true exsolution systems where the original host lattice is maintained along with established crystallographic alignment relationships. The enhanced particle mobility observed is therefore likely a consequence of weaker or different interfacial interactions with the newly formed La_2NiO_4 support rather than an intrinsic property of the Ni particles themselves.
Ni exsolution is "suppressed" up to 700 °C, but again no kinetics or oxygen partial-pressure calculations are supplied to generalize these findings.
Conclusion
Key claim: oxidation of Ni to NiO around 600–650 °C is the root cause of the activity dip in DRM catalysts. Evidence gap: No catalytic tests, no CO_2/CH_4 conversion data, and no in situ spectroscopy correlating Ni valence with CH_4 activation rates. Additional experiments (e.g., operando DRIFTS, flow-reactor tests) are needed.
Real-world relevance. The authors acknowledge that their reduced partial pressures were chosen "to slow down the transformation processes" , but do not revisit whether kinetics or phase equilibria at 1-2 Pa are transferable to bar-level DRM.
Clarify oxygen chemical potentials. Calculate pO_2 for the 1.5 Pa DRM mixture and discuss how it compares to industrial feeds; consider a second set of experiments at ≥100 mbar to test robustness.
Correct minor editorial issues (duplicate "in-situin-situ", inconsistent figure numbering, units).

Reviewer #3

(Remarks to the Author)
This study provides important insights into the structural evolution and Ni exsolution behavior of LaNiO3 perovskite under various gas environments relevant to dry reforming of methane (DRM). The in-situ electron microscopy approach is

technically impressive and yields valuable data. However, the manuscript requires major revision prior to publication. The following comments highlight issues that must be addressed to enhance clarity, consistency, and scientific rigor.

The manuscript contains multiple typographical inconsistencies, particularly with the term “in-situ,” which is repeated redundantly in several places (e.g., p3 – line 58, p4 – lines 77 and 83). Other minor errors also require correction (e.g., p5 – line 112, p13 – Figure 3J, p15 – line 279).

Further detail is required in the Experimental Section. In particular:
• Please specify the vacuum conditions—was the pressure of 1.5 Pa under air, an inert gas, or a high vacuum below 1.5 Pa?
• Was the in-situ TEM system operating under static or flowing gas conditions?

The authors should clearly state the amount of catalyst loaded on the TEM grid, as well as the molar quantity or flow rate of gas present in the chamber. Without this, catalytic interpretation is incomplete. For example, if 10 mL/min of reactant gas is used on a catalyst with a WHSV of 60,000 mL/g hr and 1 g catalyst, only 1% of its theoretical capacity is needed to convert all reactants—making apparent activity or stability potentially misleading. If the gas volume is too small relative to the catalyst's capacity, any claims about coking resistance could be overstated.

Although the authors claim to decouple thermal, reductive, and DRM-induced effects, the phenomena under DRM conditions seem to resemble vacuum annealing more than hydrogen reduction. The discussion of these observations is limited, and further analysis is necessary to clarify whether unique reactions occur under DRM. Suggested additions include measuring pH2 in the CO2/CH4 mixture and conducting mass change analyses.

It is well known that LaNiO3 decomposes into Ruddlesden–Popper phases and NiO at high temperature or low pO2. While the authors acknowledge this, further clarification is needed:
• Under vacuum or DRM conditions, is NiO formation driven purely by metal–support interaction, or are there additional factors not explained by thermodynamics?
• Since La2NiO4 and NiO are also present under vacuum, can CO2 dissociation alone explain NiO formation during DRM?
• Is there supplementary data for vacuum treatment at 600–700 C following 700 C H2 annealing? This could help distinguish thermally induced from DRM-specific effects.

In Figure 2, the authors interpret phase transformation based on changes in the La 3d 3/2 peak. However, this peak overlaps with Ni 2p, complicating interpretation. Why is there no discussion of the La 3d 5/2 peak, which should be more isolated? The explanation could be strengthened by revisiting this analysis.

The expression “local exsolution” used in Figure 5 to describe behavior at 750 C is ambiguous. If exsolved particles are not detected by surface-sensitive SE imaging, can their presence be asserted? Are they instead located beneath the surface? More discussion or complementary data is needed.

Figure S8 shows the appearance of La2NiO4 at 500 C, which contradicts the absence of step-like features in Figure 1 at the same temperature. This inconsistency should be addressed—either by reconciling the two datasets or clarifying which is more representative.

In Figure 10B, the identity of the oxidized particles should be confirmed—are they pure NiO or Ni/NiO core–shell structures as observed during vacuum annealing?

In Figure 10D, the authors attribute performance improvement above 650 C to the cessation of oxygen transfer and stabilization of reduced Ni. However, no direct evidence is provided that Ni remains reduced at 700 C. Additional structural or spectroscopic analysis is needed to substantiate this claim.

The statement that NiO dissolves and reincorporates into the lattice at 800–900 C appears to contradict thermodynamic expectations. What mechanism or driving force could explain this behavior?

Lastly, the surface smoothing observed in Figures 5 and 10 raises the question of whether La2NiO4 has reverted to LaNiO3. Has the parent phase undergone further transformation beyond Ni dissolution? If so, the mechanism of this structural recovery should be explored in more detail.

Version 1:

Reviewer comments:

Reviewer #1

(Remarks to the Author)
I thank the authors for their constructive responses to my previous comments. The clarity and significance of the manuscript has been improved. I still have several questions for clarification. Once these issues are satisfactorily addressed, I believe the manuscript would be suitable for publication in Nature Communications.

Major Comments

1. As emphasized in my first-round review, establishing a clear connection between in-situ TEM observations and realistic catalytic conditions is essential for demonstrating the broader relevance of this study. The authors are therefore encouraged to explicitly specify the exact experimental conditions for each in-situ experiment directly (gas pressure, gas composition and flow conditions, heating rate, temperature holding time etc.) in the main figures or text, rather than indicating only the type of gas atmosphere. Providing this information in a transparent and systematic manner is for enabling comparison with practical DRM conditions.
2. The authors' effort to bridge the pressure gap through a combination of in-situ TEM, in-situ XPS, and intermediate-pressure DRM experiments is important. The manuscript argues that NiO formation plays a crucial role during the DRM process. In this context, it is essential to clarify whether NiO can still exist as a relevant intermediate phase under realistic catalytic conditions.
3. Related to the previous point, the lifetime of intermediate phases represents a key aspect that must be clarified when relating low-pressure in-situ observations to real catalytic operation. Under in-situ environment, the weaker reducing environment may stabilize intermediate states such as NiO or La2NiO4, allowing them to be directly observed. In contrast, under practical DRM conditions with much higher reactant partial pressures, the reducing potential of the gas mixture is significantly enhanced. This raises the question of whether these intermediate states are rapidly bypassed and therefore have a negligible influence on catalytic performance, or whether they persist long enough to affect activity, selectivity, or stability. Clarifying this issue would substantially enhance the mechanistic relevance and overall impact of the manuscript.

Minor Comments
4. Ni is known to be highly susceptible to oxidation. Even under high-vacuum conditions (~10^{-6} Pa), adsorbed oxygen species on inert substrates such as SiN_x can oxidize nanoscale Ni particles. The authors should therefore more rigorously exclude alternative oxygen sources or, ideally, provide direct evidence for lattice oxygen participation in the observed Ni oxidation processes.
5. For clarity and consistency, the pressure units used in the Supporting Information should be unified with those in the main text, preferably using Pa.
6. The manuscript would benefit from a more detailed discussion of how the observed dynamic structural behavior impacts DRM performance. At present, this aspect is addressed only briefly in the conclusion (e.g., "As this highly dynamic behaviour clearly occurs at temperatures, where DRM is usually carried out, it is expected to have a high impact on DRM operation."). The authors are encouraged to expand this discussion and more explicitly connect the observed structural dynamics to catalytic activity, stability, and reaction pathways in DRM.

Reviewer #2

(Remarks to the Author)
The authors have addressed all the points raised. I support publication.

Reviewer #3

(Remarks to the Author)
The authors have conducted novel TEM analyses under a DRM atmosphere, and the high-quality images effectively reveal structural changes in the material. However, despite the exceptional quality of the analyses, the derived results show limited significance in explaining catalytic properties, and the authors have not achieved their intended research objectives. To strengthen this work, the authors should either (1) modify the TEM results to directly explain catalytic characteristics, (2) support the TEM observations with complementary analytical techniques, or (3) revise the overall direction of the paper.

1. The in situ TEM analysis under DRM atmosphere in Figure 8 shows a significant gap with the actual catalytic activity trends and is inadequate to explain catalyst behavior. The catalytic activation observed at 650oC is not evident in the in-situ TEM results. While the authors attribute this to the reduction of oxidized Ni near 650oC, independent verification is necessary. The authors must provide ex situ post-reaction analyses or other complementary characterizations to directly demonstrate that Ni reduction occurs.

2. The authors argue, based on Figure 8, that Ni formed via H2 treatment at 700oC oxidizes to NiO within seconds under DRM conditions at 600oC, attributing this oxidation to CO2 dissociation. Given the authors' assertion that no coke formation occurs, any CO2 dissociation event must stoichiometrically yield CO gas as a byproduct. Since the authors explicitly attribute the onset of DRM activity at 650oC to the subsequent reduction of NiO to metallic Ni, the CO gas generated from the initial CO2-driven oxidation should logically be detected prior to the catalytic activation. However, Figure S12 fails to exhibit any such CO evolution trend preceding the activity onset. The absence of this pre-activation CO signal directly contradicts the hypothesis that CO2 dissociation is the oxygen source for the rapid Ni oxidation observed at 600oC.

3. The preliminary experiments conducted under H_2 and CH4/CO2 atmospheres (Figure 3) correlate poorly with the subsequent DRM results (Figure 8). In Figure 3, the parent La2NiO4 phase with a stepped surface is maintained even at 800oC. However, in Figure 8, surface smoothing occurs at only 700oC under DRM conditions. One would expect more pronounced dissolution at the higher temperature, so this apparent contradiction requires clarification.

4. The discussion of structural changes identified in the TEM analysis needs to be strengthened. The results section primarily presents observations, and the discussion relies heavily on references rather than providing original mechanistic insights. While the study is TEM-focused, a more detailed analysis of the observed phenomena would help readers better understand the underlying mechanisms. In particular, the mechanism by which Ni particles undergo dissolution in the H_2

atmosphere at 700oC for 10 minutes should be explained more thoroughly.

5. Additional analyses are required to support and validate the TEM observations:
1) The formation of the La_2NiO_4 phase should be discussed in greater detail (Figure 3). To precisely identify the temperature at which the phase transition occurs, the authors should conduct additional TEM analyses at intermediate temperatures (e.g., 600oC) to demonstrate the transition from the $LaNiO_3$ phase to the La_2NiO_4 phase, providing more concrete evidence of the temperature-dependent phase evolution.

2) While faceted NiO particles appear to play a critical role in catalytic behavior, the evidence that these particles are indeed NiO is insufficient (Figure 8). Additionally, the mechanism by which the stepped surface disappears at elevated temperatures must be clarified; specifically, whether this represents a phase transition to $LaNiO_3$ or another structural transformation.

Version 2:

Reviewer comments:

Reviewer #1

(Remarks to the Author)
I appreciate the authors' substantial efforts in revising the manuscript and in providing additional experimental evidence, including operando/in situ XPS, PXRD, and quenching experiments under DRM conditions. The revised manuscript is considerably improved, and the added sections help to connect the in situ microscopy observations with catalytic DRM performance. Due to numerous changes, the manuscript needs further refinement, I therefore recommend that the manuscript undergo minor revision before acceptance.

Some Editorial Issues
The manuscript contains numerous typographical and grammatical errors. Examples include:
“obserbvations” “formaiton” “ftting”“atomically-resloved” “amd” “accompaying”“showin” “lattic” “strenghtens” “esssentially” “stipuletd priciples” “isNiO” “over the the 3D perovskite” et. al.

Reviewer #3

(Remarks to the Author)
The authors have made substantial improvements to the manuscript by incorporating additional in-situ characterizations, including AP-XPS and in-situ XRD, which significantly strengthen the mechanistic interpretation beyond the previous version that relied heavily on in-situ TEM. The systematic comparison under multiple atmospheres and temperature conditions provides useful insights into the dynamic surface evolution of the exsolution catalyst under practically relevant reaction environments. In addition, the inclusion of fixed-bed catalytic reaction results and in-situ XRD measurements under conditions closer to realistic operation effectively addresses concerns regarding the gap between model in-situ analyses and practical catalytic conditions.

Overall, the manuscript has been considerably improved, and most of the major concerns raised previously have been adequately addressed. I believe the work is now suitable for publication in Nature Communications.

One remaining suggestion is related to the presentation of the manuscript. The current version has become quite lengthy, and in several sections the manuscript attempts to describe nearly all possible information obtained from the measurements, which occasionally interrupts the logical flow of the discussion. I encourage the authors to further streamline the text and focus more concisely on the key findings directly relevant to the central mechanism and conclusions.

Open Access This Peer Review File is licensed under a Creative Commons Attribution 4.0 International License, which permits use, sharing, adaptation, distribution and reproduction in any medium or format, as long as you give appropriate credit to the original author(s) and the source, provide a link to the Creative Commons license, and indicate if changes were made.
In cases where reviewers are anonymous, credit should be given to 'Anonymous Referee' and the source.
The images or other third party material in this Peer Review File are included in the article's Creative Commons license, unless indicated otherwise in a credit line to the material. If material is not included in the article's Creative Commons license and your intended use is not permitted by statutory regulation or exceeds the permitted use, you will need to obtain permission directly from the copyright holder.
To view a copy of this license, visit https://creativecommons.org/licenses/by/4.0/

NCOMMS-25-39069, Point-by-point response to the reviewers' comments

Reviewer #1

In this manuscript, Cao et al. focuses on the structural evolution behavior of $LaNiO_3$ perovskite catalyst during DRM reaction. Through in-situ TEM characterization, the study elucidates the structural transformations of $LaNiO_3$, nickel exsolution phenomena, and associated kinetic processes under various gaseous environments, providing crucial insights into the complex behavior of perovskite catalysts under realistic reaction conditions. However, the main points of the manuscript are excessively dispersed, and the scientific significance requires further refinement and consolidation. Some suggestions for improvement are listed below.

1, The manuscript presents an excess of experimental details, which, at times, prove to be a source of distraction and detraction from the clarity of the central scientific arguments. In order to facilitate the identification of the key findings and mechanistic insights, it is necessary to present the material in a more concise and focused manner. Moreover, the narrative would benefit from enhanced logical coherence between sections, as the current text structure tends to list results without sufficiently integrating them into a cohesive storyline.

We would like to express our gratitude to the reviewer for their insightful commentary. In order to enhance the clarity and coherence of the narrative, we have made substantial revisions to the manuscript, focusing on the identification of key findings. We have relocated sections that are peripheral to the primary narrative to the SI, such as the experiments conducted in vacuum. Specifically, we have undertaken a comprehensive review of the Introduction, Results and Discussion sections to ensure the articulation of central scientific arguments and the overarching objective: the decoupling of reduction-induced and DRM-specific structural and morphological changes. Given the extent of the revisions made to the text, we kindly direct readers to refer to the highlighted sections within the manuscript.

2, The in-situ TEM experimental pressure significantly differs from the actual DRM operational pressure; It is essential to elucidate how the conclusions drawn from these in-situ experiments accurately reflect the phenomena occurring during the catalytic process.

Indeed, environmental (S)TEM is limited to very low pressures, in our case to 1.5 Pa. This results in a large pressure gap compared to the conditions under which real catalytic processes take place. As a result, the conclusions drawn from our experiments may not be directly transferable. To address the pressure gap, we have accordingly added catalytic DRM data as measured in our fixed-bed flow reactor (500 Pa CO_2 and CH_4 each, He added to 10^5 Pa total pressure, Figure R1) as formation of H_2 and CO, as well as H_2/CO

ratio, including a short-time stability test over 30 min to the SI of the revised version (SI: Figure S12). The experimental conditions were chosen as closely as possible to the in-situ STEM experiments. However, lower partial reactant pressures are not possible in the flow reactor system. Qualitatively, the data are similar to the tests at higher reactant partial pressure and complementary in situ XRD tests under comparable conditions in the DRM mixture show complete decomposition of $LaNiO_3$ into La_2O_3 + Ni with intermediate La_2NiO_4 formation – as described previously in: *Bonmassar, N. et al. In Situ-Determined Catalytically Active State of LaNiO3 in Methane Dry Reforming; ACS Catal. 2020, 10, 1102–1112. doi: 10.1021/acscatal.9b03687.*

As it is evident:

(1), $LaNiO_3$ can be directly activated under these catalytic DRM conditions, as judged from the H_2 and CO evolution. This behaviour is qualitatively similar to the 10^5 Pa DRM measurements as discussed in *Bonmassar, N. et al. In Situ-Determined Catalytically Active State of $LaNiO_3$ in Methane Dry Reforming; ACS Catal. 2020, 10, 1102–1112. doi: 10.1021/acscatal.9b03687*)

(2), Structure-wise (see in situ DRM PXRD measurements under similar conditions, Figure R2 (SI: Figure 13) and R3 (SI: Figure 14)), the sequence of $LaNiO_3$ → La_2NiO_4 + Ni → La_2O_3 + Ni is comparable to that under 1 bar flow conditions (see ref. *Bonmassar, N. et al. In Situ-Determined Catalytically Active State of LaNiO3 in Methane Dry Reforming; ACS Catal. 2020, 10, 1102–1112. doi: 10.1021/acscatal.9b03687*).

(3), Once $LaNiO_3$ is decomposed into Ni+La_2O_3, the light off temperatures shift to lower temperatures, as $LaNiO_3$ is already activated in the second DRM cycle.

(4), True DRM activity is measured in both scenarios, as judged from the H_2/CO ratios.

(5), Quantitative comparison is not straightforward, as for the 500 Pa CO_2 + 500 Pa CH_4 DRM mixture, we are already approaching the detection limit of the mass spectrometer. That means, the conversion as given by 65% is the lower limit and the true conversion is supposedly much higher.

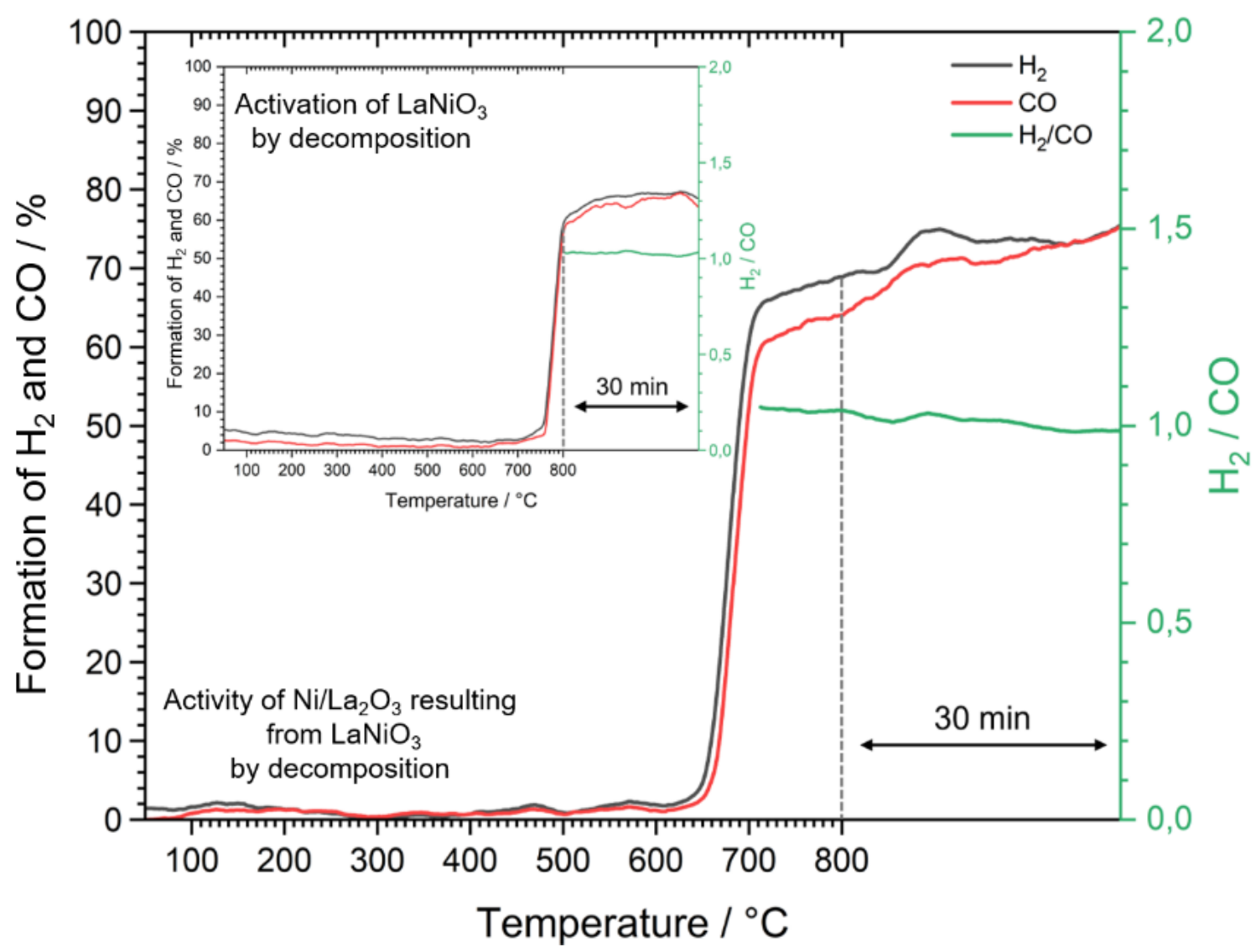

Figure R1 (SI: Figure S12): Small Panel: Catalytic DRM profiles during $LaNiO_3$ decomposition in a CO_2:CH_4 = 1:1 mixture (500 Pa each, He added to 10^5 Pa pressure). Large Panel: Catalytic DRM profiles in a CO_2:CH_4 = 1:1 mixture (500 Pa each, He added to 10^5 Pa pressure) on Ni-La_2O_3 resulting from $LaNiO_3$ decomposition. Heating rate: 5 °C min^{-1}, 200 mg catalyst.

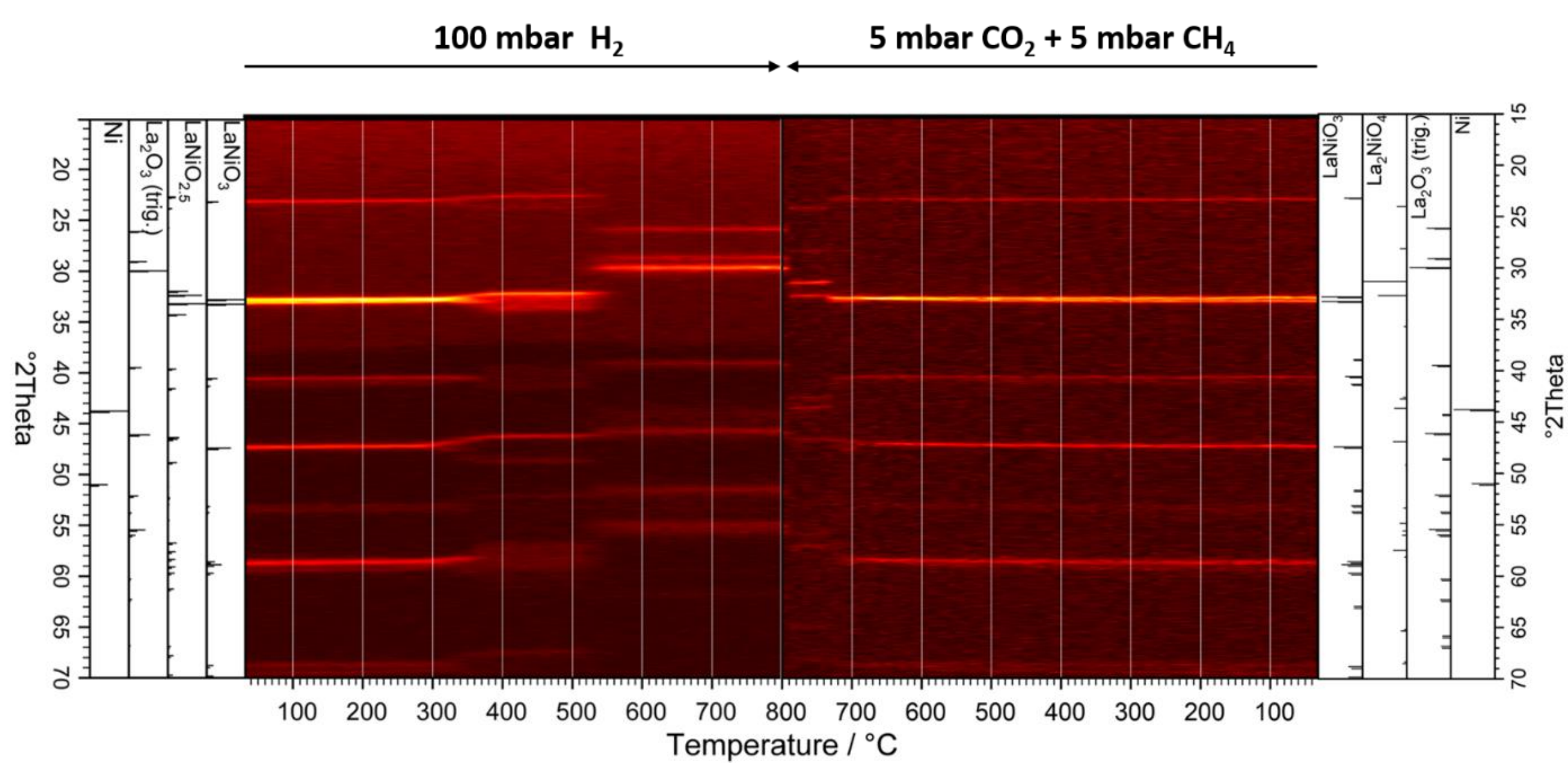

Figure R2 (SI: Figure S13): In situ PXRD experiments in 100 mbar pure H_2 as compared to a 500 Pa (5 mbar) CO_2 + 500 Pa (5 mbar) CH_4 mixture.

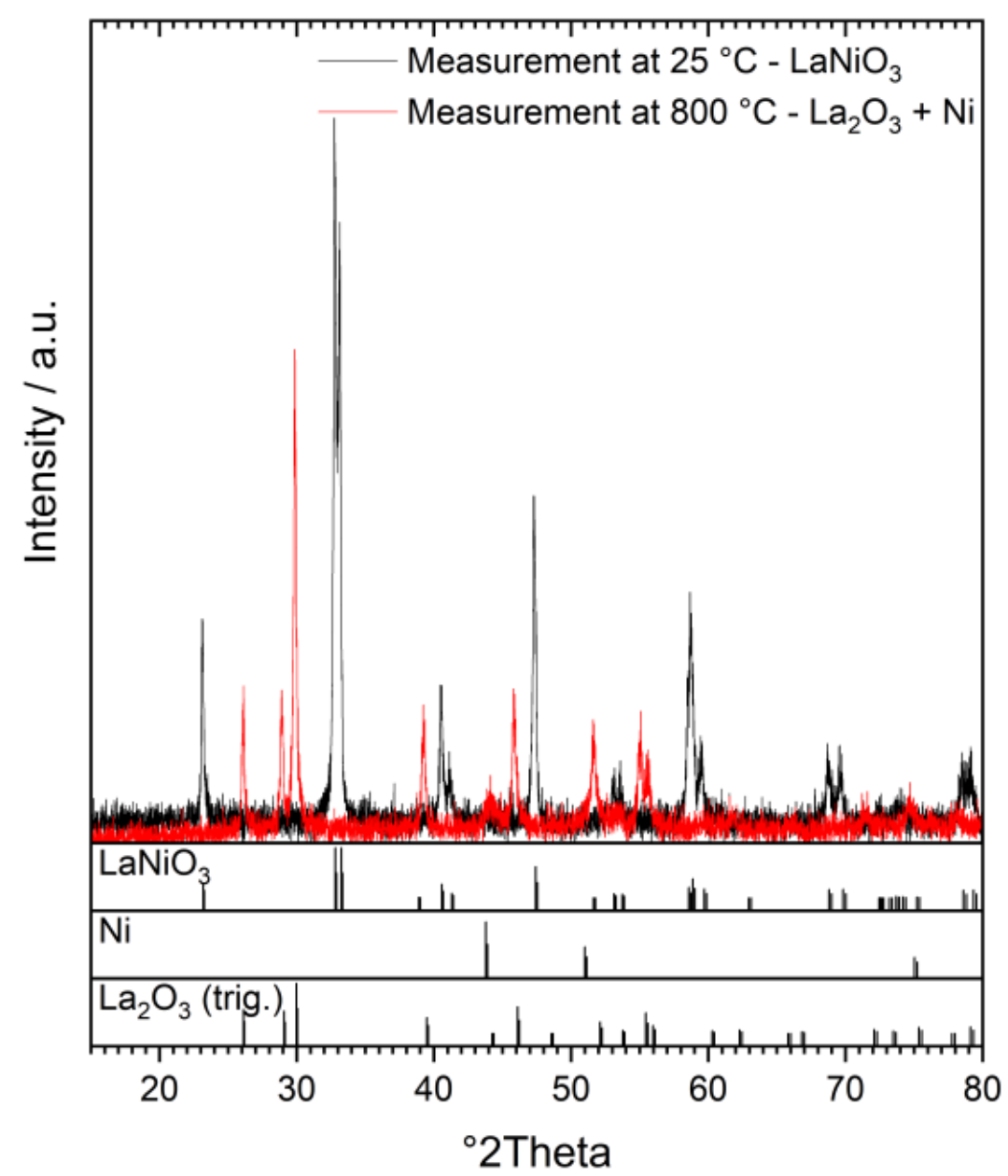

Figure R3 (SI: Figure S14): Direct comparison of the PXRD patterns in situ collected at 25 °C and 800 °C (taken from Figure R2) to verify the $LaNiO_3$ decomposition in a 500 Pa (5 mbar) CO_2 + 500 Pa (5 mbar) CH_4 mixture, which is consistent with: *Bonmassar, N. et al. In Situ-Determined Catalytically Active State of $LaNiO_3$ in Methane Dry Reforming; ACS Catal. 2020, 10, 1102–1112. doi: 10.1021/acscatal.9b03687*)

The corresponding additional data was added as supplementary information: Figure S12, Figure S13 and Figure S14.

3, The author puts forward the hypothesis that the particle coarsening observed in a hydrogen atmosphere above 550°C is attributable to Ostwald ripening. Nevertheless, the potential for additional nickel precipitation due to elevated temperatures has not been sufficiently excluded. It is therefore supplementary in-situ experiments with high spatial and temporal spatial resolution are needed.

We agree that Ostwald ripening (OR) and exsolution/precipitation may contribute simultaneously to particle coarsening in a hydrogen atmosphere above 550 °C. Our evidence for OR is based on the real-time observation that smaller particles shrink and disappear (particles 4-13 in Figure 7, Movie S1) while larger particles grow (particles 1 and 2 in Figure 7) above 550°C. Similar results are shown in Figure S18, which further supports the presence of an OR process. Concurrently, La_2NiO_4 formation is observed via atomic-resolved SE and DF images (Figure S17), indicating ongoing decomposition and exsolution. Since the phenomenon is a dynamic process showing both particle dissolution and particle growth simultaneously,

and is confirmed by real-time movies (Movie S1 and S2), the interpretation of Ostwald ripening alongside continuous Ni exsolution is robustly based on the *in-situ* high spatial and temporal resolution data already provided in Figure 7 and the Supporting Information (Movies S1, S2, Figure S17, S18). We have rephrased the manuscript accordingly in section 3.2:

"multiple concurrent mechanisms, including continuous Ni exsolution along phase transitions from $LaNiO_3$ to La_2NiO_4, OR processes, and particle migration."

In addition, to account for recrystallization processes, we have rephrased the manuscript with respect to the exsolution/formation of Ni particles in section 3.1:

"To address a critical conceptual gap of Ni particle formation during (partial) $LaNiO_3$ decomposition, we note that we in fact have to discriminate between true exsolution and solid-state recrystallization effects. While genuine exsolution often employs non-stoichiometric perovskite formulations to trigger Ni exsolution along established crystallographic alignment relationships and maintaining the original parent perovskite host lattice, recrystallization resulting in complete lattice restructuring thereby creating new socket sites with altered anchoring and interfacial properties.[39, 40] : While the latter is certainly true at more progressed states of $LaNiO_3$ decomposition, where La_2NiO_4 starts to form (alongside simultaneous Ni particle formation) and then finally collapses structurally to form La_2O_3 and Ni, we have recently shown[41], that in the early stages of hydrogen reduction, where no La_2NiO_4 is formed and the redox chemistry is essentially dominated by oxygen removal to form bulk oxygen-deficient $LaNiO_{2.7}$ and $LaNiO_{2.5}$, true exsolution of Ni occurs and surface-bound Ni particles are formed. Having said that, the type of Ni particle formation observed here is more likely associated with bulk recrystallization."

4, On page 12, line 227, the author asserts that particles precipitate within the substrate; however, neither STEM SE images nor DF images can definitively determine whether particles are located on the surface, within the interior, or on the reverse side of the substrate. The description associated with Figure 8B exhibits the same issue.

Yes, we agree that particles on the upper surface can be imaged, while the particles inside, or on the reverse side of the substrate cannot be detected by the SE detector. For vacuum heating, the SE images provided information projected along the viewing direction but did not yield distinct surface topographic signals indicative of individual Ni particles. Therefore, we could not definitively assign the resolved particles to the upper surface. We have rewritten the text to make this clearer. We now write in the SI, Section E:

"Although we cannot distinguish whether Ni-rich particles are located within the interior or on the reverse side of the substrate, this behaviour contrasts the Ni particles formed in H_2, where the exsolved Ni particles already predominantly form on the surface of the parent perovskite."

5, The vacuum heating subsection is not directly related to the catalytic reaction and does not contribute to the main arguments of the manuscript; therefore, it is unnecessary to include this discussion in the main text.

We think that the vacuum heating experiments are still meaningful, in order to decouple thermal, purely reduction-induced, and DRM-triggered structural and morphological changes. However, in order to present our results in a more concise and focused manner, we have moved the section on vacuum heating experiments to the Supplementary Information as the reviewer suggested.

6, The authors suggest that Figure 9 represents the OA process; however, particles 1, 2, and 6 do not exhibit significant size changes between 550 and 650°C. The determination of whether the disappearance of particles is not attributable to new structural evolution induced by temperature elevation remains unclear.

In order to explore particle evolution quantitively, we have added a particle size analysis to compare the volume of Ni particles at 500 °C and 650 °C. The volume of the exsolved Ni particles was estimated by assuming spherical particle shapes. We measured the diameters of particles 1 - 13 and calculated their volumes (using the equation $V = (4/3)\pi r^3$). The results show that the combined volume of particles 1, 2, and 6 at 650 °C is 210.88 nm^3, which is greater than their combined volume at 500 °C. This provides evidence for the dissolution of particles 3, 4, 5, and 7-13, and the subsequent migration and incorporation of their mass into particles 1 and 2 via mass transfer. The observed volume difference may also be attributed to Ni atoms originating from other smaller particles outside the field of view.

Table R1 (SI: Table S2): Quantitative analysis of Ni particles at 500 °C and 650 °C.

500 °C			650 °C		
No.	Diameter (nm)	Volume (nm^3)	No.	Diameter (nm)	Volume (nm^3)
1	4.8	56.8	1	6.3	132.8
2	2.9	12.9	2	5.0	65.8
3	2.8	11.4	3	0	0
4	2.5	8.5	4	0	0
5	2.7	10.1	5	0	0

6	2.9	12.8	6	2.9	12.2
7	2.5	8.1	7	0	0
8	2.0	4.0	8	0	0
9	2.7	10.2	9	0	0
10	2.2	5.2	10	0	0
11	1.0	0.6	11	0	0
12	2.4	6.9	12	0	0
13	2.1	4.6	13	0	0
Sum		152.1			210.8

We have added the data of our quantitative analysis and rephrased the text accordingly in section 3.2:

"We have performed a quantitative analysis of the particle size to estimate the volume of Ni particles at 500 °C and 650 °C (Table S2). The analysis shows that the combined volume of particles 1, 2, and 6 at 650 °C is 210.8 nm^3, which is greater than their combined volume at 500 °C. This indicates the dissolution of particles 3, 4, 5, and 7-13 and a mass transfer into particles 1 and 2. This behaviour can be assigned to an annealing-driven Ostwald ripening (OR) process, where smaller particles dissolve, diffuse, and subsequently merge into larger ones.[46] The observed increase in volume is likely attributed to additional nickel particle precipitation/formation."

Reviewer #2:

Well-designed study with sophisticated in-situ techniques and clear research objectives. The work addresses an important gap in understanding perovskite catalyst behavior. The microscopy convincingly captures qualitative pathways (Ni exsolution, $LaNiO_3 \rightarrow La_2NiO_4$) but, in its current form, the study does not yet provide sufficient quantitative or catalytic evidence to support the broader claims about DRM activity and industrial relevance. Addressing the points above would greatly enhance the manuscript's rigor and impact.

Introduction

A critical conceptual gap in this work is the inadequately distinguish between solid-state recrystallization and true exsolution, which are fundamentally different processes with distinct mechanistic and practical implications. What the authors describe as "exsolution" is actually solid-state recrystallization occurring in their stoichiometric $LaNiO_3$ starting material, which results in complete lattice restructuring to form the La_2NiO_4 Ruddlesden-Popper phase and creates new "socket" sites with different anchoring properties. This process allows for potential reversibility under certain conditions and represents a phase transformation rather than true exsolution. In contrast, genuine exsolution involves non-stoichiometric perovskites (A-site deficient) that form nanoparticles while maintaining the same residual host lattice structure, resulting in minimal structural reorganization, potentially greater long-term stability, but typically irreversible behavior due to the maintained lattice framework. This distinction is particularly important when evaluating the authors' claim on page 4 that "hydrogen treatments at elevated temperatures induced a total collapse of the perovskite structure, triggering the formation of Ni in contact with La_2O_3" - this statement is not generally applicable to all perovskite systems but is specifically characteristic of stoichiometric starting materials undergoing recrystallization. Since most recent exsolved catalysts often employ non-stoichiometric compositions designed for true exsolution, the observed recrystallization behavior may be less relevant than claimed, and the authors should qualify their conclusions about general "perovskite behavior" accordingly while acknowledging that their mechanistic insights apply specifically to stoichiometric recrystallization processes.

We thank the reviewer pointed this out, we have recently revealed (*Malleier et al., In situ Impedance spectroscopy as a marker method during $LaNiO_3$ decomposition, J. Amer. Chem. Soc, 2025*), that upon hydrogen reduction $LaNiO_3$ is reduced to $LaNiO_{2.5}$, which still bears an X-ray diffractogram close to that of $LaNiO_3$. $LaNiO_{2.5}$ formation is a result of oxygen loss from $LaNiO_3$ and, as revealed by in situ X-ray photoelectron spectroscopy at least from 280 °C on is accompanied by Ni exsolution on the surface. At 400 °C, where no La_2NiO_4 is present, but still only bulk $LaNiO_{2.5}$, Ni exsolution is visible using TEM. In essence, this is a clear exsolution process. We agree that once La_2NiO_4 starts to form, Ni formation accelerates and the energetic situation at the interface is different and might be overlaid by, as it is called, a recrystallization process. We have amended the revised version accordingly in section 3.1:

"To address a critical conceptual gap of Ni particle formation during (partial) $LaNiO_3$ decomposition, we note that we in fact have to discriminate between true exsolution and solid-state recrystallization effects. While genuine exsolution often employs non-stoichiometric perovskite

formulations to trigger Ni exsolution along established crystallographic alignment relationships and maintaining the original parent perovskite host lattice, recrystallization resulting in complete lattice restructuring thereby creating new socket sites with altered anchoring and interfacial properties.[39, 40] : While the latter is certainly true at more progressed states of $LaNiO_3$ decomposition, where La_2NiO_4 starts to form (alongside simultaneous Ni particle formation) and then finally collapses structurally to form La_2O_3 and Ni, we have recently shown[41], that in the early stages of hydrogen reduction, where no La_2NiO_4 is formed and the redox chemistry is essentially dominated by oxygen removal to form bulk oxygen-deficient $LaNiO_{2.7}$ and $LaNiO_{2.5}$, true exsolution of Ni occurs and surface-bound Ni particles are formed. Having said that, the type of Ni particle formation observed here is more likely associated with bulk recrystallization."

Good literature survey of exsolution studies, but the section repeatedly cites earlier work to justify catalytic relevance while the present study contributes no new catalytic testing (activity, selectivity, stability).

We agree. To address the pressure gap, we have accordingly added catalytic DRM data as measured in our fixed-bed flow reactor (500 Pa CO_2 and CH_4 each, He added to 10^5 Pa total pressure, Figure R1) as formation of H_2 and CO, as well as H_2/CO ratio, including a short-time stability test over 30 min to the SI of the revised version (SI: Figure S12). The experimental conditions were chosen as closely as possible to the in-situ TEM tests. However, lower partial reactant pressures are not possible in the flow reactor system. Qualitatively, the data are similar to the tests at higher reactant partial pressure and complementary in situ XRD tests under comparable conditions in the DRM mixture show complete decomposition of $LaNiO_3$ into La_2O_3 + Ni with intermediate La_2NiO_4 formation – as described previously in: *Bonmassar, N. et al. In Situ-Determined Catalytically Active State of LaNiO3 in Methane Dry Reforming; ACS Catal. 2020, 10, 1102–1112. doi: 10.1021/acscatal.9b03687.*

As it is evident:

(1), $LaNiO_3$ can be directly actived under these catalytic DRM conditions, as judged from the H_2 and CO evolution. This behaviour is qualitatively similar to the 10^5 Pa DRM measurements as discussed in *Bonmassar, N. et al. In Situ-Determined Catalytically Active State of $LaNiO_3$ in Methane Dry Reforming; ACS Catal. 2020, 10, 1102–1112. doi: 10.1021/acscatal.9b03687.*

(2), Structure-wise (see in situ DRM PXRD measurements under similar conditions, Figure R2 (SI: Figure S13) and R3 (SI: Figure S14)), the sequence of $LaNiO_3$ → La_2NiO_4 + Ni → La_2O_3 + Ni is similar to that under 1 bar flow conditions (see ref. *Bonmassar, N. et al. In Situ-Determined Catalytically Active State of LaNiO3 in Methane Dry Reforming; ACS Catal. 2020, 10, 1102–1112. doi: 10.1021/acscatal.9b03687*).

(3), Once $LaNiO_3$ is decomposed into Ni+La_2O_3, the light off temperatures shift to lower temperatures, as $LaNiO_3$ is already activated in the second DRM cycle.

(4), True DRM activity is measured in both scenarios, as judged from the H_2/CO ratios.

(5), Quantitative comparison is not straightforward, as for the 5 mbar CO_2 + 5 mbar CH_4 DRM mixture, we are already approaching the detection limit of the mass spectrometer. That means, the conversion as given by 65% is the lower limit and the true conversion is supposedly much higher.

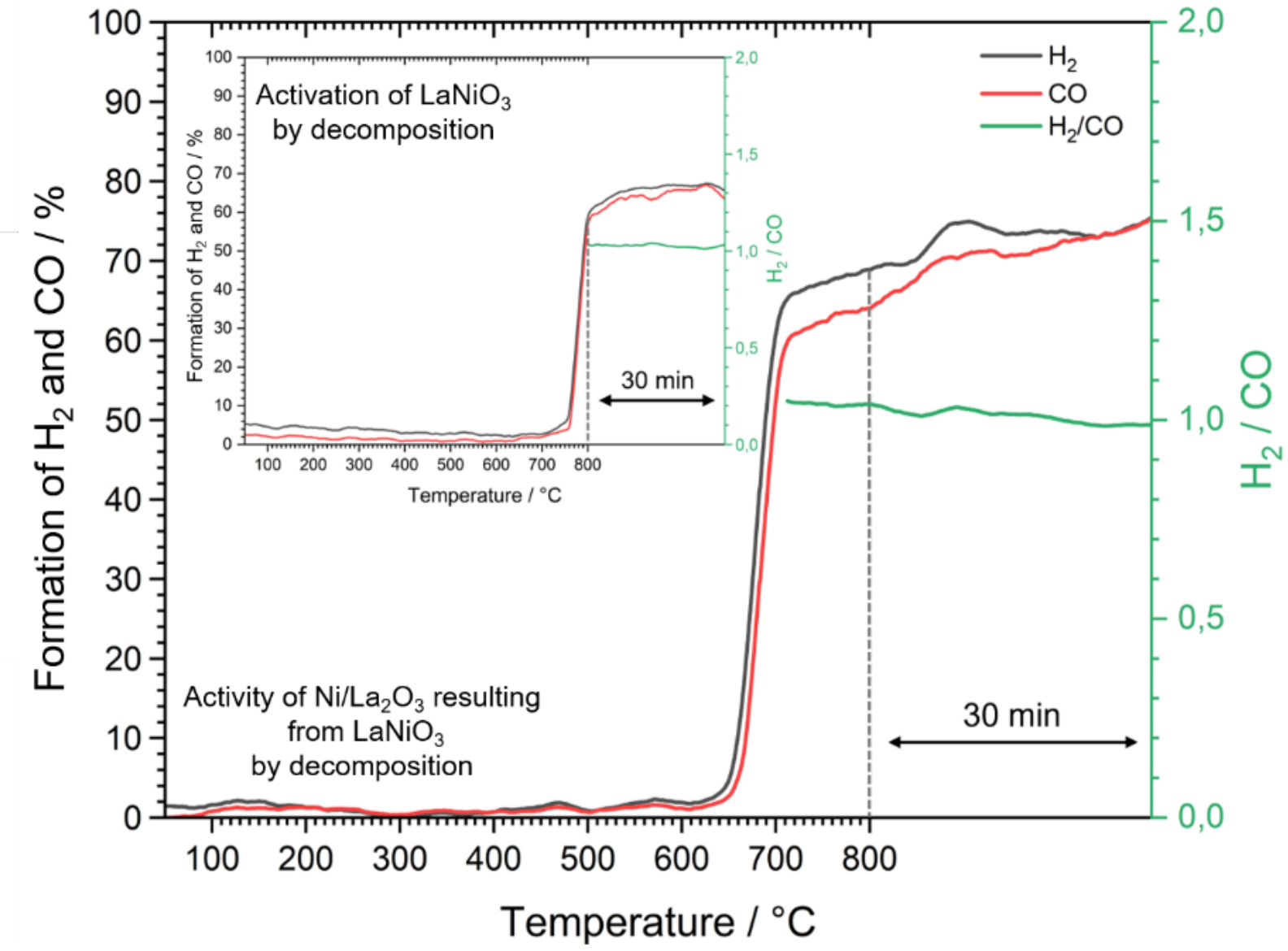

Figure R1 (SI: Figure S12): Small Panel: Catalytic DRM profiles during $LaNiO_3$ decomposition in a CO_2:CH_4 = 1:1 mixture (500 Pa each, He added to 10^5 Pa pressure). Large Panel: Catalytic DRM profiles in a CO_2:CH_4 = 1:1 mixture (500 Pa each, He added to 10^5 Pa pressure) on Ni-La_2O_3 resulting from $LaNiO_3$ decomposition. Heating rate: 5 °C min^{-1}, 200 mg catalyst.

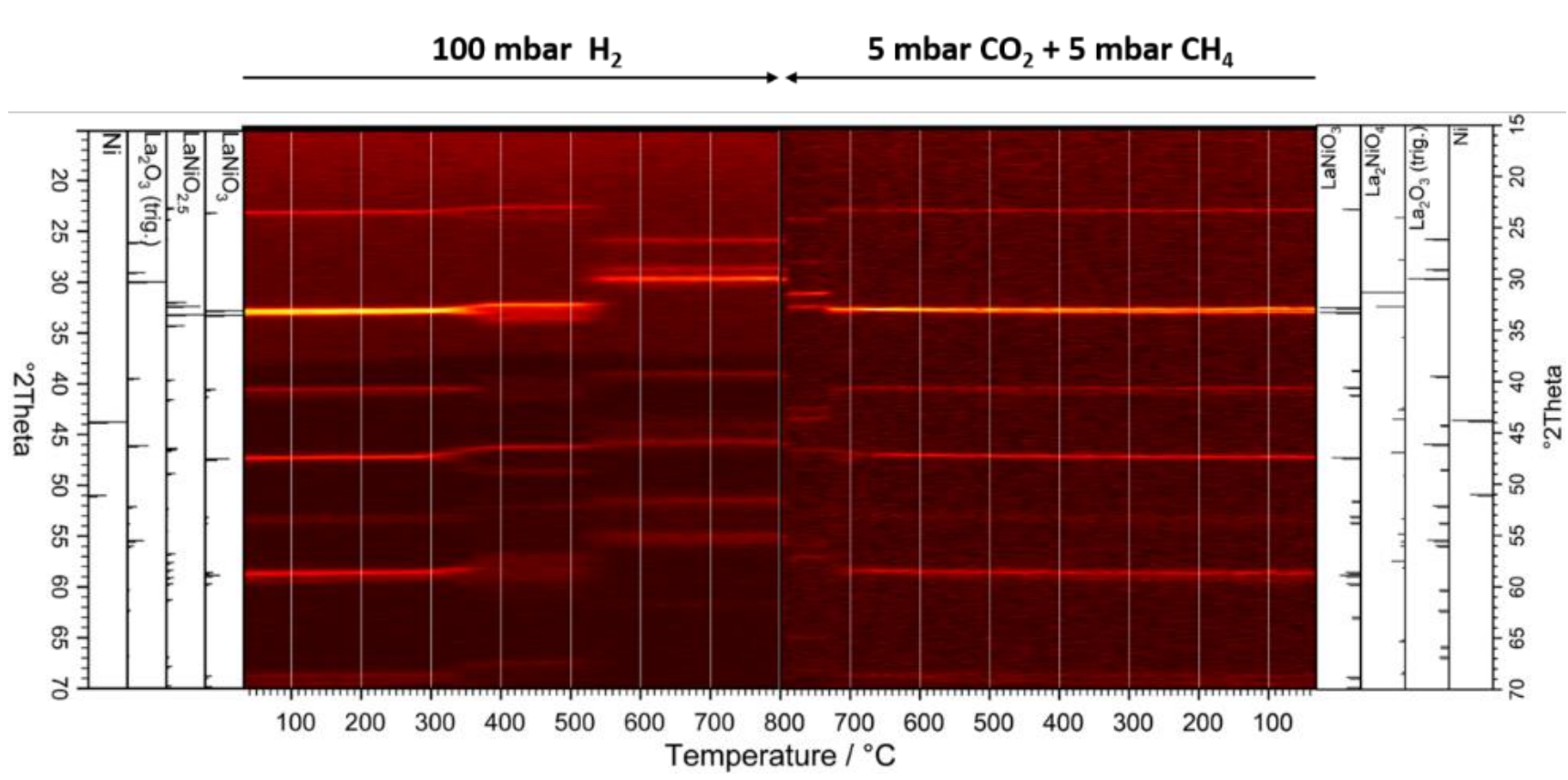

Figure R2 (SI: Figure S13): In situ PXRD experiments in 100 mbar pure H_2 as compared to a 500 Pa (5 mbar) CO_2 + 500 Pa (5 mbar) CH_4 mixture.

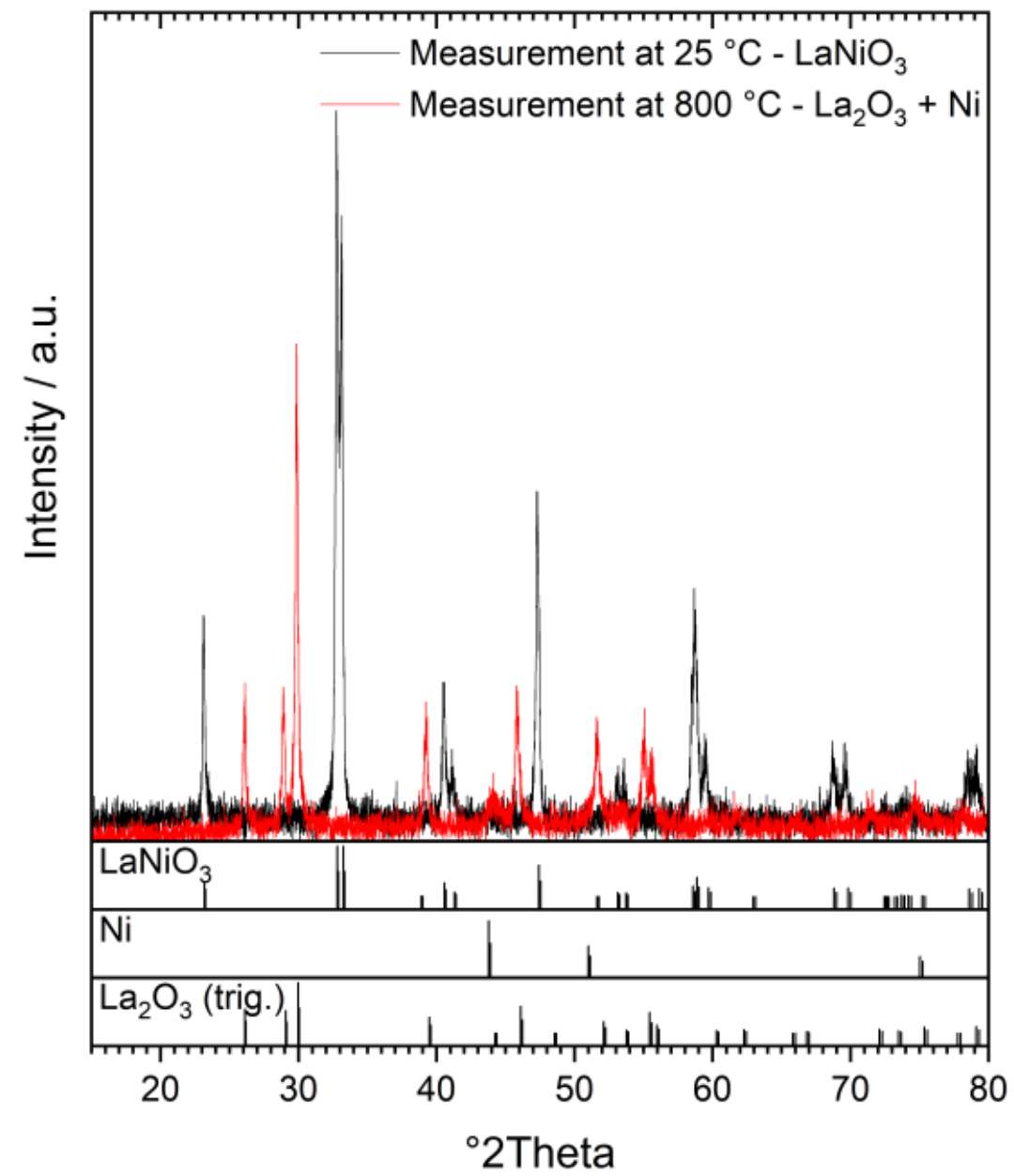

Figure R3 (SI: Figure S14): Direct comparison of the PXRD patterns in situ collected at 25 °C and 800 °C (taken from Figure R2) to verify the $LaNiO_3$ decomposition in a 500 Pa (5 mbar) CO_2 + 500 Pa (5 mbar) CH_4 mixture, which is consistent with: *Bonmassar, N. et al. In Situ-Determined Catalytically Active State of $LaNiO_3$ in Methane Dry Reforming; ACS Catal. 2020, 10, 1102–1112. doi: 10.1021/acscatal.9b03687*)

The additional data was added as supplementary information: : Figure S12, Figure S13 and Figure S14.

$LaNiO_3$ synthesis – potential reproducibility issues No particle-size or surface-area data for the as-prepared powder; these affect reduction kinetics.

We have added a statistical analysis of the particle size to the Supporting Information. The particle size of about 200 particles was measured and is mainly distributed between 20 and 40 nm (Figure R4 (SI: Figure S3)). In addition, we have added BET-measured specific surface area data and provide the pore size

distribution. (Figure R5 (SI: Figure S2)). The BET-determined area is 4.6 $m^2\ g^{-1}$. These Figures have been added to the SI.

While reduction kinetics is affected by the particle size and surface area, our study focuses on the relative comparison between reaction atmospheres (H_2 vs. Vacuum vs. DRM) using the same sample batch. This approach allows for robust mechanistic conclusions.

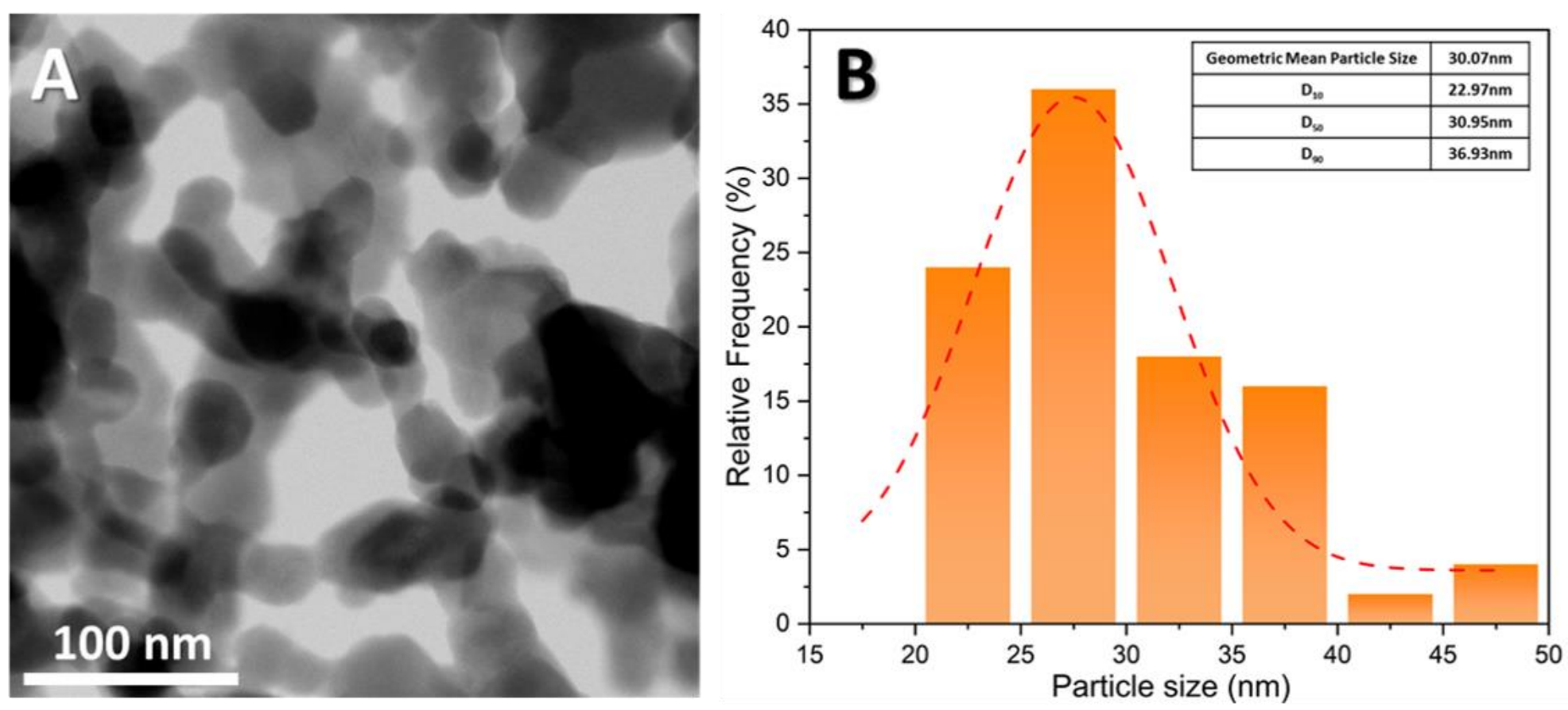

Figure R4 (SI: Figure S3): Representative TEM image of as prepared $LaNiO_3$ and the statistical analysis of particle size.

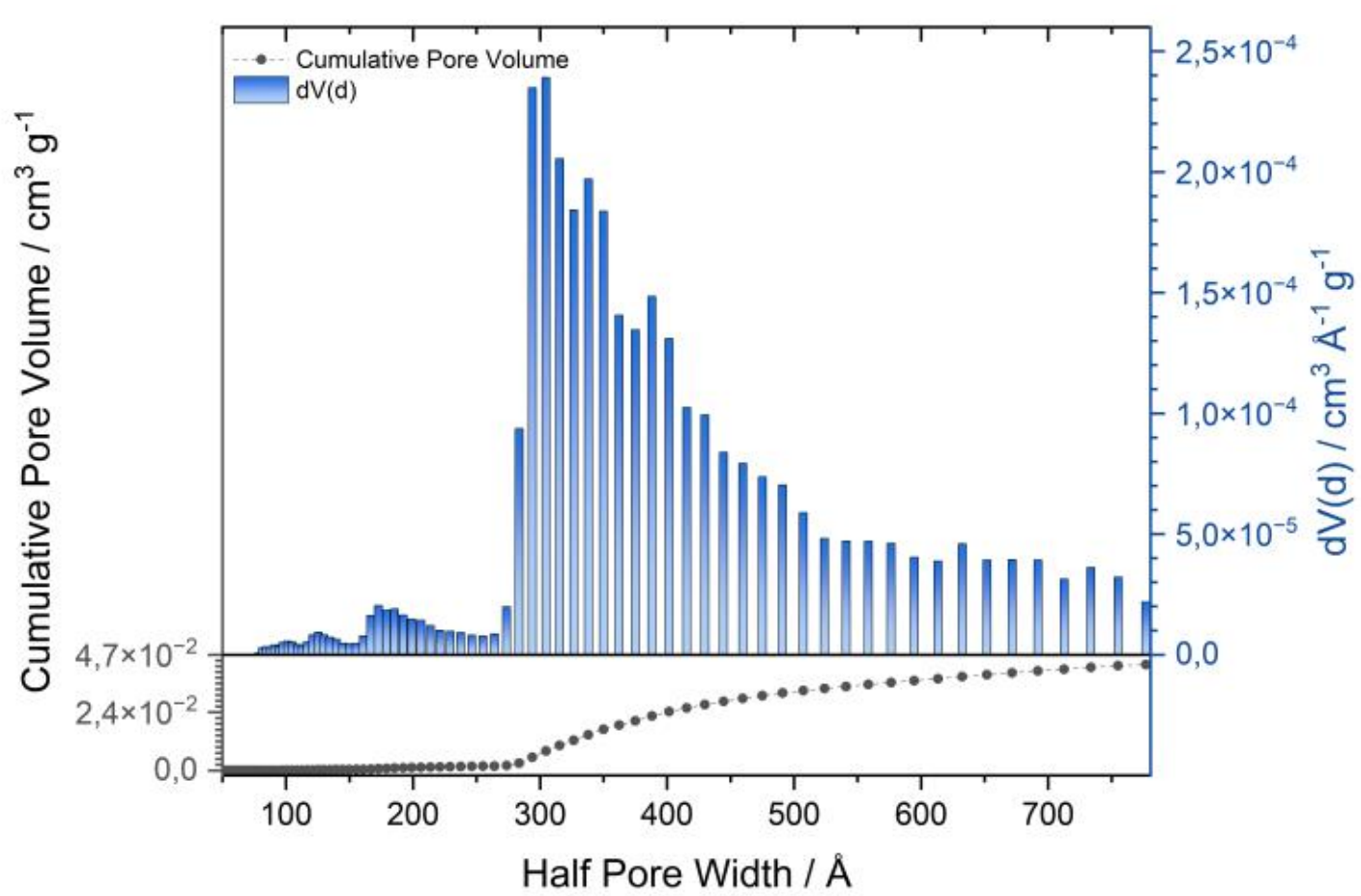

Figure R5 (SI: Figure S2): Cumulative pore volume, as well as pore size distribution for the $LaNiO_3$ sample of this work.

We have added these results in Supporting Information Figures S2 and S3, along with the corresponding description of the sample properties in Section 2.1:

"The BET determined specific surface area is 4.6 $m^2\ g^{-1}$ as determined by N_2 adsorption at - 196 °C on an Anton Paar Autosorb 6000 instrument. The respective pore size distribution alongside the cumulative pore

volume is shown in Figure S2 and reveals a maximum of the half pore width at around 300 Å. The particle size of as-prepared $LaNiO_3$ is primarily distributed between 20 and 40 nm (Section C, Figure S3)."

TEM Only a single low-T control (200 °C) is presented to argue that electron-beam irradiation is harmless. No controls at ≥400 °C where reduction clearly accelerates.

Following the reviewer's suggestion, we investigated potential beam effects at higher temperatures and exposed the sample to the electron beam at 300 °C and 400 °C for 10 minutes under vacuum. As shown in Figure R6 (SI: Figure S4), no morphological changes were induced by the electron beam, confirming that beam damage effects at higher temperature are largely negligible, as well. Throughout our in situ TEM experiments, we used a low beam current (approximately 100 – 200 pA) to reduce potential beam effects.

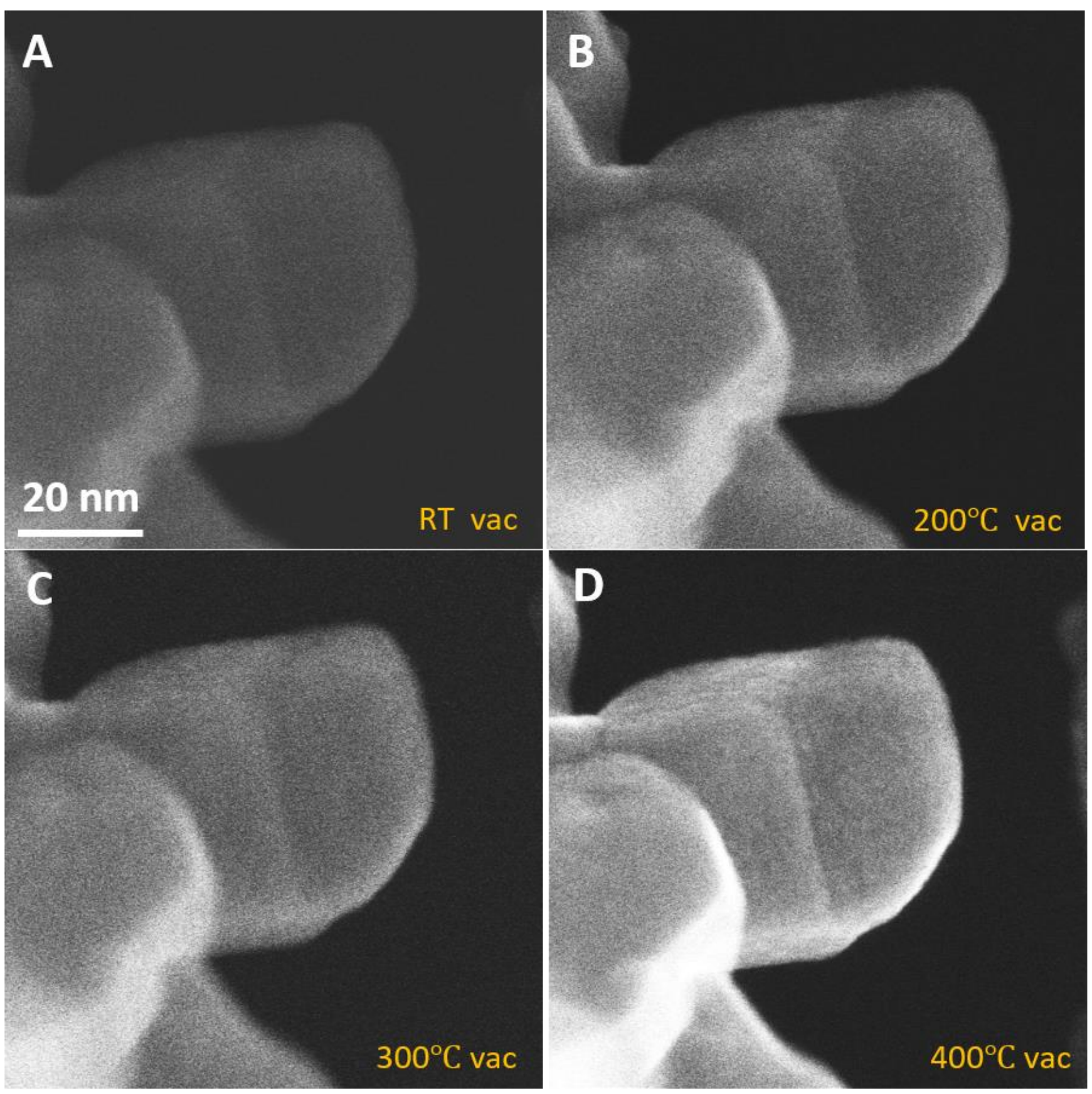

Figure R6 (SI: Figure S4) Electron-beam irradiation analysis under vacuum from room temperature to 400 °C.

We have add this information accordingly in section 2.2:

"To further assess the influence of electron beam irradiation, we exposed the sample to the electron beam at 300°C and 400°C for 10 minutes in vacuo. Morphology changes or damage of the sample due to electron beam were not detected (Section C, Figure S4)."

Heating rates in the MEMS chip, actual temperature calibration, and the number of independent repeats per atmosphere are not stated.

We have added information on the heating rates to the experimental section 2.2. Additionally, information on the MEMS chips, temperature calibration, and number of repeats was added to the SI Figure S25 and Table S3:

"We used a temperature profile where the temperature was continuously increased from 200 °C to 800 ° °C at a heating rate of 10 °C min^{-1}. After each 50 °C increment, the specimen was held for 10 min - 30 min, Images were also collected at 50 °C steps intervals."

"The tip of the MEMS-based heating holder and chip (Norcada Inc., Edmonton, Canada) are shown in Figure R7 (SI: Figure S25). The homogeneously heated region is approximately 50 μm in size and located at the center of heating coil. Temperature calibration is performed by the MEMS-chip manufacturer and a heater temperature accuracy of ±1 °C is specified. We note that the actual temperature can differ from the manufacturer's specification and strongly depends on mounting, thermal contact, etc. To get reproducible results, we have repeated our in-situ TEM experiments in various atmospheres. A detailed overview of the number of repeats is presented in Table S3."

[Figure Redacted]

Table R1 (SI: Table S3): The number of independent repeats per atmosphere.

In situ TEM experiments		
Conditions	Number of independent repeats	Notes
Vacuum	5	RT – 800 °C: 4x<br>RT – 900°C: 1x
H_2	3	RT – 800 °C: 3x
$CH_4 + CO_2$	3	RT – 800 °C: 2x<br>RT – 900 °C: 1x
Vacuum followed by $CH_4 + CO_2$	2	RT – 800 °C: Vacuum<br>800 °C - 600 °C: Vacuum<br>600 °C - 800 °C: $CH_4 + CO_2$
H_2 followed by $CH_4 + CO_2$	2	RT – 800 °C: H_2<br>800 °C - 600 °C:H_2<br>600 °C - 800 °C: $CH_4 + CO_2$

Results: First Ni appears at 400 °C in SE images (Fig. 1) and even at 300 °C in XPS. The disparity between 1.5 Pa (TEM) and 20 Pa (XPS) H_2 runs is not discussed; pressure strongly affects oxygen removal.

The text does address this, stating that the surface-bound metallic Ni first emerges at 300 °C in XPS (at 20 Pa) but is only observed at 400 °C in the SE image (at 1.5 Pa). To respond better to this comment, we firstly calculated the critical P_{O2} pressure during Ni solution from $LaNiO_3$ based on a thermodynamic analysis, as shown in the Table R2 (SI: Table S4 and Section J Thermodynamic calculations related to the oxygen partial pressure). Ni exsolution can occur when P_{O2} is lower than the critical value. We calculated p_{O2} under 1.5 Pa and 20 Pa H_2 reaction conditions. It can be found that both conditions are strongly reductive, and the p_{O2} are same as $7.4 \cdot 10^{-19}$ Pa. So the thermodynamics of both exsolution processes are similar. However, XPS with higher H_2 pressure indicates higher H_2 density, which could accelerate Ni formation/ exsolution. XPS is performed at higher pressure (20 Pa), which accelerates the reduction kinetics, leading to Ni exsolution fast to achieve the detection limits at a lower temperature (300 °C). SE imaging in the STEM is also surface-sensitive but performed at a lower pressure (1.5 Pa) , which shifts the visible onset of Ni particles to a higher temperature (400 °C). This pressure difference is key to observing the

intermediate phase La_2NiO_4 and confirms that the transformation process is highly dependent on the reduction potential/pressure, supporting the overall approach.

We have added an explicit discussion to formally address the pressure difference in the main text.

Table R2 (SI: Table S4): Critical oxygen partial pressure for Ni exsolution from LaNiO3

Temperature (℃)	O_2 partial pressure in vacuum (Pa)	Critical O_2 partial pressure (Pa)
100	$1*10^{-7}$	$2.6*10^{-41}$
200	$1*10^{-7}$	$2.6*10^{-29}$
300	$1*10^{-7}$	$2.6*10^{-22}$
400	$1*10^{-7}$	$2.6*10^{-16}$
500	$1*10^{-7}$	$2.6*10^{-12}$
600	$1*10^{-7}$	$2.6*10^{-9}$
700	$1*10^{-7}$	$2.6*10^{-7}$
800	$1*10^{-7}$	$2.6*10^{-5}$

Particle-size distribution is shown only in an inset (single field of view) with no statistical treatment or error bars.

We have performed additional statistical analysis both on the distribution and particles sizes of the exsolved Ni particles. The particle sizes of about 50 particles were measured for each of the three temperatures. The errors in particle diameter were calculated using the standard error of the mean and error bars were added accordingly. See Figure 1.

On page 10, the authors attribute the formation of different decomposition products (La_2NiO_4 under DRM conditions versus metallic Ni + La_2O_3 under hydrogen) to kinetic effects, arguing that "reduction is slowed down" under DRM conditions. However, this interpretation misses the more fundamental thermodynamic explanation. The primary distinction is thermodynamic, not kinetic - the vastly different oxygen partial pressures (pO_2) in these environments dictate different equilibrium product distributions. Under hydrogen's highly reducing conditions (very low pO_2), metallic Ni + La_2O_3 are thermodynamically favored, while the moderately oxidizing DRM atmosphere (higher pO_2 due to CO_2) thermodynamically stabilizes the intermediate La_2NiO_4 phase. While reaction kinetics may differ between atmospheres, the fundamental reason for different final products is that each environment drives the system toward different thermodynamic endpoints determined by pO_2-dependent phase stability relationships.

We agree with the referee on this point. While certainly some kinetic issues prevail, the main driving force is thermodynamic, because of the different oxygen partial pressure. The thermodynamics of the ternary La-Ni-O system has been described in several works (e.g., Zinkevich *et al*. Thermodynamic analysis of the ternary La-Ni-O system, J. Alloys Compds 375 (2004) 147-161, doi: 10.1016/j.allcom.2003.11.138). We have amended the manuscript accordingly. We have revised the text in section 3.1, regarding thermodynamic stability in addition to the kinetic argument, acknowledging that the difference in products is a result of the equilibrium being shifted by the distinct oxygen partial pressures.

"To put these findings into literature perspective, we have previously shown by in-situ XRD under hydrogen atmosphere under ambient conditions, that $LaNiO_3$ is rapidly transformed via oxygen-deficient $LaNiO_{2.7}$ and $LaNiO_{2.5}$ to metallic Ni particles and La_2O_3, without the intermediate presence of La_2NiO_4. La_2NiO_4 is only present under less reducing conditions (i.e., higher oxygen partial pressures),[26] such as those under typical DRM conditions. Beneficially, the discussed in-situ STEM-SE imaging – due to the lower hydrogen partial pressures – puts us in the prime position to stabilize the La_2NiO_4 intermediate structure along the reduction pathway and to distinguish it structurally and morphologically from the $LaNiO_3$ matrix and to monitor its role during formation of the Ni particles."

Quantitative gap. Claims of "high-density" Ni are not backed up by areal number densities.

We have quantified the average number of 46 particles and calculated an areal number density of Ni particles at the exsolution temperatures from 400 °C to 700 °C in H_2 and included this value (e.g., in particles/10nm^2) in the Section 3.1 (Figure 1I) to provide quantitative support for the qualitative claim of "high-density". The result shows that the particle density increased up to 500 °C but decreased at higher temperatures during H_2 reduction. This trend indicates that Ni particles formed emerging below 500 °C, while particle sintering occurred above this temperature. Thus, the quantification not only substantiates the "high-density" description but also elucidates the correlation between temperature and the density of exsolved Ni particles.

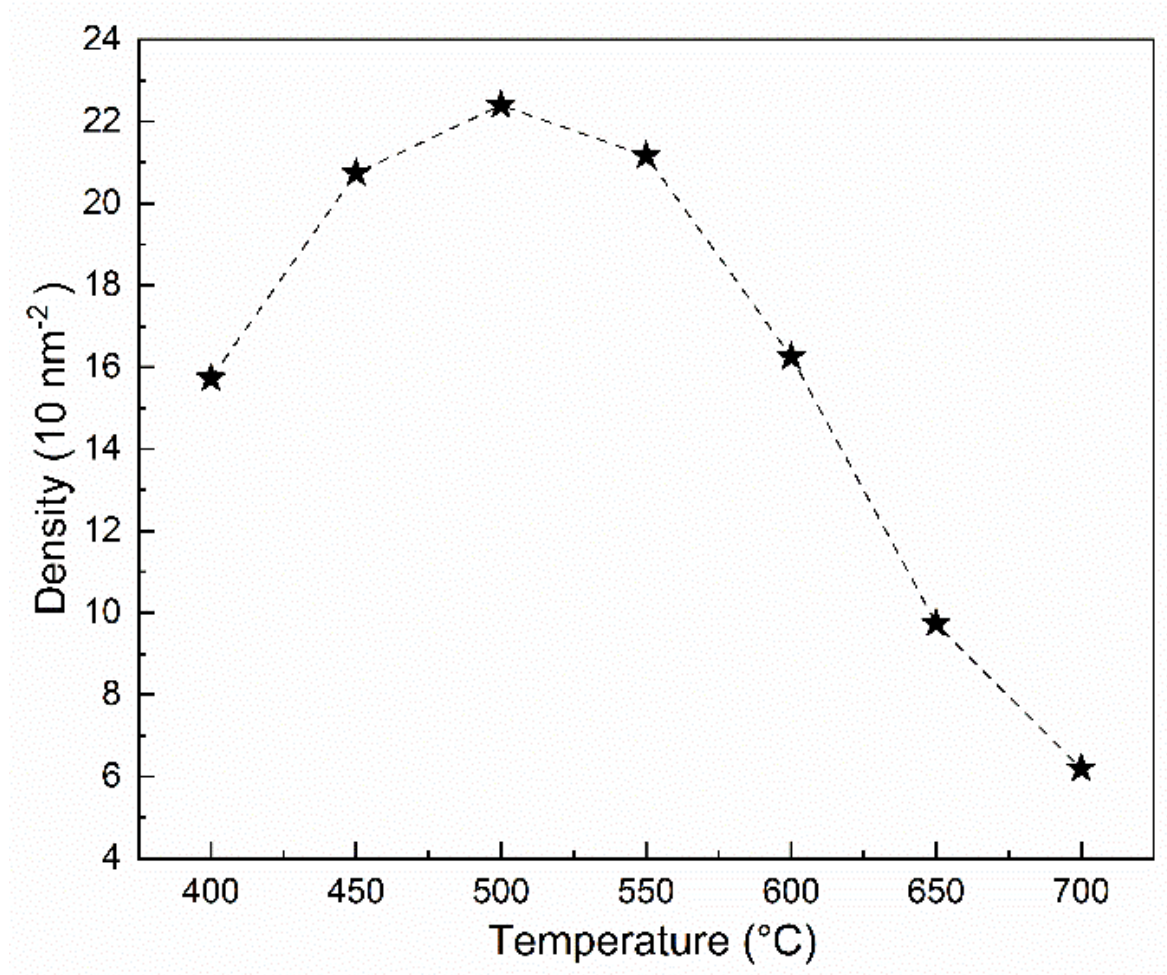

Figure R8 (Figure 1I): Ni particle density over temperature during H_2 reduction.

Observation of core–shell Ni@NiO relies on a single particle cross-section (Fig. 3 & Fig. S2). No statistics on how widespread this motif is.

As STEM-EDX analysis is bulk sensitive, the core-shell structure could be only observed on particles at sample edge (i.e., where the electron beam transmits through both the core and shell, avoiding the overlapped signals from the parent materials). The observation of the Ni@NiO core-shell structure was observed under vacuum heating. The existence of this motif was only observed confirmed in Figure S5, and similarly for the exsolved particle labeled "1" in Figure S7 C-G. We have moved the part to the SI and have toned-down the observation of core–shell Ni@NiO significantly:

"Notably, the Ni particle imaged in **Figure S5** displays a core-shell structure with a Ni core and a NiO shell."

"Ni particles formed by vacuum annealing could be prone to surface oxidation by lattice oxygen yielding a core-shell structure."

La_2NiO_4 onset (~760 °C) is supported by ex-situ synchrotron XRD in He, not vacuum. Substituting He for high vacuum may shift the equilibrium oxygen chemical potential; the authors do not justify this proxy.

For the in-situ synchrotron measurements, we used ultra-high-purity helium without an additional O_2 trap on the He line. The gas was delivered directly from an ultra-high-purity He (99.999%) cylinder. According to the supplier specifications, the impurities included approximately 1 ppm O_2 (as well as ≈1 ppm H_2O, CO, CO_2, and 5 ppm N_2). With the capillary operated near atmospheric pressure (≈1 bar), 1 ppm O_2 corresponds to an oxygen partial pressure of roughly 10^{-6} bar (≈0.1 Pa). To simulate vacuum, the use of He is the only possibility in the in-situ reactor chambers. Neither the synchrotron-based measurements, nor

the complementary lab-based in situ XRD experiments in the Reactor X chamber can be done in vacuum. This is not experimentally possible. To this extent, the amount of O_2, but in fact more important, the amount of H_2O is certainly much higher in these experiments compared to vacuum annealing. Nevertheless, if one has a look on the La-Ni-O phase diagram as a function of pO_2 (isothermal cut at 1100 °C, see below), one recognizes that at both pressures (i.e., 10^{-6} bar in the He experiments and at the STEM base pressure at 10^{-11} bar) at the nominal composition of $LaNiO_3$ we are still in the stability region of La_2NiO_4 + Ni/NiO (the phase equilibrium line separating La_2NiO_4+Ni from La_2O_3+Ni shifts to lower pO_2 values as the temperature decreases). We will explicitly justify the use of He XRD as a thermal decomposition control experiment that closely mimics the TEM high-vacuum conditions (Section 3.1)

[Figure Redacted]

Figure R9: The La–Ni–O phase diagram along the La_2NiO_4–$LaNiO_3$ - Figure 7 as shown in *M. Zinkevich and F. Aldinger, Thermodynamic analysis of the ternary La–Ni–O system, Journal of Alloys and Compounds, 2004, 375, 147-161, doi: 10.1016/j.allcom.2003.11.138*

We have also amended Figure S1 and added a corresponding explanation addressing this issue to the main manuscript.

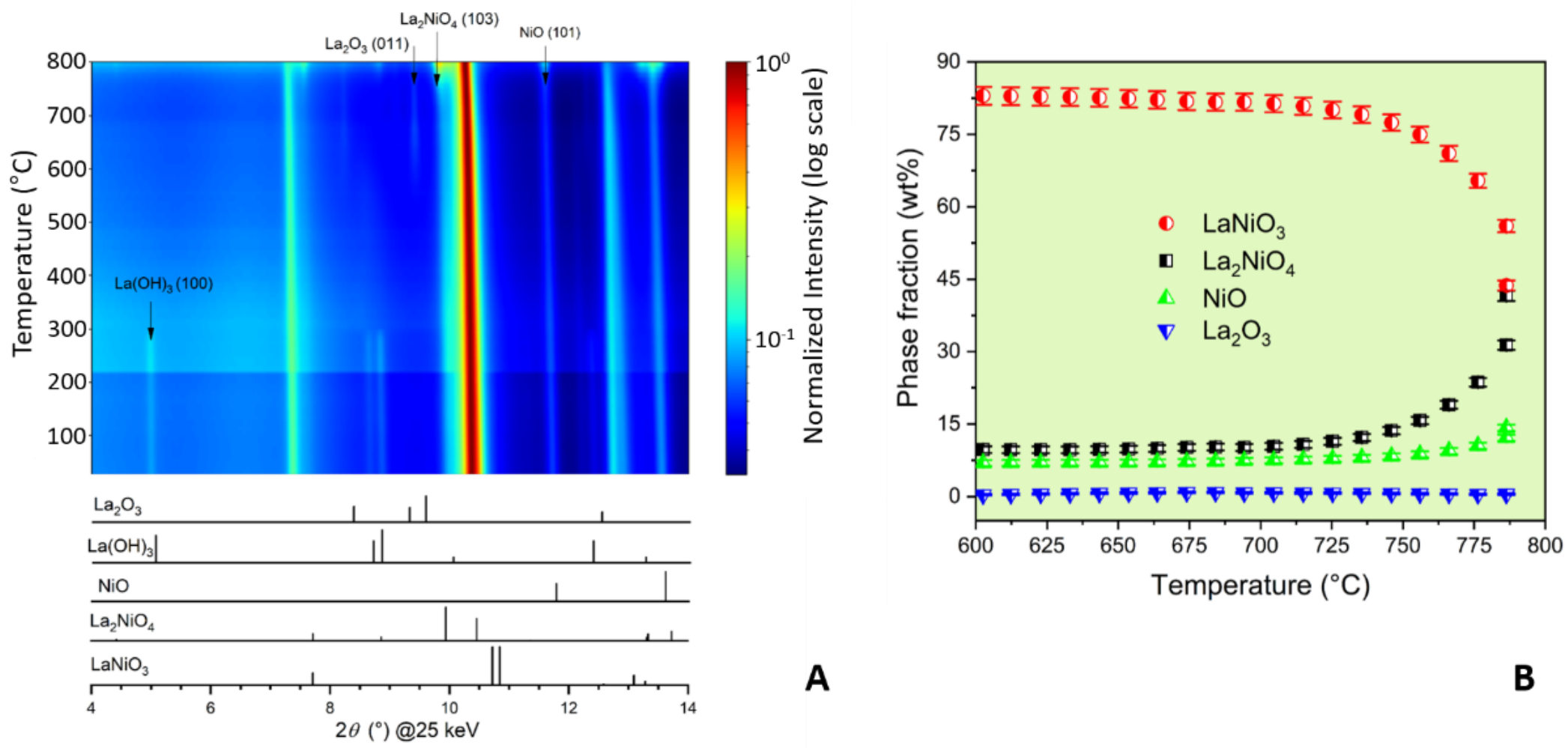

Figure R10 (SI: Figure S1): Synchrotron-based in situ PXRD tests in He.

EDX semi-quantification: counts are shown qualitatively (colour maps) bFut not converted to Ni/La atomic ratios.

We have extracted representative Ni/La atomic ratios for for the exsolved Ni-rich regions after vacuum heating. These quantitative values are included as Figure R11 which is added as Figure S8 in the Supporting Information, accompanying Figure S7 to provide support for the qualitative elemental maps. As shown in quantitative results, the Ni/La atomic ratio for Regions 01 to 10 all exceed 1, confirming that these regions are Ni-rich. This conclusion is fully consistent with the qualitative evidence from EDX signals counts.

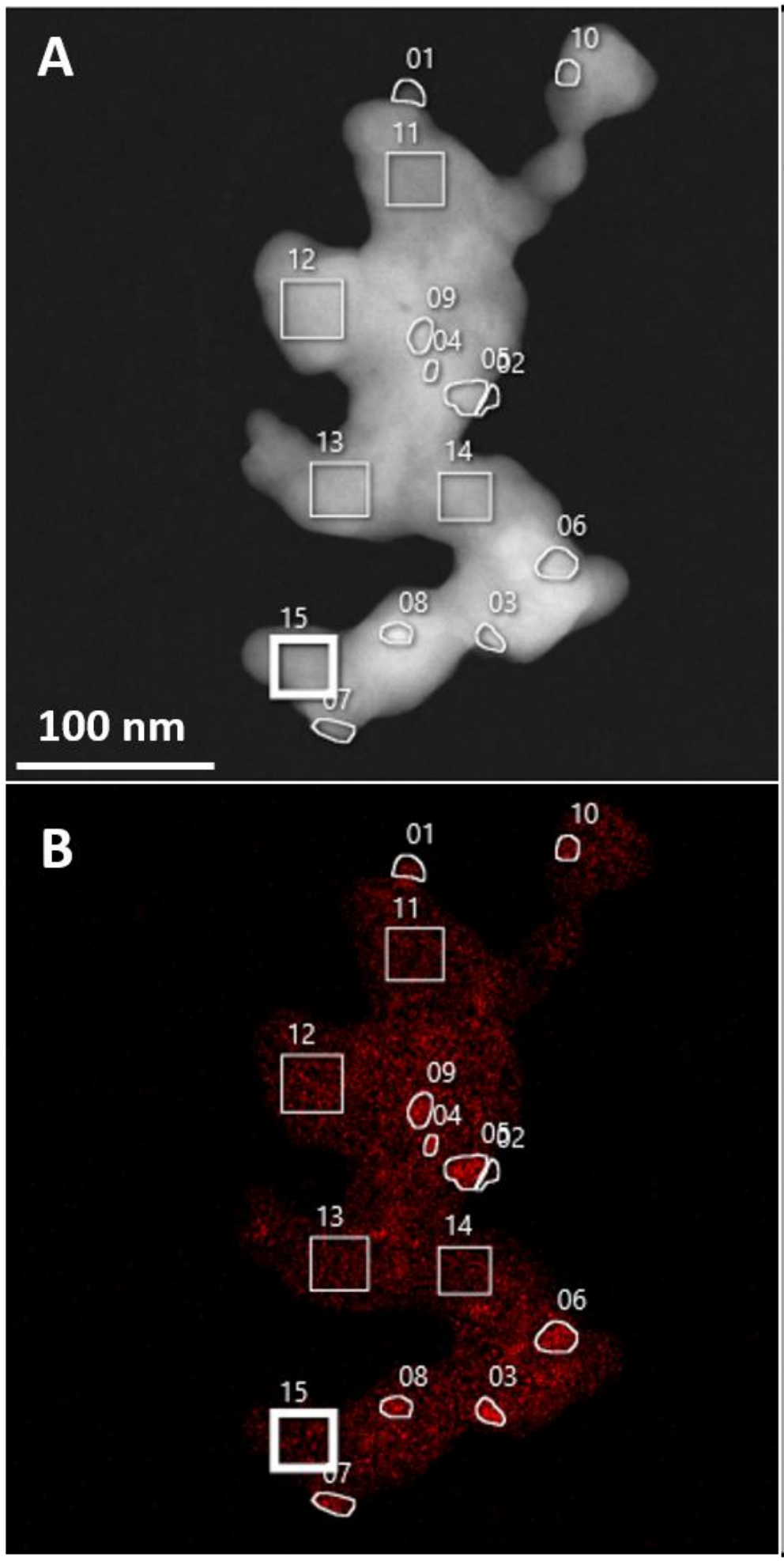

Sites	Ni atom%	La atom%	Ni/La
01	26.6	1.9	7.0
02	19.4	3.8	5.1
03	25.2	10.4	2.4
04	20.7	6.7	3.9
05	22.2	13.7	1.62
06	16.3	15.3	1.06
07	20.6	8.3	2.48
08	14.1	16.7	0.84
09	16.0	13.0	1.23
10	14.1	12.6	1.12
11	8.2	15.8	0.52
12	8.0	16.1	0.50
13	7.7	17.9	0.43
14	8.3	17.2	0.48
15	7.7	16.2	0.48

Figure R11 (SI: Figure S8): Quantitative analysis on EDX results to confirm the formation of Ni particles on $LaNiO_3$ after vacuum heating: A, HAADF-STEM image, and B, EDX elemental distribution map of Ni.

The corresponding disussion was added in SI Section E:

"We have further extracted representative Ni/La atomic ratios for for the exsolved Ni-rich regions after vacuum heating (**Figure S8**). As shown in quantitative results, for the regions 01 to 10, Ni/La atomic ratio is >1, confirming these regions are Ni rich."

On page 21, the authors attribute the observed mobility of emergent nanoparticles to inherent Ni behavior, but this is more likely due to altered metal-support interactions resulting from the structural transformation. Since the host lattice recrystallizes from $LaNiO_3$ to the La_2NiO_4 Ruddlesden-Popper phase, this creates entirely new interfacial properties and bonding environments for the nanoparticles compared to true exsolution systems where the original host lattice is maintained along with established crystallographic alignment relationships. The enhanced particle mobility observed is therefore likely a consequence of weaker or different interfacial interactions with the newly formed La_2NiO_4 support rather than an intrinsic property of the Ni particles themselves.

We have recently revealed (*Malleier et al., In situ Impedance spectroscopy as a marker method during $LaNiO_3$ decomposition, J. Amer. Chem. Soc, 2025*), that upon hydrogen reduction $LaNiO_3$ is reduced to $LaNiO_{2.5}$, which still bears an X-ray diffractogram close to that of $LaNiO_3$. $LaNiO_{2.5}$ formation is a result of oxygen loss from $LaNiO_3$ and, as revealed by in situ X-ray photoelectron spectroscopy at least from 280 °C on is accompanied by Ni exsolution on the surface. At 400 °C, where no La_2NiO_4 is present, but still only bulk $LaNiO_{2.5}$, Ni exsolution is visible in TEM. In essence, this is a clear exsolution process. We agree that once La_2NiO_4 starts to form, Ni formation accelerates and the energetic situation at the interface is different and might be overlaid by, as it is called, a recrystallization process.

We have amended the revised version accordingly in section 3.1:

"To address a critical conceptual gap of Ni particle formation during (partial) $LaNiO_3$ decomposition, we note that we in fact have to discriminate between true exsolution and solid-state recrystallization effects. While genuine exsolution often employs non-stoichiometric perovskite formulations to trigger Ni exsolution along established crystallographic alignment relationships and maintaining the original parent perovskite host lattice, recrystallization resulting in complete lattice restructuring thereby creating new socket sites with altered anchoring and interfacial properties.[39, 40] : While the latter is certainly true at more progressed states of $LaNiO_3$ decomposition, where La_2NiO_4 starts to form (alongside simultaneous Ni particle formation) and then finally collapses structurally to form La_2O_3 and Ni, we have recently shown[41], that in the early stages of hydrogen reduction, where no La_2NiO_4 is formed and the redox chemistry is essentially dominated by oxygen removal to form bulk oxygen-deficient $LaNiO_{2.7}$ and $LaNiO_{2.5}$, true exsolution of Ni occurs and surface-bound Ni particles are formed. Having said that, the type of Ni particle formation observed here is more likely associated with bulk recrystallization."

Ni exsolution is "suppressed" up to 700 °C, but again no kinetics or oxygen partial-pressure calculations are supplied to generalize these findings.

The observed suppression of Ni formation/ exsolution in the DRM atmosphere compared to H_2 is attributed to the less reducing/more oxidizing reaction mixture due to the presence of CO_2. This qualitatively points to thermodynamics governed by local oxygen partial pressure (pO_2). This thermodynamic argument is quantitatively supported by our calculation of the critical pO_2 for Ni exsolution (Table S4). At 700 °C, the p_{O2} in vacuum is close to critical p_{O2} ($2.6*10^{-7}$ Pa), indicating that Ni exsolution is thermodynamic "suppressed". In contrast, the critical p_{O2} exceeds the P_{O2} in vacuum up to 800 °C and Ni formation/ exsolution is driven by thermodynamics. In the DRM atmosphere, the partial pressure of oxygen on the sample surface is higher than in vacuum due to the CO_2 dissociation. Thus, we addressed this

quantitatively by performing the critical P_{O2} calculation and use this calculation to support the finding that Ni exsolution is suppressed in the DRM atmosphere until higher temperatures, thus backing the qualitative claim.

Key claim: oxidation of Ni to NiO around 600–650 °C is the root cause of the activity dip in DRM catalysts. Evidence gap: No catalytic tests, no CO_2/CH_4 conversion data, and no in situ spectroscopy correlating Ni valence with CH_4 activation rates. Additional experiments (e.g., operando DRIFTS, flow-reactor tests) are needed.

The paper's key contribution is to provide the structural evidence for the already-known catalytic dip observed under ambient conditions (*Bonmassar, N. et al. In Situ-Determined Catalytically Active State of LaNiO3 in Methane Dry Reforming; ACS Catal. 2020, 10, 1102–1112. doi: 10.1021/acscatal.9b03687).* Note, that under the catalytic experiments (500 Pa reactant pressure), this "dip" manifests itself as a delayed onset temperature compared to bar-level catalysis during the first catalytic cycle. The work explicitly states that it is drawing a valuable structure-activity relationship by correlating the in-situ Ni oxidation to the DRM profiles previously collected on this exact system. The in-situ TEM studies prove that dynamic Ni oxidation is responsible for this peculiar profile. The conclusion is a mechanistic explanation for an established catalytic observation. We have added a stronger introductory sentence to the final subsection (Section 3.3) to link the experimental design more clearly to the need to explain the previously reported catalytic activity decrease and elaborated upon on this issue on page 23 in the revised version of the manuscript.

For the catalytic tests, see answer to response for comment 2 above.

Real-world relevance. The authors acknowledge that their reduced partial pressures were chosen "to slow down the transformation processes" , but do not revisit whether kinetics or phase equilibria at 1-2 Pa are transferable to bar-level DRM.

Please see also response to the comment above. The DRM profiles alongside the structure-activity correlation are essentially similar to bar-level DRM.

Clarify oxygen chemical potentials. Calculate pO_2 for the 1.5 Pa DRM mixture and discuss how it compares to industrial feeds; consider a second set of experiments at ≥100 mbar to test robustness.

High-pressure experiments are not possible using current STEM hardware, but we have elaborated upon on this issue from the catalytic direction to close the “pressure gap”, see discussion on page 23 in the revised version of the manuscript.

Correct minor editorial issues (duplicate “in-situin-situ”, inconsistent figure numbering, units).

Thanks for your comments, we have corrected duplicate “in-situin-situ” to “in-situ”. We have also reviewed the manuscript thoroughly for any remaining inconsistencies in figure numbering and units.

Reviewer #3:

This study provides important insights into the structural evolution and Ni exsolution behavior of LaNiO3 perovskite under various gas environments relevant to dry reforming of methane (DRM). The in-situ electron microscopy approach is technically impressive and yields valuable data. However, the manuscript requires major revision prior to publication. The following comments highlight issues that must be addressed to enhance clarity, consistency, and scientific rigor.

The manuscript contains multiple typographical inconsistencies, particularly with the term "in-situ," which is repeated redundantly in several places (e.g., p3 – line 58, p4 – lines 77 and 83). Other minor errors also require correction (e.g., p5 – line 112, p13 – Figure 3J, p15 – line 279).

We have already corrected duplicate 'in-situ' terms through the whole manuscript. We have also proofread the manuscript carefully to correct all other reported errors and minor typos including "La(NO3)$_3$" to "La(NO$_3$)$_3$", "Figure S7J (the original Figure 3J)" to "Figure S7G (the original Figure 3G)", and "700 ^{o} C" to "700 °C".

Further detail is required in the Experimental Section. In particular:

• *Please specify the vacuum conditions—was the pressure of 1.5 Pa under air, an inert gas, or a high vacuum below 1.5 Pa?*

A pressure of 1.5 Pa was applied during the experiments conducted under pure hydrogen and the CO_2/CH_4 gas mixture. The vacuum conditions, i.e. the base pressure of the Hitachi HF5000 microscope is below 10^{-6} Pa. We have added the vacuum and gas pressure data in the Experimental Section (Section 2.2):

"In situ and ex situ STEM experiments were carried out with a Hitachi High-Technologies HF5000 environmental STEM, equipped with a secondary electron detector. Pure hydrogen and a CO_2:CH_4 =50:50 gas mixture were utilized for the reduction and DRM in situ experiments, respectively, with gas pressures in the column around 1.5 Pa. The vacuum heating experiments refer to the STEM column's high-vacuum base pressure (typically ~10^{-6} Pa)."

• *Was the in-situ TEM system operating under static or flowing gas conditions?*

The in-situ TEM system was operating under flowing gas conditions in the column, the column pressure (FRG2 shown in the Fig.R12) is controlled by a mass flow controller (in Fig.R13). The gas was introduced and maintained with continuous flow of 0.34 sccm, which equals to a pressure of 1.5 Pa at the specimen. We have added the reaction conditions to the Experimental Section (Section 2.2).

"The in situ TEM system was operating under flowing gas conditions in the column."

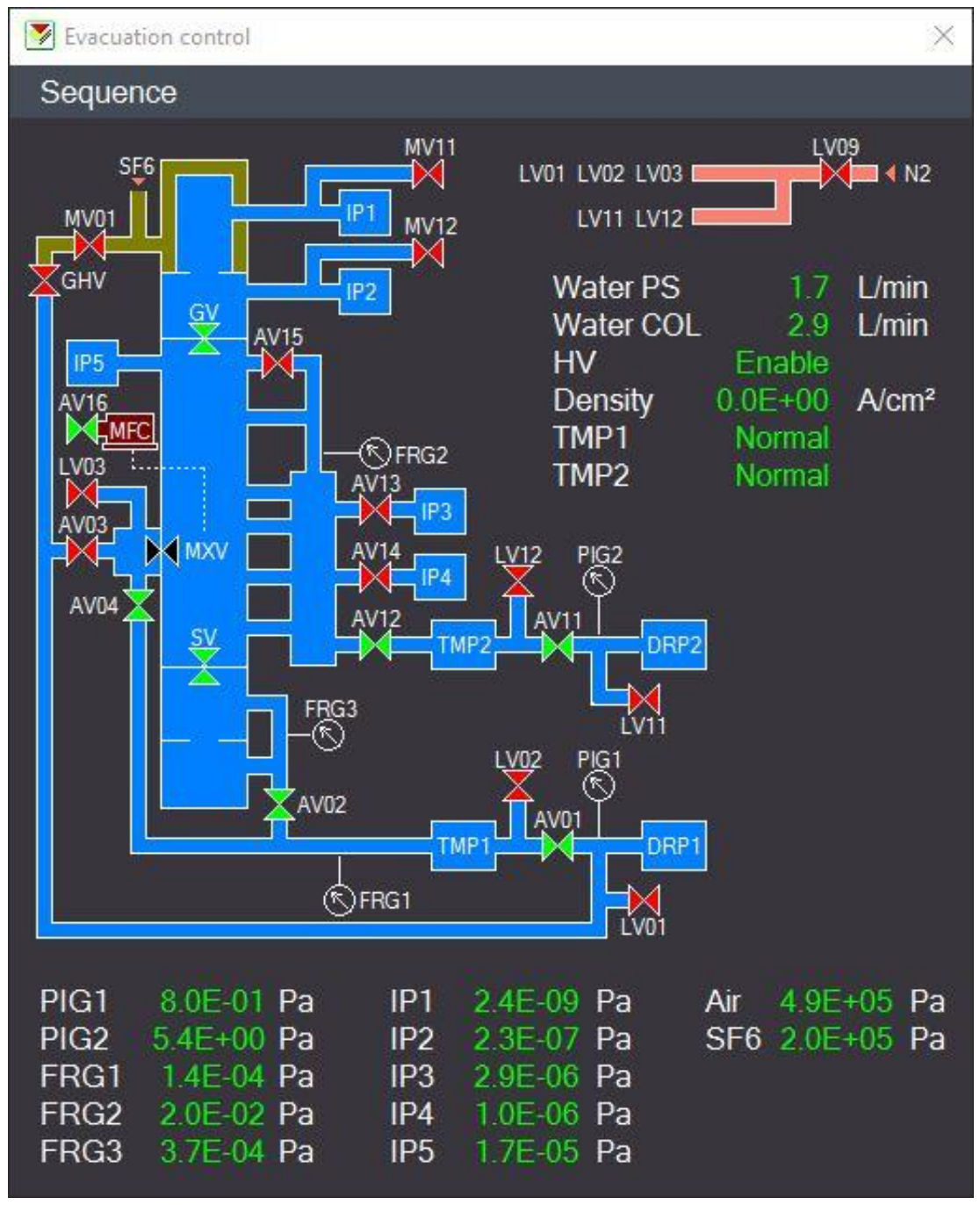

Figure R12: Sketch of vacuum system of in-situ HF5000 system.

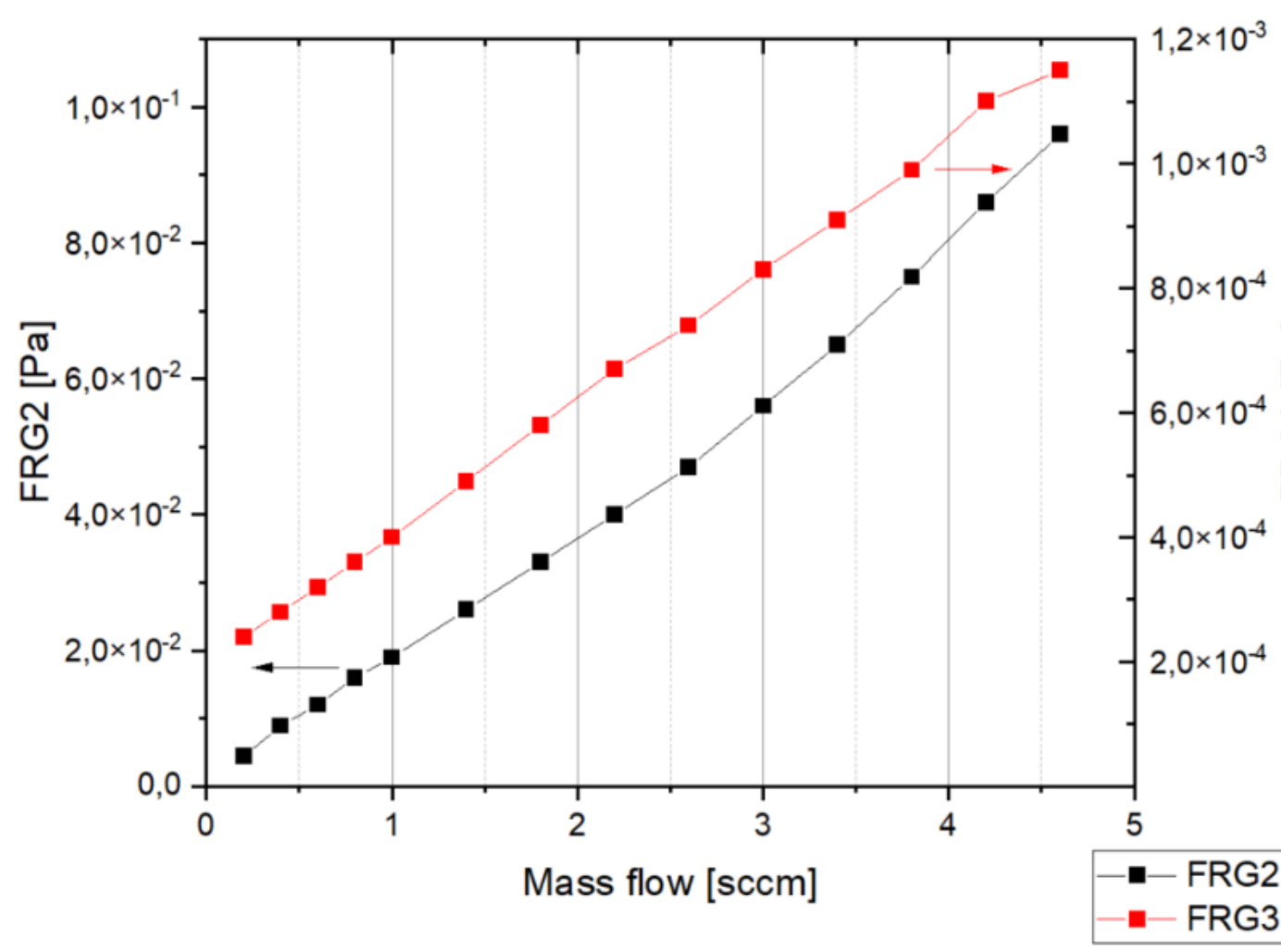

Figure R13: Mass flow in sccm vs. the column pressure gauge (FRG2) and the post column gauge (FRG3)

The authors should clearly state the amount of catalyst loaded on the TEM grid, as well as the molar quantity or flow rate of gas present in the chamber. Without this, catalytic interpretation is incomplete. For example, if 10 mL/min of reactant gas is used on a catalyst with a WHSV of 60,000 mL/g hr and 1 g catalyst, only 1% of its theoretical capacity is needed to convert all reactants—making apparent activity or stability potentially misleading. If the gas volume is too small relative to the catalyst's capacity, any claims about coking resistance could be overstated.

Our In-situ TEM experiments are performed on Micro-Electro-Mechanical System (MEMS) chipswhich makes it impossible to provide quantitative catalytic turnover rates (e.g., WHSV). The catalyst is loaded as a dispersed powder on the chip's membrane, making the amount of catalyst negligible in catalytic terms (estimated at ng to μg). The purpose is structural and mechanistic analysis, and it is quite challenging to precisely study kinetics or quantify performance. We have added in the Experimental section that the experiments are performed under gas conditions at 1.5 Pa and that the tiny amount of catalyst loaded is sufficient for structural and morphological analysis, but does not allow for quantitative catalytic analysis such as WHSV or conversion.

Regarding the relative gas volume, it is exceptionally high for catalysts on a MEMS chip. As shown in Figure R4, the flow rate in this experiment is approximately 0.34 sccm. For a 1 μg catalyst load, the calculated WHSV is 2.04×10^7 mL/(g·h). Even if we conservatively estimate the effective surface area to be 1/1000 of the total, the effective WHSV for the catalysts on the chip would be approximately 2.04×10^4 mL/(g·h), which remains very high.

Other in situ TEM studies have directly observed carbon deposition on Ni particles during dry reforming of methane. For instance, Zhichun Wang et al. (*Materials Today*, 2024, 79, 16-27) confirmed the formation of carbon deposits within an in situ TEM gas cell. This finding provides direct experimental validation that such coking occurs under relevant reaction conditions.

Our claims regarding coking resistance are based on the direct in-situ observation of no carbon formation within the field of view. We attribute this resistance to the presence of mobile oxygen species. We will also correlated Wang et.al. work to avoid overstating the catalytic relevance to larger-scale systems, in section 3.3:

"Apparently, no surface carbon deposition was observed during our in-situ DRM experiments. This is likely due to the presence of excess mobile oxygen which effectively removes surface carbon species before their potential accumulation. Given that carbon deposition is a major reason for the deactivation of the catalyst and has been directley observed on Ni surface in in situ TEM gas cell,[50] [51] these findings demonstrate the good resistance of this catalyst to carbon deposition."

Although the authors claim to decouple thermal, reductive, and DRM-induced effects, the phenomena under DRM conditions seem to resemble vacuum annealing more than hydrogen reduction. The discussion of these observations is limited, and further analysis is necessary to clarify whether unique reactions occur under DRM. Suggested additions include measuring pH_2 in the CO_2/CH_4 mixture and conducting mass change analyses.

The text explicitly notes that the early-stage DRM behavior mirrors vacuum annealing (thermal effects) in that global exsolution is suppressed and facetted regions (La_2NiO_4) appear first. This is a key finding of the study, indicating that the DRM atmosphere is less reducing than pure H_2 and is crucial for isolating the DRM-specific structural changes. Unique DRM reactions are indeed observed: (1) The rapid and dynamic oxidation of H_2-pre-reduced Ni particles to NiO upon DRM gas introduction (Figure 8, Movie S3), which is the direct structural evidence for the catalytic activity dip. (2) The reincorporation of NiO particles into the host matrix at high DRM temperatures (>700 °C), which is DRM-specific (Figures S21, S22).

We have added a stronger statement clarifying that the initial resemblance to vacuum annealing is a direct consequence of the low reduction potential of the DRM atmosphere. A mass change analysis is unfortunately not possible using our environmental STEM setup.

It is well known that $LaNiO_3$ decomposes into Ruddlesden–Popper phases and NiO at high temperature or low pO2. While the authors acknowledge this, further clarification is needed:

• Under vacuum or DRM conditions, is NiO formation driven purely by metal–support interaction, or are there additional factors not explained by thermodynamics?

The term "metal-support interaction" in this particular case refers to oxygen spillover from the reduced perovskite to Ni, where it forms NiO. In fact, at low pO_2, thermodynamically the formation of $Ni+La_2O_3$ is favored (see Zinkevich et al. Thermodynamic analysis of the ternary La-Ni-O system, J. Alloys Compds 375 (2004) 147-161, doi: 10.1016/j.allcom.2003.11.138). The prerequisite for the formation of La_2NiO_4+Ni is sufficiently high oxygen pressure (see e.g. Figure 7, left panel of 10.1016/j.allcom.2003.11.138, detailing the phase diagram La-Ni-O at 1100 °C). In this respect, the pO_2 also determines if one accesses the phase region La_2NiO_4+Ni or La_2NiO_4+NiO. We have shown in a previous publication that NiO is formed upon vacuum annealing, where oxygen from partial LaNiO decomposition forms NiO (Cao, P. et al. Atomic-Scale Insights into Nickel Exsolution on $LaNiO_3$ Catalysts via In Situ Electron Microscopy; J. Phys Chem. C 2022, 126, 786-796. doi: 10.1021/acs.jpcc.1c09257) – we therefore assume, that the oxygen pressure is high enough to form NiO. Note that according to this Figure, at even higher pO_2, the phase region $LaNiO_3$+NiO is reached. "$LaNiO_3$" in this respect is used to represent a oxygen-deficient $LaNiO_3$-x phase. It is well known, that "$LaNiO_3$" in fact is better represented by $LaNiO_{2.94}$ – $LaNiO_{2.88}$ (Malyshev, S.A. et al. Metal-oxide nanocomposites by low temperature exsolution from perovskite-like La nickelates: Synthesis, morphology, and catalytic properties in CO_2 hydrogenation, Mater. Res. Bull. 2024, 179, 112984. doi: 10.1016/j.materresbull.2024.112984 and Moriga, T. et al., Characterization of oxygen-deficient phases appearing reduction of the perovskite-type $LaNiO_3$ to $La_2Ni_2O_5$,

Solid State Ionics 1995, 79, 252-255. doi: 10.1016/0167-2738(95)000-70M), so $LaNiO_3$ itself is oxygen deficient and readily loses more oxygen to form $LaNiO_{2.7}$, both under hydrogen and DRM treatment at temperatures as low as 250 °C – 280 °C in the bulk (Bonmassar, N. et al. In Situ-Determined Catalytically Active State of $LaNiO_3$ in Methane Dry Reforming; ACS Catal. 2020, 10, 1102–1112. doi: 10.1021/acscatal.9b03687). We have recently also shown that this transformation is accompanied by surface-bound Ni exsolution (Malleier et al., In situ Impedance spectroscopy as a marker method during $LaNiO_3$ decomposition, J. Amer. Chem. Soc, 2025).

[Figure Redacted]

Figure R14: The La–Ni–O phase diagram along the La_2NiO_4–$LaNiO_3$ - Figure 7 as shown *in Zinkevich et al. Thermodynamic analysis of the ternary La-Ni-O system, J. Alloys Compds 375 (2004) 147-161, doi: 10.1016/j.allcom.2003.11.138*

• *Since La_2NiO_4 and NiO are also present under vacuum, can CO_2 dissociation alone explain NiO formation during DRM?*

It is known that CO_2 in fact is able to oxidize Ni to NiO (see, e.g., *Antill, J. E. et al. Oxidation of Nickel by Carbon Dioxide, J. Electrochem. Soc. 1967, 114, 1215, doi: 10.1149/1.2426452*). In principle under DRM conditions, NiO formation is triggered by two sources of oxygen: (1) lattice oxygen released from the ongoing decomposition of the La−Ni−O phases ($LaNiO_3$/La_2NiO_4) and (2) oxygen from CO_2 dissociation (CO_2→CO+O*). The sustained oxidation of Ni to NiO, observed upon switching the reaction

gas from H_2 to CH_4+CO_2 mixture at 600 °C (Figure 8B, E-H), demonstrates that the supply of mobile oxygen exceeds its consumption. However, the results of our reaction-induced Ni exsolution show that lattice oxygen release, which drives Ni exsolution, occurs at a higher temperature of 750 °C. This strongly implies lattice oxygen release is minimal at 600 ℃, and the additional contribution from CO_2 dissociation is significant, especially as CO_2 decomposition is considered active at oxygen vacancies that are generated during Ni exsolution. We have amended the revised version accordingly inSection 3.3:

"The dynamic oxidation process at 600 °C was recorded in Movie S3, with representative snapshots over 38.5 seconds shown in Figure 8E-H, exemplifying the dynamical sequential oxidation of Ni particles (indicated by red, green, white, and blue arrows). The oxidation process resulted in an increase in particle size due to the incorporation of oxygen atoms into the Ni lattice, which leads to an expansion of the unit cell.[48] The oxygen required for this oxidation likely stems from both lattice oxygen within the host matrix and CO_2 dissociation. However, the sustained oxidation of Ni to NiO, observed upon switching the reaction gas from H_2 to CH_4+CO_2 mixture at 600 ℃ (Figure 8B, E-H), where the results of our reaction-induced Ni exsolution show that lattice oxygen release, which drives Ni exsolution, occurrs at a higher temperature of 750℃. Therefore, lattice oxygen contribution at 600℃ must be minimal, strongly implying that CO_2 dissociation serves as the major, low-temperature oxygen source."

• *Is there supplementary data for vacuum treatment at 600–700 C following 700 C H_2 annealing? This could help distinguish thermally induced from DRM-specific effects.*

The requested sequence (H_2→Vacuum→DRM) was not performed, but the key DRM-specific experiment (H_2→DRM) has been performed (Figure 10). We think, the H_2→DRM experiment is catalytically most relevant, as it directly simulates the activation (reduction) step followed by reaction (DRM). The DRM-specific effect is the rapid re-oxidation of metallic Ni to NiO upon introduction of the CO_2/CH_4 mixture (Figure 8B), which is a unique reaction that is not possible under vacuum. This robust experiment sufficiently isolates the DRM effect and confirms the key structural finding. We will ensure the manuscript clearly states the importance of the H_2→DRM experiment for isolating the DRM-specific oxidation.

In Figure 2, the authors interpret phase transformation based on changes in the La 3d 3/2 peak. However, this peak overlaps with Ni 2p, complicating interpretation. Why is there no discussion of the La 3d 5/2 peak, which should be more isolated? The explanation could be strengthened by revisiting this analysis.

To better interpret phase transformation, we have also added a short discussion about the La 3d5/2 peak in the revised manuscript. In essence, the shape of the La 3d5/2 peak shifts from a shape typically found in $LaNiO_3$ at 300 °C (see e.g., Whitten, A. et al. Deconvoluting XPS Spectra of La-Containing Perovskites from First-Principles, JACS Au 2024, 4, 3104-3117 or Berneau et al. Uncovering the critical function of lanthanum in CH_4 production from CO_2 using exsolved $LaNiO_3$ perovskite catalysts, J. Mater. Chem. A 2024, 12, 7605, doi: 10.1039/d3ta07391b) more to the one of La_2O_3 at 750 °C (see e.g., Berneau et al. Uncovering the critical function of lanthanum in CH_4 production from CO_2 using exsolved $LaNiO_3$ perovskite catalysts, J. Mater. Chem. A 2024, 12, 7605, doi: 10.1039/d3ta07391b). Differences are minor, as the oxidation state of La still is 3^+. One can observe a slight intensity difference between both splitted La 3d5/2 peak.

The expression "local exsolution" used in Figure 5 to describe behavior at 750 C is ambiguous. If exsolved particles are not detected by surface-sensitive SE imaging, can their presence be asserted? Are they instead located beneath the surface? More discussion or complementary data is needed.

The text states that "at 750 ℃, local Ni exsolution, as evidenced by the lattice fringes indicated by arrows, occurs" (Figure 5F, magnified inset). The lattice fringes are visible in the STEM-BF image (bulk sensitive), but the particle itself is not clearly visible in the STEM-SE image. This is similar to the vacuum case and suggests that under DRM conditions some Ni particles at this temperature are located beneath surface rather than being fully exposed at the surface.

To describe this more precisely, we revised the text accordingly: "Global Ni exsolution to the surface is still suppressed in comparison to under hydrogen, at 750 °C as evidenced by the lattice fringes indicated by arrows Ni exsolution occurs.", and emphasize the distinction between bulk (BF) and surface (SE) observation in the text "The lattice fringes are visible in the STEM-BF image (bulk sensitive), but the particle itself is not clearly visible in the SE image (surface sensitive), indicating that Ni exsolved within the host matrix. ".

Figure S8 shows the appearance of La_2NiO_4 at 500 C, which contradicts the absence of step-like features in Figure 1 at the same temperature. This inconsistency should be addressed—either by reconciling the two datasets or clarifying which is more representative.

Figure S17 (the original Figure S8) shows the La_2NiO_4 phase confirmed by FFT patterns of DF images (bulk sensitive). La_2NiO_4 is observed at 500 ℃ in Figure S17. Figure 1 is an SE image series (surface sensitive), showing the initial stages of surface exsolution. The text states that the rough surface with pronounced steps (La_2NiO_4 facet formation) appears at around 650 ℃ in the SE images (Figure 1G,

H). The absence of SE features at 500 °C (Figure 1D) and the appearance of FFT patterns for La_2NiO_4 at 500 °C (Figure S17D) are consistent, showing that the phase transition starts in the bulk at a lower temperature (500 °C) before manifesting as a surface feature (steps/facets) at a higher temperature (650 °C). This highlights the power of combining bulk (DF) and surface (SE) sensitive techniques to monitor the phase transformation from the bulk to the surface. We will clarify this in the discussion of Figure S17.

In Figure 10B, the identity of the oxidized particles should be confirmed—are they pure NiO or Ni/NiO core–shell structures as observed during vacuum annealing?

The particles in Figure 8 (the original Figure 10B) are formed by rapid oxidation of pre-reduced metallic Ni. While Ni/NiO core-shell structures were observed under vacuum, the DRM condition is highly oxidizing at the Ni surface due to CO_2 dissociation and lattice oxygen transfer. This is expected to drive the oxidation more completely. The text refers to them as "spherical Ni particles rapidly oxidize into more facetted NiO particles". Given the dynamic and sustained oxidation (Figure 8E-H, Movie S3), it is reasonable to assume the smaller particles, at least, are near-fully oxidized NiO. The term NiO is a simplification, but the DRM atmosphere is known to be more oxidizing than vacuum.

We have now phrased this aspect of the discussion more cautiously that due to the rapid oxidation and sustained oxygen supply from CO_2, the final particles are most likely NiO-dominated, if not fully NiO, distinguishing them from the Ni/NiO observed under inert vacuum.

In Figure 10D, the authors attribute performance improvement above 650 C to the cessation of oxygen transfer and stabilization of reduced Ni. However, no direct evidence is provided that Ni remains reduced at 700 C. Additional structural or spectroscopic analysis is needed to substantiate this claim.

The conclusion that Ni is stabilized as Ni^0 at higher temperatures is an extrapolation based on the reported catalytic data (strong acceleration of conversion above 650 °C) and the observed cessation of the oxidation process. The argument is that NiO is a poor CH_4 activator, and since the DRM activity accelerates when oxygen transfer ceases, the active phase (Ni^0) must be stabilized. Direct spectroscopic evidence (like operando XPS) at 700°C in the full DRM gas mixture is outside the scope of the current TEM-focused manuscript. We will revise the text to frame this as the mechanistic hypothesis (Ni stabilization leading to catalytic acceleration) inferred from the TEM data (cessation of oxygen transfer) and the cited catalytic literature, rather than a proven structural fact in the TEM field of view.

The statement that NiO dissolves and reincorporates into the lattice at 800–900 C appears to contradict thermodynamic expectations. What mechanism or driving force could explain this behavior?

This reincorporation/dissolution is a key finding and does not necessarily contradict thermodynamics; it is likely an example of structural reversibility (or exsolution/re-incorporation). The mechanism is driven by the high mobility of oxygen (from CO_2 dissociation and lattice oxygen) at high temperatures, allowing oxygen to diffuse into the host matrix where it is coordinated with the reincorporated Ni. The host structure is highly defective (La_2NiO_4), and the driving force could be the reduction of high surface energy of the nanoparticles and the re-establishment of a stable, albeit defective, perovskite phase ($LaNiO_3$ or a La_2NiO_4 with dissolved NiO) at very high temperatures. This is a topic of ongoing research, and our observation simply reports on the structural event. We will clarify that this reincorporation is a DRM-specific structural reversibility event likely driven by the increased activity of oxygen vacancies and high-temperature solid-state diffusion and is further supported by surface smoothing.

Lastly, the surface smoothing observed in Figures 5 and 10 raises the question of whether La_2NiO_4 has reverted to $LaNiO_3$. Has the parent phase undergone further transformation beyond Ni dissolution? If so, the mechanism of this structural recovery should be explored in more detail.

The surface smoothing (Figure 8D, S22D) is observed at 700 °C and up to 950 °C. This smoothing is directly attributed to the reconstruction of the host matrix following the dissolution and reincorporation of NiO. The parent phase reverting from La_2NiO_4 to $LaNiO_3$ is plausible, as the driving force for La_2NiO_4 stability is low pO_2/reduction potential; the injection of Ni^{2+} and O at high temperature would move the system back toward the more oxidized $LaNiO_3$ phase. We will revise the text to explicitly suggest that the smoothing and reincorporation at high temperatures likely correspond to the structural recovery of a $LaNiO_3$ like perovskite structure from the La_2NiO_4 intermediate, demonstrating the full structural reversibility of the system.

Thank you very much for your helpful reviewer reports. We have answered the comments by a detailed point-by-point response. Generally, we have carried out additional work and substantial revision to

(i) validate the importance of the structural studies under model conditions (especially TEM, but also XPS) for the understanding of structural changes under bar-level DRM conditions. We followed a two-pronged approach: on the one hand, we verified that DRM activity is obtained in both scenarios (summarized in the new Figure 10) and on the other hand, we clarified that the salient feature of transient Ni oxidation and La2NiO4 formation as observed in the model studies is also observed during bar-level DRM operation. To do so, we performed quenching experiments under bar-level conditions along the reaction axis, where the catalytic features suggested the presence of NiO or metallic Ni and ex situ examined them by TEM. Indeed, the same correlation was found. This new result has been jointly included in the new Figure 10. This was done with respect to your own comment "In particular, Reviewers 1 and 3 raise important points regarding the disconnect between conditions in the TEM and in a DRM atmosphere that must be well addressed during this round of revision" and "Ni reduction and oxidation".

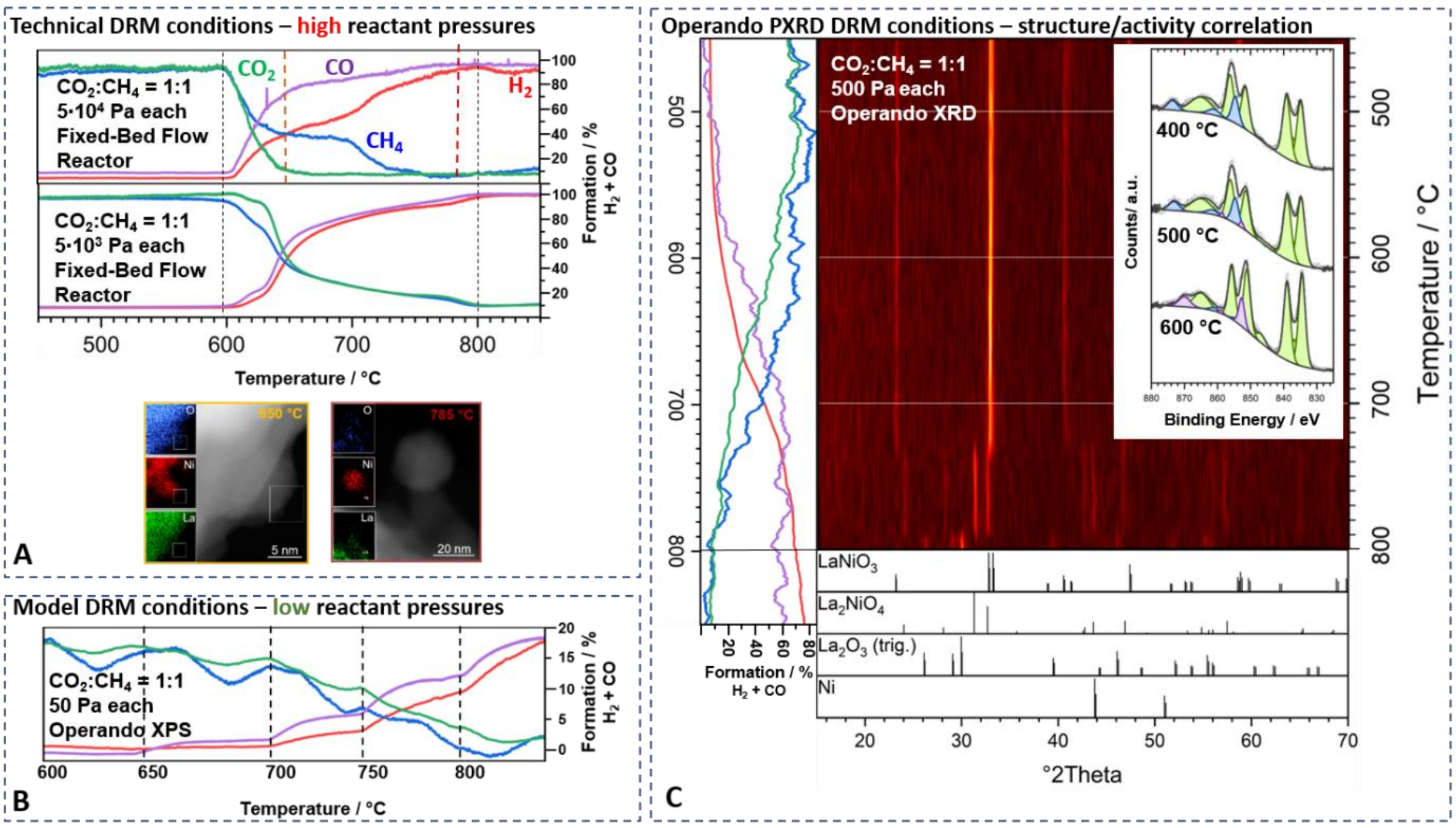

Figure R1/Figure 10: Validation of catalytic DRM profiles over $LaNiO_3$ from technical to model conditions.

(ii) We have also added new operando XPS data to follow the conditions in the TEM experiments (new Figure 2, 7, Figures S3, S15 and S17) to verify the dynamic oxygen chemistry under vacuum and hydrogen reduction conditions, (Figure 2, 7 and S8) the role of

CO_2 in Ni oxidation (Figure S17) and in vacancy quenching (Figure S15). Additional TEM was carried out to provide input into Ni oxidation (new Figure 9) and into the $LaNiO_3$-La_2NiO_4 transformation. This refers to your own comment on "new analysis"

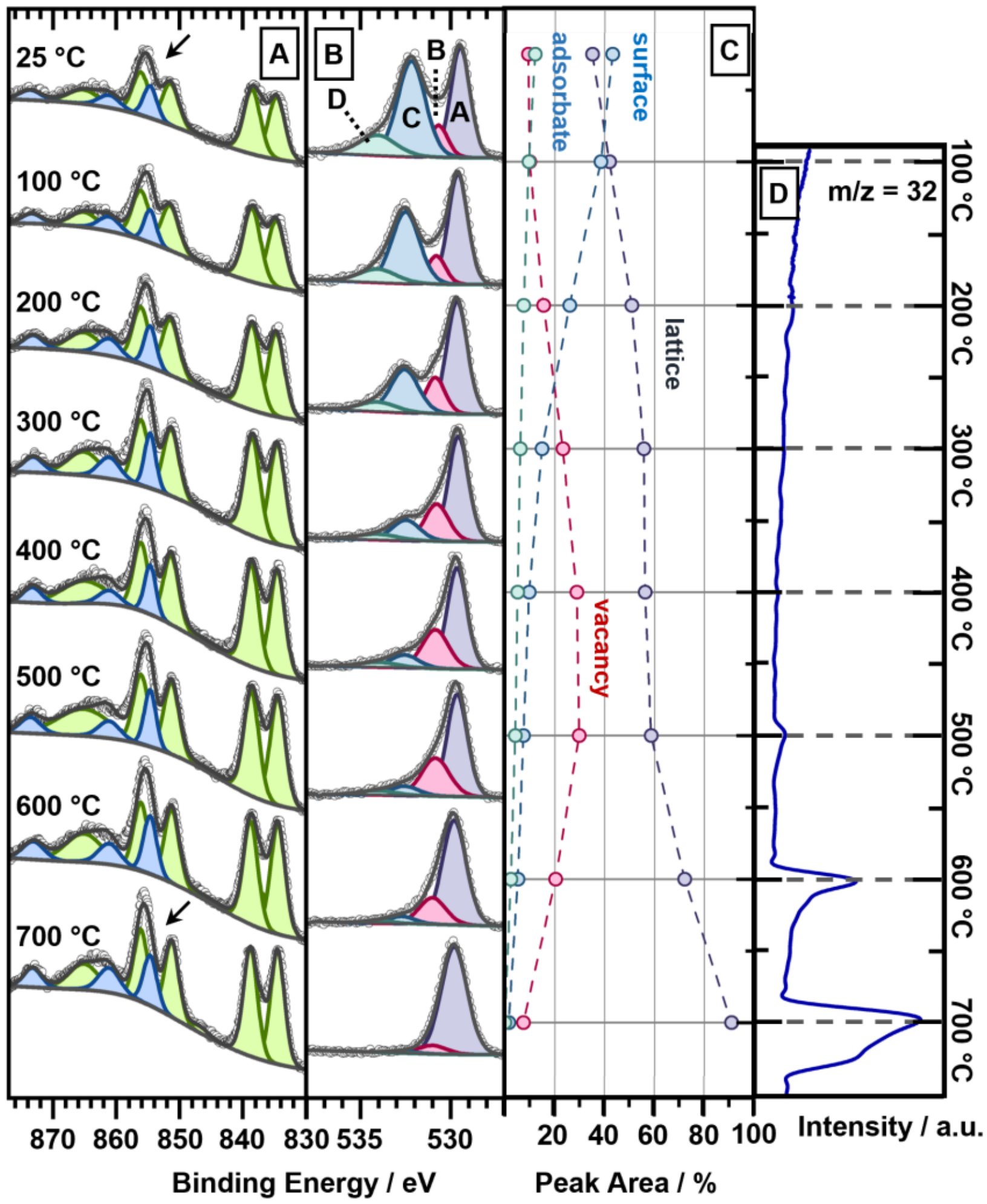

Figure R2/ Figure 7: Surface-near oxygen chemistry monitored by operando X-ray photoelectron spectroscopy during annealing in vacuo at base pressure of 10^{-6} Pa.

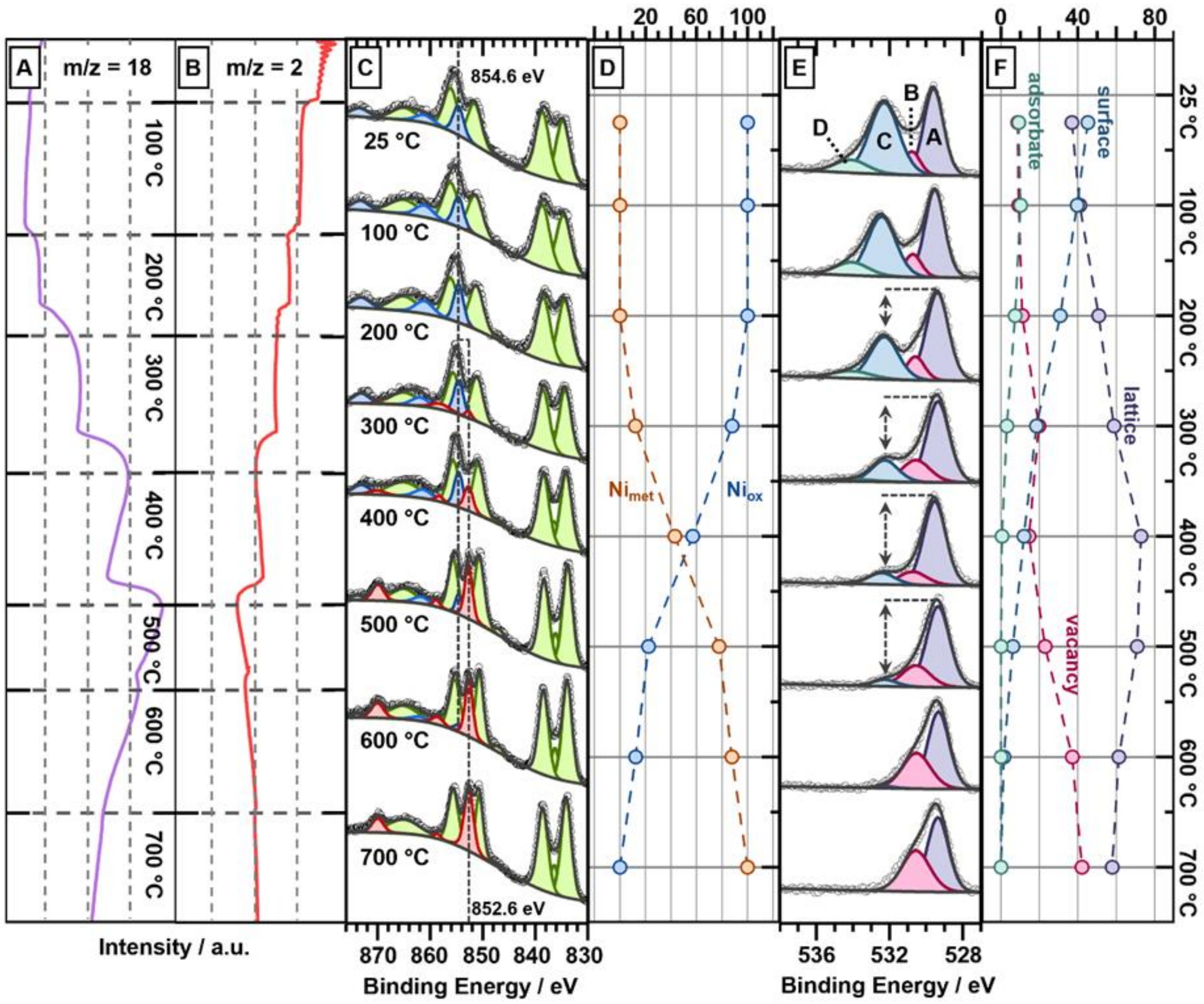

Figure R3/ Figure S3: Temperature-dependent evolution of the surface electronic structure of $LaNiO_3$ during hydrogen reduction at selected temperatures between 300 °C and 700 °C monitored by in situ X-ray photoelectron spectroscopy.

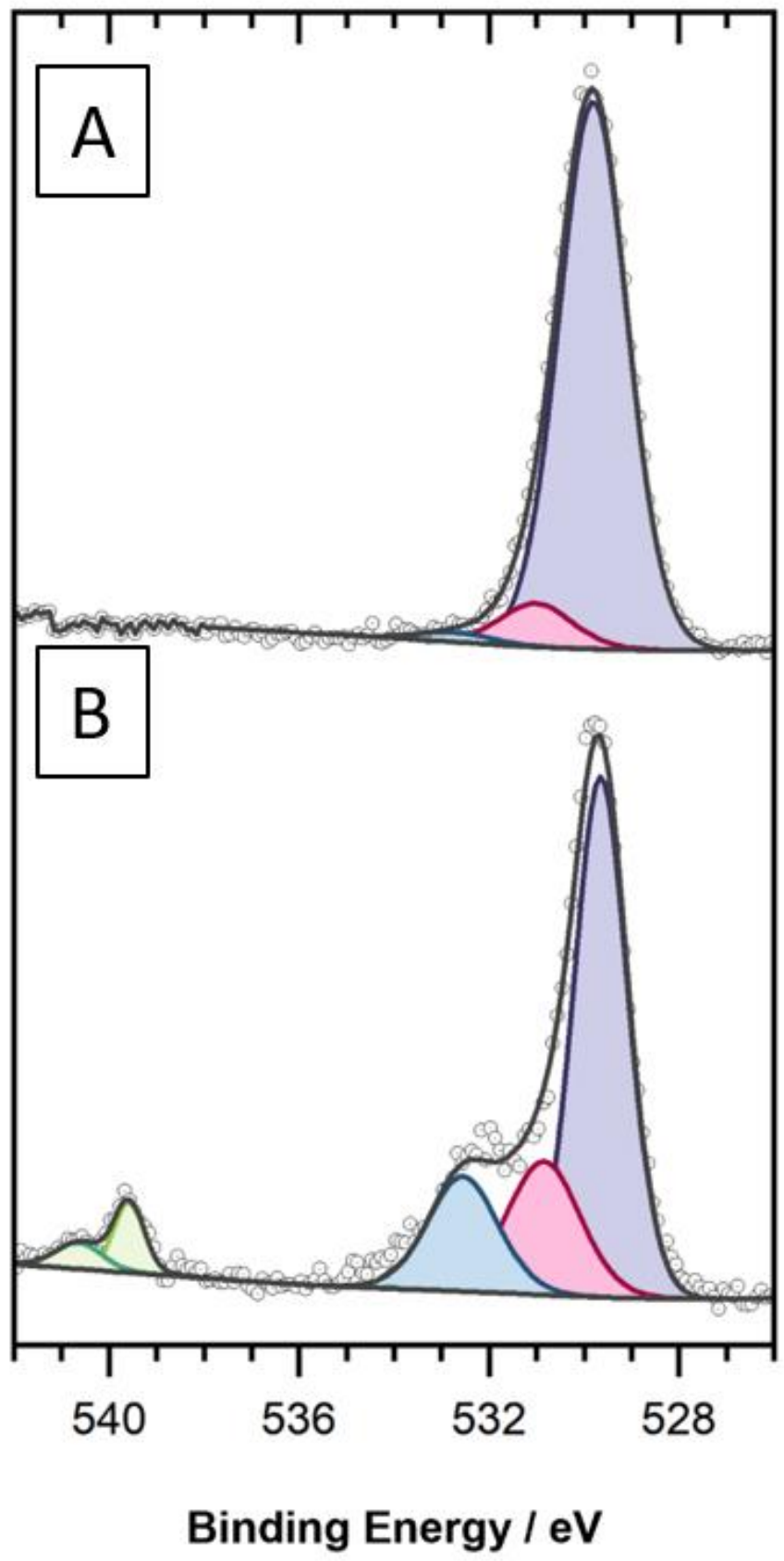

<u>Figure R4/ Figure S15</u>: High-resolution O 1s XP spectra after 700 °C annealing *in vacuo* (Panel A, corresponding to the bottom-most spectrum in Figure 7) in comparison to after a treatment in 20 Pa O_2 for 10 min at 25 °C (Panel B). Assignment of the individual oxygen species as in Figure 7. The oxygen component at 540 eV refers to gas-phase oxygen species.

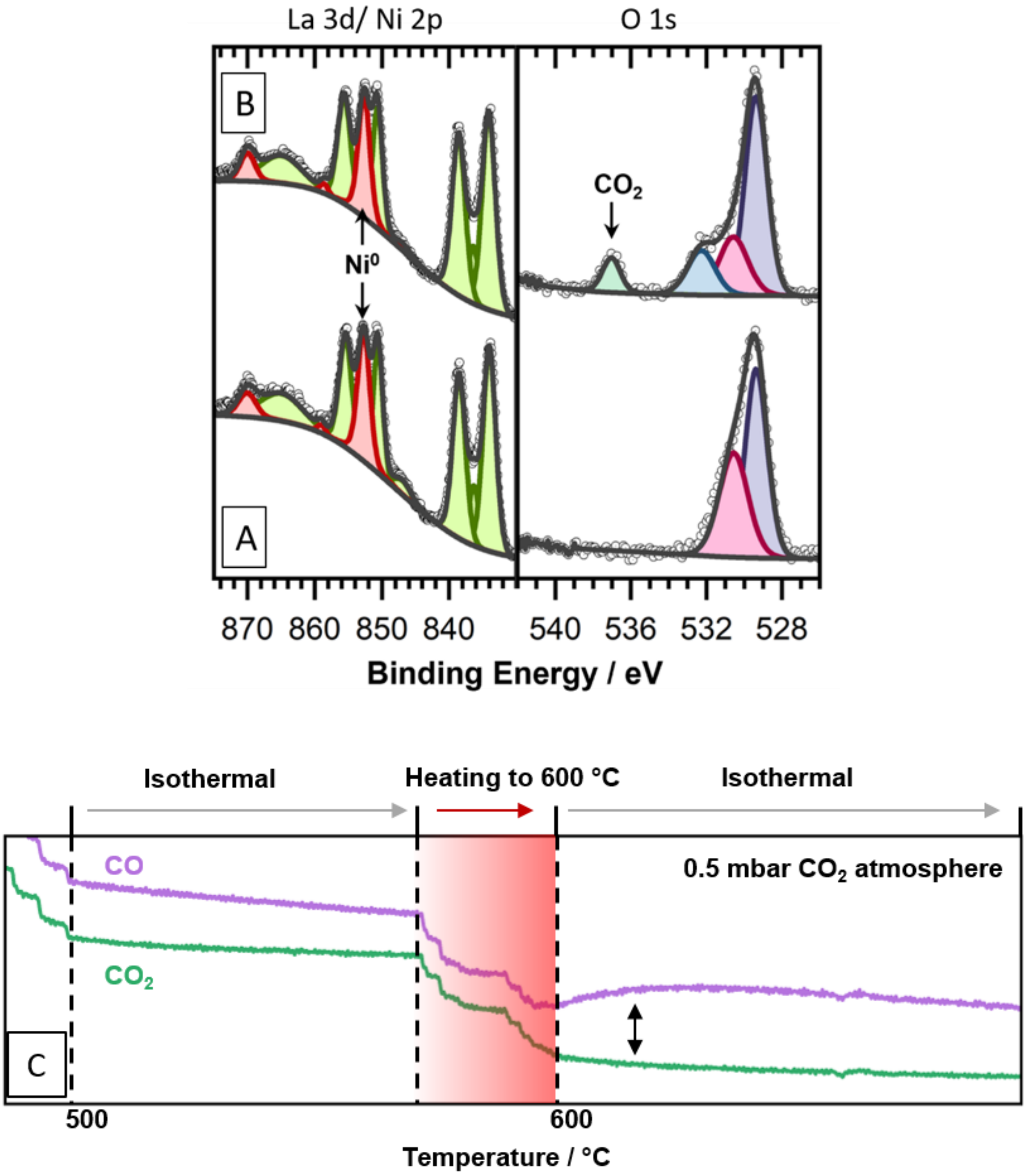

Figure R5/ Figure S17: Oxygen vacancy quenching by CO_2 after H_2 reduction.

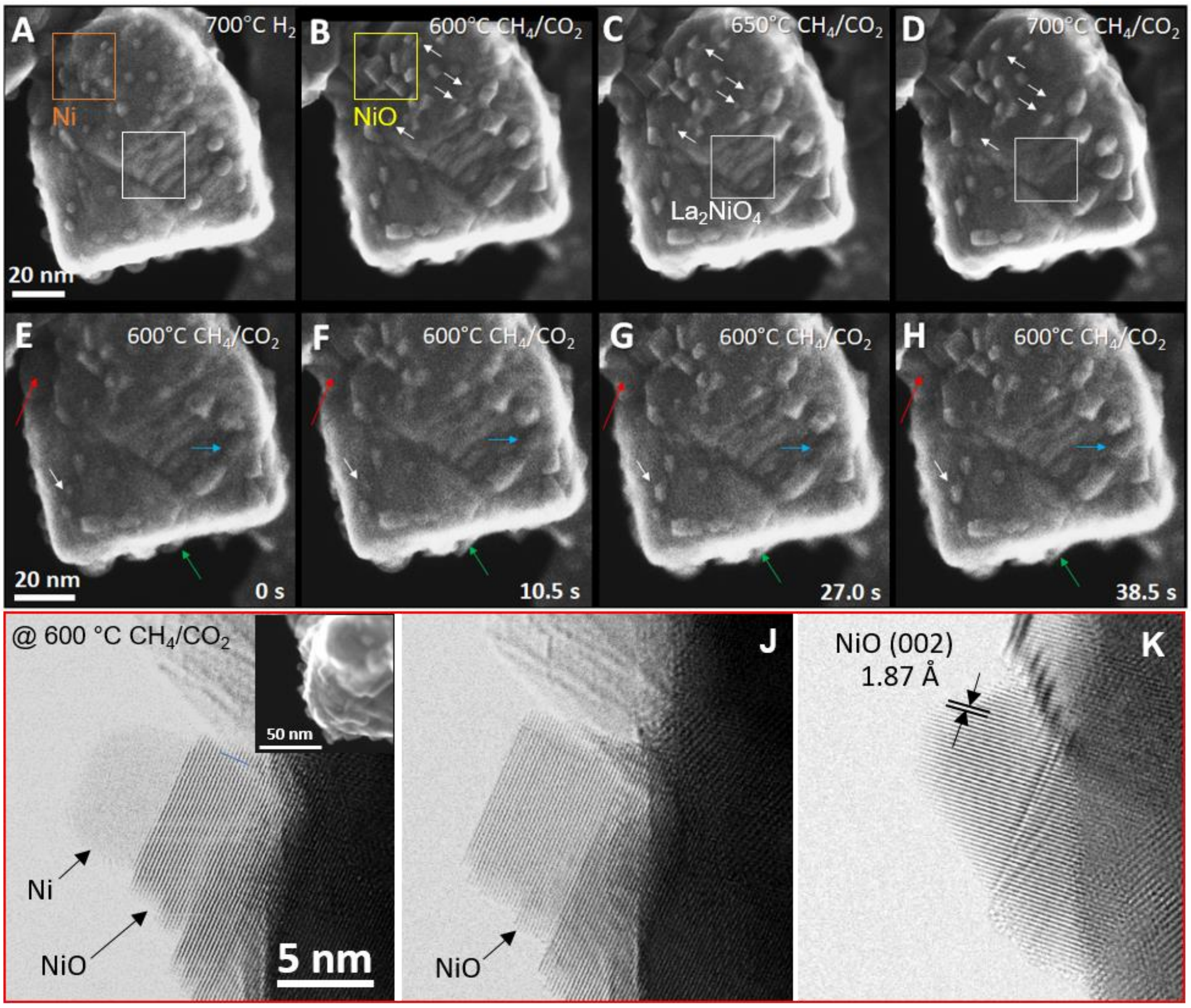

Figure R6/ Figure 9: Ni oxidation monitored by In situ STEM-SE imaging series of $LaNiO_3$ evolution after hydrogen reduction and subsequent exposure to CH_4:CO_2 = 1:1, Gas pressure: 10 Pa.

(iii) With respect to the discussion about phase stability, we have made it more clear, that the phase stabilities of NiO vs Ni, the transient role of La2NiO4 and the validity of the entire decomposition pathway from LaNiO3 into Ni +La2O3 is provided over several pressure regimes. To do so, we have moved the operando XRD data into the new Figure 10.

(iv) With respect to the substantial input into the reaction mechanism, we now have provided several key findings by combined information from different in situ and operando structural and spectroscopic techniques, whose validity is verified from simplified model studies to technical operation conditions. These include foremost: 1) the transient formation of NiO and its correlation to DRM activity, 2) the role of CO2 in vacancy quenching, but not Ni oxidation and 3) Ni oxidation is entirely done by reactive oxygen from not-yet-decomposed

$LaNiO_3/La_2NiO_4$ or 4) the apparent similarities of structural and chemical evolution in vacuo and under DRM conditions.

As a general introductory remark to the point-to-point response, we note the following:

1) Due to the incorporation of a large body of additional experiments especially by in situ X-ray photoelectron spectroscopy, we have decided to split the first authorship between the co-authors Pengfei Cao and Christoph Malleier.

2) As especially reviewer 3 rightly demanded a stronger focus on how the in situ TEM experiments implicate the catalytic properties, we decided to re-structure the manuscript to make the recurrent theme and the storyline more clear.

a) The results are now centered around two treatments (reduction vs. DRM/ in vacuo), which makes the structure-activity correlation more clear. To bolster the results from in situ TEM experiments, we have added corroborating complementary results using other methods, such as additional operando X-ray photoelectron spectroscopy or X-ray diffractometry.

b) This necessarily involved moving some parts dealing with in situ vacuum experiments from the Supporting Information back to the manuscript, to make especially the role of NiO formation and the strong correlation of vacuum and DRM experiments better visible.

c) The manuscript will be slightly longer, but to account for that, we merged some Figures together to make the storyline more cohesive.

d) The new sub-section 3.3. with its new Figure 10 is entirely devoted to address the issue of transferring the results from in situ electron microscopy to explanation of catalytic properties. We particularly focused here also on transferring the DRM activity from model low Pa to bar-level DRM operation under realistic conditions.

3) In order to provide consistent operando/ in situ XPS analysis, we have re-measured the entire hydrogen in situ XPS section. As the data quality is much better and thus, now allows to extract the Ni oxidation state from the overlapping La 3d/ Ni 2p region, we have removed the according data of the Ni 3p region and included the evolution of the O 1s region for direct comparison to the vacuum spectra shown in Figure 7. Figure R3 displays these operando XPS data.

REVIEWER COMMENTS

Reviewer #1

Major Comments

1. As emphasized in my first-round review, establishing a clear connection between in-situ TEM observations and realistic catalytic conditions is essential for demonstrating the broader relevance of this study. The authors are therefore encouraged to explicitly specify the exact experimental conditions for each in-situ experiment directly (gas pressure, gas composition and flow conditions, heating rate, temperature holding time etc.) in the main figures or text, rather than indicating only the type of gas atmosphere. Providing this information in a transparent and systematic manner is for enabling comparison with practical DRM conditions.

Answer and action taken:

We have added the exact experimental conditions in the main figures captions and text accordingly.

Methods part: "Details of the in situ heating procedure and the number of repeated individual experiments are provided in Fig. S26 and Table S3. We used a temperature profile in which the temperature increased continuously from 200 to 800 °C at a heating rate of 10 °C min^{-1}. After each 50 °C increment, the specimen was held for 10-30 min, images were also collected at 50 °C step intervals."

2. The authors' effort to bridge the pressure gap through a combination of in-situ TEM, in-situ XPS, and intermediate-pressure DRM experiments is important. The manuscript argues that NiO formation plays a crucial role during the DRM process. In this context, it is essential to clarify whether NiO can still exist as a relevant intermediate phase under realistic catalytic conditions.

Answer and action taken:

We have re-measured $LaNiO_3$ under DRM conditions to again verify the temperature region, where the dip, which we associate with intermediate NiO formation, occurs. Based on these data (in corroboration with previous measurements under similar conditions), we have performed quenching experiments, i.e., we have selected three different temperatures (650 °C, 680 °C and 785 °C), where the reaction was stopped by re-cooling. To exclude any possibility of parasitic (i.e., non-reactive formation of oxidised Ni), the samples were sealed

under inert gas and accordingly studied by analytical STEM measurements to verify the presence of oxidised Ni particles. Indeed, formation of oxidized Ni was observed in the sample at 650 °C, but metallic Ni at 785 °C, where the reaction is almost at 100 % conversion. The new TEM data have been included in the new Figure 10

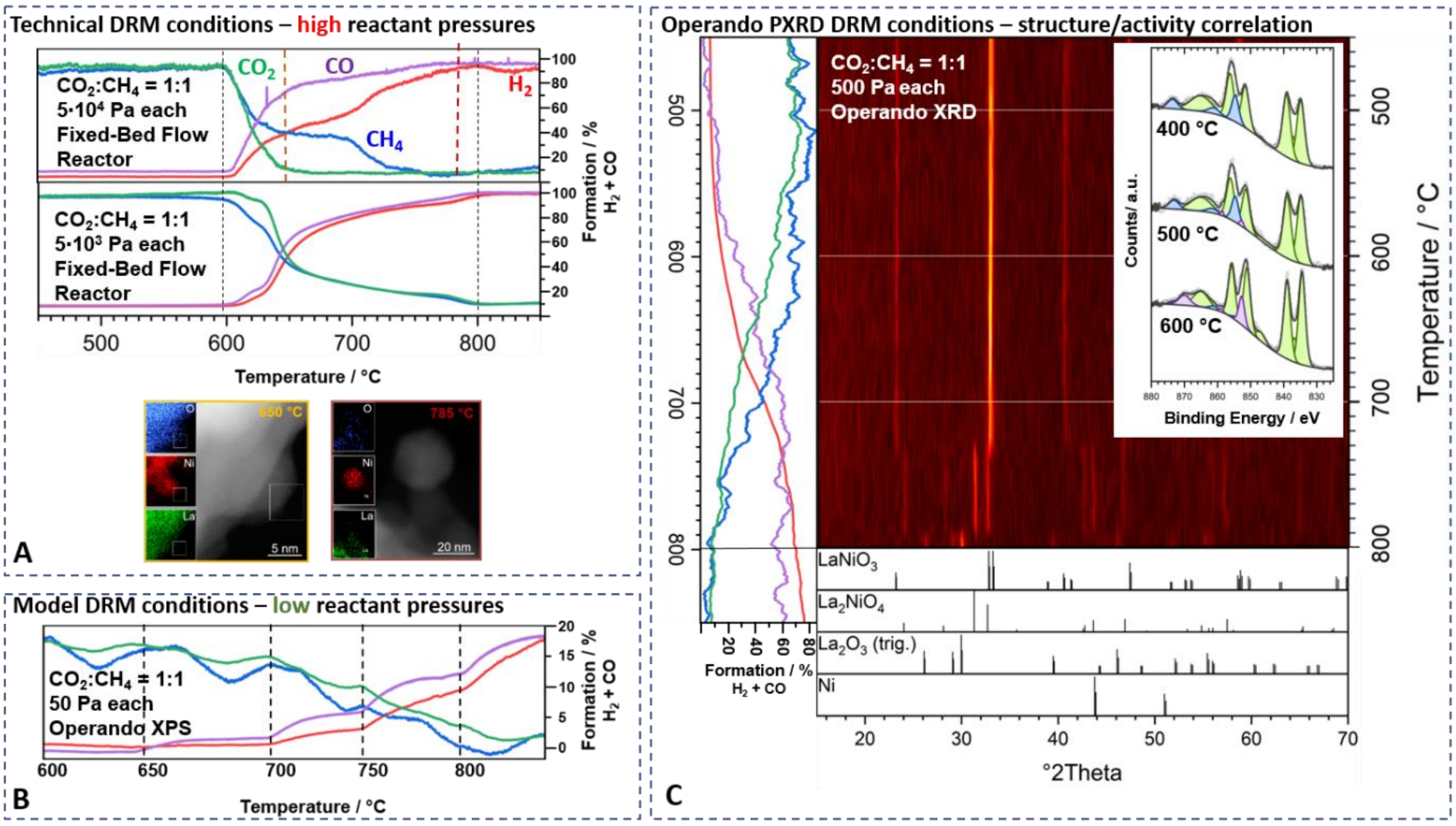

<u>Figure R1/Figure 10</u>: Validation of catalytic DRM profiles over $LaNiO_3$ from technical to model conditions and verfication of transient NiO formation under bar-level DRM conditions.

3. Related to the previous point, the lifetime of intermediate phases represents a key aspect that must be clarified when relating low-pressure in-situ observations to real catalytic operation. Under in-situ environment, the weaker reducing environment may stabilize intermediate states such as NiO or La2NiO4, allowing them to be directly observed. In contrast, under practical DRM conditions with much higher reactant partial pressures, the reducing potential of the gas mixture is significantly enhanced. This raises the question of whether these intermediate states are rapidly bypassed and therefore have a negligible influence on catalytic performance, or whether they persist long enough to affect activity, selectivity, or stability. Clarifying this issue would substantially enhance the mechanistic relevance and overall impact of the manuscript.

<u>Answer and action taken:</u>

We thank the reviewer for pointing that issue out and the answer is two-fold:

a) In fact, for bar-level catalysis, by in situ X-ray synchrotron-based diffractometry we have shown that the respective intermediates (i.e., Ni, NiO and La_2NiO_4) indeed play a crucial role in a full explanation of catalytic DRM activity. A clear structure-activity correlation has been established. See ref.23 of the main manuscript.

b) We have added the corresponding in situ X-ray diffractometry results at the lowest Pa level possible in our lab-based experiments in a more focused discussion to bridge the pressure gap. As seen in the new Figure 10 (and by comparison with ref.23), the qualitative trends in the low Pa regime and at bar-level catalysis are the same. To provide the most direct structure-activity correlation, we have performed true *operando* in situ XRD measurements (see also new Figure 10, Figure R1 above), which reveal that also the correlation between structure and catalytic properties in the low Pa-regime is the same as for the bar-level experiments. For the new section 3.3, we have set up a Figure 10 with combined information from catalysis (from model UHV conditions to bar-level catalysis, TEM and operando XRD).

Minor Comments

4. Ni is known to be highly susceptible to oxidation. Even under high-vacuum conditions (~10^{-6} Pa), adsorbed oxygen species on inert substrates such as SiN_x can oxidize nanoscale Ni particles. The authors should therefore more rigorously exclude alternative oxygen sources or, ideally, provide direct evidence for lattice oxygen participation in the observed Ni oxidation processes.

Answer and action taken:

We have added experiments directly focused at resolving that issue by in situ X-ray photoelectron spectroscopy and discussed them in the paper accordingly.

a) Experiment 1: We have added an in situ X-ray spectroscopy experiment cycle, where the reactive oxygen component is removed by vacuum heating up to 700 °C (Figure R2) and subsequently partially refilled by re-oxidation experiments in 20 Pa O_2 without the formation of metallic Ni (Figure R4). We show this additional experiment in Figure 7 and Figure S15. This directly proves the high dynamics of the reactive oxygen from $LaNiO_3$, which acts as the oxygen source for Ni particle oxidation.

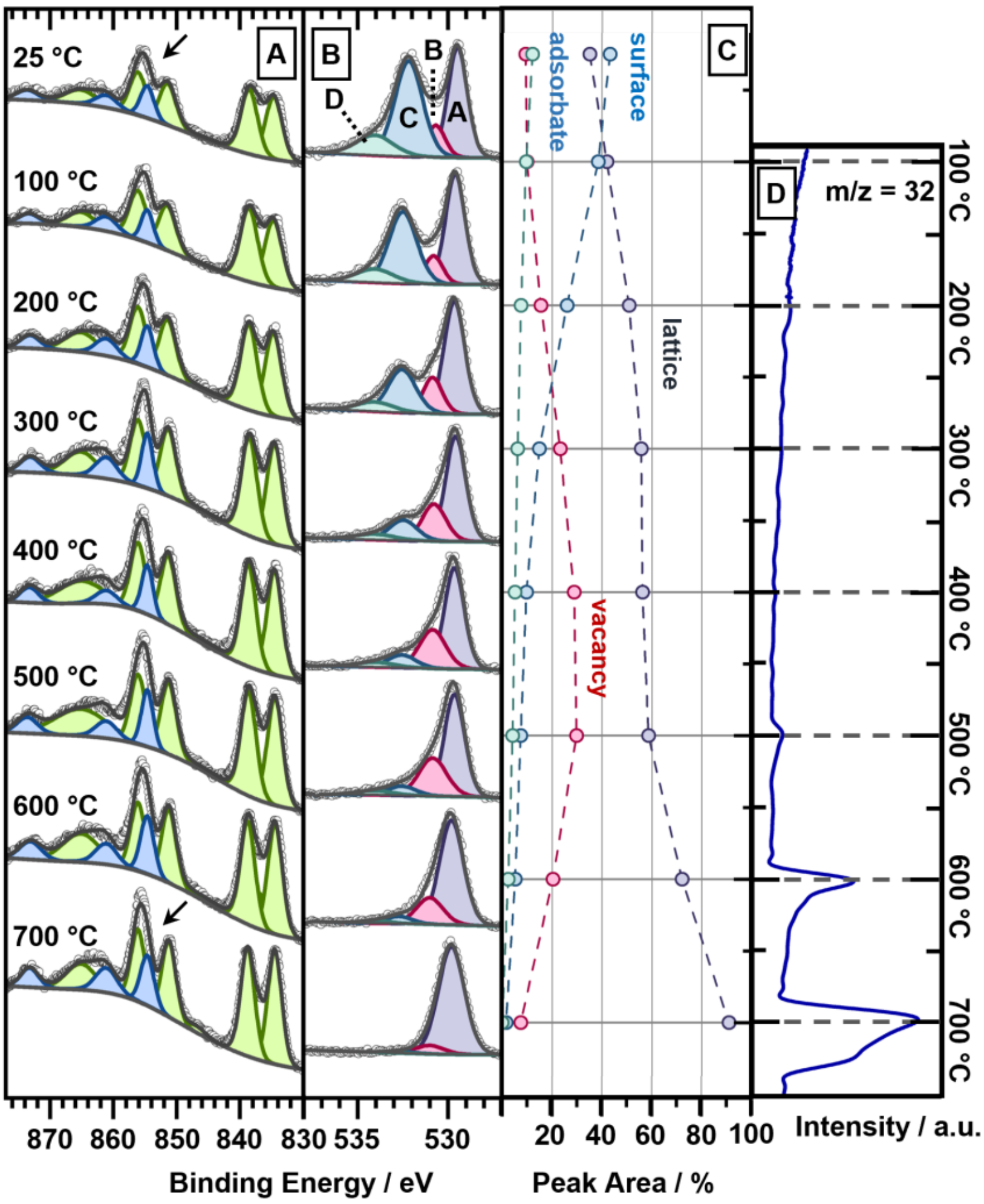

<u>**Figure R2/ Figure 7**</u>: Surface-near oxygen chemistry monitored by *operando* in situ X-ray photo-electron spectroscopy during annealing *in vacuo* at at base pressure of 10^{-6} Pa. The La 3d/ Ni 2p and the O 1s region are shown in Panels A and B. The bottom-most spectrum refers to a treatment in 20 Pa O_2 after the highest vacuum annealing temperature. Panel C: Evolution of the individual oxygen components fitted to the O 1s peak. Panel D: Simultaneously collected mass signal of m/z = 32.

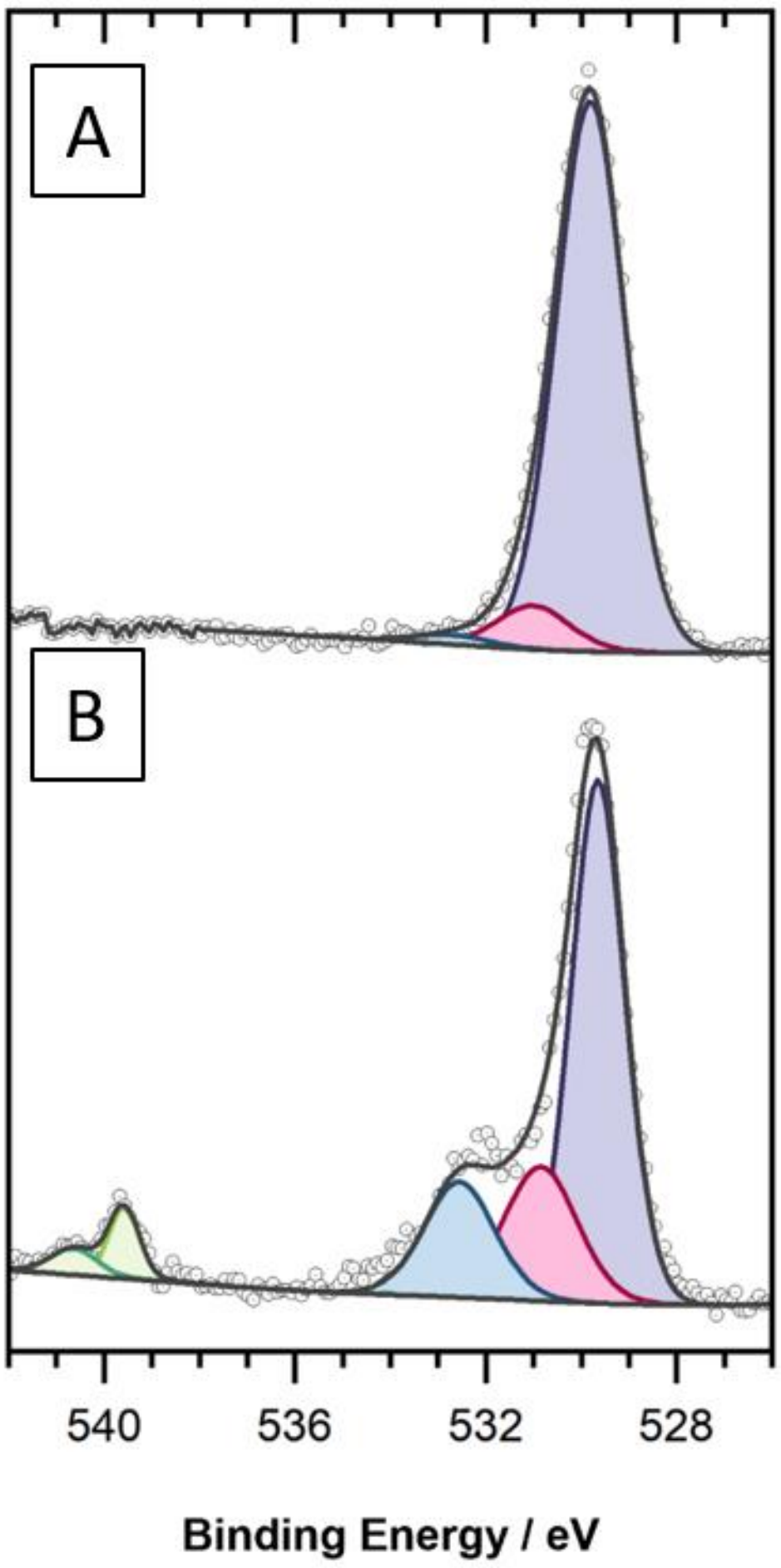

Figure R4/ Figure S15: High-resolution O 1s XP spectra after 700 °C annealing *in vacuo* (Panel A, corresponding to the bottom-most spectrum in Figure R2) in comparison to after a treatment in 20 Pa O_2 for 10 min at 25 °C (Panel B). Assignment of the individual oxygen species as in Figure R3.

b) **Experiment 2**: To exclude substantial participation of CO_2 in oxidation of the Ni particles, we pre-treated a sample in 20 Pa hydrogen at 700 °C to yield metallic exsolved Ni particles with removed O surface component (i.e., the oxygen component that is affected at first by reduction in hydrogen) and subjected it to 50 Pa pure CO_2 up to 600 °C. No substantial oxidation of Ni takes place, as seen in Figure R5/ new Figure S17. However, the results also indicate that CO_2 is capable of partially refilling the hydrogen-induced oxygen vacancies, a necessary prerequisite for CO_2 activation and carbon removal chemistry. The combined information from the above-mentioned experiments leads us directly to the conclusion, that reactive oxygen in surface-near layers is mainly responsible for the oxidation of the exsolved Ni particles and the role of CO_2 is vacancy quenching (and carbon removal via the inverse Boudouard reaction).

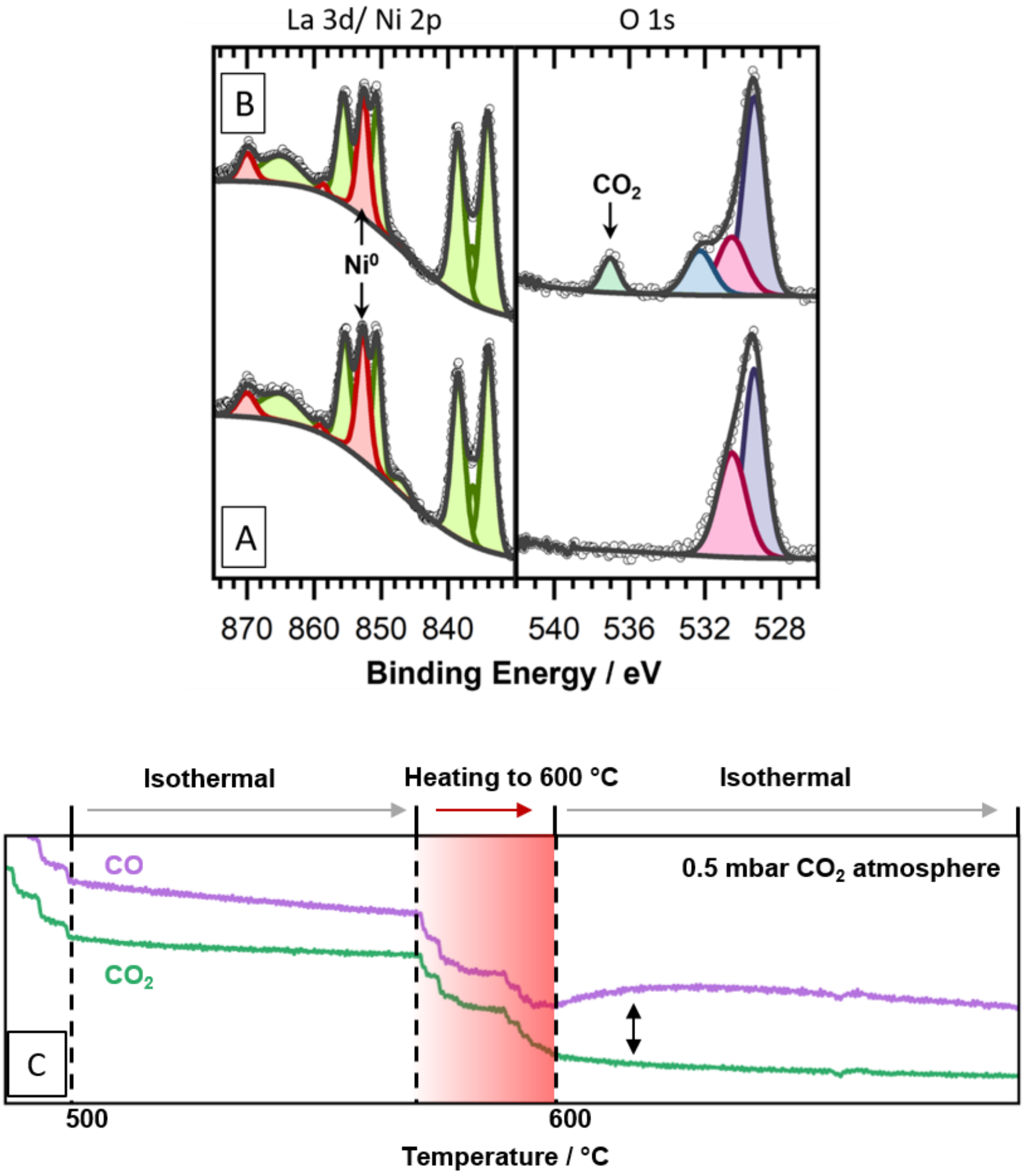

Figure R5/ Figure S17: High-resolution La 3d Ni 2p and O 1s XP spectra after reduction in 10 Pa H_2 (Panel A, cf. last spectrum in Figure 2, Panel A) and treatment in 50 Pa CO_2 at 700 °C (Panel B). CO_2 was admitted at 25 °C and spectra were taken in 100 °C steps up to 700 °C. The displayed spectrum is representative for all spectra. The feature at 537 eV refers to gas-phase CO_2. Assignment of the oxygen components as in Figure 2).

5. For clarity and consistency, the pressure units used in the Supporting Information should be unified with those in the main text, preferably using Pa.

Answer and action taken:

The pressure units have been amended to Pa accordingly.

6. The manuscript would benefit from a more detailed discussion of how the observed dynamic structural behavior impacts DRM performance. At present, this aspect is addressed only briefly

in the conclusion (e.g., “As this highly dynamic behaviour clearly occurs at temperatures, where DRM is usually carried out, it is expected to have a high impact on DRM operation.”). The authors are encouraged to expand this discussion and more explicitly connect the observed structural dynamics to catalytic activity, stability, and reaction pathways in DRM.

Answer and action taken:

As reviewer 3 rightly demanded a stronger focus on how the in situ TEM experiments implicate the catalytic properties, we decided to re-structure the manuscript to make the recurrent theme and the storyline more clear.

a) The results are now centred around two treatments (reduction vs. DRM), which makes the structure-activity correlation more clear. To bolster the results from in situ TEM experiments, we have added corroborating complementary results using other methods, such as additional operando/ in situ X-ray photoelectron spectroscopy or X-ray diffractometry. The Results sections are:

“3.1. Activation of $LaNiO_3$ under hydrogen reduction conditions

Temperature-dependent formation of Ni particles

Dynamics of emerged Ni nanoparticles and structural evolution of perovskites in H_2

3.2. Activation of $LaNiO_3$ under methane dry reforming conditions conditions

Thermally-induced Ni particle formation and Ni oxidation behavior upon vacuum annealing

Reaction-induced Ni particle formation and oxidation behaviour by heating in a 1:1 CO_2:CH_4 DRM mixture

Ni nanoparticle dynamics and structural evolution of $LaNiO_3$ by heating in a 1:1 CO_2:CH_4 DRM mixture after hydrogen pre-activation

3.3. Correlation of structure and morphology evolution to DRM activity: transfer from model studies to technically relevant DRM operation”

b) This necessarily involved moving some parts dealing with in situ vacuum experiments from the Supporting Information back to the manuscript, to make especially the role of NiO formation and the strong correlation of vacuum and DRM experiments visible.

c) The manuscript will be slightly longer, but to account for that, we merged some Figures together to make the storyline more cohesive.

d) The new sub-section 3.3. is entirely devoted to address the issue of transferring the results from in situ electron microscopy to explanation of catalytic properties. We particularly

focused here also on transferring the DRM activity from model low Pa to bar-level DRM operation under realistic conditions.

e) The conclusions section has been amended accordingly

Reviewer #2

The authors have addressed all the points raised. I support publication.

Answer and action taken:

We thank the reviewer for the positive assessment

Reviewer #3

The authors have conducted novel TEM analyses under a DRM atmosphere, and the high-quality images effectively reveal structural changes in the material. However, despite the exceptional quality of the analyses, the derived results show limited significance in explaining catalytic properties, and the authors have not achieved their intended research objectives. To strengthen this work, the authors should either (1) modify the TEM results to directly explain catalytic characteristics, (2) support the TEM observations with complementary analytical techniques, or (3) revise the overall direction of the paper.

Answer and action taken:

As reviewer 3 rightly demanded a stronger focus on how the in situ TEM experiments implicate the catalytic properties, we decided to re-structure the manuscript to make the recurrent theme and the storyline more clear.

a) The results are now centred around two treatments (reduction vs. DRM), which makes the structure-activity correlation more clear. To bolster the results from in situ TEM experiments, we have added corroborating complementary results using other methods, such as additional operando/ in situ X-ray photoelectron spectroscopy or X-ray diffractometry. The Results sections are:

“3.1. Activation of $LaNiO_3$ under hydrogen reduction conditions

Temperature-dependent formation of Ni particles

Dynamics of emerged Ni nanoparticles and structural evolution of perovskites in H_2

3.2. Activation of $LaNiO_3$ under methane dry reforming conditions conditions

Thermally-induced Ni particle formation and Ni oxidation behavior upon vacuum annealing

Reaction-induced Ni particle formation and oxidation behaviour by heating in a 1:1 CO_2:CH_4 DRM mixture

Ni nanoparticle dynamics and structural evolution of $LaNiO_3$ by heating in a 1:1 CO_2:CH_4 DRM mixture after hydrogen pre-activation

3.3. Correlation of structure and morphology evolution to DRM activity: transfer from model studies to technically relevant DRM operation"

b) This necessarily involved moving some parts dealing with in situ vacuum experiments from the Supporting Information back to the manuscript, to make especially the role of NiO formation and the strong correlation of vacuum and DRM experiments visible.

c) The manuscript will be slightly longer, but to account for that, we merged some Figures together to make the storyline more cohesive.

d) The new sub-section 3.3. is entirely devoted to address the issue of transferring the results from in situ electron microscopy to explanation of catalytic properties. We particularly focused here also on transferring the DRM activity from model low Pa to bar-level DRM operation under realistic conditions.

e) The conclusions section has been amended accordingly

1. a) The in situ TEM analysis under DRM atmosphere in Figure 8 shows a significant gap with the actual catalytic activity trends and is inadequate to explain catalyst behavior. The catalytic activation observed at 650 °C is not evident in the in-situ TEM results.

Answer and action taken:

We would like to stress here as an introductory remark, that the real and final activation upon treatment in a DRM reaction mixture as NiO reduction and La_2NiO_4 decomposition under the experimentally possible conditions for in situ electron microscopy cannot be observed for thermodynamic reasons due to the accessible oxygen partial pressure regime. Even under

hydrogen reduction under STEM conditions, decomposition of $LaNiO_3$ beyond Ni and La_2NiO_4 cannot be observed.

To validate the importance of the structural studies under model conditions (especially TEM, but also XPS) for the understanding of structural changes under bar-level DRM conditions. We followed a two-pronged approach: one the one hand, we verified that DRM activity is obtained in both scenarios (summarized in the new Figure 10) and on the other hand, we clarified that the salient feature of transient Ni oxidation and La2NiO4 formation as observed in the model studies is also observed during bar-level DRM operation. To do so, we performed quenching experiments under bar-level conditions along the reaction axis, where the catalytic features suggested the presence of NiO or metallic Ni and ex situ examined them by TEM. Indeed, the same correlation was found. This new result has been jointly included in the new Figure 10.

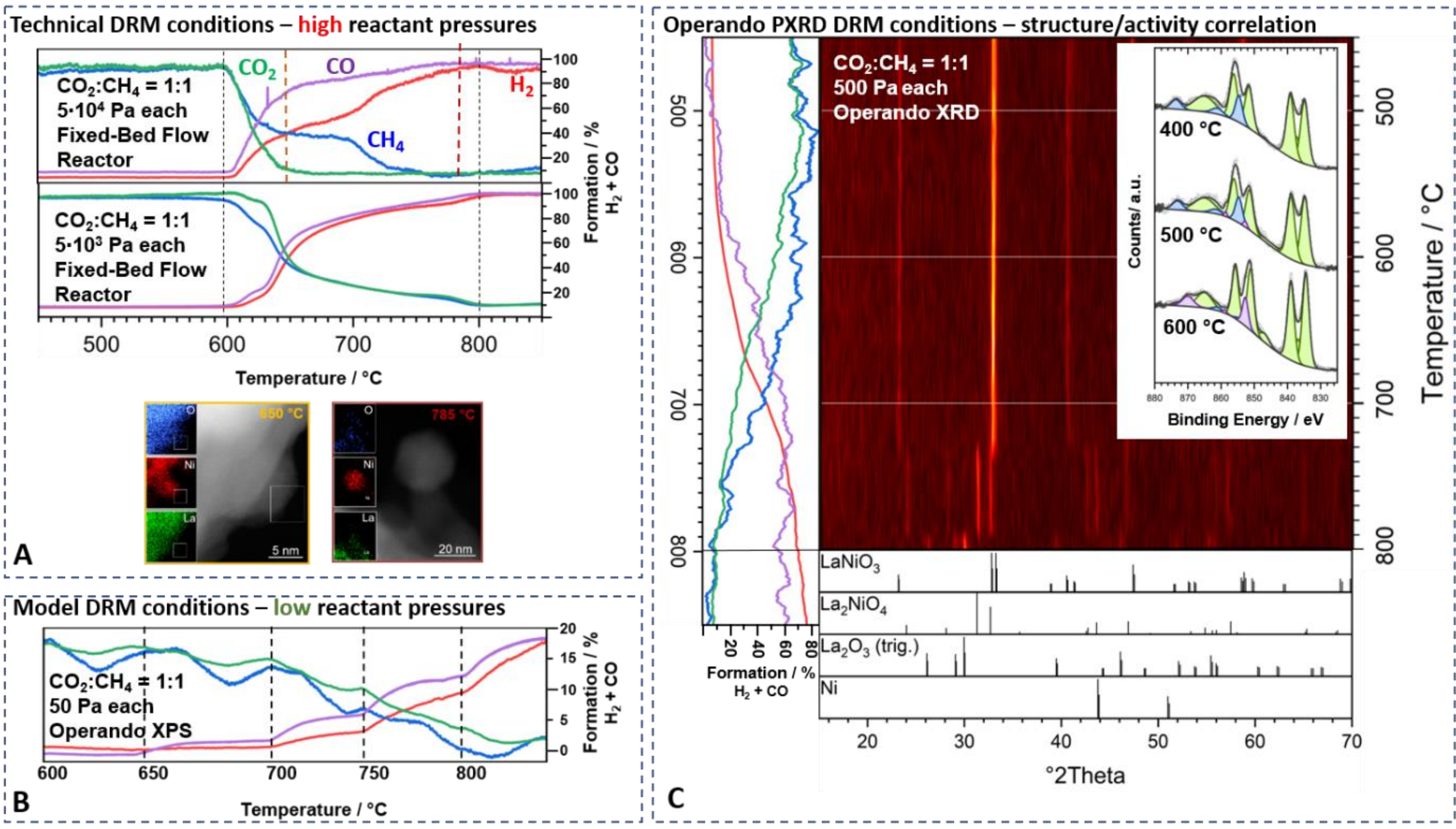

<u>Figure R1/Figure 10</u>: Validation of catalytic DRM profiles over $LaNiO_3$ from technical to model conditions and verification of transient NiO formation under bar-level DRM conditions.

<u>For the first time, due to the directed in situ TEM measurements highlighted in the present work, oxidised Ni was detected in vacuum heating experiments, under DRM conditions and after bar-level DRM operation by HRTEM, FFT analysis and EDX mapping</u>.

Our BF STEM and its corresponding FFT results clearly show that the exsolved Ni particles are affected by oxidation and that Ni and NiO crystallites coexist. Finally, metallic Ni is completely oxidized to NiO by lattice oxygen, which has been confirmed by in situ XRD.

We have made the motivation more clear in the introduction of the revised version as "As the formation of Ni particles from $LaNiO_3$ perovskites is either a reduction-induced or a self-activated process that improves the catalytic performance during DRM[22], the missing link in fully understanding the decomposition process and the catalytic action of $LaNiO_3$ is the study of the early stages of partial $LaNiO_3$ decomposition into $LaNiO_3$, Ni particles and La_2NiO_4. In particular, the simultaneous emergence of the latter two rises the question of beneficial Ni particle size (distribution) and eventual Ni particle dynamics control and its effect on DRM activity. In addition, vacuum heating and catalytic DRM experiments have already indicated that intermediate oxidation of emerged Ni particles by reactive lattice oxygen from $LaNiO_3$ has a profound effect on the catalytic action. To answer these questions, separation of thermal, purely reduction-induced and DRM-triggered structural and morphological changes is imperative. The main goal, therefore, is to monitor and reveal different pathways of Ni formation from the perovskite as a function of the reduction potential of the gas atmospheres by a multitude of in situ methods and combine it with dedicated DRM experiments to relate structure, morphology, electronic structure and catalytic properties".

b) While the authors attribute this to the reduction of oxidized Ni near 650oC, independent verification is necessary. The authors must provide ex situ post-reaction analyses or other complementary characterizations to directly demonstrate that Ni reduction occurs.

Answer and action taken:

We would like to draw the attention to Panel C of the new Figure 10, where this information is provided by operando XRD: at 800 °C in the most active state in a 500 Pa CO_2 + 500 Pa CH_4 reaction mixture, the catalyst is composed of metallic Ni + La_2O_3.

We clarified that the salient feature of transient Ni oxidation and La2NiO4 formation as observed in the model studies is also observed during bar-level DRM operation. To do so, we performed quenching experiments under bar-level conditions along the reaction axis, where the catalytic features suggested the presence of NiO or metallic Ni and ex situ examined them by TEM. Indeed, the same correlation was found. This new result has been jointly included in the new Figure 10 (see above)

2. The authors argue, based on Figure 8, that Ni formed via H_2 treatment at 700 °C oxidizes to NiO within seconds under DRM conditions at 600 °C, attributing this oxidation to CO_2 dissociation. Given the authors' assertion that no coke formation occurs, any CO_2 dissociation event must stoichiometrically yield CO gas as a byproduct. Since the authors explicitly attribute the onset of DRM activity at 650 °C to the subsequent reduction of NiO to metallic Ni, the CO gas generated from the initial CO_2-driven oxidation should logically be detected prior to the catalytic activation. However, Figure S12 fails to exhibit any such CO evolution trend preceding the activity onset. The absence of this pre-activation CO signal directly contradicts the hypothesis that CO_2 dissociation is the oxygen source for the rapid Ni oxidation observed at 600 °C.

Answer and action taken:

We thank the referee for pointing out this discrepancy. In fact, we have performed additional in situ XPS experiments to verify the role of CO_2 in oxidation of the emerged Ni particles (Figure R6 below). We have performed a hydrogen reduction (20 Pa H_2, 700 °C) cycle, and subjected the sample to the same conditions as in Figure 8 (at 600 °C), but pure CO_2 without CH_4 to maximise the potential oxidation effect of CO_2. In fact, CO_2 is not able to oxidise Ni under these experimental conditions. The La 3d/ Ni 2p region is hardly affected by the CO_2 treatment. However, the O 1s signals reveal that CO_2 is capable of partially replenishing the oxygen vacancies induced by prior hydrogen reduction. This behaviour is essential for CO_2 activation and subsequent carbon removal via the inverse Boudouard reaction under DRM conditions.

The source for formation of NiO during the DRM experiments as discussed in the context of new Figure 9 is mostly the reactive oxygen from $LaNiO_3$ (which has not yet decomposed) and not CO_2, which is activated at perovskite vacancy sites. During the CO_2 re-oxidation in situ XPS experiments, CO formation was observed in simultaneously collected mass spectra. As in pure CO_2 no coke formation occurs, CO_2 dissociation yields CO gas as anticipated (and now verified) activation product. Under DRM conditions, the situation is more complex due the presence of CH_4 and its decomposition product carbon. Therefore, the CO evolution of CO_2 activation/ carbon removal is more entangled and does not necessarily occurs sequentially.

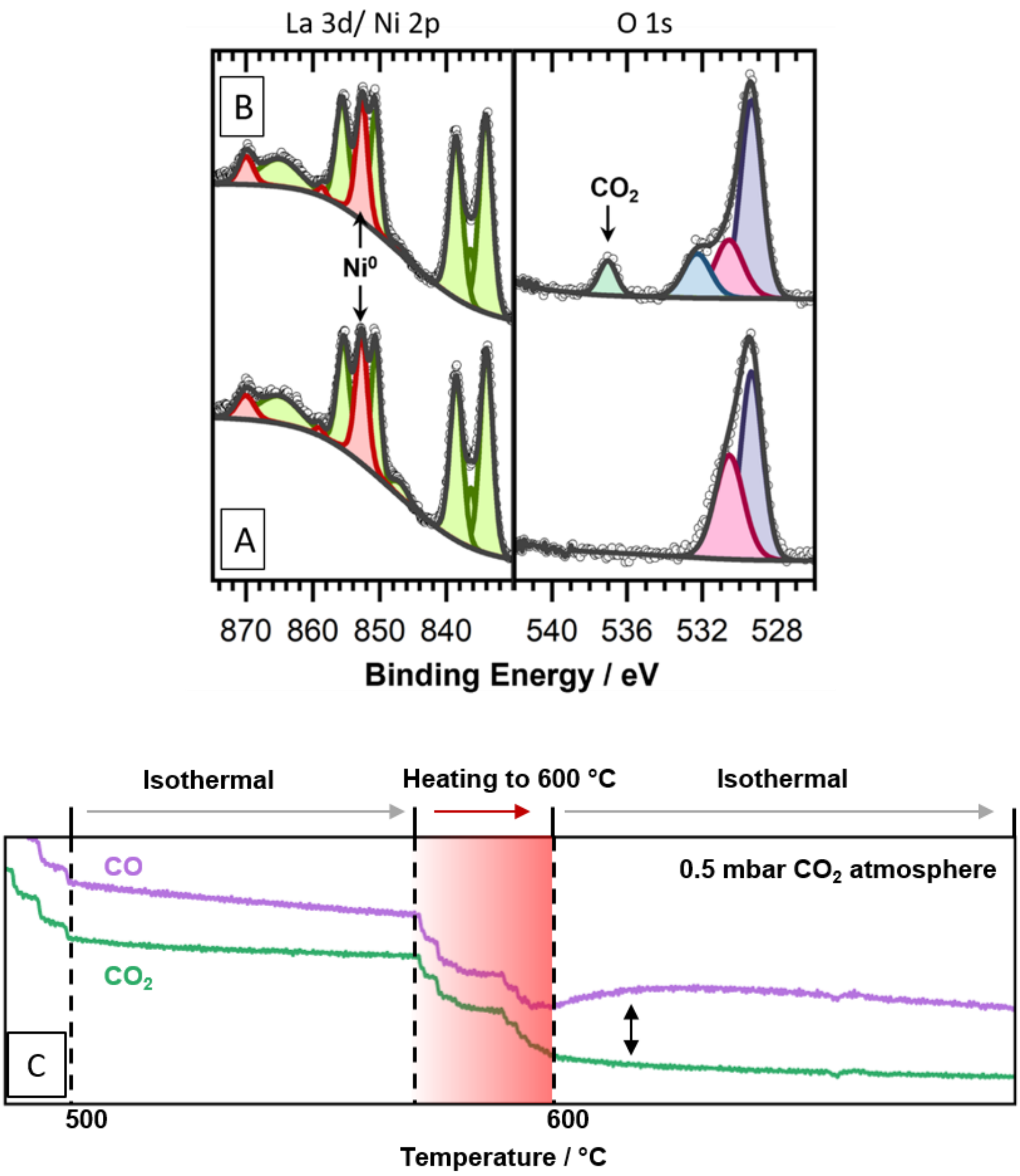

Figure R5/ Figure S17: High-resolution La 3d Ni 2p and O 1s XP spectra after reduction in 10 Pa H_2 (Panel A, cf. last spectrum in Figure 2, Panel A) and treatment in 50 Pa CO_2 at 700 °C (Panel B). CO_2 was admitted at 25 °C and spectra were taken in 100 °C steps up to 700 °C. The displayed spectrum is representative for all spectra. The feature at 537 eV refers to gas-phase CO_2. Assignment of the oxygen components as in Figure 2).

3. The preliminary experiments conducted under H₂ and CH_4/CO_2 atmospheres (Figure 3) correlate poorly with the subsequent DRM results (Figure 8). In Figure 3, the parent La_2NiO_4 phase with a stepped surface is maintained even at 800 °C. However, in Figure 8, surface smoothing occurs at only 700 °C under DRM conditions. One would expect more pronounced dissolution at the higher temperature, so this apparent contradiction requires clarification.

Answer and action taken:

We thank the reviewer for pointing out that contradiction. In fact, the statement about surface smoothing in connection with now Figure 9 at 700 °C is wrong. In essence, one can clearly see, that between 600 °C and 700 °C, the surface morphology remains the same. We have rewritten and cut the respective parts in the revised version.

4. The discussion of structural changes identified in the TEM analysis needs to be strengthened. The results section primarily presents observations, and the discussion relies heavily on references rather than providing original mechanistic insights. While the study is TEM-focused, a more detailed analysis of the observed phenomena would help readers better understand the underlying mechanisms. In particular, the mechanism by which Ni particles undergo dissolution in the H₂ atmosphere at 700 °C for 10 minutes should be explained more thoroughly.

Answer and action taken:

To make the recurrent theme more visible, we have restructured the results and discussion section and now present scientific blocks that get input not only from TEM, but also from other methods, such as in situ XPS or in situ XRD. In particular, we set up a new chapter, that is entirely devoted to structure-activity correlation, i.e. how the structural and morphological changes can be related not only to catalytic data but also across different pressure regimes. For the restructuring of the revised version we refer to the detailed answer above point 1.

As for the dissolution of Ni particles in H_2, we admit that the word “dissolution” is misleading here and “fragmentation” would be more accurate.

5. Additional analyses are required to support and validate the TEM observations:

1) The formation of the La2NiO4 phase should be discussed in greater detail (Figure 3). To precisely identify the temperature at which the phase transition occurs, the authors should conduct additional TEM analyses at intermediate temperatures (e.g., 600 °C) to demonstrate the transition from the $LaNiO_3$ phase to the La_2NiO_4 phase, providing more concrete evidence of the temperature-dependent phase evolution.

Answer and action taken:

We have included additional analyses in Figure S16, where we can establish a link between the formation of surface steps during our DRM experiments (Fig. S16E) and the formation of the La_2NiO_4 phase (Fig. S16F and H). The formation of the La_2NiO_4 phase can thus be followed by assessing the surface morphology changes in the temperature range between 600 and 700°C.

The structure of the layered Ruddlesden–Popper compounds La_2NiO_4 can be described as an alternating stacking of a $LaNiO_3$ perovskite layer and a LaO layer with a NaCl structure, suggesting that a structural transformation of the phases within a layered structure may be occurring. We have carried out numerous in-situ experiments under DRM conditions and conducted further new experiments in which we were able to observe, for example, the oxidation of Ni to NiO. However, a continuous analysis of the transformation of LaNiO□ to La□NiO□ under high-resolution conditions was not possible despite several attempts, as areas of the sample rotate away from the electron beam due to the extensive microstructural transformation, making a continuous high-resolution investigation virtually impossible.

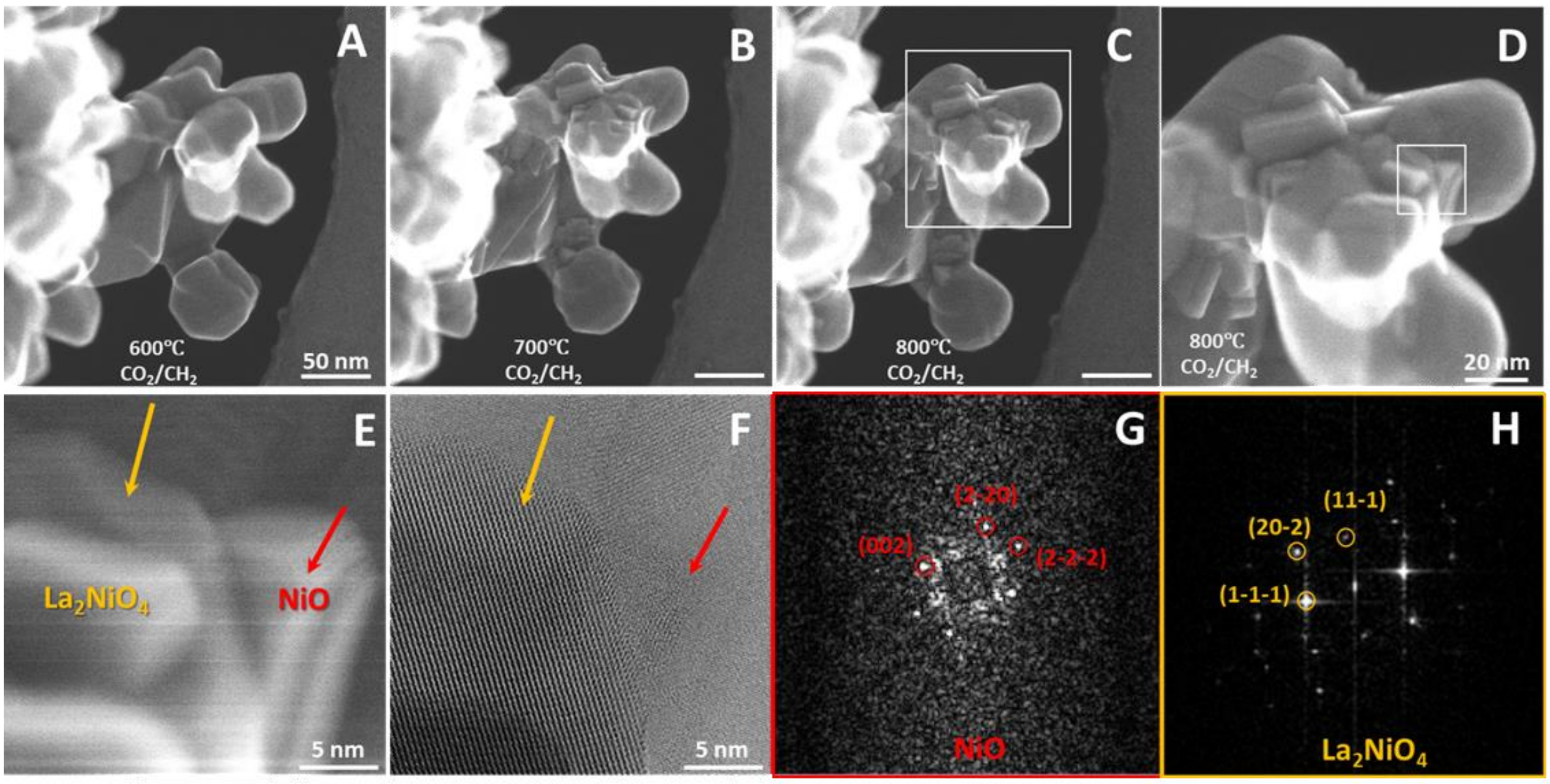

Fig R7/ Fig. S16: Formation of facetted NiO during heating in a DRM mixture without hydrogen pre-reduction. An In situ STEM-SE imaging series is shown during heating $LaNiO_3$ in a CO_2:CH_4 = 1:1 mixture at 600 ℃ (Panel A), 700 ℃ (Panel B), 800 ℃ (Panel C and D). Panel E: Enlarged SE image of the region in the white box in Panel D; Panel F: corresponding BF image of the region in white box in Panel D; Panel G and H: FFT analysis of the area indicated by a yellow and red arrow shoeing the presence of the La_2NiO_4 and NiO phase, respectively.

2) While faceted NiO particles appear to play a critical role in catalytic behavior, the evidence that these particles are indeed NiO is insufficient (Figure 8). Additionally, the mechanism by which the stepped surface disappears at elevated temperatures must be clarified; specifically, whether this represents a phase transition to LaNiO□ or another structural transformation.

Answer and action taken:

We have now re-performed the analysis contained in Figure 9 (see below) and by time-resolved HRSTEM verified, that the facetted particles are indeed NiO. The formation mechanism is in accordance with ref. https://doi.org/10.1016/j.scriptamat.2022.114660

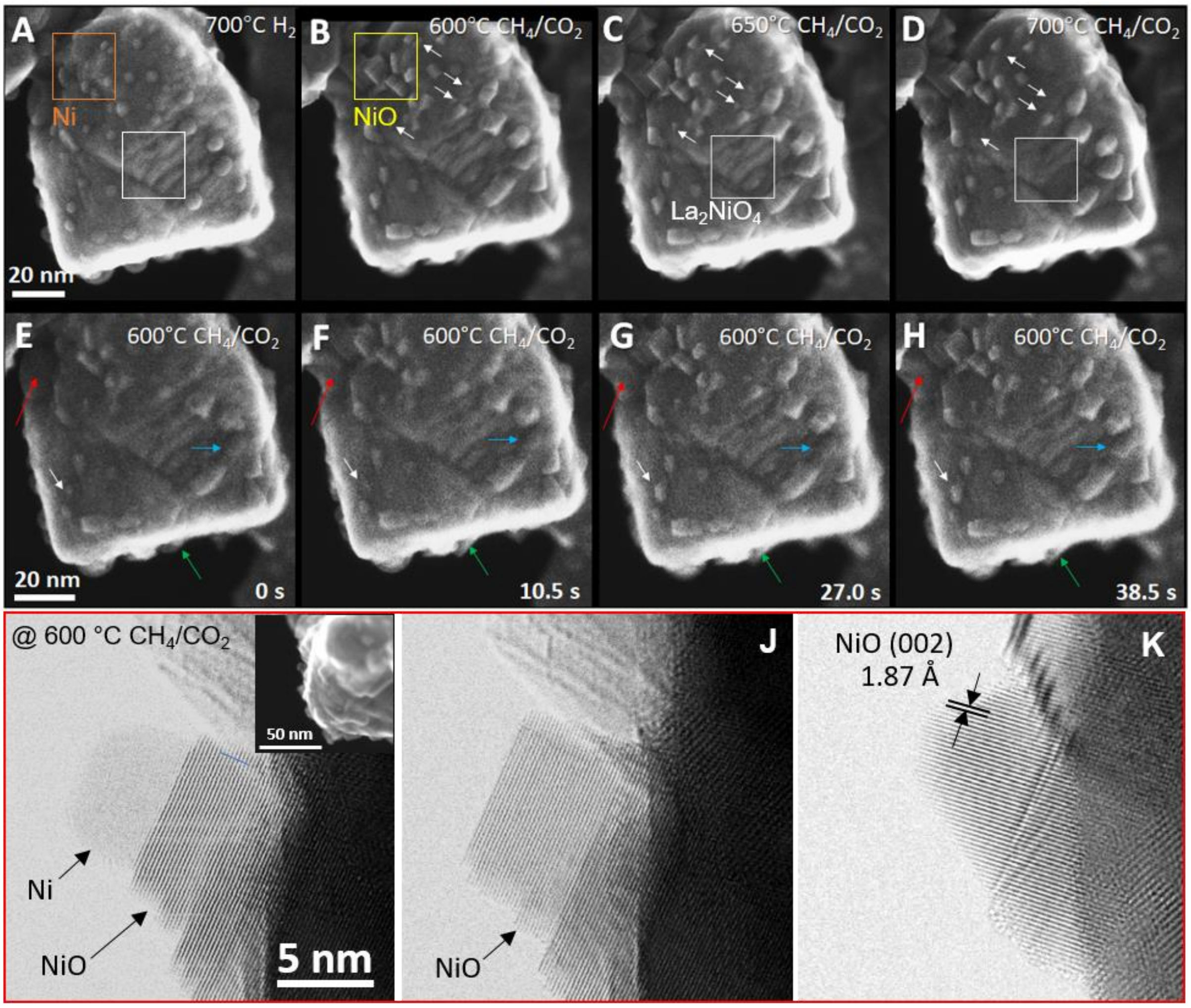

Figure R6/ Figure 9: Ni oxidation monitored by In situ STEM-SE imaging series of $LaNiO_3$ evolution after hydrogen reduction and subsequent exposure to CH_4:CO_2 = 1:1

Reviewer #1

I appreciate the authors' substantial efforts in revising the manuscript and in providing additional experimental evidence, including operando/in situ XPS, PXRD, and quenching experiments under DRM conditions. The revised manuscript is considerably improved, and the added sections help to connect the in situ microscopy observations with catalytic DRM performance. Due to numerous changes, the manuscript needs further refinement, I therefore recommend that the manuscript undergo minor revision before acceptance.

Some Editorial Issues

The manuscript contains numerous typographical and grammatical errors. Examples include: "obserbvations" "formaiton" "ftting""atomically-resloved" "amd" "accompaying""showin" "lattic" "strenghtens" "esssentially" "stipuletd priciples" "isNiO" "over the the 3D perovskite" et. al.

Answer: We have thoroughly reviewed the paper for typos and grammatical errors. In addition, a native English speaker has revised the manuscript to ensure correct grammar.

Reviewer #3

The authors have made substantial improvements to the manuscript by incorporating additional in-situ characterizations, including AP-XPS and in-situ XRD, which significantly strengthen the mechanistic interpretation beyond the previous version that relied heavily on in-situ TEM. The systematic comparison under multiple atmospheres and temperature conditions provides useful insights into the dynamic surface evolution of the exsolution catalyst under practically relevant reaction environments. In addition, the inclusion of fixed-bed catalytic reaction results and in-situ XRD measurements under conditions closer to realistic operation effectively addresses concerns regarding the gap between model in-situ analyses and practical catalytic conditions.

Overall, the manuscript has been considerably improved, and most of the major concerns raised

previously have been adequately addressed. I believe the work is now suitable for publication in Nature Communications.

One remaining suggestion is related to the presentation of the manuscript. The current version has become quite lengthy, and in several sections the manuscript attempts to describe nearly all possible information obtained from the measurements, which occasionally interrupts the logical flow of the discussion. I encourage the authors to further streamline the text and focus more concisely on the key findings directly relevant to the central mechanism and conclusions.

Answer: We have performed the following:

- The abstract has been focused
- The discussion of the in situ XRD analysis has been moved to the SI, with only a short summary sentence in the relevant section
- The sentence "Further evidence of the locally entangled appearance of both phases is provided during heat-induced structural reconstruction. Gaps between adjacent host particles at 200 °C are filled by Ni and O at 750 °C (Fig. S13), where faceted La_2NiO_4 appeared, accompanying the emergence of Ni as demonstrated by SE images." Has been moved to the SI, as it discusses a topic that is already shown in the main manuscript.
